# Epigenetic signature of preterm birth in adult twins

**DOI:** 10.1186/s13148-018-0518-8

**Published:** 2018-06-27

**Authors:** Qihua Tan, Shuxia Li, Morten Frost, Marianne Nygaard, Mette Soerensen, Martin Larsen, Kaare Christensen, Lene Christiansen

**Affiliations:** 1 Epidemiology and Biostatistics, Department of Public Health, Faculty of Health Science, University of Southern Denmark, J. B. Winsløws Vej 9B, DK-5000 Odense, Denmark; 2 Unit of Human Genetics, Department of Clinical Research, University of Southern Denmark, Odense, Denmark; 3 Department of Endocrinology, Odense University Hospital, Odense, Denmark; 4 Department of Clinical Genetics, Odense University Hospital, Odense, Denmark

**Keywords:** Preterm birth, Twins, Epigenetics, Epigenome-wide association study, Adults

## Abstract

**Background:**

Preterm birth is a leading cause of perinatal mortality and long-term health consequences. Epigenetic mechanisms may have been at play in preterm birth survivors, and these could be persistent and detrimental to health later in life.

**Methods:**

We performed a genome-wide DNA methylation profiling in adult twins of premature birth to identify genomic regions under differential epigenetic regulation in 144 twins with a median age of 33 years (age range 30–36).

**Results:**

Association analysis detected three genomic regions annotated to the *SDHAP3*, *TAGLN3* and *GSTT1* genes on chromosomes 5, 3 and 22 (FWER: 0.01, 0.02 and 0.04) respectively. These genes display strong involvement in neurodevelopmental disorders, cancer susceptibility and premature delivery. The three identified significant regions were successfully replicated in an independent sample of twins of even older age (median age 66, range 56–80) with similar regulatory patterns and nominal *p* values < 5.05e−04. Biological pathway analysis detected five significantly enriched pathways all explicitly involved in immune responses.

**Conclusion:**

We have found novel evidence associating premature delivery with epigenetic modification of important genes/pathways and revealed that preterm birth, as an early life event, could be related to differential methylation regulation patterns observable in adults and even at high ages which could potentially mediate susceptibility to age-related diseases and adult health.

**Electronic supplementary material:**

The online version of this article (10.1186/s13148-018-0518-8) contains supplementary material, which is available to authorized users.

## Background

Preterm birth (PTB) or premature birth is defined as birth before 37 weeks of pregnancy. With a prevalence estimated from 5 to 18% in singleton pregnancies across 184 countries according to the World Health Organization and over 50% in twin pregnancies in the USA [1], PTB is a leading cause of perinatal mortality as well as long-term morbidity and health consequences. Survivors of PTB were subject to adaptive mechanisms that might be deleterious later in life, and are more susceptible to early on-set chronic diseases [2] including cardiovascular disease [3], metabolic disorders [4], respiratory complications [5] and mental and cognitive impairments [6]. Despite the strong epidemiological evidence, the molecular mechanisms and etiology behind these phenotypes have been poorly understood. Preterm infants are exposed to various stressful conditions in the peridelivery period, a critical stage for their organ development. Molecular mechanisms including epigenetic modification may have been involved in the adaptation to adverse environment which, in the long-run, could be detrimental to health [7–9]. It has been hypothesized that epigenetic modifications such as DNA methylation induced by PTB may lead to long-term consequences and increased susceptibility to adult-onset diseases [10–12].

Advantaged by the emerging new technology in genomic analysis of DNA methylation, epigenome-wide association studies (EWAS) have been done to look for DNA methylation markers of PTB in neonates [13–16] and have reported differentially methylated sites implicated in neural function [16], or with increased risk for adverse health outcomes later in life [13]. Notably, PTB-associated methylation patterns were also investigated in adolescents by Cruickshank et al. [14] and Simpkin et al. [15] in their longitudinal samples. Although relatively large numbers of CpG sites were found significantly differentially methylated in association with PTB at birth, they are largely resolved in adolescents in both studies. Nevertheless, persistent methylation differences were identified at ten CpG sites in the study by Cruickshank et al. [14] reflecting a lasting epigenetic effect of PTB.

The fact that PTB is associated with an increased risk of chronic diseases in adults suggests that it is of high importance to focus on the epigenetic signature of PTB that mediates the long-term health consequences. Given the high prevalence of PTB in twin pregnancies, epigenetic analysis of PTB in twins is therefore especially important and valuable for the health of the twin population and for the general population as well. Using relatively large numbers of adult twin samples for discovery (144 twins) and replication (350 twins), we conducted an epigenomic profiling of the DNA methylome to look for genomic sites and regions under epigenetic regulation in association with PTB in adult subjects.

## Methods

### The discovery samples

**Fig. 1:**
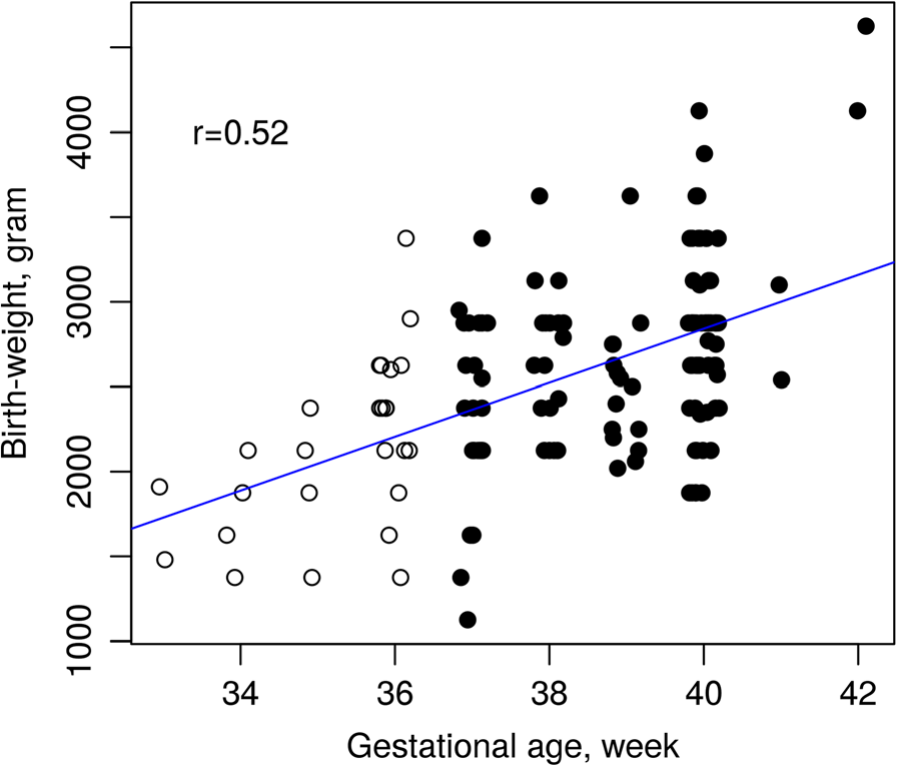
Scatter plot displaying the samples by gestational age and birthweight. PTB subjects tend to have low birthweight with a moderate correlation of 0.52

The discovery samples in this study consisted of 72 pairs of identical twins (144 individuals, 78 males and 66 females) aged 30 to 36 years with a median age of 33 (Table 1). Gestational ages were collected from the Danish Medical Birth Registry (DMBR) established in 1973. The median of gestational age was 39 weeks with a minimum of 33 and a maximum of 42 weeks. A total of 26 twins had their gestational ages < 37 weeks. The samples formed a subset (those born after 1973 when gestational age was recorded by DMBR) of 150 pairs of identical twins discordant for birthweight used in an EWAS by Tan et al. [17]. Figure 1 displays the samples by plotting individual gestational age against birthweight. There is a moderate correlation of 0.52 between gestational age (PTB indicated by empty spots) and birthweight. Although no significant association was found between birthweight and DNA methylation [17], we adjusted for individual birthweight in all our analyses.

**Table 1:** Descriptive statistics for the discovery and replication samples

Variables	Discovery	Replication
Age, year		
Median	33	66
Range	30–36	56–80
Gestational age, week		
Median	39	0 (weeks before term)
Range	33–42	0–8 (weeks before term)
Report method	DMBR	Midwife
Sample size		
Male	78	192
Female	66	158
Total	144	350
PTB	26	40 (more than 3 weeks before term)

*DMBR* Danish Medical Birth Registry

### Blood sampling and DNA extraction

Blood sampling and DNA preparation were described by Tan et al. [17]. In brief, ethylene di-amine tetra acetic acid (EDTA)-anticoagulated blood samples were collected. The blood was centrifuged at 1000*g* for 10 min, and buffy-coat was frozen in aliquots at − 80 °C. DNA was isolated from the buffy-coats using the salt precipitation method applying either a manual protocol or a semi-automated protocol based on the Autopure System (Qiagen, Hilden, Germany). Bisulphite treatment of 500 ng template genomic DNA was carried out with the EZ-96 DNA methylation kit (Zymo Research, Orange County, USA) following the manufacturer’s protocol.

### DNA methylation data

Genome-wide DNA methylation was analysed using the Illumina Infinium HumanMethylation450 Beadchip assay (Illumina, San Diego, CA, USA) at Leiden University Medical Center or at GenomeScan B.V., Leiden, The Netherlands. The array interrogates more than 485,000 CpG sites across and beyond gene and CpG island regions in the human genome. The laboratory experiment was conducted according to the array manufacturer’s instructions. Twins of each pair were processed together on the same array to minimize batch effect. The quality of DNA methylation data was controlled by calculating a detection *p* value defined as the proportion of samples reporting background signal levels for both methylated and unmethylated channels. The detection *p* was calculated using the free R package *minfi* (http://bioconductor.org/packages/release/bioc/html/minfi.html). Probes with detection *p* > 0.05 were dropped from subsequent analysis. In addition, we also removed CpG probes harbouring SNPs considering potential disruption on their methylation levels by heterozygous SNPs. As usual, we also dropped methylation data on sex chromosomes to focus on autosomal CpG sites. A total of 473,864 CpGs were available for subsequent analysis. Data normalization was done using the subset-quantile within-array normalization (SWAN) [18] implemented in *minfi*. At each CpG site, the DNA methylation level was summarized by calculating a “beta” value defined by the Illumina’s formula as β = M/(M + U + 100), where M and U are methylated (M) and unmethylated (U) signal intensities measured at the CpG site. We further removed CpGs with β < 0.05 [19] leaving 427,555 CpGs for statistical analysis. Both raw and processed DNA methylation data for the discovery samples have been deposited to the NCBI GEO database (http://www.ncbi.nlm.nih.gov/geo) under accession number GSE61496.

### The replication samples

The replication sample was comprised of 175 pairs of identical twins (350 individuals, 192 males and 158 females) aged 56 to 80 years with a median age of 66 (Table 1). The estimated weeks before term birth were provided by midwives with a range from 0 to 8 weeks. Preterm birth was defined as birth at least 3 weeks before term. A total of 40 twins were found as preterm births. Blood sampling, DNA extraction and methylation analysis were performed as described for the discovery samples. The methylation data were also normalized using the Subset-quantile Within Array Normalization (SWAN) [18]. Methylation “beta” values were likewise calculated using the Illumina’s formula.

### Estimating and adjusting cell composition

Cell composition in whole blood can change with age. Although the age range of our discovery samples was relatively short, that of the replication samples was 24 years (Table 1). For the discovery samples, we controlled for this issue by estimating and correcting for cell composition in each individual. We estimated cell composition for six blood cell types: CD8T, CD4T, natural killer cell, B cell, monocyte and granulocyte based on our measured DNA methylation data from whole blood using an approach proposed by Houseman et al. [20], using the R package *minfi*. For the replication samples, blood leukocyte subtypes (monocytes, lymphocytes, basophils, neutrophils and eosinophiles), counted using a Coulter LH 750 Haematology Analyser, were available. Missing blood cell counts were imputed by a modified version of the method supplied by PredictCellComposition (www.github.com/mvaniterson/predictcellcomposition). The effect of cell composition was then adjusted for by including the estimated proportion of each cell type as covariates in the regression analyses.

### Data analysis

The association between DNA methylation and PTB was investigated on both single CpGs and genomic regions through fitting linear models that regressed the level of DNA methylation on PTB status adjusting for sex, birthweight and cell compositions. Before fitting the models, DNA methylation *β*-values were transformed into *M* values using logit transformation to ensure normal or approximately normal distribution.

#### Single-CpG-based analysis

We applied a linear regression model with a robust sandwich variance estimator to regress the methylation *M* values on PTB status (preterm coded as 1 and term coded as 0), sex, birthweight and estimated cell compositions. The sandwich variance estimator was introduced to take into accounts the intra-pair twin correlation on DNA methylation. By estimating and testing the regression coefficient for PTB, we were interested in identifying differentially methylated CpG probes (DMPs) of PTB. The model was fitted using the *clubSandwich* package in R (https://cran.r-project.org/web/packages/clubSandwich). *P* values were adjusted for multiple testing by calculating the false discovery rate [21] (FDR) with genome-wide significance defined as FDR < 0.05.

#### Multiple-CpG-based analysis

On top of the single-CpG-based analysis, we further extended our analysis to multiple CpGs to look for differentially methylated genomic regions (DMRs) in association with PTB. This was done using the bumphunter approach introduced by Jaffe et al. [22] implemented in the R package *minfi*. The methylation *M* values were first regressed on sex, birthweight and estimated cell compositions. The residuals from the regression and PTB status were then submitted to the *bumphunter()* function in *minfi*. The approach assumes that the locus-specific estimates of regression coefficients (βs) are smooth along the strand of a chromosome and applies the loess smoothing technique to smooth coefficient βs within a pre-defined chromosomal region (300 base pairs in our analysis). After smoothing, the 99th percentile of the smoothed βs can be calculated to obtain upper and lower thresholds. These thresholds are then used to define hyper- or hypo-methylated DMRs with smoothed peaks above or below the thresholds. For each DMR identified, a sum statistic is calculated by taking the sum of the absolute values of all the smoothed βs within that region. The sum statistic is then used to rank all DMRs with the top-most important DMR having the highest sum statistic value. Statistical significance of the DMRs is assessed by computer permutation (we set 1000 replications) in combination with correction for multiple testing to obtain family-wised error rate (FWER) [22].

### Biological pathway analysis

Advantaged by the multiple-CpG-based analysis that outputs genomic locations of the identified DMRs, biological pathway analysis was conducted by submitting the chromosomal coordinates of the detected DMRs to the Genomic Regions Enrichment of Annotations Tool (GREAT) at http://bejerano.stanford.edu/great/public/html/ to analyse the functional significance of *cis*-regulatory regions identified by localized measurements of DNA binding events across an entire genome [23] using the Genome Reference Consortium Human Build 37 (GRCh37) as the RefSeq database. GREAT incorporates annotations from 20 ontologies and associates genomic regions with genes by defining a ‘regulatory domain’ for each gene such that all non-coding sequences that lie within the regulatory domain are assumed to regulate that gene. The ‘two nearest genes’ was assigned as the association rule from genomic regions to genes, which extends each gene’s regulatory domain from its transcription start site (TSS) to the nearest upstream and downstream TSS, up to 1 MB in each direction. Both the binomial test over genomic regions and the hypergeometric test over genes were performed to provide an accurate picture of annotation enrichments [23].

### Genomic plotting

Visualization and annotation of genomic segments hosting regions under differential methylation were realized by integrative plotting using R package Gviz [24]. Information on genomic annotation was taken from the UCSC hg19 assembly.

## Results

### Discovery EWAS

**Fig. 2:**
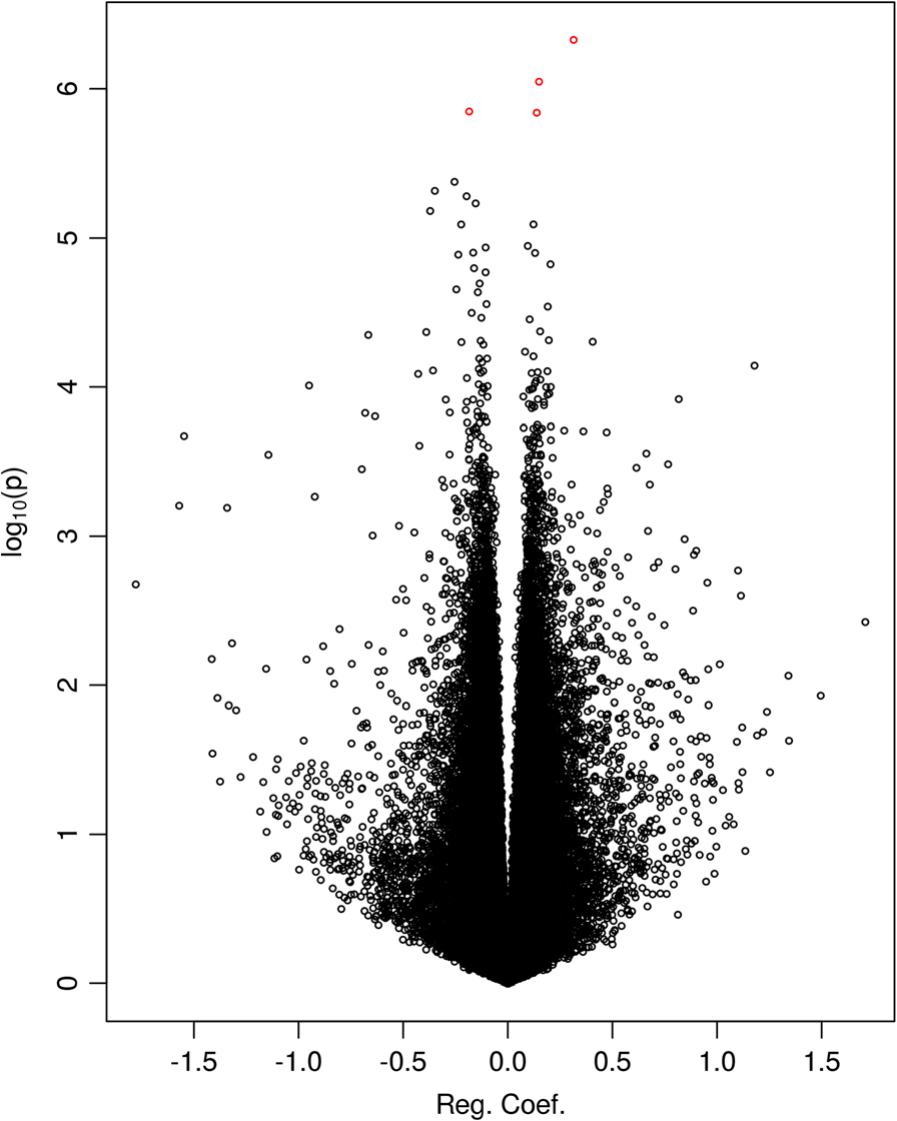
Volcano plot displaying minus log scaled *p* value with base 10 from single CpG site-based EWAS plotted against regression coefficient of PTB. The top red dots are four CpGs with 0.1 < FDR < 0.2

**Fig. 3:**
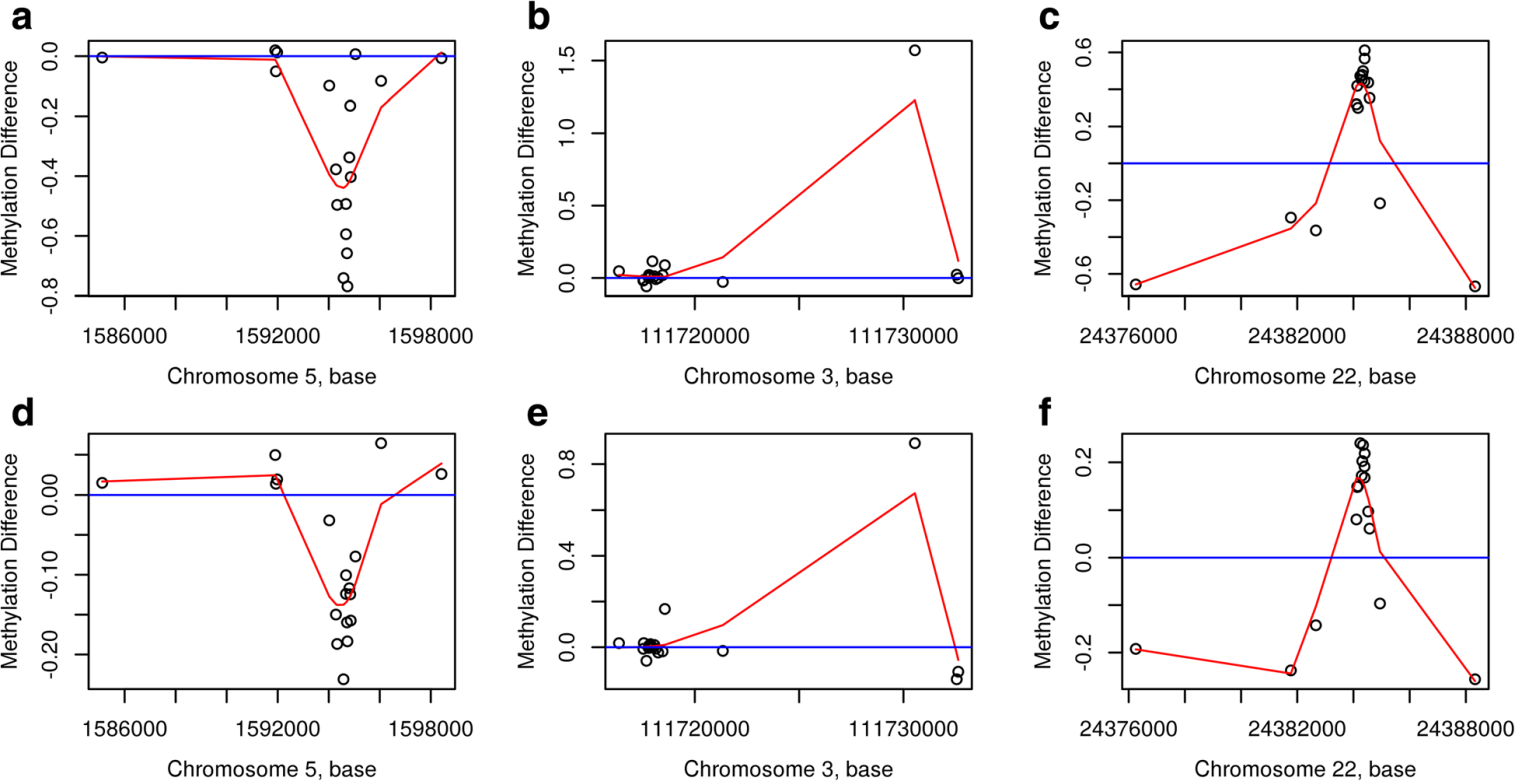
The detected differential DNA methylation patterns and corresponding genomic annotations in the three discovery DMRs with FWER< 0.05, on chromosome 5 (**a**), chromosome 3 (**b**), and chromosome 22 (**c**) and their corresponding replication results (**d**–**f**). The red line is the smooth curve for the difference in methylation (the regression coefficient of PTB) between preterm and term birth subjects

**Fig. 4:**
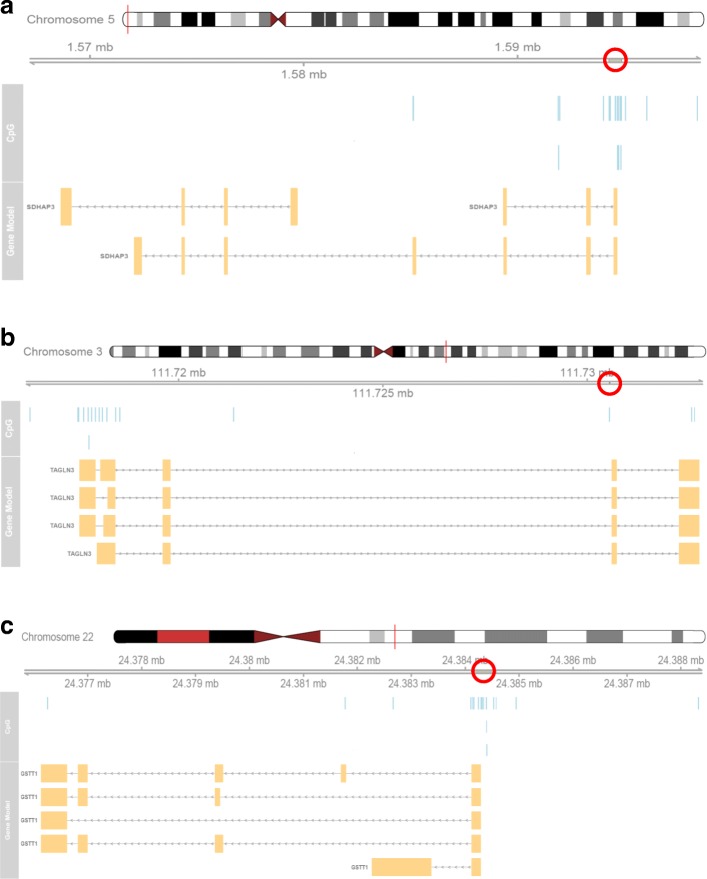
Genomic regions hosting the significant discovery DMRs (indicated by the red circles) on chromosomes 5 (**a**), 3 (**b**) and 22 (**c**) plotted against the CpG sites in Fig. 3 and corresponding gene annotations

**Fig. 5:**
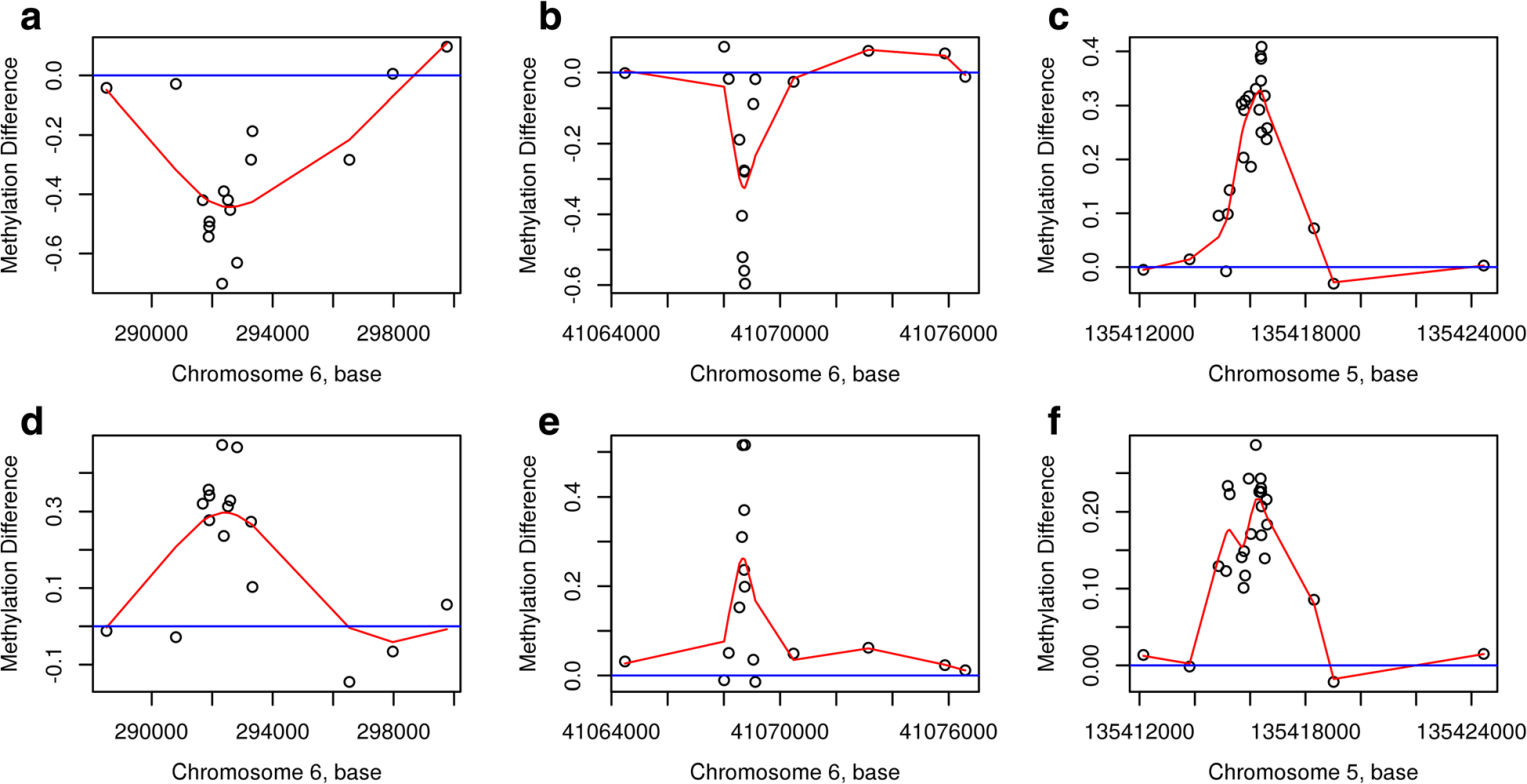
Differential DNA methylation patterns in three discovery DMRs with 0.08 < FWER< 0.1, on chromosome 6 (**a**, **b**) and chromosome 5 (**c**) and their corresponding replication results (**d**–**f**). The red line is the smooth curve for the difference in methylation (the regression coefficient of PTB) between preterm and term birth subjects

We first performed an EWAS on the 144 discovery samples using regression analysis on each of the 473,864 CpGs after filtering, measured using the Illumina Infinium HumanMethylation450 Beadchip assay (see the ‘Methods’ section for details). From the volcano plot (Fig. 2) and Manhattan plot (Additional file 1: Figure S1), it can be seen that no CpG reached genome-wide significance level of FDR < 0.05 for the effect of PTB (Additional file 2: Table S1). We continued our discovery EWAS by performing genomic region-based analysis using the *bumphunter* function in the free R package *minfi* (see the ‘Methods’ section for detail)*.* By focusing on regions with a mean methylation difference of over 10% between PTB and term births, we found a list of 16,508 regions (Additional file 3: Figure S2) and among them 2651 regions with *p* value < 0.05 (Additional file 4: Table S2). Table 2 shows the top six regions with FWER < 0.1, three of them with FWER < 0.05. Among the top three DMRs, the most significant was annotated to the promotor region of *SDHAP3* gene on chromosome 5 at p15.33 exhibiting a clear pattern of hypomethylation (Figs. 3a and 4a); the second most significant DMR was hypermethylated in the gene body (second or third intron) of *TAGLN3* on chromosome 3 at q13.2 (Figs. 3b and 4b); and the third DMR was hypermethylated in the promotor region of the GSTT1 gene on chromosome 22 at q11.23 (Figs. 3c and 4c). Of the three less significant DMRs with FWER < 0.1, two were hypomethylated in the promotor region of the *DUSP22* and *NFYA/LOC221442* genes on chromosome 6 (Fig. 5a, b), and one was hypermethylated in the promotor of *mir886* on chromosome 5 (Fig. 5c). In Additional file 5: Table S3, we show the detailed information on statistical estimate and biological annotations for single CpGs in each of the DMRs in Figs. 3, 4 and 5 (Table 2). CpGs in each DMR show similar direction of effect and tend to have low nominal *p* values that may, however, not reach statistical significance individually.

**Table 2:** Characterizations of the six identified DMRs with FWER < 0.1

DMR	Chr	Start	End	Discovery			Replication				
				Value	*p* value	FWER	Value	*p* value	Linked genes	Gene region	Location to CGI
1	5	1,594,282	1,594,863	− 0.505	4.46E−07	0.01	− 0.157	1.90E−04	SDHAP3	Body, TSS200, TSS1500	Island
2	3	111,730,545	111,730,545	1.571	7.92E−07	0.02	0.891	8.88E−05	TAGLN3	Body	NA
3	22	24,384,159	24,384,573	0.455	2.28E−06	0.04	0.184	5.03E−04	GSTT1	1stExon, 5′UTR, TSS200	Island
4	6	291,687	292,596	− 0.453	4.26E−06	0.08	0.311	2.55E−05	DUSP22	1stExon, 5′UTR, TSS200, TSS1500	Island, N-Shore
5	6	41,068,646	41,068,752	− 0.512	4.46E−06	0.08	0.382	1.70E−05	NFYA, LOC221442	3’UTR	Island
6	5	135,415,258	135,416,613	0.271	4.90E−06	0.10	0.197	8.68E−06	MIR886	Body, TSS200, TSS1500	Island, N-Shore, S-Shore

### Functional analysis of significant DMRs

To study the biological functions of the 2651 regions with *p* < 0.05 (Additiona file 5: Table S2), we used the online annotation tool GREAT (see the ‘Methods’ section) for exploring the *cis* regulatory regions of nearby genes, making use of the Molecular Signatures Database (MSigDB) which is one of the most widely used and comprehensive databases of gene sets for performing gene set enrichment analysis. The analysis identified five MSigDB pathways, all involved in immune responses (Table 3). Both the binomial test over genomic regions and the hypergeometric test over genes showed very high significance for the five pathways as indicated by their FDRs (FDR < 8.13e−28 for binomial test; FDR < 9.37e−03 for hypergeometric test).

**Table 3:** MSigDB Pathways enriched by DMRs with nominal *p* < 0.05 from GREAT analysis

Binomial model						Hypergeometric model					
Term Name	Raw*p* value	*FDR*	Fold enrichment	Exp.	Obs. Gene Hits	Raw*p* value	*FDR*	Fold enrichment	Exp.	Obs.Gene Hits	TotalGenes
Antigen processing and presentation	1.33e−55	1.76e−52	12.28	6.27	77	3.55e−5	9.37e−3	2.10	13.79	29	80
Graft-versus-host disease	5.65e−53	3.73e−50	14.46	4.63	67	2.17e−6	9.56e−4	2.98	6.38	19	37
Type I diabetes mellitus	1.52e−50	6.72e−48	10.20	7.65	78	1.24e-7	1.63e−4	3.11	7.07	22	41
Allograft rejection	7.25e−50	2.40e−47	12.55	5.42	68	7.10e-7	4.69e−4	3.15	6.03	19	35
Viral myocarditis	4.92e-30	8.13e−28	5.13	15.02	77	9.64e-6	3.18e−3	2.30	11.72	27	68

### Replication of top significant DMRs

Using the old twin samples, we performed independent replication analysis of the six DMRs in Table 2. For each DMR, the same set of CpGs as in the discovery stage was selected according to genomic location. Differential methylation between PTB and term birth replication samples was estimated in the same manner as for the discovery analysis (adjusting for age, sex and cell composition). Figure 3d–f displays the replicated differential methylation patterns corresponding to the three discovery-stage DMRs in Fig. 3a–c. As shown in Fig. 3, the three DMRs with FWER < 0.05 were all nicely replicated with nearly the same patterns. Table 2 shows very low nominal *p* values for the replication DMRs: 1.9e−04, 8.88e−05 and 5.03e−04 respectively.

In Fig. 5d–f, we show the replication results for the three less significant discovery DMRs with 0.05 < FWER < 0.1. Interestingly, although a similar methylation pattern was replicated for the DMR on chromosome 5, the other two DMRs on chromosome 6 were replicated with again similar patterns but opposite directions. The corresponding patterns in the replication samples were not random patterns considering their very low nominal *p* values (2.55e−05, 1.7e−05 and 8.68e−06 respectively) (Table 2).

## Discussion

We have performed the first genome-wide association study on the epigenetic effect of preterm birth in adults. Cruickshank et al. [14] investigated PTB-related epigenetic changes at birth and at 18 years of age and reported no genome-wide significant finding in their samples from 18-year-olds. Likewise, our analysis did not identify any CpG sites reaching genome-wide significance in the discovery samples of young adults. The highly valuable findings in this study come from genomic region-based association analysis that jointly tested the association of groups of adjacent CpGs that form DMRs. As shown in Table 2, multiple genomic regions were found as differentially methylated in association with PTB. The results indicate that, as an early life event, PTB could impose differential epigenetic patterns that can be detected in the DNA methylome of adult subjects in their thirties as in the discovery samples and even at old ages as in the replication samples.

Among the genes linked to the most significant DMRs in Table 2, *SDHAP3* has very recently been implicated in smoking, as significantly decreased methylation at the CpG island within the promoter region of *SDHAP3* on chromosome 5 was reported in smoking-exposed foetuses [25]. The PTB associated methylation pattern as shown in Figs. 3a and 4a points to the same direction and genomic location although maternal smoking information is not available in our study. A differential DNA methylation pattern was also found in *SDHAP3* when comparing autistic brains and control [26]. The second most significant DMR is in the gene body of *TAGLN3* (Fig. 4b). This gene (also known as NP22, encoding a novel cytoskeleton-associated protein) is differentially expressed in human alcoholic brain [27] and in the anterior cingulate cortex of schizophrenia [28].

Perhaps, the most interesting DMR found in this study is the third DMR in Table 2. This DMR sits in the promotor region of *GSTT1* (glutathione *S*-transferases gene theta 1) on chromosome 22 (Fig. 4c). Polymorphisms in the *GST* genes are partially responsible for the variability in *GST* enzymatic activity across individuals. Maternal genetic variations (the null genotype or homozygous deletion) in *GSTT1* have been intensively associated with an increased risk of preterm delivery and low birthweight, alone [29, 30] or in combination with smoking [31–33]. The interaction between *GST*s and smoking shows the involvement of epigenetic mechanism that links maternal behaviour and genetic susceptibility in contributing to adverse pregnancy outcomes. The association between *GST* genetic variation and PTB has been observed not only in the mother but also in the child [34]. In fact, Bustamante et al. [35] found that the child genotype is responsible for the effect after adjusting for maternal genotype. As our observation is based on PTB adults, our result is in line with their conclusion but from an epigenetic perspective. Most importantly, the latter suggests that environmental factors could also be involved in the association between *GSTT1* and PTB through the epigenetic mechanism. Taken together, both genetic and epigenetic variations in the child can be associated with PTB. The coherence between genetics and epigenetics here is sensible because DNA methylation at the promotor region turns the gene off, which is equivalent to a deletion or the null genotype of the gene.

As an extra effort, we explored the transcriptional profiles of the genes linked to significant DMRs. Gene expression data on two genes, *TAGLN3* and *GSTT1*, were available from the Agilent Human Gene Expression Microarray (v3) applied to the same discovery samples. After adjusting for covariates, no expression difference was found for *TAGLN3* between term birth and PTB (*p* = 0.639) while a borderline significance (*p* = 0.059) for the down-expression of *GSTT1* in PTB (Additional file 6: Figure S3). Although the expression of *GSTT1* can also be regulated by other mechanisms or influenced by deletion of the gene in PTB subjects, the reduced expression level in PTB group provides alternative evidence in support of DNA methylation analysis.

Among the three less significant DMRs in Table 2, the last one on chromosome 5 is replicated by a similar pattern in the old twins (Fig. 5f). The CpGs in this region are hypermethylated in the promotor region and gene body of *mir886*, a noncoding RNA repressed in cancer [36, 37]. The two DMRs on chromosome 6 display significant patterns (*p* values 2.55e−05 and 1.70e−05) in the replication samples but with opposite directions as compared to the discovery DMRs (hypomethylation in the discovery samples, and hypermethylation in the replication samples) which could possibly suggest age-dependent effects. The DMR located on chromosome 6 from bp 291,687 to 292,596 covers the promotor region of the *DUSP22* gene. Epigenetic alteration of this gene has been shown to mediate Alzheimer’s disease [38] and dementia [39]. Interestingly, hypomethylation of the *DUSP22* promotor has been reported to correlate with duration of service in firefighters [40]. The observed epigenetic modification could result from exposure to complex mixtures of toxic substances from burning and overheated materials. Although the smoking status of mothers of our twins is not available, the finding among the firefighters could resemble smoking-exposed foetuses. The DMR located from bp 41,068,646 to 41,068,752 on chromosome 6 is at the 3′UTR of the *NFYA* (nuclear transcription factor Y) gene. As a transcription factor, *NFYA* binds to the CAAT box in promotors of many genes in eukaryotes and functions as a regulator of their overexpression in several types of cancer [41]. Note that the same gene has been found to be persistently hypermethylated by PTB in an epigenome-wide association study on both newborn and 18-year-old samples [14]. In brief, the genes covered by these less significant DMRs are implicated in neurodegenerative disorders and risk of cancer as well.

PTB newborns have immature immune systems with reduced innate and adaptive immune function [42]. It is interesting to see that four of the five pathways in Table 3 overlap with pathways deduced from genes linked to CpGs showing significant correlation in maternal and PTB fetal methylation [43], and that all the five pathways appeared in the enriched functional pathways from genes with copy number variations in common miscarriage [44]. Results from our biological pathway analysis reconfirm the importance of the immune system in PTB but in adult samples. Meanwhile the overlap in biological pathways could also suggest the broad involvement of immunity in labour complications in general. Most importantly, the immune implication of PTB could persist into adult life and even old ages. The high involvement of the immune system in PTB as revealed by region-based analysis can also be seen from the Manhattan plot for DMRs (Additional file 3: Figure S2) when compared with the Manhattan plot for single-CpG sites (Additional file 1: Figure S1). The former displays a clearer enrichment pattern of DMRs in the major histocompatibility complex (MHC) region on chromosome 6.

The fact that the significant DMRs were identified and replicated in independent and much older samples has a twofold significance. First, it reveals functional genes differentially regulated in association with PTB through epigenetic mechanism; the latter could serve to link PTB with maternal environmental exposure or lifestyle factors to provide clue for prevention of PTB. Second, and also most importantly, the altered DNA methylation patterns observed in our discovery young adults persist in old subjects of up to 80 years of age, suggesting that some of the PTB-associated epigenetic modifications can be long-lasting or perhaps persistent throughout the entire life. In summary, our genomic region-based analysis of the DNA methylome identified epigenetic fingerprints of premature birth in young adult subjects, consistently replicable in old adults. Functional annotation of the significant methylation patterns associated with PTB revealed genes involved in adverse pregnancy outcomes, in neurodevelopmental disorders and in cancer susceptibility, providing epigenetic evidence of long-term effects of early life events in support of the developmental origin of disease and health.

Finally, it should be kept in mind that our significant findings are based on twins. Even though findings from this study are highly relevant to PTB in general (e.g. the *GSTT1* gene), generalization of our results should be done with caution because the aetiology of PTB in twins could involve risk factors specific for twin pregnancies [45], such as uterine overdistention [46]. Further replication studies using twin and non-twin samples are warranted for validation, justification and generalization of our findings.

## Conclusions

This study provides novel evidence for PTB-associated epigenetic regulation in important genes/pathways and meanwhile reveals that premature delivery, as an early life event, could be related to differential methylation regulation patterns observable in adults and even at high ages which could potentially mediate susceptibility to age-related diseases and adult health.

## Additional files

Additional file 1: **Figure S1.** Manhattan plot for EWAS results based on single CpG sites on autosomal chromosomes. The *y*-axis shows the negative log-base-10 of the *P* value for each CpG. (PNG 1276 kb)

Additional file 2: **Table S1.** PTB EWAS results with *p* value below 1%. (PDF 570 kb)

Additional file 3: **Figure S2.** Manhattan plot for genomic regions from region based analysis on autosomal chromosomes. The *y*-axis shows the negative log-base-10 of the *p* value for each genomic region. (PNG 932 kb)

Additional file 4: **Table S2.** PTB DMR with p value lower than 5%. (PDF 991 kb)

Additional file 5: **Table S3.** CpGs under top DMRs. (PDF 71 kb)

Additional file 6: **Figure S3.** Boxplot displaying the expression patterns of *GSTT1* gene in preterm (PTB) and term (TB) groups. The gene shows reduced expression level in the PTB group. (PNG 87 kb)

## Abbreviations

CpG: Cytosine-phosphate-guanine
DMBR: Danish medical birth registry
DMP: Differentially methylated probe
DMR: Differentially methylated region
EDTA: Ethylene di-amine tetra acetic acid
EWAS: Epigenome-wide association study
FDR: False discovery rate
FWER: Family-wise error rate
GREAT: Genomic Regions Enrichment of Annotations Tool
MHC: Major histocompatibility complex
MSigDB: Molecular Signatures Database
SWAN: Subset-quantile Within Array Normalization
TSS: Transcription start site(s)
