# Supplementary figures and images for: Epigenetic signature of preterm birth in adult twins
Source: Clin Epigenetics. 2018 Jun 27;10:87. doi: 10.1186/s13148-018-0518-8 (PMC6020425; doi:10.1186/s13148-018-0518-8)

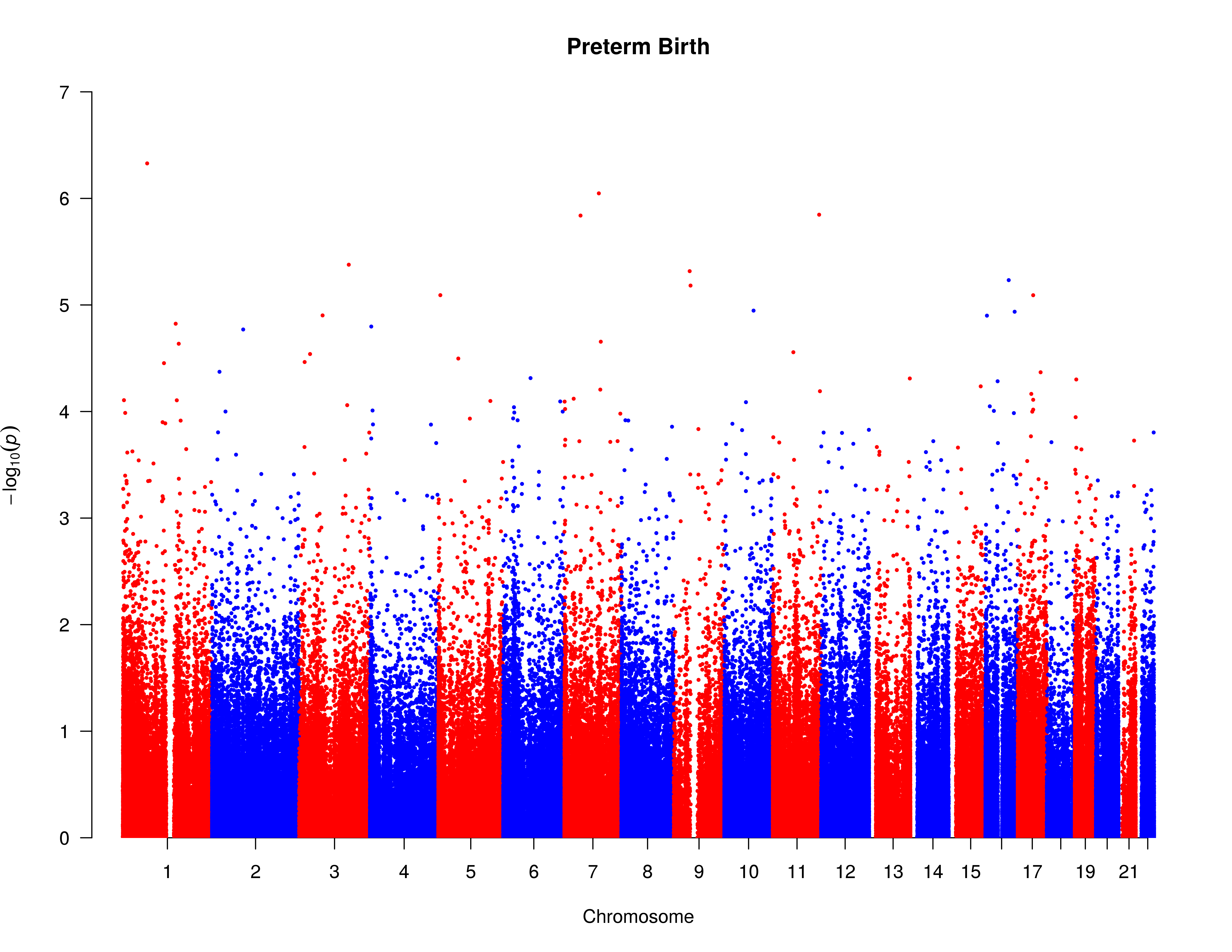

Supplement: Supplementary file 1 — Figure S1. Manhattan plot for EWAS results based on single CpG sites on autosomal chromosomes. The y-axis shows the negative log-base-10 of the P value for each CpG. (PNG 1276 kb) [file 13148_2018_518_MOESM1_ESM.png]

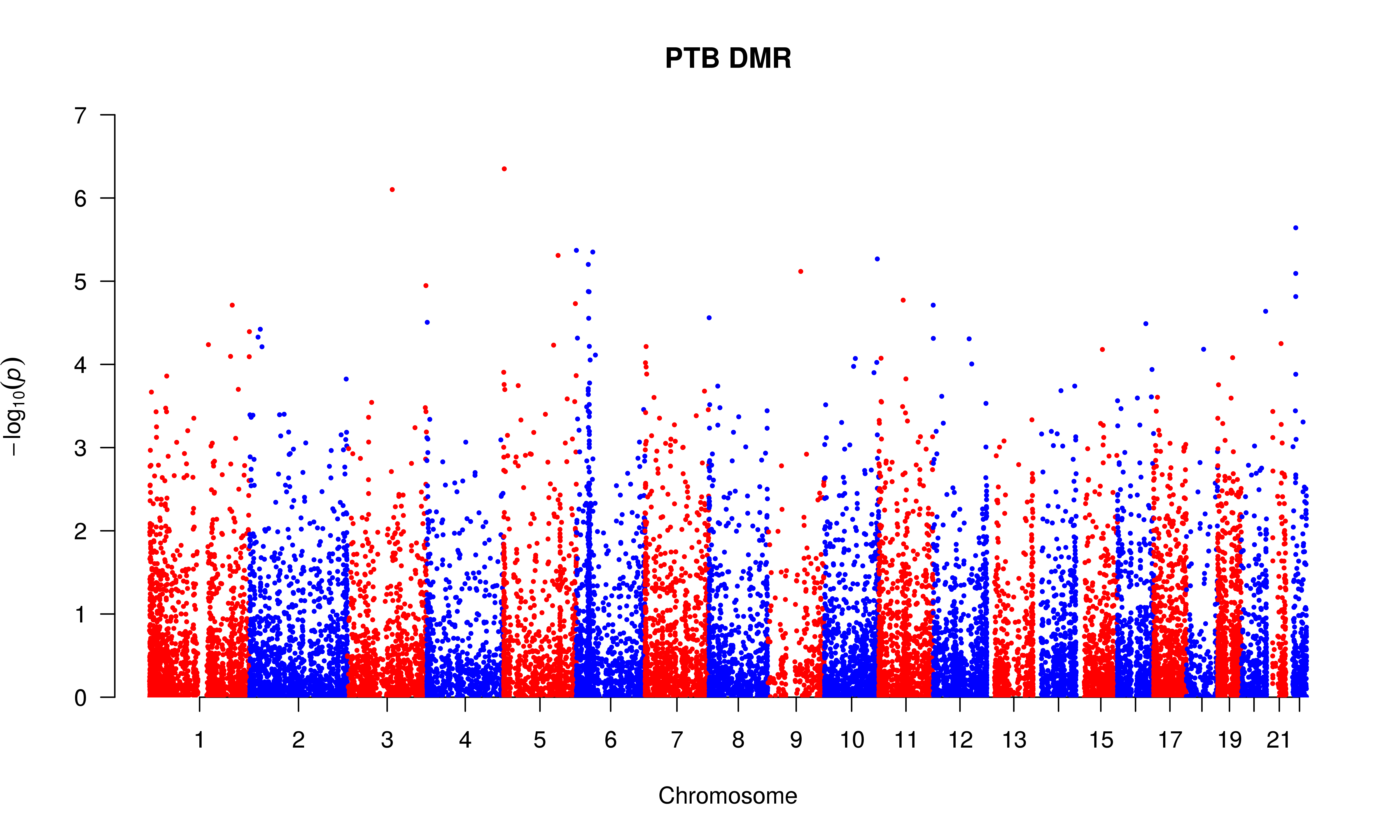

Supplement: Supplementary file 3 — Figure S2. Manhattan plot for genomic regions from region based analysis on autosomal chromosomes. The y-axis shows the negative log-base-10 of the p value for each genomic region. (PNG 932 kb) [file 13148_2018_518_MOESM3_ESM.png]

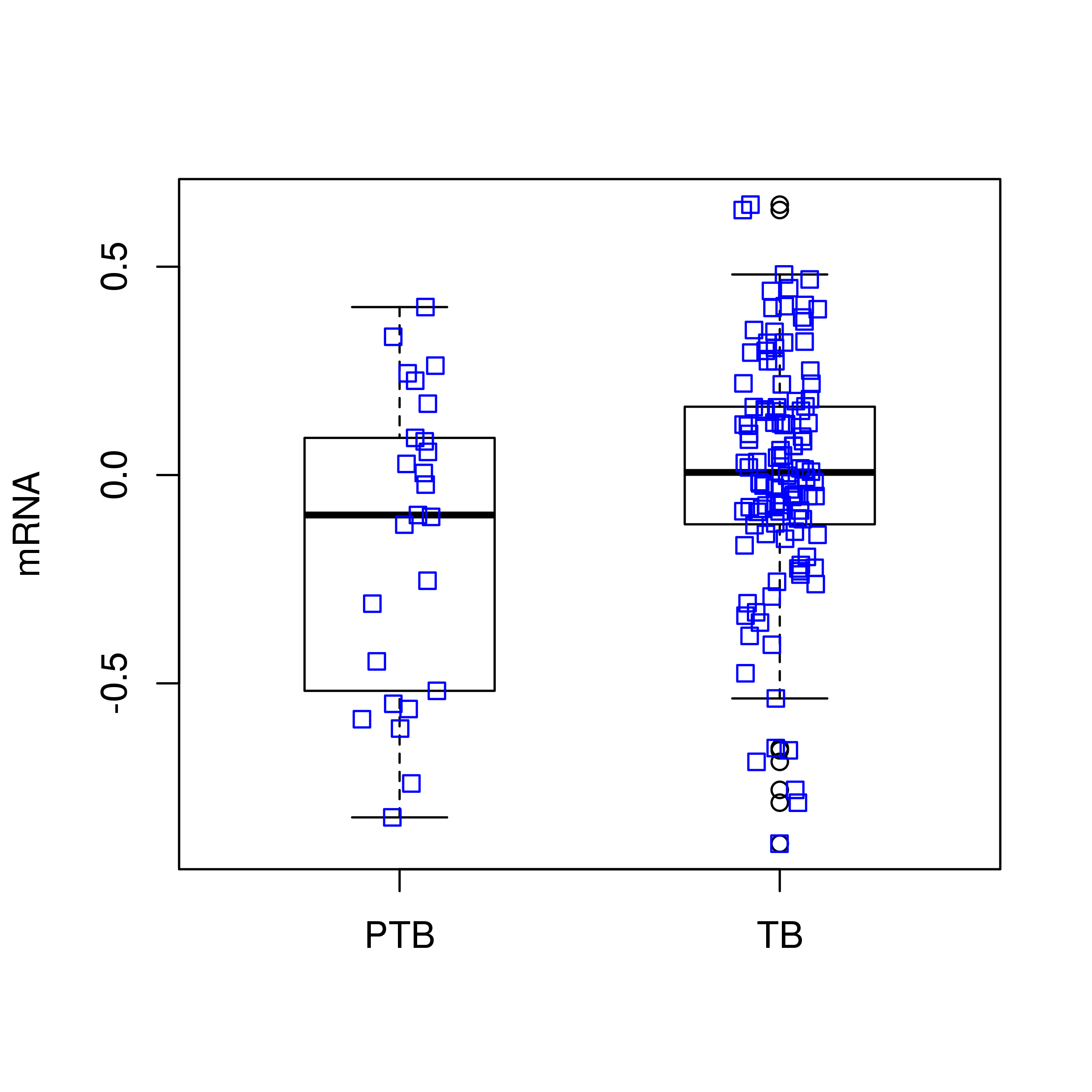

Supplement: Supplementary file 6 — Figure S3. Boxplot displaying the expression patterns of GSTT1 gene in preterm (PTB) and term (TB) groups. The gene shows reduced expression level in the PTB group. (PNG 87 kb) [file 13148_2018_518_MOESM6_ESM.png]
