# Supplementary material for: Epigenetic signature of preterm birth in adult twins
Source: Clin Epigenetics. 2018 Jun 27;10:87. doi: 10.1186/s13148-018-0518-8 (PMC6020425; doi:10.1186/s13148-018-0518-8)
Supplement: Supplementary file 2 — Table S1. PTB EWAS results with p value below 1%. (PDF 570 kb) [file 13148_2018_518_MOESM2_ESM.pdf]

sep="\t"

ID	RegCoef	SE	t	pvalue	qvalue
cg2680477	0.314607	0.059412	5.295379	4.69E-07	0.154885
cg0060464	0.149332	0.028989	5.151335	8.97E-07	0.154885
cg1628307	-0.18445	0.036546	-5.04718	1.42E-06	0.154885
cg1768377	0.13835	0.027435	5.042895	1.45E-06	0.154885
cg1184098	-0.25471	0.05309	-4.7977	4.19E-06	0.31243
cg0443187	-0.34858	0.073156	-4.76485	4.82E-06	0.31243
cg1530305	-0.19699	0.041513	-4.74516	5.24E-06	0.31243
cg0272402	-0.15364	0.032558	-4.71903	5.85E-06	0.31243
cg0527150	-0.37083	0.079048	-4.69115	6.58E-06	0.31243
cg1800207	0.122627	0.026419	4.641597	8.09E-06	0.314947
cg0636830	-0.22271	0.047983	-4.6413	8.10E-06	0.314947
cg1886028	0.095555	0.02095	4.56115	1.13E-05	0.345511
cg1051146	-0.10605	0.023281	-4.55528	1.16E-05	0.345511
cg2547311	-0.16535	0.036453	-4.53596	1.25E-05	0.345511
cg2351932	0.130399	0.028756	4.534707	1.26E-05	0.345511
cg0852242	-0.23658	0.052243	-4.52839	1.29E-05	0.345511
cg0396549	0.204022	0.045418	4.492135	1.50E-05	0.377237
cg2334499	-0.16133	0.036033	-4.47722	1.59E-05	0.378632
cg1265379	-0.10621	0.023806	-4.46155	1.70E-05	0.382318
cg0596163	-0.1346	0.030461	-4.41882	2.02E-05	0.431878
cg0617366	-0.24584	0.05592	-4.39634	2.21E-05	0.44926
cg2513388	-0.14329	0.032674	-4.38537	2.31E-05	0.44926
cg1096421	-0.10183	0.023467	-4.33954	2.78E-05	0.514465
cg1754672	0.190928	0.044097	4.329768	2.89E-05	0.514465
cg2652236	-0.17279	0.040134	-4.30532	3.18E-05	0.544314
cg0382738	-0.1262	0.029442	-4.28626	3.43E-05	0.556946
cg1603630	0.104325	0.024374	4.280109	3.52E-05	0.556946
cg0812524	0.154599	0.036524	4.232775	4.24E-05	0.629224
cg0728358	-0.38946	0.092069	-4.2301	4.28E-05	0.629224
cg1234267	-0.66659	0.158003	-4.21885	4.48E-05	0.629224
cg1888291	0.196048	0.046702	4.19789	4.86E-05	0.629224
cg2530772	-0.12974	0.030922	-4.19585	4.90E-05	0.629224
cg2454400	0.4059	0.096829	4.191916	4.97E-05	0.629224
cg0132883	-0.22154	0.052869	-4.19031	5.00E-05	0.629224
cg2719176	-0.11667	0.027908	-4.18037	5.20E-05	0.635387
cg2593617	0.082505	0.019871	4.152096	5.80E-05	0.689407
cg1739805	0.122601	0.029657	4.133926	6.23E-05	0.707511
cg0171289	-0.09884	0.023963	-4.12487	6.45E-05	0.707511
cg2554688	-0.13652	0.033097	-4.12468	6.45E-05	0.707511
cg2567359	-0.12329	0.029998	-4.10981	6.83E-05	0.710672
cg0054029	1.179028	0.287822	4.096376	7.20E-05	0.710672
cg0415758	-0.13856	0.033938	-4.08265	7.58E-05	0.710672
cg1117833	-0.35734	0.087651	-4.07679	7.76E-05	0.710672
cg0107987	-0.11751	0.028841	-4.07437	7.83E-05	0.710672
cg0094478	0.189323	0.046474	4.073775	7.85E-05	0.710672
cg1247077	0.142912	0.035115	4.069869	7.96E-05	0.710672
cg2407666	-0.12933	0.031799	-4.06715	8.05E-05	0.710672
cg1103631	0.114078	0.028054	4.066318	8.07E-05	0.710672

cg2704046	-0.42828	0.105409	-4.06306	8.17E-05	0.710672
cg0814005	-0.19535	0.04828	-4.04612	8.72E-05	0.710672
cg1019602	0.155689	0.03854	4.039656	8.94E-05	0.710672
cg1501828	0.135004	0.033463	4.034373	9.12E-05	0.710672
cg2640032	0.129266	0.032122	4.02425	9.48E-05	0.710672
cg0926305	-0.1393	0.034646	-4.02073	9.60E-05	0.710672
cg2036041	-0.94965	0.236482	-4.01576	9.78E-05	0.710672
cg1014933	-0.09652	0.024047	-4.01355	9.87E-05	0.710672
cg1516239	0.205245	0.051185	4.009892	0.0001	0.710672
cg2380891	-0.11861	0.02958	-4.0097	0.0001	0.710672
cg2431252	0.183253	0.045708	4.009206	0.0001	0.710672
cg2433196	0.120463	0.030085	4.004102	0.000102	0.710672
cg2168137	-0.11336	0.028327	-4.00194	0.000103	0.710672
cg0698020	0.11386	0.028458	4.000987	0.000103	0.710672
cg0462248	0.102955	0.025753	3.997809	0.000105	0.710672
cg0024203	-0.12354	0.030983	-3.98729	0.000109	0.722688
cg0420797	0.198365	0.049813	3.982189	0.000111	0.722688
cg0817263	0.18932	0.047598	3.977521	0.000113	0.722688
cg1776759	0.074232	0.018696	3.970373	0.000116	0.722688
cg1094970	-0.09736	0.024527	-3.96933	0.000117	0.722688
cg1328941	0.816989	0.206321	3.959789	0.000121	0.722688
cg2302634	0.143543	0.036251	3.959692	0.000121	0.722688
cg1093054	-0.16573	0.04187	-3.95822	0.000122	0.722688
cg0409479	-0.29632	0.074869	-3.95785	0.000122	0.722688
cg0099552	-0.19609	0.04966	-3.94864	0.000126	0.726969
cg0425268	0.173015	0.043826	3.947795	0.000126	0.726969
cg2610009	-0.13195	0.033472	-3.94197	0.000129	0.726969
cg0138179	0.100328	0.025467	3.93957	0.00013	0.726969
cg0665655	0.11684	0.029687	3.935762	0.000132	0.726969
cg0019050	0.173224	0.044023	3.934868	0.000133	0.726969
cg0939361	0.126695	0.032252	3.928315	0.000136	0.735528
cg1360161	0.11427	0.029132	3.922501	0.000139	0.742244
cg1088533	-0.20071	0.051275	-3.91441	0.000143	0.755503
cg0623066	-0.14509	0.03712	-3.90871	0.000146	0.760983
cg1883393	-0.27713	0.070977	-3.90443	0.000149	0.760983
cg2705748	-0.68153	0.174631	-3.90267	0.00015	0.760983
cg1412554	-0.12438	0.031977	-3.88968	0.000157	0.762806
cg2061808	-0.63423	0.163083	-3.88899	0.000157	0.762806
cg1728615	-0.12359	0.031783	-3.88849	0.000158	0.762806
cg0672609	-0.1438	0.036983	-3.88835	0.000158	0.762806
cg1492069	-0.11034	0.02839	-3.88643	0.000159	0.762806
cg2609455	-0.10125	0.026185	-3.86652	0.000171	0.804999
cg1251548	-0.19391	0.050197	-3.86286	0.000173	0.804999
cg2748232	-0.1354	0.035068	-3.86102	0.000174	0.804999
cg0063128	0.141354	0.036681	3.853575	0.000179	0.804999
cg1790762	0.205562	0.053438	3.846717	0.000184	0.804999
cg0236161	-0.15235	0.039645	-3.84287	0.000186	0.804999
cg0259962	0.138257	0.035991	3.841452	0.000187	0.804999
cg0237010	0.075628	0.019688	3.841331	0.000187	0.804999
cg0980690	0.163277	0.042541	3.838163	0.00019	0.804999

cg2680356	0.12202	0.031793	3.837954	0.00019	0.804999
cg0765039	-0.14966	0.038999	-3.83751	0.00019	0.804999
cg2338030	-0.16525	0.043105	-3.83372	0.000193	0.804999
cg0766711	0.155087	0.040472	3.831942	0.000194	0.804999
cg0475235	0.269584	0.070384	3.830186	0.000195	0.804999
cg0847447	0.361293	0.094408	3.826934	0.000198	0.804999
cg2505017	-0.18505	0.048356	-3.82689	0.000198	0.804999
cg1529666	0.472364	0.123575	3.822486	0.000201	0.808389
cg0070096	-0.14544	0.038068	-3.82058	0.000202	0.808389
cg2363656	-0.15412	0.040418	-3.81305	0.000208	0.82016
cg0513473	-1.54716	0.406417	-3.80684	0.000213	0.82016
cg1258500	0.125656	0.033009	3.806752	0.000213	0.82016
cg2631541	0.13573	0.035687	3.803368	0.000215	0.82016
cg1223761	-0.12873	0.033847	-3.80334	0.000215	0.82016
cg0539007	0.14505	0.038171	3.800052	0.000218	0.82016
cg2440805	-0.17731	0.046669	-3.79926	0.000219	0.82016
cg1808093	0.09013	0.023766	3.792323	0.000224	0.828082
cg0611675	0.105936	0.027944	3.79096	0.000225	0.828082
cg1001123	0.20581	0.054313	3.78934	0.000227	0.828082
cg2432747	0.124732	0.032936	3.787135	0.000229	0.828082
cg1137704	0.101526	0.026877	3.777467	0.000237	0.849297
cg2234178	0.152284	0.040334	3.775537	0.000238	0.849297
cg1360845	-0.11737	0.031108	-3.77309	0.00024	0.849777
cg0000357	-0.18319	0.048586	-3.77051	0.000243	0.850742
cg0593860	-0.42264	0.112243	-3.7654	0.000247	0.856502
cg0607044	0.106862	0.028389	3.764154	0.000248	0.856502
cg0980850	0.121517	0.032308	3.76121	0.000251	0.858713
cg0816297	-0.09448	0.025142	-3.7579	0.000254	0.858713
cg1144427	0.119411	0.031785	3.75683	0.000255	0.858713
cg1112245	-0.18643	0.049697	-3.75142	0.00026	0.868813
cg0217947	0.662122	0.177401	3.732353	0.000279	0.905856
cg2421798	0.156846	0.042061	3.729047	0.000282	0.905856
cg1371742	-0.27821	0.074625	-3.72807	0.000283	0.905856
cg1539214	-1.14401	0.306959	-3.72692	0.000284	0.905856
cg0250865	0.131782	0.035368	3.726023	0.000285	0.905856
cg2285972	0.141298	0.037928	3.725481	0.000286	0.905856
cg2595596	0.11303	0.030355	3.72363	0.000287	0.905856
cg0688517	0.158812	0.042666	3.722193	0.000289	0.905856
cg0105981	-0.11854	0.031869	-3.71965	0.000292	0.905856
cg0978595	0.214325	0.057709	3.713899	0.000298	0.905856
cg0502947	0.09701	0.026122	3.713739	0.000298	0.905856
cg0361830	-0.11206	0.030178	-3.71329	0.000298	0.905856
cg1553011	-0.12095	0.032577	-3.71292	0.000299	0.905856
cg1300909	-0.18575	0.050095	-3.70795	0.000304	0.915631
cg2267057	-0.18446	0.049788	-3.70495	0.000307	0.919043
cg0833034	0.098754	0.026688	3.700325	0.000313	0.927899
cg1507797	-0.14568	0.039437	-3.69412	0.00032	0.942167
cg0135675	0.766138	0.207843	3.686141	0.000329	0.960195
cg1980986	0.151335	0.041081	3.683782	0.000332	0.960195
cg2695798	-0.10961	0.029777	-3.68119	0.000335	0.960195

cg1019592	-0.15488	0.042088	-3.68002	0.000336	0.960195
cg2507370	-0.13312	0.036181	-3.6793	0.000337	0.960195
cg0882199	0.61487	0.167503	3.6708	0.000347	0.967968
cg2686202	0.10008	0.027272	3.669669	0.000349	0.967968
cg2605989	-0.11833	0.032252	-3.66874	0.00035	0.967968
cg0153632	-0.13634	0.037183	-3.66679	0.000352	0.967968
cg2302085	0.124933	0.034078	3.666073	0.000353	0.967968
cg2492343	-0.69768	0.190388	-3.66451	0.000355	0.967968
cg2119576	0.136239	0.037181	3.664202	0.000355	0.967968
cg1043628	-0.08926	0.024384	-3.66076	0.00036	0.97368
cg2499080	-0.16964	0.04639	-3.65674	0.000365	0.974034
cg0717053	-0.13446	0.036777	-3.65608	0.000366	0.974034
cg1299183	-0.11243	0.030765	-3.65451	0.000368	0.974034
cg0140857	-0.12462	0.034184	-3.64553	0.00038	0.974034
cg2180142	0.141545	0.038842	3.644077	0.000382	0.974034
cg1553055	-0.14359	0.039413	-3.64321	0.000383	0.974034
cg2069114	-0.06014	0.016518	-3.64096	0.000386	0.974034
cg2343020	0.191125	0.052516	3.639324	0.000388	0.974034
cg1445342	-0.17179	0.047211	-3.63869	0.000389	0.974034
cg2119476	0.08298	0.022806	3.638526	0.000389	0.974034
cg0050332	-0.09102	0.025017	-3.63823	0.00039	0.974034
cg0988290	-0.0911	0.025046	-3.63727	0.000391	0.974034
cg1131598	-0.13327	0.036643	-3.63703	0.000391	0.974034
cg1097239	-0.12648	0.034786	-3.63581	0.000393	0.974034
cg1416203	0.117559	0.032341	3.635028	0.000394	0.974034
cg0369961	-0.10644	0.029315	-3.63104	0.0004	0.977999
cg0493343	0.078546	0.021634	3.63062	0.0004	0.977999
cg1538125	-0.09419	0.025974	-3.62652	0.000406	0.981437
cg2205444	0.110299	0.030415	3.626402	0.000406	0.981437
cg2668717	-0.19933	0.055038	-3.62172	0.000413	0.981753
cg1837429	0.139846	0.038617	3.621387	0.000414	0.981753
cg0372332	-0.11915	0.032925	-3.61879	0.000417	0.981753
cg0029728	0.094166	0.026022	3.618634	0.000418	0.981753
cg0197573	-0.31332	0.086597	-3.61815	0.000418	0.981753
cg1505312	0.146078	0.040399	3.61583	0.000422	0.981753
cg1653817	-0.09623	0.026631	-3.61342	0.000425	0.981753
cg0178946	0.104032	0.028794	3.612932	0.000426	0.981753
cg1059503	-0.11226	0.031079	-3.61201	0.000427	0.981753
cg1676832	-0.16036	0.044433	-3.6091	0.000432	0.981753
cg1685044	0.122135	0.033846	3.608499	0.000433	0.981753
cg2536730	0.152826	0.042434	3.601514	0.000443	0.981753
cg0201619	-0.25927	0.071989	-3.6015	0.000443	0.981753
cg1778927	0.144625	0.040162	3.60105	0.000444	0.981753
cg2458072	-0.11737	0.032608	-3.59931	0.000447	0.981753
cg0617694	0.089247	0.024798	3.598874	0.000448	0.981753
cg0576756	-0.12955	0.036011	-3.59753	0.00045	0.981753
cg1327560	0.304908	0.084757	3.597413	0.00045	0.981753
cg0925280	0.678747	0.188683	3.597299	0.00045	0.981753
cg1544776	0.113843	0.031697	3.591582	0.000459	0.991388
cg2136108	-0.11318	0.031552	-3.58705	0.000467	0.991388

cg0175911	-0.11299	0.031503	-3.58669	0.000467	0.991388
cg1163769	0.135226	0.037709	3.586018	0.000468	0.991388
cg1305315	-0.30443	0.0849	-3.58576	0.000469	0.991388
cg0205525	-0.12285	0.034288	-3.5829	0.000473	0.991388
cg1306015	-0.07922	0.022111	-3.58264	0.000474	0.991388
cg2294740	0.475667	0.132864	3.580109	0.000478	0.991388
cg1636175	0.10357	0.028948	3.577829	0.000482	0.991388
cg2217990	0.114286	0.031957	3.576204	0.000485	0.991388
cg1637064	0.117192	0.032812	3.571576	0.000492	0.991388
cg0551185	0.14927	0.041794	3.571543	0.000492	0.991388
cg1729800	0.119794	0.033552	3.570426	0.000494	0.991388
cg0739729	0.103754	0.029085	3.56728	0.0005	0.991388
cg1942426	-0.11218	0.031457	-3.566	0.000502	0.991388
cg1677949	0.212121	0.059503	3.56488	0.000504	0.991388
cg2722961	-0.20865	0.058587	-3.56139	0.00051	0.991388
cg1411169	-0.08193	0.023016	-3.55955	0.000513	0.991388
cg0262285	-0.1177	0.03308	-3.55809	0.000516	0.991388
cg1370640	-0.15753	0.044305	-3.55561	0.000521	0.991388
cg0004755	0.47844	0.134561	3.555557	0.000521	0.991388
cg0314538	-0.13897	0.039087	-3.55552	0.000521	0.991388
cg1878494	0.167188	0.047028	3.555069	0.000522	0.991388
cg0005893	-0.14677	0.041342	-3.55027	0.00053	0.991388
cg1220800	0.119148	0.033568	3.549465	0.000532	0.991388
cg2262247	0.163681	0.046116	3.549354	0.000532	0.991388
cg1420887	0.081704	0.023036	3.546864	0.000537	0.991388
cg0712290	-0.10057	0.028369	-3.54524	0.00054	0.991388
cg1132465	0.137874	0.038895	3.544783	0.00054	0.991388
cg2518942	-0.12297	0.03469	-3.54468	0.000541	0.991388
cg0735734	0.129622	0.036573	3.544166	0.000542	0.991388
cg0021821	-0.19117	0.05394	-3.54411	0.000542	0.991388
cg1774663	-0.9224	0.260313	-3.54344	0.000543	0.991388
cg0585506	0.209156	0.059053	3.541812	0.000546	0.991388
cg0922646	0.16321	0.046098	3.540468	0.000549	0.991388
cg0996280	0.115192	0.032551	3.538772	0.000552	0.991388
cg0133234	-0.09922	0.028038	-3.5387	0.000552	0.991388
cg1162782	-0.09656	0.027309	-3.53594	0.000557	0.991388
cg2617666	0.119013	0.033659	3.5358	0.000558	0.991388
cg0846603	-0.25318	0.071613	-3.53543	0.000558	0.991388
cg0908632	0.255877	0.072393	3.534552	0.00056	0.991388
cg2102752	0.149154	0.042209	3.533672	0.000562	0.991388
cg2295993	-0.06873	0.01945	-3.53349	0.000562	0.991388
cg0428716	-0.13587	0.038456	-3.53308	0.000563	0.991388
cg1625753	0.145493	0.041218	3.529822	0.000569	0.991388
cg0361085	0.170061	0.048183	3.529512	0.00057	0.991388
cg0790428	-0.1133	0.032106	-3.52879	0.000571	0.991388
cg2618623	-0.08747	0.024799	-3.52697	0.000575	0.991388
cg1752002	-0.15194	0.04309	-3.5262	0.000576	0.991388
cg0544529	-0.19066	0.054071	-3.52604	0.000577	0.991388
cg0639890	0.080573	0.022859	3.524775	0.000579	0.991388
cg1809022	0.152379	0.043235	3.524442	0.00058	0.991388

cg2213394	0.098273	0.027883	3.524432	0.00058	0.991388
cg0284770	-0.24185	0.068637	-3.52353	0.000582	0.991388
cg0088433	-0.12377	0.03513	-3.52312	0.000583	0.991388
cg1897789	-0.18142	0.051511	-3.52202	0.000585	0.991388
cg0344456	-0.1228	0.034876	-3.5211	0.000587	0.991388
cg1197539	0.458373	0.130226	3.519822	0.000589	0.991836
cg0741889	0.137142	0.038989	3.517439	0.000594	0.996102
cg0460208	0.129125	0.036746	3.513934	0.000601	0.99943
cg1971023	-0.18323	0.052159	-3.51286	0.000604	0.99943
cg2527206	0.098873	0.028149	3.512487	0.000604	0.99943
cg0345357	0.107324	0.03056	3.511956	0.000605	0.99943
cg1692164	0.167873	0.047854	3.508004	0.000614	0.999985
cg2729229	0.125913	0.035897	3.507654	0.000614	0.999985
cg0652674	-0.07783	0.022192	-3.50698	0.000616	0.999985
cg2292979	-0.15145	0.043187	-3.50694	0.000616	0.999985
cg0742128	-0.13691	0.03908	-3.50334	0.000624	0.999985
cg0217488	-0.05738	0.01638	-3.50325	0.000624	0.999985
cg0070466	-1.57046	0.448296	-3.50319	0.000624	0.999985
cg1578054	0.182936	0.05228	3.49917	0.000633	0.999985
cg1426615	-0.07248	0.020732	-3.4958	0.00064	0.999985
cg1523440	-0.15028	0.042995	-3.49522	0.000641	0.999985
cg1069985	-0.23484	0.067205	-3.49439	0.000643	0.999985
cg1066376	0.196023	0.056096	3.494386	0.000643	0.999985
cg0416533	-1.34145	0.383983	-3.49351	0.000645	0.999985
cg1884910	-0.07224	0.02069	-3.49138	0.00065	0.999985
cg1875113	-0.22436	0.064274	-3.4907	0.000651	0.999985
cg0349790	0.146111	0.041871	3.48958	0.000654	0.999985
cg2501526	0.134552	0.038563	3.489177	0.000655	0.999985
cg0682936	0.116047	0.033275	3.48751	0.000658	0.999985
cg2405233	-0.13328	0.03822	-3.48712	0.000659	0.999985
cg0415446	-0.13257	0.038019	-3.48701	0.00066	0.999985
cg1615798	0.109115	0.031321	3.483727	0.000667	0.999985
cg0175975	0.440815	0.126545	3.483466	0.000668	0.999985
cg1678658	-0.13766	0.039523	-3.48305	0.000669	0.999985
cg2476592	0.127177	0.036517	3.482696	0.000669	0.999985
cg1727700	-0.16467	0.047283	-3.4826	0.00067	0.999985
cg1038751	-0.11988	0.034429	-3.48184	0.000671	0.999985
cg1650570	-0.09874	0.028368	-3.48065	0.000674	0.999985
cg1857723	0.218358	0.062737	3.480504	0.000674	0.999985
cg0103736	-0.24454	0.070274	-3.47985	0.000676	0.999985
cg2075617	0.115408	0.033179	3.478382	0.000679	0.999985
cg1739749	0.127267	0.036596	3.477573	0.000681	0.999985
cg0487767	-0.23634	0.067975	-3.47683	0.000683	0.999985
cg1734292	-0.0608	0.017505	-3.47301	0.000692	0.999985
cg0953880	-0.16605	0.047824	-3.47211	0.000694	0.999985
cg0572137	0.220843	0.06362	3.471312	0.000696	0.999985
cg0188555	0.13538	0.039011	3.470358	0.000698	0.999985
cg2344366	-0.11005	0.031712	-3.47028	0.000698	0.999985
cg2303148	-0.09555	0.027583	-3.46414	0.000713	0.999985
cg1189900	0.104455	0.030162	3.463144	0.000716	0.999985

cg1645190	0.163251	0.047157	3.46186	0.000719	0.999985
cg1861597	0.344901	0.099671	3.460404	0.000722	0.999985
cg2128973	0.105194	0.030403	3.459966	0.000723	0.999985
cg1841269	0.137287	0.039736	3.455017	0.000736	0.999985
cg1572494	0.134215	0.038864	3.45348	0.00074	0.999985
cg0399713	0.28944	0.083855	3.451671	0.000744	0.999985
cg1398268	0.156393	0.045312	3.451479	0.000745	0.999985
cg0192573	-0.2006	0.058137	-3.45054	0.000747	0.999985
cg0904017	0.082436	0.023892	3.450359	0.000748	0.999985
cg2124004	-0.0978	0.028345	-3.45028	0.000748	0.999985
cg0561893	0.138999	0.04031	3.448282	0.000753	0.999985
cg0253710	0.108946	0.031602	3.447489	0.000755	0.999985
cg0205979	-0.07619	0.022114	-3.44551	0.00076	0.999985
cg2504305	-0.14906	0.043263	-3.44531	0.000761	0.999985
cg2638187	-0.06918	0.020081	-3.44526	0.000761	0.999985
cg0033051	-0.15598	0.045301	-3.44313	0.000766	0.999985
cg1511579	-0.06833	0.019849	-3.44244	0.000768	0.999985
cg1090071	0.181661	0.05278	3.441864	0.000769	0.999985
cg1118626	0.188723	0.054841	3.441259	0.000771	0.999985
cg1417627	-0.13007	0.037812	-3.43996	0.000774	0.999985
cg0654514	-0.08848	0.02573	-3.43867	0.000778	0.999985
cg1244530	-0.12055	0.035059	-3.43854	0.000778	0.999985
cg0990110	0.147966	0.043055	3.436655	0.000783	0.999985
cg0059324	0.120513	0.035076	3.435792	0.000785	0.999985
cg2702532	-0.15226	0.044318	-3.43555	0.000786	0.999985
cg1498293	0.095342	0.027759	3.434644	0.000789	0.999985
cg2340375	-0.10626	0.030941	-3.43437	0.000789	0.999985
cg1202369	0.109175	0.031797	3.433537	0.000791	0.999985
cg0326311	-0.1535	0.044706	-3.43347	0.000792	0.999985
cg1832899	-0.24313	0.070819	-3.43313	0.000793	0.999985
cg1251002	0.213259	0.062119	3.43305	0.000793	0.999985
cg0303762	0.219999	0.064104	3.431892	0.000796	0.999985
cg1117605	-0.0928	0.027048	-3.43076	0.000799	0.999985
cg1486052	0.140527	0.040963	3.430593	0.000799	0.999985
cg2650816	0.109006	0.031802	3.427655	0.000807	0.999985
cg0733387	-0.0898	0.026207	-3.42674	0.00081	0.999985
cg2066520	0.114886	0.033531	3.426303	0.000811	0.999985
cg2223471	0.307987	0.090053	3.420045	0.000828	0.999985
cg2664037	0.121372	0.035507	3.418258	0.000834	0.999985
cg1970843	-0.12387	0.036246	-3.41747	0.000836	0.999985
cg0388073	-0.08227	0.024081	-3.4164	0.000839	0.999985
cg1820336	-0.15274	0.044713	-3.41602	0.00084	0.999985
cg2233325	0.221606	0.064928	3.413083	0.000848	0.999985
cg2712974	-0.51976	0.152345	-3.41172	0.000852	0.999985
cg1384563	-0.12155	0.035657	-3.40898	0.00086	0.999985
cg0903263	0.149595	0.043885	3.408806	0.000861	0.999985
cg1561254	-0.11458	0.033616	-3.40844	0.000862	0.999985
cg0061262	-0.11321	0.033215	-3.40834	0.000862	0.999985
cg2326380	-0.07744	0.022723	-3.40787	0.000863	0.999985
cg0397665	0.195022	0.057232	3.407545	0.000864	0.999985

cg0678410	-0.15079	0.044273	-3.40603	0.000869	0.999985
cg0030407	0.173615	0.050984	3.405305	0.000871	0.999985
cg0711721	-0.10563	0.031027	-3.40446	0.000873	0.999985
cg0827800	-0.11148	0.03276	-3.40299	0.000878	0.999985
cg0302514	0.118092	0.034704	3.402801	0.000878	0.999985
cg1392522	-0.11815	0.034733	-3.40158	0.000882	0.999985
cg1066537	-0.09532	0.028025	-3.40112	0.000883	0.999985
cg0155819	0.2083	0.061247	3.400966	0.000884	0.999985
cg0176889	0.085429	0.025122	3.400511	0.000885	0.999985
cg2341730	-0.09259	0.027249	-3.39805	0.000892	0.999985
cg1331504	0.257062	0.075656	3.397785	0.000893	0.999985
cg2503738	0.123255	0.036305	3.39498	0.000902	0.999985
cg2652514	0.105578	0.031104	3.394375	0.000903	0.999985
cg1350195	0.160319	0.047237	3.39389	0.000905	0.999985
cg1510914	0.105472	0.031085	3.393055	0.000907	0.999985
cg1088252	0.133975	0.039497	3.392006	0.000911	0.999985
cg0039283	-0.09779	0.028833	-3.3914	0.000912	0.999985
cg1452914	0.138271	0.040778	3.390828	0.000914	0.999985
cg0458982	-0.19136	0.05646	-3.38922	0.000919	0.999985
cg0119180	0.670082	0.197767	3.388245	0.000922	0.999985
cg1849556	0.380777	0.112391	3.387979	0.000923	0.999985
cg2643528	-0.44664	0.132091	-3.38133	0.000944	0.999985
cg2016215	-0.10746	0.031802	-3.37908	0.000951	0.999985
cg1848975	-0.29809	0.088226	-3.37867	0.000952	0.999985
cg2758564	-0.10219	0.030249	-3.37812	0.000954	0.999985
cg1241417	0.42701	0.126452	3.376854	0.000958	0.999985
cg0963193	0.175625	0.052014	3.376506	0.000959	0.999985
cg0436709	-0.10871	0.03221	-3.37489	0.000964	0.999985
cg0716074	-0.21825	0.064687	-3.37393	0.000968	0.999985
cg0308356	0.132269	0.039213	3.373064	0.00097	0.999985
cg2363825	-0.07682	0.022776	-3.37285	0.000971	0.999985
cg0414593	-0.1041	0.03088	-3.3712	0.000976	0.999985
cg0208100	-0.15464	0.045886	-3.37006	0.00098	0.999985
cg0283449	-0.14297	0.042434	-3.36924	0.000983	0.999985
cg1097446	-0.06344	0.018836	-3.36802	0.000987	0.999985
cg1968719	-0.6459	0.191829	-3.36703	0.00099	0.999985
cg1710414	-0.14513	0.043128	-3.36499	0.000997	0.999985
cg2227254	-0.12967	0.038551	-3.36357	0.001002	0.999985
cg1647913	0.121113	0.036008	3.363467	0.001002	0.999985
cg1662940	0.08619	0.025626	3.363332	0.001002	0.999985
cg0458225	0.195533	0.058186	3.360515	0.001012	0.999985
cg0469287	-0.27381	0.081479	-3.3605	0.001012	0.999985
cg0510408	0.162921	0.048492	3.359741	0.001015	0.999985
cg2633124	-0.10131	0.030163	-3.35865	0.001018	0.999985
cg0758602	-0.1284	0.038231	-3.35838	0.001019	0.999985
cg1435748	0.236777	0.070522	3.357493	0.001022	0.999985
cg0056910	-0.21475	0.063992	-3.35595	0.001027	0.999985
cg0576406	-0.12866	0.038361	-3.35386	0.001035	0.999985
cg0384528	-0.1167	0.034795	-3.35384	0.001035	0.999985
cg0739992	-0.23318	0.069552	-3.35259	0.001039	0.999985

cg2410612	-0.10208	0.030455	-3.35168	0.001042	0.999985
cg1596542	0.096591	0.028824	3.351061	0.001044	0.999985
cg1870590	-0.12387	0.036968	-3.35069	0.001046	0.999985
cg0440454	-0.10768	0.032138	-3.35056	0.001046	0.999985
cg1615195	0.845093	0.252257	3.350121	0.001048	0.999985
cg0010202	-0.13811	0.041233	-3.3496	0.001049	0.999985
cg1077851	0.114086	0.03408	3.347567	0.001057	0.999985
cg1258318	0.143488	0.042876	3.346582	0.00106	0.999985
cg1101191	0.096254	0.028768	3.34586	0.001063	0.999985
cg1881558	-0.09208	0.027524	-3.34531	0.001064	0.999985
cg1652530	-0.13281	0.039712	-3.34433	0.001068	0.999985
cg1692548	0.091689	0.027422	3.343621	0.00107	0.999985
cg1460433	0.220535	0.065974	3.342786	0.001073	0.999985
cg0641484	0.112288	0.033592	3.34275	0.001074	0.999985
cg1151600	0.104102	0.031156	3.341303	0.001079	0.999985
cg0002202	-0.20027	0.059948	-3.34081	0.001081	0.999985
cg0794422	-0.1101	0.032961	-3.34017	0.001083	0.999985
cg1111976	0.132946	0.039815	3.339129	0.001087	0.999985
cg2738049	0.088488	0.026515	3.337231	0.001093	0.999985
cg2621954	-0.0533	0.015978	-3.33609	0.001098	0.999985
cg1057721	0.138704	0.041594	3.334707	0.001103	0.999985
cg0480143	0.097322	0.029186	3.334512	0.001103	0.999985
cg1573242	0.172309	0.051706	3.332489	0.001111	0.999985
cg1334219	-0.10007	0.030039	-3.33138	0.001115	0.999985
cg0616085	0.159286	0.047816	3.331226	0.001115	0.999985
cg0324288	0.088125	0.026458	3.330746	0.001117	0.999985
cg0272194	-0.15379	0.046185	-3.32993	0.00112	0.999985
cg1772481	0.126878	0.03811	3.329287	0.001123	0.999985
cg0371685	-0.22866	0.068688	-3.32892	0.001124	0.999985
cg1763237	-0.09878	0.029692	-3.32686	0.001132	0.999985
cg0677615	-0.20609	0.061948	-3.32683	0.001132	0.999985
cg1285213	-0.15512	0.046637	-3.32605	0.001135	0.999985
cg0619480	-0.22793	0.068585	-3.32335	0.001145	0.999985
cg2114244	-0.1227	0.036925	-3.32309	0.001146	0.999985
cg0073360	0.106637	0.032112	3.320768	0.001155	0.999985
cg1175991	0.106392	0.032063	3.318245	0.001164	0.999985
cg1063135	0.118189	0.035644	3.315826	0.001174	0.999985
cg1502300	-0.12362	0.037286	-3.3155	0.001175	0.999985
cg1640587	-0.15005	0.045269	-3.31466	0.001178	0.999985
cg2164294	-0.09408	0.028386	-3.31422	0.00118	0.999985
cg0478008	-0.09935	0.029982	-3.31367	0.001182	0.999985
cg2645222	-0.19518	0.058919	-3.31262	0.001186	0.999985
cg0069719	0.113817	0.034382	3.3104	0.001195	0.999985
cg2627341	0.093578	0.028279	3.309106	0.0012	0.999985
cg1670126	0.146147	0.044173	3.308476	0.001203	0.999985
cg2582846	-0.08864	0.026795	-3.30823	0.001204	0.999985
cg2043866	-0.11773	0.035594	-3.30756	0.001206	0.999985
cg1752338	-0.09129	0.027625	-3.30473	0.001218	0.999985
cg0758182	0.095636	0.028943	3.304329	0.001219	0.999985
cg0410229	-0.19235	0.058213	-3.30426	0.00122	0.999985

cg2689657	0.110032	0.033317	3.302558	0.001226	0.999985
cg2662644	0.300981	0.09116	3.301689	0.00123	0.999985
cg0942031	0.133221	0.040355	3.301193	0.001232	0.999985
cg0657687	-0.07051	0.021368	-3.29976	0.001238	0.999985
cg2101777	0.091839	0.027836	3.29932	0.00124	0.999985
cg0499816	-0.12979	0.039373	-3.29644	0.001251	0.999985
cg1492837	0.900494	0.273208	3.295998	0.001253	0.999985
cg1415485	-0.16221	0.049252	-3.29354	0.001263	0.999985
cg1507681	0.09624	0.029223	3.293303	0.001264	0.999985
cg0692013	0.132657	0.040288	3.292718	0.001267	0.999985
cg0557781	-0.08651	0.026275	-3.29246	0.001268	0.999985
cg0271511	0.102469	0.031126	3.292035	0.00127	0.999985
cg0343258	0.477129	0.144979	3.291031	0.001274	0.999985
cg2484599	-0.12461	0.037878	-3.28984	0.001279	0.999985
cg2321700	0.093713	0.02849	3.289288	0.001281	0.999985
cg1478328	0.136916	0.041634	3.28858	0.001284	0.999985
cg0211077	0.113594	0.034554	3.28741	0.001289	0.999985
cg1193839	0.152999	0.046577	3.28482	0.0013	0.999985
cg0845195	-0.10638	0.032396	-3.28367	0.001305	0.999985
cg1379184	-0.14303	0.043561	-3.28345	0.001306	0.999985
cg0065338	0.125001	0.038072	3.283246	0.001307	0.999985
cg0717224	0.114497	0.034876	3.282949	0.001308	0.999985
cg2354259	-0.17941	0.054653	-3.28275	0.001309	0.999985
cg0002370	0.143354	0.043675	3.282284	0.001311	0.999985
cg1366841	0.119805	0.036502	3.282131	0.001312	0.999985
cg2062790	-0.07294	0.022224	-3.2818	0.001313	0.999985
cg1972537	0.137395	0.041873	3.281247	0.001315	0.999985
cg1942426	0.232644	0.070909	3.280888	0.001317	0.999985
cg0722015	0.126838	0.038663	3.280634	0.001318	0.999985
cg1964563	0.112171	0.0342	3.279829	0.001322	0.999985
cg0002041	0.096449	0.029414	3.278979	0.001325	0.999985
cg0678185	-0.37449	0.114211	-3.2789	0.001326	0.999985
cg0379037	0.098048	0.029905	3.278602	0.001327	0.999985
cg1122476	0.149969	0.045742	3.278583	0.001327	0.999985
cg0404825	0.158078	0.048226	3.277847	0.00133	0.999985
cg1662016	-0.22169	0.06764	-3.27752	0.001332	0.999985
cg0404345	0.889474	0.271434	3.27694	0.001334	0.999985
cg1496048	0.093892	0.028653	3.276875	0.001334	0.999985
cg2632176	0.119397	0.036445	3.276056	0.001338	0.999985
cg2541375	-0.08105	0.024741	-3.27596	0.001338	0.999985
cg0748572	0.189713	0.05793	3.274892	0.001343	0.999985
cg1888438	-0.15844	0.048383	-3.2747	0.001344	0.999985
cg0840012	-0.1062	0.032434	-3.27443	0.001345	0.999985
cg0997338	0.13399	0.040933	3.273396	0.00135	0.999985
cg0236990	-0.09278	0.028352	-3.27224	0.001355	0.999985
cg2269419	-0.1394	0.042622	-3.27064	0.001362	0.999985
cg1987069	-0.1568	0.047957	-3.2696	0.001367	0.999985
cg0999632	-0.09796	0.029975	-3.26785	0.001374	0.999985
cg2700793	-0.16893	0.051708	-3.26706	0.001378	0.999985
cg1440897	0.102247	0.031308	3.265884	0.001383	0.999985

cg2640049	0.574968	0.176085	3.265288	0.001386	0.999985
cg1421586	0.106201	0.032529	3.264861	0.001388	0.999985
cg1193792	0.236181	0.072365	3.263741	0.001393	0.999985
cg0050747	-0.10802	0.033106	-3.26295	0.001397	0.999985
cg0464946	0.190799	0.058495	3.261801	0.001402	0.999985
cg1968479	0.151571	0.046474	3.26142	0.001404	0.999985
cg2147158	-0.3745	0.114845	-3.26093	0.001406	0.999985
cg1875935	-0.0998	0.030608	-3.26047	0.001408	0.999985
cg1571902	-0.11154	0.034209	-3.2604	0.001408	0.999985
cg2134227	0.110995	0.034063	3.258532	0.001417	0.999985
cg1877501	0.126115	0.038722	3.256965	0.001424	0.999985
cg0461037	0.136071	0.04182	3.253708	0.001439	0.999985
cg1863225	0.090871	0.027951	3.251081	0.001452	0.999985
cg1130240	0.15504	0.047692	3.250895	0.001453	0.999985
cg1058564	0.168822	0.051949	3.249775	0.001458	0.999985
cg0925855	-0.1795	0.055241	-3.24941	0.00146	0.999985
cg0253292	-0.12232	0.037649	-3.24902	0.001462	0.999985
cg0880284	0.412351	0.126983	3.247301	0.00147	0.999985
cg1368999	-0.30729	0.094652	-3.24655	0.001473	0.999985
cg0551210	-0.30435	0.09378	-3.24543	0.001479	0.999985
cg2748662	0.125904	0.038801	3.244815	0.001482	0.999985
cg2009140	-0.11368	0.035039	-3.2444	0.001484	0.999985
cg1409815	0.123535	0.03808	3.244097	0.001485	0.999985
cg0376699	0.11883	0.036639	3.243269	0.001489	0.999985
cg2703599	0.456555	0.140791	3.242782	0.001492	0.999985
cg1637773	0.720219	0.222114	3.242569	0.001493	0.999985
cg0803168	-0.09579	0.029543	-3.24229	0.001494	0.999985
cg1152978	-0.09031	0.027859	-3.24157	0.001497	0.999985
cg2161932	-0.07272	0.022438	-3.24088	0.001501	0.999985
cg2347688	0.260528	0.080397	3.24051	0.001503	0.999985
cg1576925	0.090231	0.027853	3.239531	0.001507	0.999985
cg1703841	0.135758	0.041914	3.238939	0.00151	0.999985
cg2727236	-0.1084	0.033477	-3.23816	0.001514	0.999985
cg1676645	0.128308	0.039637	3.237063	0.00152	0.999985
cg1628066	-0.15165	0.046854	-3.23676	0.001521	0.999985
cg0843943	-0.10423	0.032204	-3.23658	0.001522	0.999985
cg0001024	0.121368	0.037507	3.235858	0.001526	0.999985
cg2039841	-0.12776	0.039485	-3.23555	0.001527	0.999985
cg1936675	-0.17165	0.053084	-3.2336	0.001537	0.999985
cg0694170	0.190354	0.058874	3.233239	0.001539	0.999985
cg0526739	0.328234	0.101532	3.2328	0.001541	0.999985
cg1036333	0.417617	0.129194	3.232481	0.001542	0.999985
cg0706522	0.193842	0.059988	3.231322	0.001548	0.999985
cg1049584	-0.17152	0.053106	-3.22984	0.001556	0.999985
cg2258619	0.115551	0.035789	3.228702	0.001561	0.999985
cg2499461	0.110571	0.034252	3.228218	0.001564	0.999985
cg0791420	-0.10048	0.031134	-3.22726	0.001569	0.999985
cg1206701	0.403766	0.12512	3.227026	0.00157	0.999985
cg0760220	-0.09094	0.028186	-3.22636	0.001573	0.999985
cg0161489	-0.15852	0.049137	-3.22611	0.001575	0.999985

cg0224546	-0.11159	0.03459	-3.22604	0.001575	0.999985
cg0372955	0.174183	0.053997	3.225793	0.001576	0.999985
cg0532344	0.114382	0.03546	3.225663	0.001577	0.999985
cg2073152	0.133096	0.041279	3.224276	0.001584	0.999985
cg2307533	-0.08987	0.027887	-3.22274	0.001592	0.999985
cg0258344	0.20392	0.063298	3.221608	0.001598	0.999985
cg1184485	-0.09157	0.02844	-3.21969	0.001608	0.999985
cg1543029	-0.10172	0.031595	-3.21932	0.00161	0.999985
cg0382576	0.091984	0.028575	3.219087	0.001611	0.999985
cg1248989	0.190372	0.059176	3.217029	0.001622	0.999985
cg0719651	0.514386	0.159904	3.216837	0.001623	0.999985
cg1092633	0.109229	0.033959	3.216519	0.001624	0.999985
cg1316715	0.697779	0.21696	3.216163	0.001626	0.999985
cg2182164	0.355057	0.110405	3.215934	0.001627	0.999985
cg0333252	0.224188	0.069769	3.213302	0.001641	0.999985
cg0072011	-0.06963	0.021673	-3.2129	0.001643	0.999985
cg2169062	0.194508	0.060544	3.212661	0.001645	0.999985
cg2435878	-0.1604	0.049947	-3.21135	0.001652	0.999985
cg0687231	0.110279	0.034341	3.211326	0.001652	0.999985
cg0659585	0.101541	0.031635	3.209758	0.00166	0.999985
cg0882181	-0.10149	0.031622	-3.20953	0.001661	0.999985
cg1594072	-0.12553	0.039112	-3.20945	0.001662	0.999985
cg2580435	0.801083	0.249661	3.208685	0.001666	0.999985
cg0799469	0.103953	0.032405	3.207891	0.00167	0.999985
cg1814709	-0.17625	0.054949	-3.20754	0.001672	0.999985
cg1688932	0.138539	0.043192	3.207488	0.001672	0.999985
cg0662148	-0.27518	0.085814	-3.20667	0.001677	0.999985
cg1275757	-0.08171	0.025483	-3.20657	0.001677	0.999985
cg1361533	-0.15466	0.048237	-3.20632	0.001679	0.999985
cg2578196	0.104988	0.03275	3.205759	0.001682	0.999985
cg1793623	-0.09181	0.028646	-3.20508	0.001685	0.999985
cg0412478	-0.12273	0.038302	-3.20429	0.00169	0.999985
cg2067383	1.100311	0.343607	3.202235	0.001701	0.999985
cg2471801	0.154811	0.048352	3.201775	0.001703	0.999985
cg1140121	0.413577	0.129222	3.200514	0.00171	0.999985
cg0802112	0.264012	0.082498	3.200242	0.001712	0.999985
cg0321713	0.167538	0.052359	3.199824	0.001714	0.999985
cg1422193	-0.10372	0.032428	-3.19849	0.001722	0.999985
cg2272764	0.141567	0.044268	3.197947	0.001725	0.999985
cg2743499	0.113923	0.035627	3.197614	0.001726	0.999985
cg1591201	0.124306	0.038884	3.196886	0.00173	0.999985
cg1198898	-0.09822	0.030727	-3.19662	0.001732	0.999985
cg0613133	0.112124	0.035076	3.196583	0.001732	0.999985
cg1923533	0.137384	0.04299	3.195707	0.001737	0.999985
cg0965500	0.174744	0.054682	3.195656	0.001737	0.999985
cg1964775	-0.07956	0.02491	-3.194	0.001747	0.999985
cg0755942	0.086654	0.027132	3.193829	0.001748	0.999985
cg1149636	-0.07515	0.023532	-3.19371	0.001748	0.999985
cg1227158	-0.11723	0.036713	-3.19312	0.001752	0.999985
cg0431155	0.208885	0.065423	3.192816	0.001753	0.999985

cg0957711	0.155241	0.048627	3.192512	0.001755	0.999985
cg0415707	0.081377	0.025494	3.191983	0.001758	0.999985
cg1222776	0.126781	0.039721	3.191767	0.001759	0.999985
cg1533513	-0.22437	0.070298	-3.19175	0.001759	0.999985
cg1233913	0.436094	0.136667	3.190918	0.001764	0.999985
cg2512262	0.167418	0.052475	3.19044	0.001767	0.999985
cg0834463	0.272473	0.085403	3.190427	0.001767	0.999985
cg0597031	-0.1287	0.040343	-3.19007	0.001769	0.999985
cg1426377	0.092342	0.028961	3.188497	0.001778	0.999985
cg1875866	-0.09694	0.03041	-3.18789	0.001781	0.999985
cg2016691	-0.09992	0.031351	-3.18705	0.001786	0.999985
cg1875523	0.08213	0.025772	3.186743	0.001788	0.999985
cg1879466	-0.08012	0.025142	-3.18656	0.001789	0.999985
cg0713953	-0.24929	0.078245	-3.18606	0.001792	0.999985
cg2751640	-0.08858	0.027806	-3.18553	0.001795	0.999985
cg2750136	-0.15571	0.048888	-3.18502	0.001798	0.999985
cg0137459	-0.12444	0.039075	-3.1845	0.001801	0.999985
cg0864451	-0.07094	0.022277	-3.18422	0.001802	0.999985
cg2176689	0.093829	0.02947	3.183892	0.001804	0.999985
cg1308303	-0.1047	0.032889	-3.18359	0.001806	0.999985
cg1225589	-0.17059	0.053588	-3.18331	0.001808	0.999985
cg2685150	-0.10738	0.033734	-3.18311	0.001809	0.999985
cg0563646	0.45059	0.141616	3.181783	0.001817	0.999985
cg2142198	0.065071	0.020454	3.181376	0.001819	0.999985
cg0982051	-0.1065	0.033485	-3.18061	0.001823	0.999985
cg2216209	0.129294	0.040662	3.179746	0.001828	0.999985
cg0748535	0.093791	0.029507	3.178558	0.001835	0.999985
cg1606621	0.121355	0.03819	3.177648	0.001841	0.999985
cg2765114	0.158084	0.049751	3.177507	0.001842	0.999985
cg0481953	-0.08993	0.028308	-3.17679	0.001846	0.999985
cg1789076	-0.09299	0.029272	-3.17669	0.001846	0.999985
cg2698654	-0.10568	0.033271	-3.17646	0.001848	0.999985
cg1243280	0.535004	0.168478	3.17551	0.001853	0.999985
cg0905988	0.198951	0.062666	3.174796	0.001858	0.999985
cg1759198	-0.10677	0.03364	-3.17388	0.001863	0.999985
cg1894586	0.201689	0.063552	3.173622	0.001865	0.999985
cg1909905	-0.12288	0.03872	-3.17354	0.001865	0.999985
cg2156686	0.094572	0.029801	3.173449	0.001866	0.999985
cg2085909	-0.116	0.036554	-3.17331	0.001867	0.999985
cg2279561	-0.09631	0.030357	-3.17265	0.001871	0.999985
cg0634480	0.104377	0.0329	3.17259	0.001871	0.999985
cg0560062	0.252675	0.079652	3.172232	0.001873	0.999985
cg1017393	-0.16718	0.05272	-3.17103	0.00188	0.999985
cg2452659	-0.12551	0.039591	-3.17013	0.001886	0.999985
cg0125546	0.110102	0.034736	3.169713	0.001888	0.999985
cg1263046	0.368949	0.116411	3.169364	0.00189	0.999985
cg0399615	-0.14674	0.046306	-3.16881	0.001894	0.999985
cg0272267	0.082729	0.026111	3.168323	0.001897	0.999985
cg1893197	-0.09016	0.028465	-3.16735	0.001903	0.999985
cg1381641	-0.29542	0.093278	-3.16714	0.001904	0.999985

cg0031966	-0.15831	0.049993	-3.16667	0.001907	0.999985
cg0424686	-0.39876	0.125928	-3.16653	0.001908	0.999985
cg1985027	-0.12481	0.039433	-3.16515	0.001916	0.999985
cg1758609	0.161406	0.051002	3.164692	0.001919	0.999985
ch.10.1028	0.064156	0.020279	3.163704	0.001925	0.999985
cg0890785	-0.10862	0.034334	-3.16357	0.001926	0.999985
cg0221469	-0.11084	0.035036	-3.16349	0.001926	0.999985
cg1679114	-0.166	0.052475	-3.16329	0.001927	0.999985
cg0120127	-0.28824	0.091134	-3.16287	0.00193	0.999985
cg1247809	0.173148	0.054755	3.162263	0.001934	0.999985
cg1322985	-0.15149	0.047912	-3.16192	0.001936	0.999985
cg2542388	-0.0775	0.024511	-3.16169	0.001937	0.999985
cg2683719	-0.15895	0.050278	-3.16145	0.001939	0.999985
cg1472755	-0.10295	0.032577	-3.16034	0.001946	0.999985
cg1678697	-0.17711	0.056049	-3.16002	0.001948	0.999985
cg0954692	-0.09668	0.030599	-3.15967	0.00195	0.999985
cg2082374	0.117991	0.037345	3.159439	0.001951	0.999985
cg2394824	-0.16422	0.051977	-3.15941	0.001951	0.999985
cg2284995	-0.08395	0.026574	-3.15894	0.001954	0.999985
cg0899164	0.176813	0.055975	3.158782	0.001955	0.999985
cg0076988	-0.09264	0.029335	-3.15805	0.00196	0.999985
cg1603151	-0.2537	0.080352	-3.15731	0.001965	0.999985
cg2286642	0.184622	0.058485	3.156724	0.001968	0.999985
cg2326182	0.144684	0.045839	3.156334	0.001971	0.999985
cg0490431	0.177443	0.056225	3.155944	0.001973	0.999985
cg0010797	0.073229	0.023205	3.155813	0.001974	0.999985
cg0594542	-0.11283	0.035756	-3.15549	0.001976	0.999985
cg1444715	-0.10512	0.033328	-3.15402	0.001985	0.999985
cg1777753	-0.08324	0.026392	-3.154	0.001985	0.999985
cg1317007	-0.18375	0.058268	-3.15354	0.001988	0.999985
cg1253895	0.082918	0.0263	3.152744	0.001993	0.999985
cg0251092	-0.07512	0.023829	-3.15265	0.001994	0.999985
cg2526042	-0.08625	0.027358	-3.15253	0.001995	0.999985
cg2130277	-0.10368	0.032896	-3.15181	0.001999	0.999985
cg0954708	-0.08981	0.028517	-3.14934	0.002015	0.999985
cg0141363	-0.09197	0.029206	-3.14918	0.002016	0.999985
cg1246567	0.086747	0.027552	3.148481	0.002021	0.999985
cg0978327	-0.11439	0.036335	-3.14829	0.002022	0.999985
cg0984431	-0.10923	0.034698	-3.14792	0.002024	0.999985
cg0909534	0.163698	0.052007	3.147587	0.002026	0.999985
cg1281993	0.131165	0.041679	3.147043	0.00203	0.999985
cg2553504	0.117659	0.037391	3.146709	0.002032	0.999985
cg2351318	-0.24803	0.078822	-3.14668	0.002032	0.999985
cg1627707	-0.07232	0.022983	-3.14665	0.002032	0.999985
cg0492418	-0.08779	0.0279	-3.14656	0.002033	0.999985
cg1005377	-0.13309	0.042306	-3.14585	0.002038	0.999985
cg0098680	0.082158	0.026119	3.145531	0.00204	0.999985
cg2122335	0.102098	0.03246	3.145394	0.002041	0.999985
cg1322158	-0.11428	0.03634	-3.14459	0.002046	0.999985
cg1703237	-0.08433	0.026823	-3.14403	0.002049	0.999985

cg1168690	0.170263	0.054165	3.143383	0.002054	0.999985
cg1657693	0.953691	0.303398	3.14336	0.002054	0.999985
cg1291026	-0.16024	0.050983	-3.14308	0.002056	0.999985
cg1130014	0.204632	0.06513	3.141893	0.002063	0.999985
cg0330026	-0.09493	0.030217	-3.14176	0.002064	0.999985
cg1614044	0.145066	0.046178	3.141423	0.002067	0.999985
cg0664294	-0.0867	0.027602	-3.14089	0.00207	0.999985
cg0431375	-0.1005	0.032004	-3.1403	0.002074	0.999985
cg0481884	-0.10457	0.033314	-3.13899	0.002083	0.999985
cg1571771	-0.06655	0.021204	-3.13869	0.002085	0.999985
cg0105270	-0.10928	0.034818	-3.13862	0.002085	0.999985
cg0540368	0.28578	0.091057	3.138458	0.002086	0.999985
cg2034584	-0.09194	0.029301	-3.13769	0.002091	0.999985
cg0130323	0.086863	0.027686	3.13746	0.002093	0.999985
cg0597265	0.090493	0.028845	3.137268	0.002094	0.999985
cg0059955	-0.09246	0.029477	-3.13657	0.002099	0.999985
cg0383934	-0.11817	0.037676	-3.1364	0.0021	0.999985
cg0404925	-0.18922	0.06034	-3.1359	0.002103	0.999985
cg2140479	0.235012	0.074953	3.135472	0.002106	0.999985
cg1203278	-0.18627	0.05941	-3.13534	0.002107	0.999985
cg1941574	-1.77803	0.567162	-3.13496	0.002109	0.999985
cg0523389	0.174644	0.055723	3.134145	0.002115	0.999985
cg2510229	0.079393	0.025333	3.133988	0.002116	0.999985
cg2246667	0.245248	0.078264	3.133591	0.002119	0.999985
cg0258026	-0.11823	0.037735	-3.13322	0.002121	0.999985
cg2707497	-0.08546	0.027277	-3.1331	0.002122	0.999985
cg2758401	-0.1234	0.039389	-3.13282	0.002124	0.999985
cg1151970	0.445694	0.142295	3.132185	0.002128	0.999985
cg0098511	-0.14944	0.047716	-3.13189	0.00213	0.999985
ch.17.3384	-0.08672	0.02769	-3.13174	0.002131	0.999985
cg0483217	-0.12569	0.040142	-3.13126	0.002134	0.999985
cg1824891	0.104939	0.033515	3.131143	0.002135	0.999985
cg0157793	-0.16591	0.052993	-3.13076	0.002138	0.999985
cg2277223	-0.21656	0.069175	-3.13055	0.002139	0.999985
cg1014180	-0.08975	0.028671	-3.13027	0.002141	0.999985
cg0418189	0.110982	0.035458	3.129974	0.002143	0.999985
cg1209442	-0.16097	0.051438	-3.12928	0.002148	0.999985
cg2605106	-0.06868	0.021951	-3.12863	0.002152	0.999985
cg2172404	0.158239	0.050582	3.128342	0.002154	0.999985
cg1325742	-0.19994	0.063921	-3.12791	0.002157	0.999985
cg0370267	-0.12734	0.040716	-3.12745	0.00216	0.999985
cg0019671	0.107191	0.034279	3.127012	0.002163	0.999985
cg1617996	0.111479	0.035652	3.126886	0.002164	0.999985
cg1499521	-0.06869	0.021973	-3.12614	0.002169	0.999985
cg0141222	-0.11146	0.035658	-3.12573	0.002172	0.999985
cg0026965	-0.08172	0.026145	-3.12552	0.002173	0.999985
cg1620415	-0.08489	0.027165	-3.12485	0.002178	0.999985
cg2091118	0.096811	0.030981	3.124805	0.002178	0.999985
cg1989614	0.110347	0.035339	3.122507	0.002194	0.999985
cg0577760	-0.1457	0.046662	-3.12249	0.002194	0.999985

cg1581650	0.124011	0.039715	3.122477	0.002195	0.999985
cg1549170	0.291423	0.093356	3.121623	0.0022	0.999985
cg0732676	0.229628	0.073561	3.121622	0.0022	0.999985
cg0560502	-0.08885	0.028465	-3.12151	0.002201	0.999985
cg0237820	0.171297	0.05488	3.121319	0.002203	0.999985
cg0798198	0.061009	0.019547	3.121215	0.002203	0.999985
cg2117008	-0.15334	0.049129	-3.1211	0.002204	0.999985
cg2308277	-0.09392	0.030093	-3.12097	0.002205	0.999985
cg0892671	0.305337	0.097841	3.120734	0.002207	0.999985
cg2018107	0.116773	0.037422	3.120398	0.002209	0.999985
cg1833736	0.098448	0.031555	3.119918	0.002212	0.999985
cg1453533	0.425825	0.136496	3.119685	0.002214	0.999985
cg0445693	-0.09176	0.029417	-3.11943	0.002216	0.999985
cg0639036	0.231186	0.074116	3.119257	0.002217	0.999985
cg0788820	-0.19477	0.062443	-3.11923	0.002217	0.999985
cg1434854	0.111037	0.035598	3.119204	0.002217	0.999985
cg2464039	-0.0681	0.021838	-3.11833	0.002223	0.999985
cg0614646	0.126949	0.040714	3.118033	0.002226	0.999985
cg1639044	0.078274	0.025109	3.117372	0.00223	0.999985
cg1377967	-0.10553	0.033854	-3.11726	0.002231	0.999985
cg2454646	-0.09442	0.030296	-3.11665	0.002235	0.999985
cg2571200	-0.29549	0.094812	-3.11658	0.002236	0.999985
cg0078841	0.181861	0.058357	3.116336	0.002238	0.999985
cg2140789	-0.17737	0.056918	-3.11623	0.002238	0.999985
cg2681506	0.160059	0.051369	3.115886	0.002241	0.999985
cg2383238	-0.16697	0.053596	-3.1153	0.002245	0.999985
cg0233323	0.16502	0.052979	3.114847	0.002248	0.999985
cg2346970	-0.10876	0.034926	-3.11396	0.002254	0.999985
cg1875572	0.157056	0.050437	3.113899	0.002255	0.999985
cg1263057	0.109066	0.035034	3.113127	0.00226	0.999985
cg1415408	-0.5008	0.160877	-3.11296	0.002262	0.999985
cg0502708	-0.10568	0.03395	-3.11287	0.002262	0.999985
cg1654549	-0.14267	0.045834	-3.11283	0.002263	0.999985
cg0841902	-0.095	0.030521	-3.11257	0.002264	0.999985
cg2736801	0.114248	0.036706	3.112538	0.002265	0.999985
cg2184291	0.141144	0.04537	3.110944	0.002276	0.999985
cg0424037	-0.13112	0.04215	-3.11093	0.002276	0.999985
cg0762474	-0.13931	0.044784	-3.1106	0.002278	0.999985
cg2235043	0.115914	0.037268	3.110249	0.002281	0.999985
cg1734527	0.283921	0.091286	3.110232	0.002281	0.999985
cg1984613	-0.08295	0.026676	-3.10945	0.002287	0.999985
cg1182740	-0.10002	0.032167	-3.10924	0.002288	0.999985
cg1811047	-0.08612	0.027703	-3.1085	0.002294	0.999985
cg1808149	-0.10115	0.032548	-3.10774	0.002299	0.999985
cg0121064	-0.10973	0.035313	-3.10725	0.002303	0.999985
cg2218494	-0.09161	0.029481	-3.10724	0.002303	0.999985
cg0617960	-0.10877	0.035015	-3.10622	0.00231	0.999985
cg0852614	0.106579	0.034312	3.106139	0.002311	0.999985
cg1721673	0.088997	0.028655	3.105844	0.002313	0.999985
cg0707769	0.115988	0.037349	3.105524	0.002315	0.999985

cg2027550	-0.1154	0.037166	-3.10505	0.002319	0.999985
cg1885012	0.102289	0.032943	3.105016	0.002319	0.999985
cg0752445	0.189722	0.061107	3.104776	0.002321	0.999985
cg0493153	-0.09975	0.032141	-3.10364	0.002329	0.999985
cg1939153	-0.21861	0.070458	-3.10272	0.002336	0.999985
cg1367908	0.103568	0.033381	3.102605	0.002337	0.999985
cg2702267	-0.09209	0.029684	-3.10215	0.00234	0.999985
cg0253203	0.122369	0.039453	3.101623	0.002344	0.999985
cg1447670	0.224018	0.072257	3.100282	0.002354	0.999985
cg0882156	0.179033	0.057747	3.100273	0.002354	0.999985
cg0084482	-0.09145	0.029505	-3.09939	0.00236	0.999985
cg0636530	0.135193	0.043622	3.09922	0.002362	0.999985
cg0849582	-0.09541	0.030791	-3.09871	0.002366	0.999985
cg0248532	0.113992	0.036788	3.09866	0.002366	0.999985
cg2157519	-0.06654	0.021473	-3.09861	0.002366	0.999985
cg1644005	0.289374	0.093395	3.098374	0.002368	0.999985
cg0746662	0.184278	0.059478	3.098234	0.002369	0.999985
cg2326674	0.093813	0.030287	3.097489	0.002375	0.999985
cg1966422	-0.14338	0.046293	-3.09733	0.002376	0.999985
cg1083725	0.125634	0.040563	3.097276	0.002376	0.999985
cg0019188	0.10676	0.03447	3.097198	0.002377	0.999985
cg1952665	-0.1157	0.037357	-3.09716	0.002377	0.999985
cg1114725	0.124373	0.040175	3.095775	0.002387	0.999985
cg1524703	0.045657	0.014748	3.09573	0.002388	0.999985
cg2089496	-0.14188	0.045839	-3.09529	0.002391	0.999985
cg0180305	0.131965	0.042646	3.09441	0.002398	0.999985
cg2257212	0.079188	0.025592	3.094212	0.002399	0.999985
cg1752025	-0.12431	0.040179	-3.0938	0.002402	0.999985
cg2510599	0.113901	0.03682	3.093425	0.002405	0.999985
cg1101535	0.108127	0.034961	3.092751	0.00241	0.999985
cg0688552	-0.1017	0.032885	-3.09265	0.002411	0.999985
cg0710291	-0.26487	0.085653	-3.0924	0.002413	0.999985
cg1959484	0.150228	0.048586	3.092004	0.002416	0.999985
cg0069511	-0.08858	0.028652	-3.09153	0.00242	0.999985
cg0792689	-0.14958	0.048388	-3.09131	0.002421	0.999985
cg1679689	0.094659	0.030621	3.091303	0.002421	0.999985
cg2459078	0.227782	0.073708	3.090341	0.002429	0.999985
cg1261431	-0.06662	0.021559	-3.09015	0.00243	0.999985
cg1330818	0.123558	0.03999	3.089704	0.002433	0.999985
cg2319675	-0.10513	0.034027	-3.08958	0.002434	0.999985
cg2198357	-0.09841	0.031855	-3.08933	0.002436	0.999985
cg0096676	-0.15987	0.05175	-3.0892	0.002437	0.999985
cg2635859	-0.09764	0.031633	-3.08671	0.002456	0.999985
cg2307470	-0.31163	0.100976	-3.08613	0.002461	0.999985
cg0457032	-0.30115	0.097594	-3.08575	0.002464	0.999985
cg2263284	0.202768	0.065736	3.084569	0.002473	0.999985
cg1248156	0.092746	0.030068	3.084532	0.002473	0.999985
cg1187601	0.086496	0.028042	3.084502	0.002474	0.999985
cg0921211	0.092107	0.029867	3.083859	0.002479	0.999985
cg1974900	0.098198	0.031845	3.083629	0.00248	0.999985

cg0417017	0.096159	0.03119	3.083051	0.002485	0.999985
cg1132343	0.092357	0.029958	3.08292	0.002486	0.999985
cg1637263	-0.22765	0.073845	-3.08287	0.002486	0.999985
cg0584932	-0.12243	0.039728	-3.08173	0.002495	0.999985
cg1169128	-0.15948	0.05175	-3.08166	0.002496	0.999985
cg0133518	-0.22245	0.072193	-3.08135	0.002498	0.999985
cg1331240	0.090493	0.029372	3.080927	0.002501	0.999985
cg0091414	0.198925	0.064574	3.080587	0.002504	0.999985
cg1041826	-0.08564	0.027799	-3.08053	0.002505	0.999985
cg2739025	-0.09737	0.031608	-3.0805	0.002505	0.999985
cg1025155	-0.11974	0.03887	-3.08045	0.002505	0.999985
cg0316517	-0.19154	0.062185	-3.0801	0.002508	0.999985
cg1614056	1.114242	0.361815	3.079592	0.002512	0.999985
cg1416483	0.175486	0.056986	3.079454	0.002513	0.999985
cg2118346	-0.09014	0.029278	-3.07896	0.002517	0.999985
cg1804207	0.097092	0.031538	3.078571	0.00252	0.999985
cg0174359	-0.14813	0.048118	-3.07839	0.002521	0.999985
cg1511134	-0.11597	0.037674	-3.07814	0.002523	0.999985
cg1201527	0.085647	0.027826	3.077968	0.002525	0.999985
cg0589852	-0.06859	0.022291	-3.07723	0.002531	0.999985
cg1179890	-0.15333	0.049831	-3.07704	0.002532	0.999985
cg2674244	-0.1289	0.041892	-3.07686	0.002534	0.999985
cg0625522	-0.10939	0.035552	-3.07685	0.002534	0.999985
cg0722014	0.083644	0.027194	3.075802	0.002542	0.999985
cg0993679	-0.13165	0.042807	-3.07538	0.002545	0.999985
cg1229895	-0.16319	0.053065	-3.07534	0.002546	0.999985
cg0367090	-0.11415	0.037121	-3.07517	0.002547	0.999985
cg1472648	-0.07887	0.025658	-3.07396	0.002557	0.999985
cg1367581	0.107663	0.035026	3.073782	0.002558	0.999985
cg2064495	0.098535	0.032066	3.072922	0.002565	0.999985
cg2130089	-0.25154	0.081859	-3.07281	0.002566	0.999985
cg2180610	-0.09036	0.029408	-3.07246	0.002569	0.999985
cg2046976	-0.15442	0.050284	-3.07088	0.002581	0.999985
cg1418407	0.110195	0.035887	3.070591	0.002584	0.999985
cg0674888	0.10373	0.033797	3.069201	0.002595	0.999985
cg0056387	0.136907	0.044608	3.069135	0.002595	0.999985
cg1327334	0.085517	0.027871	3.068276	0.002602	0.999985
cg1175154	0.144544	0.047114	3.067968	0.002605	0.999985
cg0256350	-0.07496	0.024435	-3.06758	0.002608	0.999985
cg1027633	-0.1079	0.035176	-3.06745	0.002609	0.999985
cg1422567	-0.12456	0.040616	-3.06689	0.002614	0.999985
cg0630129	0.08965	0.029234	3.066584	0.002616	0.999985
cg0154480	0.063926	0.020846	3.066561	0.002616	0.999985
cg2079122	0.17086	0.055725	3.066103	0.00262	0.999985
cg0314475	-0.09159	0.029872	-3.06609	0.00262	0.999985
cg0987717	0.120579	0.039328	3.066024	0.002621	0.999985
cg0831517	-0.07712	0.025159	-3.06513	0.002628	0.999985
cg0149919	-0.06925	0.022593	-3.06508	0.002629	0.999985
cg0517206	-0.11646	0.038	-3.06484	0.002631	0.999985
cg0993039	0.084284	0.02751	3.06376	0.002639	0.999985

cg0530849	-0.22083	0.072095	-3.06308	0.002645	0.999985
cg0578151	0.121643	0.039722	3.062359	0.002651	0.999985
cg2294963	-0.08661	0.028297	-3.06071	0.002665	0.999985
cg1800002	-0.13431	0.043886	-3.06036	0.002668	0.999985
cg0471552	-0.11365	0.037139	-3.06024	0.002669	0.999985
cg0209617	-0.53352	0.174359	-3.05989	0.002672	0.999985
cg1623433	-0.20208	0.066047	-3.05956	0.002674	0.999985
cg1734117	0.108107	0.035335	3.059525	0.002675	0.999985
cg0882773	0.124481	0.040696	3.058786	0.002681	0.999985
cg2348061	0.570762	0.18663	3.058245	0.002685	0.999985
cg2159280	0.111262	0.036383	3.058103	0.002686	0.999985
cg1631872	0.125602	0.041079	3.057545	0.002691	0.999985
cg0264906	-0.48592	0.158945	-3.05714	0.002694	0.999985
cg2246721	-0.05657	0.018507	-3.05659	0.002699	0.999985
cg1750766	-0.12359	0.040441	-3.05612	0.002703	0.999985
cg1524236	-0.10981	0.035932	-3.05591	0.002705	0.999985
cg1914611	-0.16369	0.053571	-3.05557	0.002708	0.999985
cg0405931	-0.11525	0.037725	-3.05503	0.002712	0.999985
cg0859705	0.113159	0.037041	3.054995	0.002713	0.999985
cg2109034	-0.16475	0.053942	-3.05426	0.002719	0.999985
cg1161575	-0.09571	0.031345	-3.05358	0.002725	0.999985
cg0846348	0.078986	0.025876	3.052461	0.002734	0.999985
cg0258426	0.341938	0.112028	3.052267	0.002736	0.999985
cg0706799	0.151738	0.049717	3.052052	0.002738	0.999985
cg0502092	0.087473	0.028661	3.052025	0.002738	0.999985
cg0610047	0.135917	0.044536	3.051862	0.002739	0.999985
cg1788060	0.140517	0.04605	3.051369	0.002743	0.999985
cg0646088	0.117826	0.038624	3.050585	0.00275	0.999985
cg1240668	0.404964	0.132765	3.050238	0.002753	0.999985
cg1198041	0.111099	0.036433	3.049419	0.00276	0.999985
cg1355654	0.147041	0.048234	3.048485	0.002768	0.999985
cg0048222	0.152702	0.050093	3.04839	0.002769	0.999985
cg1567698	-0.07988	0.026207	-3.04789	0.002773	0.999985
cg1636654	0.207367	0.068038	3.047802	0.002774	0.999985
cg1518144	-0.10222	0.033543	-3.04752	0.002776	0.999985
cg1110912	-0.1074	0.03525	-3.04688	0.002782	0.999985
cg1366854	0.168188	0.055208	3.046463	0.002786	0.999985
cg2666178	0.089373	0.029338	3.04638	0.002786	0.999985
cg2493252	0.12603	0.041372	3.046292	0.002787	0.999985
cg0956507	0.212055	0.069611	3.046278	0.002787	0.999985
cg1277747	0.393097	0.129065	3.045722	0.002792	0.999985
cg0610061	-0.15171	0.049816	-3.04528	0.002796	0.999985
cg1698063	-0.08874	0.029146	-3.04468	0.002801	0.999985
cg0444672	0.227597	0.074753	3.044661	0.002801	0.999985
cg0070254	-0.1247	0.04096	-3.04436	0.002804	0.999985
cg0496488	0.211915	0.069614	3.044158	0.002806	0.999985
cg1454887	-0.09447	0.031038	-3.04385	0.002808	0.999985
cg0529570	-0.09446	0.031044	-3.04268	0.002819	0.999985
cg1808376	-0.27747	0.091205	-3.04225	0.002822	0.999985
cg2710512	0.440994	0.144958	3.042222	0.002823	0.999985

cg2467607	0.115209	0.03788	3.041458	0.002829	0.999985
cg1591194	-0.13765	0.04526	-3.04138	0.00283	0.999985
cg1749309	-0.13211	0.043444	-3.04092	0.002834	0.999985
cg2000086	0.07889	0.025945	3.040623	0.002837	0.999985
cg1094037	-0.14376	0.047283	-3.0405	0.002838	0.999985
cg2669860	-0.09514	0.031293	-3.04039	0.002839	0.999985
cg0429194	-0.12625	0.041529	-3.04006	0.002842	0.999985
cg2483142	-0.23827	0.078384	-3.0398	0.002844	0.999985
cg2124035	-0.09066	0.029825	-3.03966	0.002845	0.999985
cg1645102	-0.11844	0.038968	-3.03944	0.002847	0.999985
cg0159763	-0.11114	0.036585	-3.03797	0.00286	0.999985
cg0705753	-0.10903	0.03589	-3.03788	0.002861	0.999985
cg0666937	0.263984	0.086921	3.037059	0.002868	0.999985
cg0888713	-0.0962	0.031677	-3.03703	0.002868	0.999985
cg2236678	0.09107	0.02999	3.036725	0.002871	0.999985
cg0245538	-0.0488	0.016072	-3.03631	0.002875	0.999985
cg2148763	-0.08743	0.0288	-3.03586	0.002879	0.999985
cg0274514	-0.1068	0.035183	-3.03559	0.002881	0.999985
cg1347102	0.110882	0.03653	3.03534	0.002884	0.999985
cg2154437	-0.09333	0.030758	-3.03451	0.002891	0.999985
cg0085348	0.235321	0.07755	3.034443	0.002892	0.999985
cg2548986	-0.14647	0.048272	-3.03432	0.002893	0.999985
cg2014005	0.10217	0.033675	3.033999	0.002896	0.999985
cg1618008	0.100828	0.033237	3.03356	0.002899	0.999985
cg2615183	-0.12406	0.040899	-3.03323	0.002902	0.999985
cg2400716	-0.12999	0.042858	-3.03308	0.002904	0.999985
cg0400434	-0.1992	0.06568	-3.03286	0.002906	0.999985
cg2040609	-0.13097	0.043183	-3.03284	0.002906	0.999985
cg1966784	0.08277	0.027293	3.032666	0.002908	0.999985
cg2672634	0.108863	0.035901	3.03235	0.00291	0.999985
cg0117133	0.154419	0.050948	3.03091	0.002923	0.999985
cg1578682	-0.12894	0.04257	-3.02876	0.002943	0.999985
cg0749068	-0.17796	0.058759	-3.02865	0.002944	0.999985
cg2249337	-0.06863	0.022666	-3.02811	0.002949	0.999985
cg0597118	-0.12605	0.041628	-3.02808	0.002949	0.999985
cg1347071	0.190559	0.062934	3.02791	0.002951	0.999985
cg1377751	-0.14085	0.046518	-3.02782	0.002951	0.999985
cg1513721	0.097729	0.032277	3.02779	0.002952	0.999985
cg0584075	-0.1232	0.040696	-3.02739	0.002955	0.999985
cg0580108	-0.06731	0.022234	-3.02731	0.002956	0.999985
cg2282194	-0.17457	0.057667	-3.02726	0.002957	0.999985
cg0939338	0.184407	0.060917	3.027162	0.002958	0.999985
cg1305500	0.104056	0.034378	3.026803	0.002961	0.999985
cg2668031	0.61347	0.202692	3.026611	0.002963	0.999985
cg0743523	-0.15532	0.05132	-3.02641	0.002964	0.999985
cg2717315	0.079179	0.026164	3.026225	0.002966	0.999985
cg1784389	-0.20465	0.067646	-3.02524	0.002975	0.999985
cg0389585	-0.19194	0.063447	-3.02517	0.002976	0.999985
cg2490181	-0.08705	0.028779	-3.0249	0.002978	0.999985
cg0399833	-0.38802	0.128279	-3.02477	0.002979	0.999985

cg2351879	0.090571	0.029943	3.024755	0.00298	0.999985
cg1479200	0.106569	0.035233	3.024666	0.00298	0.999985
cg1790938	-0.07854	0.025971	-3.02437	0.002983	0.999985
cg2680278	-0.09429	0.03118	-3.02418	0.002985	0.999985
cg1889244	0.121307	0.040117	3.023823	0.002988	0.999985
cg2673109	0.094801	0.031364	3.02256	0.003	0.999985
cg0368695	0.119393	0.039501	3.022513	0.003	0.999985
cg0961830	0.097619	0.032299	3.022392	0.003001	0.999985
cg0347279	0.125076	0.041384	3.022332	0.003002	0.999985
cg2089827	0.112405	0.037195	3.022076	0.003004	0.999985
cg1448470	-0.10676	0.035325	-3.02207	0.003004	0.999985
cg0245851	-0.09388	0.031068	-3.02173	0.003008	0.999985
cg0741768	0.222759	0.07374	3.020886	0.003015	0.999985
cg1599372	-0.12035	0.039841	-3.02077	0.003017	0.999985
cg0989021	-0.09008	0.02982	-3.02073	0.003017	0.999985
cg1353548	-0.10125	0.03352	-3.02072	0.003017	0.999985
cg2288555	-0.07732	0.025599	-3.02045	0.00302	0.999985
cg2641122	-0.14363	0.047555	-3.02028	0.003021	0.999985
cg2244594	-0.09992	0.033084	-3.02005	0.003023	0.999985
cg2190092	0.099017	0.032788	3.019952	0.003024	0.999985
cg1272064	-0.07339	0.024302	-3.01978	0.003026	0.999985
cg0786196	-0.09942	0.032924	-3.01974	0.003026	0.999985
cg0130542	0.114053	0.037779	3.018982	0.003033	0.999985
cg0701712	0.119266	0.039507	3.018831	0.003035	0.999985
cg0662623	-0.09787	0.032421	-3.01863	0.003037	0.999985
cg2680153	-0.13511	0.044762	-3.01853	0.003037	0.999985
cg0512402	0.259215	0.085881	3.01831	0.00304	0.999985
cg0988566	0.099065	0.032824	3.018097	0.003042	0.999985
cg0731860	0.107936	0.035778	3.016806	0.003054	0.999985
cg0743535	0.117882	0.039075	3.016785	0.003054	0.999985
cg0936880	-0.09897	0.032811	-3.01647	0.003057	0.999985
cg1963989	-0.16066	0.053266	-3.01611	0.00306	0.999985
cg0407077	-0.10117	0.03355	-3.01555	0.003066	0.999985
cg2588994	-0.15303	0.050761	-3.01467	0.003074	0.999985
cg0203927	0.103543	0.034351	3.014269	0.003078	0.999985
cg2695874	-0.12781	0.042402	-3.01412	0.003079	0.999985
cg2277724	-0.15464	0.051309	-3.014	0.00308	0.999985
cg2229126	0.200574	0.066566	3.013171	0.003088	0.999985
cg0832377	-0.08963	0.029746	-3.01316	0.003088	0.999985
cg2538871	0.16296	0.054084	3.013103	0.003089	0.999985
cg0747633	-0.19592	0.065024	-3.01308	0.003089	0.999985
cg0335470	-0.10486	0.034821	-3.01145	0.003105	0.999985
cg2710711	0.061381	0.020383	3.01137	0.003105	0.999985
cg1505993	0.134604	0.0447	3.011289	0.003106	0.999985
cg0096515	0.09042	0.030028	3.011193	0.003107	0.999985
cg0732177	0.346867	0.115236	3.010058	0.003118	0.999985
cg0910323	-0.0804	0.026713	-3.0099	0.003119	0.999985
cg1737765	0.094806	0.031499	3.009791	0.003121	0.999985
cg0991939	-0.16467	0.05472	-3.00931	0.003125	0.999985
cg1402435	0.142205	0.047261	3.008956	0.003129	0.999985

cg1517315	-0.08102	0.026928	-3.00874	0.003131	0.999985
cg1964756	0.057664	0.019166	3.008655	0.003131	0.999985
cg0259214	0.103735	0.034481	3.008489	0.003133	0.999985
cg2141857	-0.105	0.034903	-3.00831	0.003135	0.999985
cg1859668	0.087951	0.029241	3.007829	0.003139	0.999985
cg2633345	0.064552	0.021464	3.007461	0.003143	0.999985
cg0265869	-0.12578	0.041825	-3.00733	0.003144	0.999985
cg2392152	0.091221	0.030336	3.007027	0.003147	0.999985
cg2689137	-0.09565	0.031825	-3.00549	0.003162	0.999985
cg0847171	-0.36624	0.121866	-3.0053	0.003164	0.999985
cg1857761	-0.1097	0.036501	-3.00524	0.003165	0.999985
cg0884981	0.885505	0.294661	3.005163	0.003165	0.999985
cg0659563	0.112	0.037272	3.004923	0.003168	0.999985
cg0732117	-0.12011	0.039978	-3.00441	0.003173	0.999985
cg1724177	0.159838	0.053205	3.004199	0.003175	0.999985
cg1474957	-0.12904	0.042957	-3.00386	0.003178	0.999985
cg0826865	-0.11999	0.03995	-3.00353	0.003181	0.999985
cg0412437	0.082538	0.02749	3.002467	0.003192	0.999985
cg1583054	-0.08463	0.028187	-3.00229	0.003193	0.999985
cg2301592	-0.16537	0.055082	-3.00219	0.003194	0.999985
cg2149160	-0.08374	0.027894	-3.0021	0.003195	0.999985
cg2382748	-0.11548	0.038471	-3.00181	0.003198	0.999985
cg2222542	0.124593	0.041512	3.001371	0.003203	0.999985
cg0884751	-0.08918	0.029712	-3.00136	0.003203	0.999985
cg0376239	-0.08115	0.02704	-3.00133	0.003203	0.999985
cg2306781	0.137041	0.045665	3.00103	0.003206	0.999985
cg2611410	0.10242	0.034137	3.000231	0.003214	0.999985
cg0133045	-0.08895	0.029651	-2.99996	0.003216	0.999985
cg0959081	-0.1083	0.036106	-2.99946	0.003221	0.999985
cg1308441	-0.1523	0.050778	-2.99928	0.003223	0.999985
cg2372310	0.174656	0.058234	2.999194	0.003224	0.999985
cg1246109	0.077114	0.025712	2.999163	0.003224	0.999985
cg0413984	-0.13118	0.043738	-2.99912	0.003225	0.999985
cg1468269	-0.16851	0.056186	-2.99911	0.003225	0.999985
cg1310730	0.116011	0.038688	2.99867	0.003229	0.999985
cg0302997	0.212529	0.070875	2.998623	0.00323	0.999985
cg0445359	-0.07377	0.02461	-2.99749	0.003241	0.999985
cg0945725	0.147151	0.049102	2.996848	0.003247	0.999985
cg2693572	-0.09535	0.031823	-2.99639	0.003252	0.999985
cg0119192	-0.25746	0.085956	-2.9953	0.003263	0.999985
cg0661538	-0.10324	0.034467	-2.99518	0.003264	0.999985
cg1516523	0.150512	0.050256	2.994891	0.003267	0.999985
cg2732143	-0.17034	0.056885	-2.99457	0.00327	0.999985
cg2467900	-0.07932	0.026487	-2.99454	0.00327	0.999985
cg2562854	0.522721	0.174587	2.994051	0.003275	0.999985
cg0679542	0.104324	0.034852	2.993336	0.003283	0.999985
cg1954906	-0.09666	0.032295	-2.99292	0.003287	0.999985
cg2389129	-0.12168	0.040658	-2.9927	0.003289	0.999985
cg2282221	-0.09855	0.032929	-2.99262	0.00329	0.999985
cg1440766	0.128902	0.043074	2.992542	0.003291	0.999985

cg1880275	-0.15865	0.053017	-2.99253	0.003291	0.999985
cg2398697	-0.20617	0.068899	-2.99235	0.003293	0.999985
cg2216838	0.171826	0.057423	2.992273	0.003293	0.999985
cg0797483	-0.27674	0.092484	-2.99224	0.003294	0.999985
cg0470665	-0.14159	0.047333	-2.99136	0.003303	0.999985
cg0505397	0.142672	0.047696	2.991297	0.003303	0.999985
cg1160957	0.143596	0.048007	2.991146	0.003305	0.999985
cg0013262	-0.08213	0.027458	-2.99096	0.003307	0.999985
cg0856417	-0.09964	0.033319	-2.99038	0.003312	0.999985
cg1460737	0.218104	0.072978	2.988615	0.00333	0.999985
cg0132090	0.096994	0.032458	2.988273	0.003334	0.999985
cg2388070	-0.10234	0.034249	-2.98824	0.003334	0.999985
cg2332672	0.123219	0.041235	2.988208	0.003335	0.999985
cg0322167	-0.08328	0.027883	-2.98687	0.003348	0.999985
cg1122825	-0.07154	0.023955	-2.98655	0.003352	0.999985
cg0526598	-0.11719	0.039254	-2.98536	0.003364	0.999985
cg0513401	0.159329	0.053395	2.983998	0.003378	0.999985
cg0869073	0.10254	0.034367	2.983657	0.003381	0.999985
cg2208270	0.094758	0.03176	2.983621	0.003382	0.999985
cg1248518	-0.17247	0.057814	-2.98325	0.003386	0.999985
cg0089012	0.306545	0.10277	2.982833	0.00339	0.999985
cg1935638	0.074337	0.024924	2.982555	0.003393	0.999985
cg0432856	-0.08222	0.02757	-2.98208	0.003398	0.999985
cg1970534	-0.08462	0.028379	-2.98183	0.0034	0.999985
cg2446753	-0.16595	0.05567	-2.98098	0.003409	0.999985
cg0047519	0.137501	0.04615	2.979429	0.003426	0.999985
cg2706323	0.113858	0.038215	2.979392	0.003426	0.999985
cg0788574	-0.14898	0.05001	-2.97898	0.00343	0.999985
cg1343645	-0.12191	0.040928	-2.9786	0.003434	0.999985
cg2026276	-0.07169	0.02407	-2.97849	0.003435	0.999985
cg0979508	-0.1533	0.051481	-2.97782	0.003442	0.999985
cg0141656	-0.2214	0.074374	-2.97682	0.003453	0.999985
cg0856567	0.094747	0.031829	2.97673	0.003454	0.999985
cg1062536	0.177667	0.059687	2.976648	0.003455	0.999985
cg0950397	-0.08537	0.02868	-2.97651	0.003456	0.999985
cg2089284	-0.13145	0.044171	-2.97596	0.003462	0.999985
cg0749540	0.688204	0.231256	2.975943	0.003462	0.999985
cg1772512	0.191985	0.064514	2.975846	0.003463	0.999985
cg0231997	0.136775	0.045965	2.97564	0.003465	0.999985
cg0189072	0.345448	0.116117	2.975002	0.003472	0.999985
cg1495014	-0.07205	0.024221	-2.9746	0.003476	0.999985
cg0541849	-0.12644	0.042508	-2.97459	0.003477	0.999985
cg0839697	-0.05673	0.019077	-2.97392	0.003484	0.999985
cg0108583	0.148961	0.05009	2.973889	0.003484	0.999985
cg0576920	-0.09577	0.032207	-2.97369	0.003486	0.999985
cg1743138	-0.13102	0.044066	-2.97331	0.00349	0.999985
cg0304684	0.091902	0.030915	2.972779	0.003496	0.999985
cg1397517	0.154457	0.051969	2.972098	0.003503	0.999985
cg0317136	-0.10469	0.035237	-2.97089	0.003516	0.999985
cg2492540	-0.09106	0.030653	-2.97081	0.003517	0.999985

cg1129431	0.135433	0.045592	2.970524	0.00352	0.999985
cg1555442	-0.09257	0.031165	-2.97039	0.003521	0.999985
cg0618710	0.093893	0.031617	2.969659	0.003529	0.999985
cg0174687	-0.11903	0.040084	-2.96954	0.003531	0.999985
cg1244224	0.329127	0.110846	2.969233	0.003534	0.999985
cg2348179	-0.05395	0.01817	-2.96909	0.003535	0.999985
cg0082721	-0.1782	0.060022	-2.96895	0.003537	0.999985
cg1260243	0.119611	0.040288	2.968911	0.003537	0.999985
cg1803913	-0.1311	0.044164	-2.96848	0.003542	0.999985
cg1080681	0.104505	0.035209	2.968122	0.003546	0.999985
cg2610641	0.103252	0.034795	2.967481	0.003553	0.999985
cg1176262	0.103493	0.03488	2.967105	0.003557	0.999985
cg0670920	0.106477	0.035888	2.966957	0.003559	0.999985
cg0632082	-0.11436	0.038545	-2.9669	0.003559	0.999985
cg2581568	-0.10671	0.035969	-2.96678	0.00356	0.999985
cg1996784	0.102497	0.034552	2.966469	0.003564	0.999985
cg1401940	-0.17838	0.060138	-2.96612	0.003568	0.999985
cg2352549	-0.15396	0.051909	-2.96599	0.003569	0.999985
cg2155410	0.114538	0.038623	2.965536	0.003574	0.999985
cg0917487	-0.14157	0.047746	-2.96506	0.003579	0.999985
cg2638522	-0.10811	0.036466	-2.96458	0.003584	0.999985
cg2145438	0.118655	0.040026	2.964437	0.003586	0.999985
cg1009293	-0.08586	0.028963	-2.96434	0.003587	0.999985
cg1219383	0.263788	0.088988	2.964291	0.003588	0.999985
cg1135485	-0.10577	0.035688	-2.96368	0.003594	0.999985
cg0673622	0.430953	0.145412	2.963665	0.003594	0.999985
cg2633870	0.118233	0.039895	2.96363	0.003595	0.999985
cg0130256	-0.29677	0.100139	-2.96358	0.003595	0.999985
cg2522191	-0.07907	0.026683	-2.96348	0.003596	0.999985
cg2656157	-0.07809	0.026352	-2.9634	0.003597	0.999985
cg2715967	0.409752	0.13828	2.9632	0.0036	0.999985
cg1712940	0.117728	0.03975	2.961744	0.003616	0.999985
cg1665519	0.114425	0.038639	2.96135	0.00362	0.999985
cg2317439	-0.16129	0.054465	-2.96135	0.00362	0.999985
cg1049997	-0.09782	0.033032	-2.9613	0.00362	0.999985
cg1080893	-0.0922	0.031134	-2.96127	0.003621	0.999985
cg2380058	0.104878	0.035427	2.960414	0.00363	0.999985
cg2736191	0.091476	0.030901	2.960341	0.003631	0.999985
cg2762053	0.062274	0.021036	2.960275	0.003632	0.999985
cg0423188	-0.13093	0.044231	-2.9602	0.003633	0.999985
cg0223355	-0.08436	0.028501	-2.9601	0.003634	0.999985
cg2583212	0.121643	0.041098	2.959841	0.003637	0.999985
cg1738003	-0.10657	0.03601	-2.9595	0.00364	0.999985
cg2358002	0.203485	0.068764	2.959177	0.003644	0.999985
cg1297390	-0.07975	0.026952	-2.95911	0.003645	0.999985
cg1125185	0.162185	0.054816	2.958707	0.003649	0.999985
cg2023777	-0.11068	0.037411	-2.95857	0.003651	0.999985
cg1192788	-0.15344	0.051864	-2.95841	0.003652	0.999985
cg0350919	-0.15986	0.054037	-2.95836	0.003653	0.999985
cg1666576	-0.05207	0.017602	-2.95833	0.003653	0.999985

cg1846304	-0.11967	0.040454	-2.95823	0.003654	0.999985
cg1572964	0.151478	0.051211	2.9579	0.003658	0.999985
cg0804140	0.166247	0.056207	2.957774	0.00366	0.999985
cg1183644	0.103915	0.035136	2.957508	0.003662	0.999985
cg0301589	0.139264	0.04709	2.957407	0.003664	0.999985
cg2187066	-0.19577	0.066216	-2.9566	0.003673	0.999985
cg1918746	0.324897	0.109895	2.956415	0.003675	0.999985
cg1143134	-0.09672	0.032717	-2.95623	0.003677	0.999985
cg2745269	-0.10928	0.036968	-2.95604	0.003679	0.999985
cg2680098	-0.08113	0.027448	-2.95575	0.003682	0.999985
cg0936353	0.13083	0.044266	2.955548	0.003684	0.999985
cg1461623	-0.18939	0.064086	-2.95522	0.003688	0.999985
cg0108700	-0.05942	0.020108	-2.9552	0.003688	0.999985
cg0215999	0.091203	0.030868	2.954584	0.003695	0.999985
cg1925451	0.14705	0.049775	2.954313	0.003698	0.999985
cg1673568	-0.13712	0.046417	-2.95414	0.0037	0.999985
cg2209059	-0.08998	0.030461	-2.95392	0.003703	0.999985
cg1428634	0.094669	0.03205	2.953806	0.003704	0.999985
cg2111534	0.074082	0.025083	2.953495	0.003707	0.999985
cg0255717	-0.21563	0.073028	-2.95271	0.003716	0.999985
cg1458367	0.141877	0.048059	2.952142	0.003723	0.999985
cg1631146	0.098393	0.033331	2.952024	0.003724	0.999985
cg1297645	0.094982	0.032178	2.951798	0.003727	0.999985
cg1784868	-0.07718	0.026154	-2.9511	0.003735	0.999985
cg0882853	0.094607	0.032062	2.950723	0.003739	0.999985
cg0787286	0.129877	0.044032	2.949619	0.003751	0.999985
cg1800948	-0.36649	0.124256	-2.94946	0.003753	0.999985
cg0033445	-0.14229	0.048249	-2.94899	0.003759	0.999985
cg2553122	-0.10559	0.035812	-2.94837	0.003766	0.999985
cg0136647	-0.09429	0.031982	-2.94819	0.003768	0.999985
cg2680730	0.065763	0.022307	2.948102	0.003769	0.999985
cg0896630	-0.10868	0.036868	-2.94797	0.00377	0.999985
cg1447979	-0.16553	0.056149	-2.94794	0.003771	0.999985
cg2145247	0.126634	0.042957	2.947928	0.003771	0.999985
cg1830358	0.096637	0.032783	2.947761	0.003773	0.999985
cg0459072	-0.08236	0.027941	-2.94769	0.003773	0.999985
cg0852247	1.708745	0.579718	2.947544	0.003775	0.999985
cg1189773	-0.08061	0.027349	-2.94735	0.003777	0.999985
cg2625642	-0.0544	0.018458	-2.94694	0.003782	0.999985
cg0227257	0.225391	0.076489	2.946714	0.003785	0.999985
cg2303617	-0.07357	0.024969	-2.94661	0.003786	0.999985
cg1155847	0.117999	0.040051	2.946193	0.003791	0.999985
cg2134899	-0.11744	0.039866	-2.94595	0.003793	0.999985
cg1403394	0.159407	0.054139	2.944406	0.003811	0.999985
cg2623479	-0.15637	0.053108	-2.94437	0.003812	0.999985
cg0379860	-0.16534	0.056159	-2.9442	0.003814	0.999985
cg0562372	-0.09206	0.031267	-2.94413	0.003814	0.999985
cg0194900	-0.10797	0.036672	-2.94411	0.003815	0.999985
cg0148240	0.100973	0.034297	2.944062	0.003815	0.999985
cg0584028	0.593623	0.201661	2.943666	0.00382	0.999985

cg2622722	0.31744	0.107865	2.942932	0.003828	0.999985
cg2655828	-0.08178	0.027795	-2.94235	0.003835	0.999985
cg0838249	0.088375	0.030036	2.942307	0.003836	0.999985
cg1321379	-0.20683	0.070302	-2.94197	0.003839	0.999985
cg1498131	0.103968	0.035341	2.94183	0.003841	0.999985
cg2038286	-0.12873	0.043765	-2.94147	0.003845	0.999985
cg1248574	-0.08225	0.027966	-2.9412	0.003848	0.999985
cg2203353	0.143676	0.048852	2.941031	0.00385	0.999985
cg0253714	0.258856	0.088023	2.940764	0.003854	0.999985
cg1371632	-0.13815	0.046978	-2.94067	0.003855	0.999985
cg2616214	0.148073	0.050356	2.940513	0.003856	0.999985
cg2054817	0.071309	0.024253	2.940178	0.00386	0.999985
cg1752742	-0.11454	0.038959	-2.93988	0.003864	0.999985
cg1653850	0.157142	0.053481	2.938291	0.003883	0.999985
cg2722728	0.115802	0.039414	2.938131	0.003884	0.999985
cg0952253	-0.07481	0.025463	-2.93792	0.003887	0.999985
cg1630739	-0.10218	0.034788	-2.93724	0.003895	0.999985
cg2205568	-0.07835	0.026678	-2.93704	0.003897	0.999985
cg0460639	-0.11921	0.04059	-2.93683	0.0039	0.999985
cg0144609	0.099522	0.033894	2.9363	0.003906	0.999985
cg1463189	-0.07584	0.025832	-2.93602	0.003909	0.999985
cg1003211	-0.19287	0.065697	-2.93569	0.003913	0.999985
cg0114281	-0.07099	0.024192	-2.93447	0.003928	0.999985
cg1202910	0.12004	0.040918	2.933712	0.003937	0.999985
cg1153028	-0.09373	0.031948	-2.93369	0.003937	0.999985
cg1219470	-0.096	0.032727	-2.9334	0.00394	0.999985
cg0733515	-0.11006	0.037524	-2.93313	0.003944	0.999985
cg1605849	-0.20435	0.069678	-2.93284	0.003947	0.999985
cg2587281	-0.14313	0.048814	-2.93216	0.003955	0.999985
cg0662191	0.748368	0.255238	2.932043	0.003957	0.999985
cg2481105	0.077395	0.0264	2.931624	0.003962	0.999985
cg0960178	0.214792	0.073271	2.931486	0.003963	0.999985
cg0856015	0.128156	0.043721	2.931204	0.003967	0.999985
cg2756786	-0.1129	0.038519	-2.93111	0.003968	0.999985
cg1661123	0.097799	0.033368	2.930953	0.00397	0.999985
cg0470618	-0.08193	0.027957	-2.9307	0.003973	0.999985
cg0910318	0.094233	0.032155	2.930608	0.003974	0.999985
cg0460561	0.212287	0.072443	2.930411	0.003976	0.999985
cg1964030	-0.14337	0.048927	-2.93033	0.003977	0.999985
cg1549592	-0.10129	0.034567	-2.93011	0.00398	0.999985
cg0108242	-0.09406	0.032104	-2.92979	0.003984	0.999985
cg0103976	-0.12381	0.042261	-2.92965	0.003985	0.999985
cg2649966	0.111815	0.038169	2.929434	0.003988	0.999985
cg0183694	-0.17323	0.059136	-2.92928	0.00399	0.999985
cg0690260	0.116131	0.03965	2.928858	0.003995	0.999985
cg1005219	-0.23692	0.0809	-2.92854	0.003999	0.999985
cg0029585	0.10233	0.034943	2.9285	0.003999	0.999985
cg2139902	-0.11032	0.037672	-2.92838	0.004001	0.999985
cg1719427	-0.1277	0.043611	-2.92829	0.004002	0.999985
cg0627580	-0.09397	0.032091	-2.92815	0.004004	0.999985

cg0798690	-0.09065	0.030966	-2.92733	0.004013	0.999985
cg0882302	0.471313	0.161005	2.927316	0.004014	0.999985
cg1874346	-0.22457	0.076718	-2.92723	0.004015	0.999985
cg2254835	-0.09429	0.032212	-2.92711	0.004016	0.999985
cg1181970	-0.11522	0.039368	-2.92676	0.00402	0.999985
cg1227741	-0.17552	0.059977	-2.92645	0.004024	0.999985
cg1152883	0.20409	0.069747	2.92614	0.004028	0.999985
cg0997051	0.10322	0.035277	2.925999	0.00403	0.999985
cg0240560	-0.13058	0.04464	-2.92509	0.004041	0.999985
cg1654662	0.117676	0.040231	2.925023	0.004042	0.999985
cg0781349	0.050927	0.017414	2.924534	0.004047	0.999985
cg1050107	0.183264	0.062668	2.924356	0.00405	0.999985
cg2542855	0.155186	0.053079	2.923704	0.004058	0.999985
cg1291142	-0.14276	0.048834	-2.92347	0.00406	0.999985
cg0215782	0.404991	0.138538	2.923324	0.004062	0.999985
cg1070467	-0.12455	0.042609	-2.92309	0.004065	0.999985
cg1445173	-0.13326	0.045594	-2.92263	0.004071	0.999985
cg1329329	0.126852	0.043407	2.922363	0.004074	0.999985
cg0721099	0.098912	0.033852	2.921871	0.00408	0.999985
cg0066120	0.09792	0.033519	2.92135	0.004087	0.999985
cg2277879	-0.09122	0.031225	-2.92131	0.004087	0.999985
cg0214719	-0.13084	0.044795	-2.92096	0.004091	0.999985
cg1611082	0.173843	0.059528	2.920336	0.004099	0.999985
cg0754613	-0.14387	0.049268	-2.92016	0.004101	0.999985
cg1081029	-0.06536	0.022384	-2.9201	0.004102	0.999985
cg1081084	0.078684	0.026947	2.919936	0.004104	0.999985
cg1062747	-0.11419	0.039112	-2.91959	0.004108	0.999985
cg1719830	-0.10318	0.035342	-2.91951	0.004109	0.999985
cg1632877	-0.09669	0.033121	-2.91916	0.004114	0.999985
cg1370309	0.071593	0.024527	2.918972	0.004116	0.999985
cg2161096	0.09521	0.032619	2.918909	0.004117	0.999985
cg1783433	0.094331	0.032322	2.918513	0.004122	0.999985
cg1817871	0.140806	0.048249	2.918339	0.004124	0.999985
cg1133238	-0.09698	0.033238	-2.91787	0.00413	0.999985
cg1671675	0.122977	0.042148	2.91772	0.004132	0.999985
cg0182192	-0.07946	0.027233	-2.91763	0.004133	0.999985
cg1315553	0.122168	0.041875	2.917451	0.004135	0.999985
cg0840049	0.082219	0.028185	2.917107	0.004139	0.999985
cg1759657	0.13225	0.045337	2.917054	0.00414	0.999985
cg1939058	0.08355	0.028642	2.917046	0.00414	0.999985
cg1913268	-0.08298	0.028448	-2.91702	0.00414	0.999985
cg0475560	0.188003	0.064451	2.917005	0.00414	0.999985
cg1192012	-0.0798	0.027358	-2.9169	0.004142	0.999985
cg1357673	0.101408	0.034776	2.916039	0.004153	0.999985
cg2390822	0.384771	0.131959	2.915825	0.004155	0.999985
cg0033607	0.075194	0.025789	2.915708	0.004157	0.999985
cg1134253	0.097106	0.033305	2.915695	0.004157	0.999985
cg1608744	-0.18725	0.064222	-2.91564	0.004157	0.999985
cg2058612	0.17292	0.059308	2.915611	0.004158	0.999985
cg2409453	0.13501	0.046315	2.915047	0.004165	0.999985

cg1864993	-0.11562	0.039665	-2.91488	0.004167	0.999985
cg2654631	0.15828	0.054302	2.91482	0.004168	0.999985
cg1358202	-0.06415	0.02201	-2.9144	0.004173	0.999985
cg1475722	-0.16015	0.054952	-2.91426	0.004175	0.999985
cg2034319	-0.12873	0.044182	-2.91373	0.004181	0.999985
cg2080533	0.11966	0.041076	2.913113	0.004189	0.999985
cg0248968	0.127791	0.043868	2.913071	0.00419	0.999985
cg1379518	-0.08315	0.028547	-2.91274	0.004194	0.999985
cg0105208	-0.16247	0.055785	-2.91244	0.004198	0.999985
cg0068931	-0.13861	0.047596	-2.91216	0.004201	0.999985
cg1733260	0.06582	0.022604	2.911899	0.004205	0.999985
cg2147012	0.34478	0.118414	2.91165	0.004208	0.999985
cg1871439	0.102076	0.035059	2.911575	0.004209	0.999985
cg1448891	-0.80418	0.276218	-2.9114	0.004211	0.999985
cg0942533	-0.11528	0.039599	-2.91129	0.004212	0.999985
cg0988463	-0.08566	0.029422	-2.91127	0.004213	0.999985
cg1252908	0.120056	0.041243	2.910969	0.004216	0.999985
cg0256651	-0.09111	0.031317	-2.90927	0.004238	0.999985
cg1516129	0.104238	0.035831	2.909159	0.004239	0.999985
cg2064779	-0.15144	0.052059	-2.90907	0.004241	0.999985
cg0771776	-0.06756	0.023227	-2.90878	0.004244	0.999985
cg1669548	-0.0798	0.02744	-2.90828	0.004251	0.999985
cg0616773	-0.17035	0.058578	-2.90806	0.004253	0.999985
cg0866909	0.082989	0.028539	2.907897	0.004256	0.999985
cg0651878	-0.14563	0.050081	-2.90783	0.004256	0.999985
cg0596574	0.201972	0.069458	2.907815	0.004257	0.999985
cg0454160	-0.05496	0.018905	-2.90742	0.004262	0.999985
cg1357957	-0.16144	0.055529	-2.90721	0.004264	0.999985
cg1414132	-0.08472	0.029149	-2.90636	0.004275	0.999985
cg1735108	0.071676	0.024662	2.906333	0.004276	0.999985
cg1636481	0.113602	0.039088	2.906306	0.004276	0.999985
cg1451803	0.13028	0.04483	2.906087	0.004279	0.999985
cg1106429	-0.12095	0.041621	-2.9059	0.004281	0.999985
cg2012680	-0.11353	0.039075	-2.90554	0.004286	0.999985
cg1531267	-0.10132	0.034871	-2.90546	0.004287	0.999985
cg2666941	-0.08249	0.028397	-2.90495	0.004293	0.999985
cg1647879	-0.11785	0.040571	-2.90474	0.004296	0.999985
cg0567373	-0.105	0.036152	-2.90446	0.0043	0.999985
cg0875550	0.079599	0.027406	2.904414	0.0043	0.999985
cg2708378	-0.09502	0.032721	-2.90408	0.004305	0.999985
cg0942261	-0.13532	0.046597	-2.90401	0.004306	0.999985
cg0890792	-0.09121	0.031408	-2.904	0.004306	0.999985
cg0224856	0.082577	0.028437	2.903889	0.004307	0.999985
cg0928644	-0.08363	0.028802	-2.90379	0.004308	0.999985
cg1331422	0.072933	0.025118	2.903598	0.004311	0.999985
cg0491493	0.099075	0.034123	2.903482	0.004312	0.999985
cg1720548	-0.13568	0.046736	-2.90321	0.004316	0.999985
cg1375687	0.148208	0.051057	2.902817	0.004321	0.999985
cg1332139	-0.10603	0.036531	-2.90256	0.004324	0.999985
cg0428544	0.09824	0.033847	2.902429	0.004326	0.999985

cg1429868	-0.06524	0.022482	-2.90189	0.004333	0.999985
cg0332074	0.121426	0.041846	2.901763	0.004335	0.999985
cg0279478	-0.15923	0.054886	-2.90119	0.004342	0.999985
cg1435416	-0.20476	0.070582	-2.90109	0.004343	0.999985
cg0003785	-0.12129	0.04181	-2.90091	0.004346	0.999985
cg1770641	-0.10071	0.034716	-2.90083	0.004347	0.999985
cg2582392	0.096812	0.033376	2.900654	0.004349	0.999985
cg1339311	0.081207	0.027998	2.900457	0.004352	0.999985
cg1947965	-0.13956	0.048126	-2.89994	0.004358	0.999985
cg2010412	-0.07469	0.025755	-2.89989	0.004359	0.999985
cg2074680	0.090656	0.03127	2.899145	0.004369	0.999985
cg1792465	-0.08073	0.027847	-2.89897	0.004371	0.999985
cg0468448	-0.1035	0.035707	-2.89856	0.004376	0.999985
cg0687075	0.130176	0.044912	2.898469	0.004378	0.999985
cg1838963	0.103461	0.035695	2.898443	0.004378	0.999985
cg0356058	-0.08782	0.030301	-2.89809	0.004383	0.999985
cg1557096	-0.10913	0.037666	-2.89745	0.004391	0.999985
cg0205698	-0.08927	0.030823	-2.89635	0.004406	0.999985
cg1720427	-0.11359	0.039224	-2.89592	0.004411	0.999985
cg1746498	-0.11275	0.038949	-2.89496	0.004424	0.999985
cg0226048	-0.14341	0.049536	-2.89495	0.004424	0.999985
cg0950866	-0.14354	0.049586	-2.8947	0.004427	0.999985
cg0949909	-0.24571	0.084908	-2.89378	0.00444	0.999985
cg1412916	0.147212	0.05088	2.893335	0.004446	0.999985
cg1597066	0.236563	0.081762	2.893315	0.004446	0.999985
cg0782261	-0.08686	0.030023	-2.89328	0.004446	0.999985
cg1870271	-0.09274	0.032053	-2.89322	0.004447	0.999985
cg1825034	-0.15921	0.055035	-2.89296	0.004451	0.999985
cg0309613	-0.14769	0.051056	-2.89281	0.004453	0.999985
cg0770084	0.320849	0.110913	2.892803	0.004453	0.999985
cg1674962	-0.49791	0.172127	-2.89271	0.004454	0.999985
cg1860146	-0.08918	0.030834	-2.89218	0.004461	0.999985
cg0248632	-0.11709	0.040484	-2.89218	0.004461	0.999985
cg0121142	-0.09619	0.033261	-2.89191	0.004465	0.999985
cg2282265	-0.16949	0.058617	-2.89146	0.004471	0.999985
cg1267490	0.091645	0.031696	2.891388	0.004472	0.999985
cg0335172	-0.0978	0.033827	-2.89135	0.004472	0.999985
cg1105440	0.191028	0.066069	2.891346	0.004472	0.999985
cg1843703	0.112512	0.038916	2.891156	0.004475	0.999985
cg1355243	0.096424	0.033362	2.890251	0.004487	0.999985
cg1264889	0.09764	0.033784	2.890164	0.004488	0.999985
cg0115868	0.119419	0.041319	2.890134	0.004488	0.999985
cg1159254	0.098502	0.034087	2.889686	0.004494	0.999985
cg1458062	0.132632	0.045898	2.889684	0.004494	0.999985
cg1455945	0.133769	0.046297	2.889362	0.004499	0.999985
cg0808115	0.175135	0.060619	2.889112	0.004502	0.999985
cg0282971	-0.15523	0.053731	-2.88894	0.004505	0.999985
cg2384411	-0.08281	0.028665	-2.88875	0.004507	0.999985
cg0704076	0.093429	0.032344	2.888561	0.00451	0.999985
cg0584521	0.099393	0.03441	2.888487	0.004511	0.999985

cg0838755	-0.12603	0.043635	-2.88837	0.004512	0.999985
cg2102950	0.080417	0.027847	2.887777	0.00452	0.999985
cg2102411	0.112248	0.038871	2.887679	0.004521	0.999985
cg2513662	-0.09366	0.032439	-2.88717	0.004528	0.999985
cg0411803	-0.07425	0.025719	-2.88691	0.004532	0.999985
cg2728782	0.164748	0.05707	2.886773	0.004534	0.999985
cg0722364	0.073607	0.025502	2.886341	0.00454	0.999985
cg1892008	-0.07967	0.027608	-2.8859	0.004546	0.999985
cg0535417	-0.10032	0.034764	-2.88577	0.004547	0.999985
cg1412388	0.110512	0.038296	2.885757	0.004548	0.999985
cg0574660	-0.12592	0.043637	-2.88558	0.00455	0.999985
cg1236075	0.089712	0.031095	2.885067	0.004557	0.999985
cg2683122	0.070345	0.024384	2.88483	0.00456	0.999985
cg0013909	-0.09369	0.032478	-2.88465	0.004563	0.999985
cg2296228	-0.09066	0.031429	-2.88461	0.004563	0.999985
cg2368650	0.085595	0.029679	2.884056	0.004571	0.999985
cg0653292	0.10371	0.035961	2.883935	0.004572	0.999985
cg2710715	0.356503	0.123644	2.88331	0.004581	0.999985
cg0927607	-0.06369	0.022091	-2.88328	0.004581	0.999985
cg0614973	0.174141	0.060397	2.883268	0.004582	0.999985
cg2314437	0.149208	0.051755	2.882963	0.004586	0.999985
cg0897176	0.109702	0.038063	2.88213	0.004597	0.999985
cg1951047	-0.0744	0.025821	-2.88134	0.004608	0.999985
cg1401873	0.060546	0.021016	2.880993	0.004613	0.999985
cg0578229	0.137454	0.047711	2.880964	0.004613	0.999985
cg2633695	0.082245	0.028552	2.880536	0.004619	0.999985
cg0174624	-0.25869	0.089809	-2.88045	0.00462	0.999985
cg2139783	0.62421	0.216714	2.880335	0.004622	0.999985
cg1801748	-0.12604	0.043759	-2.88021	0.004624	0.999985
cg1519359	0.087478	0.030376	2.879846	0.004629	0.999985
cg1165781	-0.18392	0.06388	-2.87921	0.004637	0.999985
cg0811418	0.160802	0.055851	2.879155	0.004638	0.999985
cg0609086	0.090887	0.031568	2.879067	0.004639	0.999985
cg0171066	0.204025	0.070874	2.878701	0.004644	0.999985
cg0802271	-0.09416	0.03271	-2.8786	0.004646	0.999985
cg1197079	0.099824	0.034681	2.87834	0.004649	0.999985
cg2457326	0.208214	0.072345	2.878073	0.004653	0.999985
cg1468117	-0.10045	0.034904	-2.87782	0.004657	0.999985
cg2430925	-0.08616	0.029943	-2.87761	0.00466	0.999985
cg1994484	-0.1751	0.060859	-2.87717	0.004666	0.999985
cg0962377	-0.18824	0.065429	-2.87709	0.004667	0.999985
cg0130166	-0.10296	0.035791	-2.87683	0.00467	0.999985
cg2350473	-0.09311	0.032366	-2.87679	0.004671	0.999985
cg0248193	-0.12155	0.042256	-2.87662	0.004673	0.999985
cg0299679	-0.07849	0.027287	-2.87657	0.004674	0.999985
cg2415390	0.517453	0.1799	2.876342	0.004677	0.999985
cg1400677	0.120458	0.041889	2.875655	0.004687	0.999985
cg2124792	0.139093	0.048372	2.875467	0.004689	0.999985
cg2265494	-0.12858	0.044718	-2.87529	0.004692	0.999985
cg0881133	-0.119	0.041387	-2.87515	0.004694	0.999985

cg1773004	-0.09243	0.032154	-2.87469	0.0047	0.999985
cg1096379	0.109536	0.038107	2.874412	0.004704	0.999985
cg0559291	0.204771	0.071249	2.874017	0.00471	0.999985
cg0735954	-0.10105	0.035162	-2.87378	0.004713	0.999985
cg2413348	0.115366	0.040145	2.873762	0.004713	0.999985
cg2764429	-0.27673	0.096303	-2.87355	0.004716	0.999985
cg1516795	-0.06768	0.023553	-2.87334	0.004719	0.999985
cg1184811	-0.10646	0.037055	-2.87297	0.004724	0.999985
cg1141736	-0.07712	0.026845	-2.87261	0.00473	0.999985
cg0218814	0.432538	0.150586	2.872355	0.004733	0.999985
cg2508221	0.088955	0.030972	2.872121	0.004736	0.999985
cg2250058	0.128963	0.044905	2.871891	0.00474	0.999985
cg1247788	0.34343	0.119592	2.871674	0.004743	0.999985
cg2230122	-0.10502	0.03657	-2.87165	0.004743	0.999985
cg1492721	-0.09258	0.032241	-2.87161	0.004744	0.999985
cg2299953	-0.16879	0.058783	-2.87137	0.004747	0.999985
cg2545871	-0.06106	0.021267	-2.87093	0.004753	0.999985
cg2104892	0.131592	0.045838	2.870824	0.004755	0.999985
cg0903101	0.124219	0.04327	2.870808	0.004755	0.999985
cg0787450	-0.07663	0.026694	-2.87071	0.004756	0.999985
cg0506557	-0.11715	0.040813	-2.87037	0.004761	0.999985
cg0365233	0.116787	0.040691	2.870061	0.004766	0.999985
cg2470209	-0.09911	0.034533	-2.87004	0.004766	0.999985
cg2380821	-0.17567	0.061209	-2.87001	0.004766	0.999985
cg1114916	-0.13726	0.047827	-2.87	0.004766	0.999985
cg0516256	-0.05641	0.019655	-2.86979	0.004769	0.999985
cg0360161	0.111863	0.038985	2.86939	0.004775	0.999985
cg1007592	0.067751	0.023614	2.869102	0.004779	0.999985
cg2197352	0.102067	0.03558	2.86867	0.004785	0.999985
cg0770327	-0.09899	0.034507	-2.86864	0.004786	0.999985
cg2679148	-0.0657	0.022906	-2.86827	0.004791	0.999985
cg2183072	-0.12982	0.045262	-2.86812	0.004793	0.999985
cg1472676	-0.18185	0.063408	-2.86788	0.004797	0.999985
cg1535121	0.099438	0.034675	2.867688	0.004799	0.999985
cg2410531	-0.08021	0.027973	-2.86748	0.004802	0.999985
cg1028139	-0.09003	0.031399	-2.86717	0.004807	0.999985
cg1207422	0.17624	0.061468	2.867167	0.004807	0.999985
cg0870747	-0.05746	0.020045	-2.86672	0.004813	0.999985
cg1894452	0.101414	0.035378	2.866583	0.004815	0.999985
cg2270739	0.546741	0.190732	2.86655	0.004816	0.999985
cg1298254	-0.09108	0.031778	-2.86625	0.00482	0.999985
cg1516512	-0.13274	0.046319	-2.86576	0.004827	0.999985
cg1568448	-0.20009	0.069827	-2.86544	0.004832	0.999985
cg2146769	0.092276	0.032205	2.865321	0.004833	0.999985
cg1614900	0.087122	0.030412	2.864712	0.004842	0.999985
cg2132719	0.12023	0.04197	2.864651	0.004843	0.999985
cg0345883	0.132618	0.046299	2.864395	0.004847	0.999985
cg2064506	-0.05606	0.01957	-2.86433	0.004848	0.999985
cg1248716	-0.38208	0.133401	-2.86414	0.00485	0.999985
cg0885072	0.143931	0.050253	2.864141	0.00485	0.999985

cg0101368	0.086187	0.030095	2.863794	0.004855	0.999985
cg0292524	-0.08859	0.030934	-2.86377	0.004856	0.999985
cg2197120	-0.10013	0.034967	-2.86345	0.00486	0.999985
cg1406620	-0.09214	0.032182	-2.86296	0.004867	0.999985
cg2303856	0.110512	0.038601	2.862893	0.004868	0.999985
cg0991603	0.093256	0.032583	2.862115	0.00488	0.999985
cg1187223	0.080225	0.028035	2.861643	0.004886	0.999985
cg1767945	0.106218	0.037123	2.861204	0.004893	0.999985
cg0208680	-0.07659	0.026773	-2.86084	0.004898	0.999985
cg1694833	0.079029	0.027626	2.860612	0.004901	0.999985
cg0571838	-0.29728	0.103928	-2.86044	0.004904	0.999985
cg0114528	-0.13421	0.046919	-2.86042	0.004904	0.999985
cg0220231	0.099693	0.034853	2.860358	0.004905	0.999985
cg0659515	-0.20633	0.072148	-2.8598	0.004913	0.999985
cg2596602	0.130816	0.045744	2.859763	0.004914	0.999985
cg1551509	0.151611	0.053018	2.859588	0.004916	0.999985
cg2283958	-0.07632	0.026694	-2.85919	0.004922	0.999985
cg1042637	-0.09518	0.03329	-2.85918	0.004922	0.999985
cg1930150	0.310604	0.108635	2.85914	0.004923	0.999985
cg1067880	0.128979	0.045122	2.85844	0.004933	0.999985
cg0905218	0.441973	0.154631	2.858232	0.004936	0.999985
cg1654519	0.05472	0.019146	2.858048	0.004939	0.999985
cg1788958	-0.14353	0.05022	-2.85802	0.004939	0.999985
cg1102560	-0.11065	0.038717	-2.85799	0.00494	0.999985
cg0989053	-0.06625	0.023181	-2.8579	0.004941	0.999985
cg1429948	-0.30632	0.107209	-2.85721	0.004951	0.999985
cg2332211	-0.15245	0.053363	-2.85692	0.004955	0.999985
cg1695852	-0.21091	0.073831	-2.85663	0.00496	0.999985
cg1657169	0.108314	0.03792	2.856384	0.004963	0.999985
cg0315987	-0.07787	0.027261	-2.85631	0.004964	0.999985
cg2402796	-0.24554	0.085986	-2.85562	0.004975	0.999985
cg2307550	-0.18098	0.06338	-2.85543	0.004977	0.999985
cg2679533	0.343027	0.120138	2.855265	0.00498	0.999985
cg2100668	-0.18281	0.064028	-2.85521	0.004981	0.999985
cg1313035	-0.10375	0.036339	-2.85507	0.004983	0.999985
cg1607342	-0.07459	0.026128	-2.8548	0.004987	0.999985
cg0533722	-0.11694	0.040969	-2.85441	0.004993	0.999985
cg1628767	-0.11846	0.041512	-2.85373	0.005003	0.999985
cg2495207	-0.06965	0.024406	-2.85367	0.005004	0.999985
cg1354673	0.092498	0.032414	2.853644	0.005004	0.999985
cg1548137	-0.12007	0.042083	-2.85309	0.005012	0.999985
cg0001531	-0.07779	0.027267	-2.85291	0.005015	0.999985
cg2373202	0.239491	0.083953	2.85269	0.005018	0.999985
cg0769505	0.119859	0.04202	2.852449	0.005022	0.999985
cg1772989	0.242913	0.085172	2.852026	0.005028	0.999985
cg2442474	-0.07641	0.026793	-2.85183	0.005031	0.999985
cg2422801	-0.07527	0.026393	-2.85175	0.005032	0.999985
cg1471874	0.128707	0.045133	2.851707	0.005033	0.999985
cg1251732	-0.10522	0.036899	-2.85166	0.005033	0.999985
cg1363473	-0.11699	0.041026	-2.85159	0.005034	0.999985

cg0645514	-0.06886	0.024154	-2.85073	0.005047	0.999985
cg0085016	-0.09669	0.033919	-2.85058	0.00505	0.999985
cg1838312	0.119708	0.041997	2.850387	0.005052	0.999985
cg0596836	0.145771	0.051145	2.850148	0.005056	0.999985
cg2597952	0.103872	0.036449	2.849783	0.005061	0.999985
cg2486760	0.097147	0.034091	2.849613	0.005064	0.999985
cg1966072	-0.10372	0.036398	-2.84955	0.005065	0.999985
cg2217182	0.149861	0.052599	2.849143	0.005071	0.999985
cg1748101	0.364449	0.127923	2.84898	0.005074	0.999985
cg2500936	-0.14717	0.051663	-2.84868	0.005078	0.999985
cg0438412	-0.10037	0.035234	-2.84865	0.005078	0.999985
cg1763508	-0.1229	0.043148	-2.84835	0.005083	0.999985
cg0111769	-0.09543	0.033504	-2.84826	0.005084	0.999985
cg1984881	-0.10142	0.035609	-2.84801	0.005088	0.999985
cg1155298	0.150137	0.052729	2.847359	0.005098	0.999985
cg1366847	-0.09986	0.035073	-2.84717	0.005101	0.999985
cg1886791	-0.32773	0.115109	-2.84712	0.005102	0.999985
cg1990796	-0.09047	0.031778	-2.84704	0.005103	0.999985
cg2594638	-0.18529	0.065084	-2.84685	0.005106	0.999985
cg2537916	0.102785	0.036107	2.846685	0.005108	0.999985
cg1754890	-0.04106	0.014426	-2.84656	0.00511	0.999985
cg0926711	0.129722	0.045573	2.846483	0.005111	0.999985
cg2094228	0.444568	0.156196	2.846212	0.005115	0.999985
cg0733273	0.117062	0.041146	2.845056	0.005133	0.999985
cg1578139	0.164043	0.057672	2.844394	0.005143	0.999985
cg2306064	0.11105	0.039044	2.844227	0.005145	0.999985
cg0695452	0.116763	0.041054	2.844131	0.005147	0.999985
cg1555188	0.121226	0.042624	2.844082	0.005148	0.999985
cg0311374	0.06817	0.023972	2.843742	0.005153	0.999985
cg0091760	-0.13929	0.048987	-2.8434	0.005158	0.999985
cg0755935	-0.12769	0.044912	-2.84321	0.005161	0.999985
cg1494826	0.076775	0.027004	2.84307	0.005163	0.999985
cg1700397	0.345952	0.121686	2.843	0.005164	0.999985
cg1624139	0.131122	0.046123	2.842909	0.005166	0.999985
cg2254422	0.135029	0.047511	2.842053	0.005179	0.999985
cg2671655	-0.084	0.029557	-2.84203	0.005179	0.999985
cg0649467	-0.12179	0.042861	-2.84157	0.005186	0.999985
cg0940935	-0.07253	0.025525	-2.84154	0.005186	0.999985
cg2574064	0.138746	0.048828	2.841532	0.005187	0.999985
cg2119821	0.117556	0.041374	2.841298	0.00519	0.999985
cg2270296	-0.09261	0.032595	-2.84126	0.005191	0.999985
cg1672200	0.181004	0.063737	2.839872	0.005212	0.999985
cg0787628	-0.1434	0.050501	-2.83955	0.005217	0.999985
cg1161939	0.089251	0.031432	2.839533	0.005217	0.999985
cg1432853	0.073282	0.025813	2.838989	0.005226	0.999985
cg1486280	-0.06267	0.022078	-2.83875	0.005229	0.999985
cg1216384	-0.08279	0.029167	-2.83839	0.005235	0.999985
cg1501104	-0.09073	0.031968	-2.83807	0.00524	0.999985
cg2582107	-0.11474	0.040431	-2.838	0.005241	0.999985
cg0913520	0.104125	0.03669	2.837952	0.005242	0.999985

cg0118857	-1.31792	0.464414	-2.83781	0.005244	0.999985
cg2073707	0.183718	0.064746	2.837539	0.005248	0.999985
cg1381064	-0.07877	0.027762	-2.83749	0.005249	0.999985
cg2661793	0.196345	0.069213	2.836842	0.005259	0.999985
cg2042627	0.359782	0.12683	2.836732	0.005261	0.999985
cg0873724	0.118041	0.041633	2.835274	0.005283	0.999985
cg0366145	-0.11518	0.040626	-2.83518	0.005285	0.999985
cg0266478	-0.08711	0.030728	-2.83497	0.005288	0.999985
cg0618686	-0.13576	0.047887	-2.83493	0.005289	0.999985
cg0950323	-0.16006	0.056461	-2.83484	0.00529	0.999985
cg2024654	0.299342	0.105606	2.834509	0.005295	0.999985
cg0745965	-0.07324	0.025846	-2.8337	0.005308	0.999985
cg0861203	-0.09955	0.035137	-2.83318	0.005316	0.999985
cg0596295	-0.33388	0.117873	-2.83257	0.005326	0.999985
cg2672902	-0.0851	0.030045	-2.83254	0.005326	0.999985
cg0110459	-0.19683	0.06949	-2.8325	0.005327	0.999985
cg2036897	-0.11099	0.039184	-2.83245	0.005328	0.999985
cg2071323	-0.13758	0.048578	-2.83223	0.005331	0.999985
cg0806426	0.119717	0.042274	2.831952	0.005335	0.999985
cg2022421	-0.05661	0.019994	-2.8313	0.005346	0.999985
cg0167341	-0.1193	0.042138	-2.83118	0.005348	0.999985
cg0022690	-0.12008	0.042419	-2.83085	0.005353	0.999985
cg1799456	0.139014	0.049108	2.830807	0.005353	0.999985
cg2640558	-0.12861	0.04544	-2.83042	0.00536	0.999985
cg0330636	0.104943	0.037077	2.83036	0.00536	0.999985
cg1947261	0.087556	0.030936	2.830203	0.005363	0.999985
cg2553090	-0.11818	0.04176	-2.83008	0.005365	0.999985
cg0857607	0.098963	0.034972	2.829765	0.00537	0.999985
cg1941965	-0.21558	0.076183	-2.82972	0.005371	0.999985
cg2471544	0.157813	0.055773	2.829528	0.005374	0.999985
cg0362994	-0.08761	0.030963	-2.82942	0.005375	0.999985
cg2432978	0.18041	0.063762	2.829416	0.005375	0.999985
cg0263918	0.246718	0.087199	2.829369	0.005376	0.999985
cg2264837	0.095622	0.0338	2.829051	0.005381	0.999985
cg1879516	-0.66519	0.235129	-2.82905	0.005381	0.999985
cg2375825	0.073864	0.02611	2.828947	0.005383	0.999985
cg0843027	0.653307	0.230953	2.828737	0.005386	0.999985
cg0863388	-0.18781	0.066393	-2.82873	0.005386	0.999985
cg1969449	-0.11025	0.038977	-2.82869	0.005387	0.999985
cg0136951	0.24432	0.086378	2.828499	0.00539	0.999985
cg0823956	0.124953	0.044177	2.828437	0.005391	0.999985
cg2179422	-0.05963	0.021082	-2.82832	0.005393	0.999985
cg2245883	0.090909	0.032144	2.82816	0.005395	0.999985
cg1578222	0.318468	0.112615	2.827924	0.005399	0.999985
cg2392382	-0.08954	0.031662	-2.82789	0.0054	0.999985
cg2126904	0.153353	0.054233	2.827688	0.005403	0.999985
cg1729573	-0.11892	0.04206	-2.8275	0.005406	0.999985
cg0438370	0.175403	0.062035	2.827488	0.005406	0.999985
cg1443398	0.185243	0.065532	2.826741	0.005418	0.999985
cg1863055	-0.06	0.021226	-2.82673	0.005418	0.999985

cg1561268	-0.08238	0.029146	-2.82658	0.00542	0.999985
cg0474822	0.097855	0.034621	2.826461	0.005422	0.999985
cg0351248	0.109879	0.038876	2.826399	0.005423	0.999985
cg1135046	-0.09589	0.033928	-2.8262	0.005427	0.999985
cg2697427	0.118458	0.041916	2.826118	0.005428	0.999985
cg0042348	-0.08609	0.030463	-2.82609	0.005428	0.999985
cg0683631	0.414446	0.146653	2.826028	0.005429	0.999985
cg1843012	0.13947	0.049354	2.825897	0.005431	0.999985
cg1456271	-0.13965	0.049417	-2.82584	0.005432	0.999985
cg1465252	0.401061	0.141934	2.825692	0.005435	0.999985
cg0729019	-0.08483	0.030021	-2.82566	0.005435	0.999985
cg1594578	0.113962	0.040331	2.825634	0.005436	0.999985
cg0779146	0.076122	0.026942	2.825381	0.00544	0.999985
cg0250632	0.053712	0.019012	2.825211	0.005442	0.999985
cg2039045	0.106654	0.037752	2.825097	0.005444	0.999985
cg1097898	0.147748	0.052303	2.824863	0.005448	0.999985
cg0112873	-0.09351	0.033109	-2.82445	0.005455	0.999985
cg2264432	0.11982	0.042424	2.824361	0.005456	0.999985
cg0015152	-0.08211	0.029077	-2.8239	0.005464	0.999985
cg2761558	0.14417	0.051055	2.823839	0.005464	0.999985
cg2433008	-0.08254	0.029232	-2.8237	0.005467	0.999985
cg0932073	-0.13901	0.049235	-2.82334	0.005472	0.999985
cg0600829	-0.10606	0.037567	-2.8232	0.005475	0.999985
cg1565120	-0.16561	0.05866	-2.82313	0.005476	0.999985
cg2588687	0.084391	0.029895	2.822921	0.005479	0.999985
cg1280992	-0.12326	0.043666	-2.82274	0.005482	0.999985
cg2350596	-0.21245	0.075272	-2.82236	0.005488	0.999985
cg0750615	-0.88241	0.312651	-2.82233	0.005489	0.999985
cg2107279	-0.0535	0.018958	-2.82214	0.005492	0.999985
cg2392712	-0.06758	0.023946	-2.82213	0.005492	0.999985
cg1282795	-0.10643	0.037722	-2.82131	0.005505	0.999985
cg0600045	-0.0959	0.03399	-2.8213	0.005505	0.999985
cg0071072	-0.13956	0.049471	-2.82112	0.005508	0.999985
cg0045990	0.286005	0.101381	2.821086	0.005509	0.999985
cg1407165	-0.14349	0.050863	-2.82101	0.00551	0.999985
cg1699343	-0.10389	0.036828	-2.8209	0.005512	0.999985
cg2115723	0.210945	0.074784	2.820736	0.005515	0.999985
cg2521492	0.087992	0.031195	2.820721	0.005515	0.999985
cg2127951	0.131587	0.046651	2.820658	0.005516	0.999985
cg0567803	-0.15091	0.053511	-2.82011	0.005525	0.999985
cg0682038	0.109354	0.038777	2.820041	0.005526	0.999985
cg1031524	-0.05686	0.020167	-2.81942	0.005536	0.999985
cg0844012	-0.06442	0.022855	-2.81882	0.005546	0.999985
cg2655120	0.038939	0.013814	2.818801	0.005546	0.999985
cg0390019	-0.1671	0.059289	-2.81845	0.005552	0.999985
cg1516849	0.36785	0.130519	2.818361	0.005553	0.999985
cg0095924	-0.1445	0.051274	-2.8182	0.005556	0.999985
cg1027787	0.44749	0.158798	2.817992	0.005559	0.999985
cg0435190	0.076657	0.027203	2.817922	0.00556	0.999985
cg1709348	-0.20344	0.072199	-2.81783	0.005562	0.999985

cg0386421	0.064854	0.023018	2.817494	0.005567	0.999985
cg1292590	0.221828	0.078733	2.817455	0.005568	0.999985
cg1295426	0.165098	0.058604	2.81716	0.005573	0.999985
cg1157853	0.281655	0.099994	2.816711	0.00558	0.999985
cg2070662	-0.17525	0.062252	-2.81514	0.005606	0.999985
cg2709608	0.075915	0.026967	2.815135	0.005606	0.999985
cg1094657	0.128573	0.045681	2.814583	0.005615	0.999985
cg1302785	-0.08197	0.029125	-2.81446	0.005617	0.999985
cg0086999	-0.13035	0.04633	-2.81342	0.005634	0.999985
cg0734002	0.122865	0.043676	2.81307	0.00564	0.999985
cg0680967	-0.17987	0.063944	-2.81292	0.005643	0.999985
cg2316200	-0.09973	0.035457	-2.8126	0.005648	0.999985
cg0172906	-0.07179	0.025524	-2.81245	0.00565	0.999985
cg1750830	-0.34804	0.123752	-2.81237	0.005652	0.999985
cg1189524	0.069332	0.024653	2.812304	0.005653	0.999985
cg1465022	-0.29212	0.103874	-2.81228	0.005653	0.999985
cg1152196	-0.05114	0.018184	-2.81228	0.005653	0.999985
cg1086307	-0.08906	0.031675	-2.81174	0.005662	0.999985
cg0842474	-0.0777	0.027639	-2.81131	0.005669	0.999985
cg1297594	0.080551	0.028654	2.811193	0.005671	0.999985
cg1083577	0.129377	0.046023	2.811129	0.005672	0.999985
cg2168863	0.128165	0.045595	2.810953	0.005675	0.999985
cg0118443	0.112166	0.039912	2.810325	0.005686	0.999985
cg2722334	-0.10675	0.037987	-2.81021	0.005688	0.999985
cg0739270	-0.10317	0.036714	-2.80996	0.005692	0.999985
cg1632041	-0.11926	0.042449	-2.80955	0.005699	0.999985
cg2430442	0.093281	0.033212	2.808659	0.005714	0.999985
cg1821170	-0.10946	0.038977	-2.80824	0.005721	0.999985
cg2477224	-0.09058	0.032257	-2.80797	0.005725	0.999985
cg1520554	0.094537	0.033674	2.807421	0.005734	0.999985
cg0347084	-0.1122	0.039967	-2.80739	0.005735	0.999985
cg2284107	-0.08892	0.031675	-2.80729	0.005737	0.999985
cg0314028	0.127749	0.045507	2.807272	0.005737	0.999985
cg1708790	-0.0922	0.032847	-2.80684	0.005744	0.999985
cg1986283	0.468326	0.166859	2.80672	0.005746	0.999985
cg1460109	-0.15892	0.056634	-2.80601	0.005758	0.999985
cg2333648	-0.3645	0.129901	-2.806	0.005758	0.999985
cg1390131	-0.15446	0.055051	-2.80572	0.005763	0.999985
cg0178997	-0.08379	0.029869	-2.80533	0.00577	0.999985
cg1072175	0.088776	0.031647	2.805221	0.005771	0.999985
cg1019100	0.174817	0.062318	2.805213	0.005772	0.999985
cg2202325	0.133498	0.047594	2.804939	0.005776	0.999985
cg0211596	-0.06607	0.023559	-2.80461	0.005782	0.999985
cg1617692	0.116924	0.041693	2.804424	0.005785	0.999985
cg2707974	0.149557	0.053331	2.80431	0.005787	0.999985
cg0803666	0.068535	0.02444	2.80419	0.005789	0.999985
cg1394409	-0.10169	0.036265	-2.80408	0.005791	0.999985
cg1692910	-0.08101	0.028893	-2.80389	0.005794	0.999985
cg0187801	-0.07471	0.026645	-2.80372	0.005797	0.999985
cg2678441	-0.14057	0.050144	-2.80342	0.005802	0.999985

cg1014827	-0.12333	0.043998	-2.80303	0.005809	0.999985
cg0451301	0.192765	0.068775	2.802826	0.005812	0.999985
cg0074473	-0.08655	0.030885	-2.80246	0.005818	0.999985
cg0724192	0.082023	0.029272	2.802099	0.005824	0.999985
cg2698117	0.078514	0.028021	2.802006	0.005826	0.999985
cg1065205	-0.08724	0.031136	-2.8018	0.00583	0.999985
cg2233921	0.132611	0.047337	2.801431	0.005836	0.999985
cg1996082	0.083363	0.029758	2.801351	0.005837	0.999985
cg1391390	0.133514	0.047668	2.800922	0.005845	0.999985
cg2229350	-0.11616	0.041474	-2.80073	0.005848	0.999985
cg0067698	-0.10038	0.035841	-2.80064	0.005849	0.999985
cg1758480	0.12152	0.043391	2.800559	0.005851	0.999985
cg1610114	-0.10923	0.039009	-2.80019	0.005857	0.999985
cg0327019	-0.09561	0.034146	-2.80015	0.005858	0.999985
cg0280107	-0.14291	0.051041	-2.79992	0.005862	0.999985
cg2356197	0.17903	0.063949	2.799578	0.005868	0.999985
cg0494366	-0.11911	0.042549	-2.7994	0.005871	0.999985
cg0659694	-0.1113	0.03976	-2.79932	0.005872	0.999985
cg1596554	0.159963	0.057152	2.798888	0.005879	0.999985
cg1952183	0.268215	0.095831	2.798832	0.00588	0.999985
cg1599451	-0.0755	0.026979	-2.79846	0.005887	0.999985
cg0390272	-0.09876	0.035291	-2.79838	0.005888	0.999985
cg0514818	-0.10677	0.038162	-2.79791	0.005896	0.999985
cg0699046	-0.06546	0.023399	-2.79744	0.005904	0.999985
cg2051620	0.12751	0.045582	2.797389	0.005905	0.999985
cg0313167	0.077213	0.027604	2.797163	0.005909	0.999985
cg2650039	0.106015	0.037902	2.79709	0.00591	0.999985
cg0500411	0.15689	0.056098	2.796707	0.005917	0.999985
cg2687248	-0.12236	0.043752	-2.79668	0.005917	0.999985
cg0782161	-0.07967	0.028494	-2.79618	0.005926	0.999985
cg1477259	0.083532	0.029876	2.795965	0.00593	0.999985
cg1492189	-0.12851	0.045968	-2.79565	0.005935	0.999985
cg0671276	-0.59744	0.213719	-2.79543	0.005939	0.999985
cg2749416	-0.11734	0.041979	-2.79515	0.005944	0.999985
cg0803392	-0.11114	0.039763	-2.79491	0.005948	0.999985
cg0994833	-0.20498	0.073348	-2.79466	0.005952	0.999985
cg2522515	-0.15632	0.055941	-2.79445	0.005956	0.999985
cg2275544	-0.07409	0.026513	-2.79434	0.005958	0.999985
cg2256390	0.066276	0.023724	2.793634	0.00597	0.999985
cg0425391	0.097113	0.03477	2.793019	0.005981	0.999985
cg1230411	-0.13216	0.047319	-2.79299	0.005982	0.999985
cg1909177	0.091629	0.032813	2.79245	0.005991	0.999985
cg1968547	-0.10589	0.037923	-2.79225	0.005995	0.999985
cg0522384	-0.09488	0.033979	-2.79223	0.005995	0.999985
cg1094069	0.101083	0.036204	2.792017	0.005999	0.999985
cg1627548	-0.08973	0.032141	-2.79167	0.006005	0.999985
cg1416871	0.139915	0.05012	2.791568	0.006007	0.999985
cg1301984	0.132383	0.047423	2.791521	0.006007	0.999985
cg2189767	-0.18554	0.066492	-2.79047	0.006026	0.999985
cg1451580	-0.14693	0.052659	-2.79023	0.00603	0.999985

cg0717827	0.188577	0.067591	2.789991	0.006034	0.999985
cg1372299	-0.1049	0.0376	-2.78992	0.006035	0.999985
cg1104426	-0.07553	0.027073	-2.78969	0.006039	0.999985
cg2691599	-0.12062	0.043241	-2.78955	0.006042	0.999985
cg2597337	-0.08611	0.030869	-2.78951	0.006043	0.999985
cg2731083	0.097056	0.034797	2.789221	0.006048	0.999985
cg1728200	0.092163	0.033043	2.789179	0.006049	0.999985
cg0665537	-0.07973	0.028588	-2.78899	0.006052	0.999985
cg0687484	-0.06599	0.023663	-2.78863	0.006058	0.999985
cg0086654	0.10064	0.036092	2.78844	0.006062	0.999985
cg0092941	0.137931	0.049466	2.788421	0.006062	0.999985
cg1543650	0.10256	0.036781	2.788411	0.006062	0.999985
cg0033257	-0.07894	0.028309	-2.78832	0.006064	0.999985
cg2385629	-0.12185	0.043703	-2.78822	0.006065	0.999985
cg1565311	0.149558	0.053644	2.787961	0.00607	0.999985
cg2712788	-0.10656	0.038224	-2.78787	0.006072	0.999985
cg1258168	0.120598	0.043262	2.787607	0.006076	0.999985
cg0255109	-0.15997	0.057387	-2.78758	0.006077	0.999985
cg1276029	0.216196	0.077565	2.787279	0.006082	0.999985
cg0347839	-0.17817	0.063928	-2.78699	0.006087	0.999985
cg2665130	0.264797	0.095026	2.78658	0.006095	0.999985
cg0076788	-0.10283	0.036901	-2.78656	0.006095	0.999985
cg1487421	0.55336	0.198608	2.786197	0.006101	0.999985
cg2553229	-0.20614	0.07399	-2.78603	0.006104	0.999985
cg0894615	0.110772	0.039761	2.785958	0.006106	0.999985
cg2032125	-0.07984	0.028659	-2.78587	0.006107	0.999985
cg1456527	0.291388	0.104596	2.785839	0.006108	0.999985
cg1470629	0.148399	0.053271	2.785735	0.00611	0.999985
cg2067307	0.090063	0.032333	2.785488	0.006114	0.999985
cg2137404	-0.06059	0.021753	-2.78532	0.006117	0.999985
cg1055086	0.150144	0.053908	2.785206	0.006119	0.999985
cg0547882	-0.14336	0.05148	-2.7848	0.006126	0.999985
cg1151177	-0.06948	0.024952	-2.78462	0.006129	0.999985
cg2722866	0.129409	0.046475	2.784472	0.006132	0.999985
cg0388258	-0.10006	0.035942	-2.78395	0.006141	0.999985
cg1045010	-0.07306	0.026245	-2.78391	0.006142	0.999985
cg2322691	0.224292	0.080571	2.783788	0.006144	0.999985
cg1349503	-0.13659	0.049067	-2.78368	0.006146	0.999985
cg2714212	0.109744	0.039431	2.783167	0.006155	0.999985
cg0661617	0.145881	0.052418	2.783047	0.006158	0.999985
cg1886175	-0.1571	0.056453	-2.78291	0.00616	0.999985
cg2479918	0.12787	0.045952	2.782655	0.006165	0.999985
cg0740874	-0.15548	0.055888	-2.78196	0.006177	0.999985
cg0036990	0.115072	0.041369	2.78158	0.006184	0.999985
cg0621387	-0.10144	0.03647	-2.78158	0.006184	0.999985
cg0305612	0.148475	0.05338	2.781457	0.006186	0.999985
cg2146449	0.223292	0.080292	2.781004	0.006194	0.999985
cg2595117	0.499878	0.179757	2.78086	0.006197	0.999985
cg2097115	0.13097	0.047111	2.780018	0.006212	0.999985
cg1093440	0.178204	0.064105	2.779896	0.006214	0.999985

cg0684024	0.122118	0.043933	2.779674	0.006218	0.999985
cg0924834	0.125342	0.045097	2.77941	0.006223	0.999985
cg1474095	-0.18057	0.064981	-2.77884	0.006233	0.999985
cg0578345	-0.07839	0.028216	-2.77831	0.006243	0.999985
cg0640915	0.129739	0.046698	2.778232	0.006244	0.999985
cg1307177	-0.11382	0.04097	-2.77812	0.006246	0.999985
cg0046402	0.139461	0.0502	2.778085	0.006247	0.999985
cg0879930	-0.08244	0.029677	-2.77808	0.006247	0.999985
cg1432986	-0.0824	0.029665	-2.77783	0.006252	0.999985
cg2038769	-0.15769	0.056777	-2.77739	0.00626	0.999985
cg0997684	0.126967	0.045715	2.77738	0.00626	0.999985
cg0378303	-0.13023	0.046897	-2.77696	0.006268	0.999985
cg1670938	-0.06569	0.023656	-2.77672	0.006272	0.999985
cg2647428	-0.08049	0.028988	-2.77655	0.006275	0.999985
cg1291149	0.136679	0.049228	2.776462	0.006277	0.999985
cg1827253	-0.11111	0.040024	-2.77621	0.006281	0.999985
cg0341788	-0.19485	0.070196	-2.77576	0.006289	0.999985
cg1452833	-0.10984	0.03957	-2.77573	0.00629	0.999985
cg2057449	-0.07134	0.025704	-2.77558	0.006293	0.999985
cg2471899	0.114341	0.041201	2.775223	0.006299	0.999985
cg1047534	-0.07196	0.025931	-2.77503	0.006303	0.999985
cg2217786	-0.15253	0.054973	-2.7746	0.006311	0.999985
cg2241907	0.059684	0.021512	2.774448	0.006314	0.999985
cg0461725	-0.19429	0.070031	-2.77439	0.006315	0.999985
cg1447930	0.138986	0.050096	2.774376	0.006315	0.999985
cg1393483	-0.17053	0.061469	-2.77434	0.006316	0.999985
cg1387551	0.07218	0.026019	2.774144	0.006319	0.999985
cg2610310	-0.16628	0.059943	-2.774	0.006322	0.999985
cg1138347	-0.14109	0.050865	-2.77378	0.006326	0.999985
cg2280883	0.240933	0.086867	2.773578	0.00633	0.999985
cg1791982	-0.08763	0.031594	-2.77355	0.00633	0.999985
cg2758218	0.106119	0.038263	2.773433	0.006332	0.999985
cg2146443	-0.13237	0.047729	-2.77342	0.006332	0.999985
cg0960684	0.187448	0.067588	2.773382	0.006333	0.999985
cg1734732	-0.06616	0.023855	-2.77334	0.006334	0.999985
cg0006167	-0.10362	0.037367	-2.77307	0.006339	0.999985
cg1487094	0.108114	0.038992	2.772699	0.006346	0.999985
cg0819712	-0.08705	0.031395	-2.77261	0.006347	0.999985
cg0024203	-0.09722	0.035068	-2.77247	0.00635	0.999985
cg1977937	0.133662	0.048222	2.771802	0.006362	0.999985
cg2293890	-0.09425	0.034004	-2.77171	0.006364	0.999985
cg1369365	-0.3233	0.116648	-2.77158	0.006366	0.999985
cg2102279	-0.09797	0.035351	-2.7712	0.006373	0.999985
cg1794058	0.084087	0.030349	2.770687	0.006383	0.999985
cg2082104	0.261794	0.094494	2.770486	0.006387	0.999985
cg2162514	0.167524	0.060473	2.770252	0.006391	0.999985
cg0267847	-0.11462	0.041377	-2.77025	0.006391	0.999985
cg2660065	0.064536	0.023296	2.770235	0.006391	0.999985
cg0317672	0.19225	0.069405	2.769994	0.006396	0.999985
cg1057428	-0.08048	0.029056	-2.76998	0.006396	0.999985

cg2229511	-0.09018	0.032559	-2.76985	0.006398	0.999985
cg0602306	-0.15967	0.05765	-2.76971	0.006401	0.999985
cg1503326	0.059881	0.021623	2.769295	0.006409	0.999985
cg0527641	0.079289	0.028634	2.769069	0.006413	0.999985
cg2428764	0.093785	0.03387	2.768997	0.006414	0.999985
cg2083903	-0.07741	0.027961	-2.76849	0.006424	0.999985
cg1828090	0.668077	0.24132	2.768427	0.006425	0.999985
cg2334610	-0.08109	0.029292	-2.76841	0.006425	0.999985
cg0059279	0.061084	0.022066	2.768297	0.006427	0.999985
cg1254249	-0.10174	0.036758	-2.76792	0.006434	0.999985
cg0088001	-0.08041	0.029053	-2.76782	0.006436	0.999985
cg1155566	0.200461	0.072432	2.767573	0.006441	0.999985
cg2099512	0.068066	0.024596	2.767343	0.006445	0.999985
cg2106629	0.112763	0.040748	2.767337	0.006445	0.999985
cg0346693	0.141844	0.051266	2.766825	0.006455	0.999985
cg2462254	-0.17155	0.062007	-2.76657	0.00646	0.999985
cg0934421	0.133386	0.048214	2.766552	0.00646	0.999985
cg1793561	-0.09235	0.033391	-2.76563	0.006477	0.999985
cg0117555	-0.07744	0.028007	-2.76524	0.006484	0.999985
cg0485150	0.067963	0.024578	2.765142	0.006486	0.999985
cg0573099	0.106592	0.03855	2.765048	0.006488	0.999985
cg2306380	-0.09565	0.034595	-2.76493	0.00649	0.999985
cg0006048	0.138269	0.050008	2.764913	0.006491	0.999985
cg1611223	-0.0921	0.033314	-2.76461	0.006496	0.999985
cg0424484	0.089672	0.032437	2.764546	0.006498	0.999985
cg0101408	0.09232	0.033399	2.764142	0.006505	0.999985
cg2156203	0.083804	0.030319	2.764088	0.006506	0.999985
cg1501900	0.697641	0.252398	2.764051	0.006507	0.999985
cg0850140	0.133527	0.04831	2.763992	0.006508	0.999985
cg0353903	0.224221	0.081122	2.763989	0.006508	0.999985
cg1378323	-0.11556	0.041808	-2.76396	0.006509	0.999985
cg0376542	0.154684	0.055971	2.763658	0.006514	0.999985
cg1205486	-0.06438	0.023298	-2.76355	0.006516	0.999985
cg1936647	0.105394	0.038139	2.763391	0.006519	0.999985
cg2421963	-0.07461	0.026999	-2.76333	0.00652	0.999985
cg0681666	-0.12055	0.043625	-2.76322	0.006523	0.999985
cg0703840	0.105939	0.03834	2.763122	0.006524	0.999985
cg2135007	-0.18378	0.066525	-2.76255	0.006535	0.999985
cg0170654	0.14951	0.054121	2.762504	0.006536	0.999985
cg0207179	0.082422	0.029837	2.762397	0.006538	0.999985
cg1636702	-0.09341	0.033814	-2.76231	0.00654	0.999985
cg1844510	0.035013	0.012676	2.762169	0.006542	0.999985
cg0067589	-0.13261	0.048014	-2.76182	0.006549	0.999985
cg0548720	0.120966	0.0438	2.761813	0.006549	0.999985
cg0503199	-0.13301	0.048161	-2.76172	0.006551	0.999985
cg1412673	-0.09305	0.0337	-2.76129	0.006559	0.999985
cg1427150	0.078484	0.028424	2.761169	0.006561	0.999985
cg0911050	-0.0705	0.025532	-2.76113	0.006562	0.999985
cg2077849	-0.05429	0.019663	-2.76113	0.006562	0.999985
cg1327812	-0.09095	0.032942	-2.76092	0.006566	0.999985

cg2713622	0.083747	0.030336	2.760652	0.006571	0.999985
cg1362031	-0.17922	0.064923	-2.76048	0.006574	0.999985
cg2063241	-0.10346	0.037483	-2.76006	0.006583	0.999985
cg0791556	-0.0977	0.035397	-2.76	0.006584	0.999985
cg0065317	0.171167	0.062021	2.759843	0.006587	0.999985
cg0253399	0.16695	0.060497	2.759641	0.006591	0.999985
cg0340078	-0.22629	0.082	-2.75962	0.006591	0.999985
cg2698513	0.096323	0.034905	2.759593	0.006591	0.999985
cg0441145	-0.09836	0.035644	-2.75944	0.006594	0.999985
cg2162816	-0.10314	0.037385	-2.759	0.006603	0.999985
cg1757266	-0.08771	0.03179	-2.75895	0.006604	0.999985
cg2642414	-0.12898	0.046749	-2.75893	0.006604	0.999985
cg2363760	-0.12551	0.045502	-2.75831	0.006616	0.999985
cg1679317	0.15869	0.057538	2.758018	0.006622	0.999985
cg1710346	0.115706	0.041954	2.75792	0.006623	0.999985
cg0113747	-0.18439	0.066861	-2.75784	0.006625	0.999985
cg0437144	-0.09589	0.034772	-2.75776	0.006627	0.999985
cg2539097	-0.0905	0.032817	-2.75772	0.006627	0.999985
cg1700848	0.1869	0.067776	2.757588	0.00663	0.999985
cg1002583	-0.0434	0.015738	-2.75749	0.006632	0.999985
cg1180668	-0.09934	0.036028	-2.75731	0.006635	0.999985
cg1025260	-0.07745	0.028091	-2.75703	0.006641	0.999985
cg0311251	0.269719	0.097836	2.756855	0.006644	0.999985
cg2246355	-0.14815	0.053742	-2.7567	0.006647	0.999985
cg1664086	-0.17041	0.061818	-2.75661	0.006649	0.999985
cg1194163	-0.22571	0.081883	-2.75648	0.006651	0.999985
cg2492228	-0.07552	0.027399	-2.75644	0.006652	0.999985
cg2005171	0.084415	0.030625	2.756379	0.006653	0.999985
cg1525224	0.087341	0.031688	2.75631	0.006654	0.999985
cg2694683	-0.07334	0.02661	-2.75601	0.00666	0.999985
cg0945588	-0.10597	0.038458	-2.75556	0.006669	0.999985
cg0508681	-0.10734	0.038954	-2.75543	0.006671	0.999985
cg2648854	-0.12065	0.043789	-2.75522	0.006675	0.999985
cg1232985	0.274843	0.099779	2.754527	0.006689	0.999985
cg0020249	0.106234	0.038571	2.754267	0.006694	0.999985
cg0639482	-1.4148	0.513702	-2.75412	0.006697	0.999985
cg0837213	-0.14204	0.051575	-2.75403	0.006698	0.999985
cg1599120	-0.11073	0.040208	-2.75384	0.006702	0.999985
cg0771926	-0.07464	0.027105	-2.75375	0.006704	0.999985
cg0646602	-0.12056	0.043783	-2.75356	0.006707	0.999985
cg2435185	0.076025	0.027611	2.753406	0.00671	0.999985
cg0834910	0.256964	0.093334	2.753176	0.006715	0.999985
cg0331576	-0.09619	0.034939	-2.75299	0.006718	0.999985
cg1831506	0.276645	0.100501	2.752655	0.006725	0.999985
cg2226754	0.102523	0.037246	2.752582	0.006726	0.999985
cg1124479	0.092823	0.033725	2.752343	0.006731	0.999985
cg1254960	-0.15803	0.057416	-2.7523	0.006732	0.999985
cg1030964	0.145352	0.052814	2.752136	0.006735	0.999985
cg0854206	0.421374	0.153113	2.752046	0.006737	0.999985
cg1010746	-0.10369	0.037678	-2.75201	0.006738	0.999985

cg2100750	0.094166	0.034218	2.751915	0.006739	0.999985
cg0059812	-0.96198	0.349592	-2.75173	0.006743	0.999985
cg0640974	0.103851	0.037741	2.751698	0.006744	0.999985
cg1372481	-0.11322	0.041146	-2.7516	0.006746	0.999985
cg1210627	0.385719	0.140223	2.750748	0.006762	0.999985
cg0649762	0.149259	0.054262	2.750715	0.006763	0.999985
cg0252876	0.064165	0.023327	2.75069	0.006763	0.999985
cg0952651	-0.08887	0.03231	-2.7506	0.006765	0.999985
cg1863263	-0.16579	0.060283	-2.75027	0.006771	0.999985
cg2172471	0.162724	0.059177	2.749794	0.006781	0.999985
cg2727830	-0.07232	0.026307	-2.7492	0.006792	0.999985
cg0984429	-0.10257	0.03731	-2.74907	0.006795	0.999985
cg1990442	0.165463	0.060193	2.748869	0.006799	0.999985
cg1066797	0.105747	0.03847	2.748803	0.0068	0.999985
cg0647310	0.126655	0.046085	2.748309	0.00681	0.999985
cg0966319	0.074009	0.02693	2.748225	0.006812	0.999985
cg2319990	0.09045	0.032913	2.748172	0.006813	0.999985
cg0033066	-0.06866	0.024983	-2.74813	0.006814	0.999985
cg0693985	0.162229	0.059033	2.748105	0.006814	0.999985
cg2074541	-0.10865	0.039541	-2.74774	0.006821	0.999985
cg1942982	0.090124	0.0328	2.747726	0.006821	0.999985
cg2287752	0.107811	0.039237	2.747647	0.006823	0.999985
cg2381011	-0.13779	0.050151	-2.74752	0.006825	0.999985
cg1930136	0.542017	0.19729	2.747315	0.00683	0.999985
cg1002661	-0.08509	0.030971	-2.74729	0.00683	0.999985
cg0834612	-0.10681	0.038885	-2.74685	0.006839	0.999985
cg0191588	0.132211	0.048136	2.746619	0.006843	0.999985
cg1813555	-0.23008	0.083771	-2.74658	0.006844	0.999985
cg1362540	0.125418	0.045664	2.746536	0.006845	0.999985
cg1704798	-0.09224	0.033597	-2.7454	0.006867	0.999985
cg0701417	-0.12392	0.045139	-2.74539	0.006868	0.999985
cg2310219	-0.4117	0.149963	-2.74538	0.006868	0.999985
cg2347053	-0.10054	0.036622	-2.74532	0.006869	0.999985
cg1563408	0.572164	0.20845	2.744857	0.006878	0.999985
cg1513237	-0.13161	0.047947	-2.74485	0.006878	0.999985
cg0075703	0.318273	0.115956	2.744783	0.00688	0.999985
cg2325339	0.236823	0.086285	2.744678	0.006882	0.999985
cg2753985	0.102863	0.037478	2.744638	0.006882	0.999985
cg2567616	-0.34539	0.125843	-2.74462	0.006883	0.999985
cg0466967	-0.06799	0.024774	-2.74426	0.00689	0.999985
cg2262814	0.117093	0.042669	2.744232	0.006891	0.999985
cg2159764	0.098604	0.035938	2.74373	0.0069	0.999985
cg2709623	0.121073	0.044133	2.743349	0.006908	0.999985
cg1086734	0.210445	0.076717	2.74315	0.006912	0.999985
cg0170779	-0.42781	0.155956	-2.74313	0.006912	0.999985
cg0405638	-0.17501	0.063813	-2.74258	0.006923	0.999985
cg0890007	0.108151	0.039435	2.742475	0.006925	0.999985
cg1308859	0.119666	0.043635	2.742456	0.006926	0.999985
cg2530627	-0.23455	0.085536	-2.74208	0.006933	0.999985
cg0101317	-0.07866	0.028692	-2.74164	0.006942	0.999985

cg0554583	0.098594	0.035962	2.741613	0.006943	0.999985
cg1766218	-0.08464	0.030876	-2.74127	0.00695	0.999985
cg2044519	-0.11493	0.041927	-2.74122	0.006951	0.999985
cg2451388	0.297758	0.108626	2.741134	0.006952	0.999985
cg1462934	-0.08577	0.031292	-2.74093	0.006956	0.999985
cg0383379	0.113955	0.041578	2.740775	0.006959	0.999985
cg0719091	0.103978	0.03794	2.740606	0.006963	0.999985
cg1191552	-0.1194	0.04357	-2.74054	0.006964	0.999985
cg1097012	0.050406	0.018394	2.740344	0.006968	0.999985
cg0515856	-0.08486	0.030967	-2.74028	0.006969	0.999985
cg1827102	0.1214	0.044306	2.740044	0.006974	0.999985
cg0305716	-0.07148	0.026089	-2.73992	0.006977	0.999985
cg0080629	-0.11026	0.040245	-2.73976	0.00698	0.999985
cg0077277	0.115002	0.041976	2.739745	0.00698	0.999985
cg0327755	-0.23181	0.08461	-2.73973	0.00698	0.999985
cg1028552	-0.40805	0.148953	-2.73948	0.006985	0.999985
cg1397157	-0.11267	0.041129	-2.73935	0.006988	0.999985
cg2183128	0.094503	0.034502	2.739074	0.006994	0.999985
cg1853614	-0.07743	0.028272	-2.73891	0.006997	0.999985
cg1416894	0.121734	0.044448	2.738802	0.006999	0.999985
cg2139754	0.299252	0.109265	2.738763	0.007	0.999985
cg1670743	-0.12091	0.04415	-2.73872	0.007001	0.999985
cg1827341	-0.09156	0.033435	-2.73851	0.007005	0.999985
cg0826620	-0.08259	0.030162	-2.73813	0.007013	0.999985
cg0548713	0.165041	0.060279	2.737974	0.007016	0.999985
cg0437297	-0.09771	0.035689	-2.73794	0.007016	0.999985
cg2030167	-0.08771	0.032036	-2.7378	0.007019	0.999985
cg0597077	-0.21617	0.078958	-2.73777	0.00702	0.999985
cg2224411	-0.13441	0.049097	-2.73762	0.007023	0.999985
cg0866899	0.084777	0.030968	2.737609	0.007023	0.999985
cg0390986	0.422355	0.154279	2.737605	0.007023	0.999985
cg2660762	-0.43934	0.160506	-2.73724	0.007031	0.999985
cg0128419	0.114921	0.041987	2.737071	0.007034	0.999985
cg1019893	0.104604	0.038218	2.737031	0.007035	0.999985
cg1484043	0.285241	0.104216	2.737013	0.007035	0.999985
cg0296305	0.104978	0.038357	2.73684	0.007039	0.999985
cg0007649	-0.09546	0.034878	-2.73681	0.007039	0.999985
cg0171524	-0.14873	0.054356	-2.7362	0.007052	0.999985
cg2413356	0.227932	0.083306	2.736094	0.007054	0.999985
cg2262735	0.081062	0.029628	2.735938	0.007057	0.999985
cg0909678	0.196319	0.07176	2.735773	0.00706	0.999985
cg1836233	-0.11901	0.043504	-2.73565	0.007063	0.999985
cg0348362	-0.13786	0.050395	-2.73559	0.007064	0.999985
cg0815532	-0.28973	0.105919	-2.73534	0.007069	0.999985
cg0030090	-0.13002	0.047537	-2.73504	0.007075	0.999985
cg2748461	-0.1361	0.049765	-2.73475	0.007081	0.999985
cg0675544	-0.10322	0.037745	-2.73466	0.007083	0.999985
cg0581053	-0.11536	0.042186	-2.73456	0.007085	0.999985
cg1706863	-0.25141	0.091941	-2.7345	0.007086	0.999985
cg1221509	-0.08625	0.031544	-2.7343	0.00709	0.999985

cg1439085	-0.08064	0.029503	-2.73337	0.007109	0.999985
cg1749688	-0.05058	0.018505	-2.7333	0.007111	0.999985
cg0300333	0.352083	0.128825	2.733034	0.007116	0.999985
cg0749535	0.156002	0.05708	2.733027	0.007116	0.999985
cg1298827	0.10669	0.039038	2.733008	0.007117	0.999985
cg0377898	-0.12284	0.044949	-2.73284	0.00712	0.999985
cg0310250	0.086081	0.031501	2.732601	0.007125	0.999985
cg1354587	-0.08187	0.029961	-2.73257	0.007126	0.999985
cg1132338	-0.14518	0.053129	-2.73257	0.007126	0.999985
cg1930636	-0.08133	0.029762	-2.73251	0.007127	0.999985
cg1973797	0.144909	0.053037	2.732245	0.007132	0.999985
cg2116347	0.081036	0.029659	2.732241	0.007132	0.999985
cg0428446	-0.08968	0.032825	-2.7322	0.007133	0.999985
cg0236789	-0.09609	0.035175	-2.73188	0.00714	0.999985
cg0462587	-0.18683	0.068392	-2.73183	0.007141	0.999985
cg1128066	0.261625	0.095781	2.731487	0.007148	0.999985
cg1085354	-0.11803	0.043213	-2.73147	0.007148	0.999985
cg0093565	-0.05531	0.020253	-2.73119	0.007154	0.999985
cg0545774	-0.11831	0.043322	-2.73103	0.007157	0.999985
cg1922352	0.106561	0.039023	2.730734	0.007163	0.999985
cg2274200	0.110074	0.04031	2.730708	0.007164	0.999985
cg2576425	0.087118	0.031904	2.730591	0.007166	0.999985
cg0828538	-0.07852	0.028755	-2.73055	0.007167	0.999985
cg0727312	0.110447	0.040449	2.730519	0.007168	0.999985
cg0835344	0.149896	0.054901	2.730313	0.007172	0.999985
cg0159725	0.223043	0.081694	2.730228	0.007174	0.999985
cg2044682	0.172904	0.063338	2.729862	0.007181	0.999985
cg1085072	0.126514	0.046347	2.72972	0.007184	0.999985
cg0447887	-0.09903	0.036282	-2.72939	0.007191	0.999985
cg0743112	0.08395	0.030762	2.729013	0.007199	0.999985
cg1217917	-0.27534	0.100902	-2.7288	0.007203	0.999985
cg0498558	-0.45571	0.167009	-2.72867	0.007206	0.999985
cg0214120	-0.74547	0.273212	-2.72853	0.007209	0.999985
cg2701973	-0.09475	0.034725	-2.72852	0.007209	0.999985
cg1634623	0.107592	0.039439	2.728067	0.007218	0.999985
cg0371512	0.176502	0.064703	2.7279	0.007222	0.999985
cg0780564	-0.13632	0.049974	-2.72778	0.007224	0.999985
cg0930079	0.161726	0.059291	2.727675	0.007227	0.999985
cg1412525	-0.09723	0.035647	-2.72767	0.007227	0.999985
cg2439489	0.072009	0.0264	2.727619	0.007228	0.999985
cg0732732	-0.10424	0.038217	-2.72759	0.007228	0.999985
cg0141055	0.136946	0.050208	2.727574	0.007229	0.999985
cg2704607	-0.07981	0.029261	-2.7275	0.00723	0.999985
cg1377015	0.223609	0.081988	2.727338	0.007234	0.999985
cg0489049	-0.14264	0.052304	-2.72719	0.007237	0.999985
cg0395851	0.115702	0.04243	2.726921	0.007242	0.999985
cg0436469	0.320805	0.117654	2.726684	0.007247	0.999985
cg2206928	-0.10014	0.036727	-2.72662	0.007248	0.999985
cg2017601	-0.09385	0.034423	-2.72626	0.007256	0.999985
cg2374344	0.084095	0.030847	2.726211	0.007257	0.999985

cg1870990	1.01303	0.371601	2.726125	0.007259	0.999985
cg2216867	-0.11874	0.043562	-2.72582	0.007265	0.999985
cg1397552	0.10648	0.039065	2.725679	0.007268	0.999985
cg1541724	-0.10455	0.03836	-2.72558	0.00727	0.999985
cg2549473	-0.12143	0.044556	-2.72546	0.007273	0.999985
cg1426621	-0.08112	0.029766	-2.72543	0.007273	0.999985
cg0695167	-0.19475	0.071465	-2.7251	0.00728	0.999985
cg1405308	0.103766	0.038079	2.725012	0.007282	0.999985
cg0071844	-0.08837	0.032428	-2.72497	0.007283	0.999985
cg1804956	0.080693	0.029614	2.724783	0.007287	0.999985
cg2518368	-0.09431	0.034614	-2.72468	0.007289	0.999985
cg0268503	-0.11747	0.043116	-2.72445	0.007294	0.999985
cg0949052	-0.10462	0.038409	-2.72395	0.007304	0.999985
cg0106984	-0.06478	0.023782	-2.72378	0.007308	0.999985
cg1954623	0.191179	0.070205	2.723164	0.007321	0.999985
cg0081389	-0.08914	0.032734	-2.72303	0.007324	0.999985
cg2391041	0.159659	0.058634	2.722957	0.007325	0.999985
cg0029457	0.082182	0.030181	2.722947	0.007325	0.999985
cg0170499	0.077341	0.028404	2.722938	0.007325	0.999985
cg2621378	-0.16083	0.059076	-2.72238	0.007337	0.999985
cg0138391	0.097864	0.035948	2.722372	0.007337	0.999985
cg0994126	-0.14474	0.053169	-2.72231	0.007339	0.999985
cg1226823	0.243326	0.089391	2.722044	0.007344	0.999985
cg0432063	-0.10265	0.037714	-2.72181	0.007349	0.999985
cg2469069	0.081252	0.029854	2.721662	0.007352	0.999985
cg0743675	0.087439	0.032128	2.721616	0.007353	0.999985
cg2398029	-0.05771	0.021208	-2.72111	0.007364	0.999985
cg1435364	-0.12721	0.046757	-2.72067	0.007373	0.999985
cg0663579	0.173328	0.063711	2.720543	0.007376	0.999985
cg0923437	0.150017	0.055151	2.720126	0.007385	0.999985
cg0581723	0.098112	0.036073	2.719837	0.007391	0.999985
cg2705278	-0.1621	0.059601	-2.71976	0.007393	0.999985
cg1486109	-0.18621	0.068468	-2.71961	0.007396	0.999985
cg2268215	-0.19242	0.070771	-2.71886	0.007412	0.999985
cg0065673	0.106561	0.039196	2.718702	0.007415	0.999985
cg2469336	0.059636	0.021936	2.718633	0.007416	0.999985
cg2025869	0.119696	0.044033	2.718304	0.007423	0.999985
cg2252126	0.170638	0.062775	2.718243	0.007425	0.999985
cg0660947	-0.34592	0.127265	-2.71808	0.007428	0.999985
cg0765765	0.121268	0.044627	2.717361	0.007443	0.999985
cg0241337	0.405731	0.149333	2.716955	0.007452	0.999985
cg1322945	-0.14111	0.051944	-2.71661	0.007459	0.999985
cg0745279	-0.06815	0.025086	-2.7166	0.00746	0.999985
cg0608431	0.119356	0.04394	2.716339	0.007465	0.999985
cg0001007	-0.06704	0.024683	-2.71601	0.007472	0.999985
cg1967667	-0.17099	0.062956	-2.71598	0.007473	0.999985
cg0308845	0.13865	0.05105	2.715963	0.007473	0.999985
cg2445187	-0.29376	0.108173	-2.7157	0.007479	0.999985
cg0631556	-0.12879	0.04743	-2.7154	0.007485	0.999985
cg1757425	0.0995	0.036648	2.71501	0.007494	0.999985

cg0401279	-0.08998	0.033142	-2.71501	0.007494	0.999985
cg2416893	-0.09015	0.033204	-2.715	0.007494	0.999985
cg0189332	-0.10748	0.039591	-2.71464	0.007502	0.999985
cg0040968	-0.0494	0.0182	-2.71442	0.007506	0.999985
cg1141204	0.07427	0.027364	2.714183	0.007511	0.999985
cg0770801	-0.13048	0.048073	-2.71412	0.007513	0.999985
cg2425512	-0.13436	0.049507	-2.71404	0.007514	0.999985
cg2267600	0.093642	0.034506	2.713817	0.007519	0.999985
cg0508237	0.411037	0.151469	2.713669	0.007522	0.999985
cg1522777	0.083816	0.030888	2.713549	0.007525	0.999985
cg0519635	0.180032	0.066347	2.713475	0.007527	0.999985
cg0101611	0.114748	0.042294	2.713077	0.007535	0.999985
cg1346488	0.098049	0.036141	2.712932	0.007538	0.999985
cg1491992	0.316518	0.116675	2.712819	0.007541	0.999985
cg0247282	-0.07608	0.028046	-2.71276	0.007542	0.999985
cg0431776	-0.13093	0.048266	-2.71268	0.007544	0.999985
cg0044021	-0.13427	0.049512	-2.71189	0.007561	0.999985
cg0699096	0.184283	0.067969	2.711265	0.007574	0.999985
cg2444274	0.182151	0.067185	2.711176	0.007576	0.999985
cg1154720	0.203991	0.075245	2.711014	0.00758	0.999985
cg0084722	-0.07807	0.028802	-2.71041	0.007593	0.999985
cg0754585	-0.08568	0.031615	-2.71025	0.007596	0.999985
cg0513808	-0.10074	0.037171	-2.71022	0.007597	0.999985
cg2551142	-0.08689	0.032066	-2.70988	0.007604	0.999985
cg0979614	-0.19838	0.073209	-2.70974	0.007607	0.999985
cg1018879	-0.09336	0.034454	-2.70971	0.007608	0.999985
cg0330917	-0.0879	0.032441	-2.70955	0.007612	0.999985
cg2244216	0.117127	0.043237	2.708922	0.007625	0.999985
cg1171105	-0.11501	0.042462	-2.70847	0.007635	0.999985
cg0805343	0.131499	0.048558	2.708072	0.007644	0.999985
cg0590934	-0.18882	0.069725	-2.70805	0.007644	0.999985
cg1678190	-0.11007	0.040648	-2.70795	0.007646	0.999985
cg1938036	-0.13163	0.048618	-2.70738	0.007659	0.999985
cg2281244	0.155298	0.057363	2.707261	0.007661	0.999985
cg0779530	-0.10845	0.040059	-2.70717	0.007663	0.999985
cg0193383	0.103176	0.038114	2.707073	0.007666	0.999985
cg1050723	0.092462	0.03416	2.706747	0.007673	0.999985
cg0688741	-0.08538	0.031549	-2.70629	0.007683	0.999985
cg1195969	-0.18287	0.067579	-2.70598	0.007689	0.999985
cg1485397	-0.07814	0.028878	-2.70598	0.00769	0.999985
cg1651454	-0.10868	0.040165	-2.70586	0.007692	0.999985
cg1243118	-0.09667	0.035728	-2.70567	0.007696	0.999985
cg1527382	-0.11443	0.042297	-2.70548	0.0077	0.999985
cg1907154	-0.06024	0.022265	-2.70547	0.007701	0.999985
cg0276318	0.075247	0.027814	2.705359	0.007703	0.999985
cg1889302	0.097918	0.036198	2.705075	0.007709	0.999985
cg0079765	0.209262	0.077366	2.704834	0.007715	0.999985
cg1018636	-0.14796	0.054712	-2.70428	0.007727	0.999985
cg1032969	-0.0755	0.027917	-2.70426	0.007727	0.999985
ch.12.2538	0.271816	0.100516	2.704196	0.007729	0.999985

cg1327370	-0.13868	0.051286	-2.70414	0.00773	0.999985
cg0255351	-0.0781	0.028882	-2.7041	0.007731	0.999985
cg1366065	-0.09867	0.036491	-2.70403	0.007732	0.999985
cg1185864	0.096422	0.03566	2.703932	0.007735	0.999985
cg0326062	-0.18153	0.06714	-2.70378	0.007738	0.999985
cg1391081	0.246865	0.091305	2.703746	0.007739	0.999985
cg1581880	-0.11581	0.042832	-2.70368	0.00774	0.999985
cg2655086	-0.15982	0.059112	-2.70366	0.007741	0.999985
cg1644464	-0.07803	0.028862	-2.70357	0.007743	0.999985
cg1571725	-0.09357	0.03461	-2.70356	0.007743	0.999985
cg1699016	-0.06595	0.024395	-2.70332	0.007748	0.999985
cg2200203	-0.05375	0.019883	-2.70307	0.007753	0.999985
cg0709298	-0.13989	0.051756	-2.70287	0.007758	0.999985
cg1900188	-0.11312	0.041856	-2.70262	0.007764	0.999985
cg1834018	0.058432	0.021621	2.702603	0.007764	0.999985
cg0890344	0.126466	0.046799	2.702322	0.00777	0.999985
cg1179970	-0.20686	0.07655	-2.70225	0.007772	0.999985
cg1030754	-0.05808	0.021496	-2.70213	0.007774	0.999985
cg0484834	-0.14876	0.055052	-2.70211	0.007775	0.999985
cg1685993	-0.07988	0.029564	-2.70198	0.007778	0.999985
cg1812190	-1.15487	0.427434	-2.70188	0.00778	0.999985
cg2556187	-0.13028	0.048223	-2.70163	0.007785	0.999985
cg2458838	-0.09322	0.034514	-2.70086	0.007803	0.999985
cg2336288	0.136043	0.050371	2.700826	0.007803	0.999985
cg1605174	0.16333	0.060478	2.700654	0.007807	0.999985
cg1236206	0.116614	0.043182	2.700549	0.007809	0.999985
cg1168285	0.156291	0.057875	2.700502	0.007811	0.999985
cg1193982	0.10693	0.039597	2.70045	0.007812	0.999985
cg1678886	0.122216	0.045259	2.700353	0.007814	0.999985
cg2722855	-0.09084	0.033641	-2.70025	0.007816	0.999985
cg1891194	0.136558	0.050576	2.700053	0.007821	0.999985
cg2128912	0.138452	0.051287	2.699554	0.007832	0.999985
cg0545335	-0.40049	0.148354	-2.69954	0.007832	0.999985
cg2537244	0.957921	0.354857	2.699453	0.007834	0.999985
cg2299200	0.09515	0.035248	2.699432	0.007834	0.999985
cg1693836	-0.14552	0.053909	-2.69936	0.007836	0.999985
cg2489243	-0.14306	0.053009	-2.69889	0.007846	0.999985
cg0289993	-0.09474	0.035103	-2.69886	0.007847	0.999985
cg2366597	0.072383	0.026825	2.698353	0.007858	0.999985
cg0430876	-0.10515	0.038971	-2.69818	0.007862	0.999985
cg0076152	-0.08943	0.033145	-2.69817	0.007863	0.999985
cg1341249	-0.08712	0.032288	-2.69811	0.007864	0.999985
cg1352053	-0.17932	0.066475	-2.69757	0.007876	0.999985
cg2169751	-0.073	0.027061	-2.69751	0.007877	0.999985
cg0040395	0.190188	0.070513	2.697203	0.007884	0.999985
cg1361404	-0.09465	0.035095	-2.69688	0.007892	0.999985
cg1075605	-0.10676	0.039594	-2.69647	0.007901	0.999985
cg0235157	0.128021	0.04748	2.69633	0.007904	0.999985
cg0180241	-0.14946	0.055431	-2.69628	0.007905	0.999985
cg1867932	-0.10073	0.037359	-2.69616	0.007908	0.999985

cg0725686	-0.0953	0.035346	-2.69605	0.00791	0.999985
cg2422430	0.074721	0.027721	2.695442	0.007924	0.999985
cg0556985	0.086488	0.032089	2.695298	0.007927	0.999985
cg2404328	-0.11183	0.041494	-2.695	0.007934	0.999985
cg0778522	-0.06732	0.024982	-2.69465	0.007942	0.999985
cg2568674	-0.07768	0.028828	-2.69461	0.007943	0.999985
cg1288327	-0.13508	0.050131	-2.69454	0.007944	0.999985
cg2029496	-0.08383	0.031115	-2.69425	0.007951	0.999985
cg0087666	-0.16792	0.062325	-2.69421	0.007952	0.999985
cg2335683	-0.20622	0.076543	-2.69411	0.007954	0.999985
cg2582070	-0.11605	0.043077	-2.69402	0.007956	0.999985
cg0102287	0.123335	0.045784	2.693866	0.007959	0.999985
cg0768733	-0.08338	0.030953	-2.69373	0.007962	0.999985
cg1384556	-0.09738	0.036153	-2.6936	0.007966	0.999985
cg1980112	0.11936	0.044315	2.693439	0.007969	0.999985
cg1709748	0.083874	0.031141	2.693341	0.007971	0.999985
cg0745129	0.07796	0.028946	2.693319	0.007972	0.999985
cg2068214	-0.09602	0.035657	-2.69282	0.007983	0.999985
cg1603986	-0.10349	0.038436	-2.69246	0.007991	0.999985
cg0869243	-0.06576	0.024423	-2.69233	0.007994	0.999985
cg1810228	-0.12493	0.046405	-2.69212	0.007999	0.999985
cg2234938	-0.45516	0.169076	-2.69205	0.008001	0.999985
cg1023031	0.095906	0.035626	2.691995	0.008002	0.999985
cg1664977	-0.10426	0.038731	-2.69188	0.008004	0.999985
cg1778172	0.094913	0.035263	2.691597	0.008011	0.999985
cg0375824	0.087611	0.032551	2.691489	0.008013	0.999985
cg0907115	-0.1016	0.037749	-2.69146	0.008014	0.999985
cg2034902	-0.59165	0.219829	-2.69142	0.008015	0.999985
cg0424587	0.403563	0.149959	2.691153	0.008021	0.999985
cg0141435	0.085569	0.031797	2.691081	0.008023	0.999985
cg1843456	-0.09339	0.034709	-2.69075	0.00803	0.999985
cg0537654	0.132104	0.049097	2.690661	0.008032	0.999985
cg0488013	-0.12476	0.046368	-2.69062	0.008033	0.999985
cg2370833	-0.39046	0.145131	-2.69037	0.008039	0.999985
cg1612237	-0.0793	0.029475	-2.69034	0.00804	0.999985
cg1579214	0.104476	0.038838	2.690083	0.008046	0.999985
cg1141183	0.11844	0.04403	2.689985	0.008048	0.999985
cg1146863	-0.07202	0.026776	-2.6897	0.008054	0.999985
cg0816415	-0.84869	0.315549	-2.68955	0.008058	0.999985
cg2758181	-0.07365	0.027385	-2.68955	0.008058	0.999985
cg1019520	-0.06251	0.023244	-2.68947	0.00806	0.999985
cg0375677	0.110996	0.041271	2.68942	0.008061	0.999985
cg1667744	-0.11135	0.041404	-2.68937	0.008062	0.999985
cg2457921	0.296863	0.110388	2.689263	0.008064	0.999985
cg0222048	-0.10142	0.037716	-2.68911	0.008068	0.999985
cg0860238	-0.07543	0.028051	-2.68898	0.008071	0.999985
cg0203298	0.143203	0.053258	2.688844	0.008074	0.999985
cg2582240	-0.05746	0.021371	-2.68873	0.008077	0.999985
cg1111877	0.12756	0.047451	2.688274	0.008087	0.999985
cg1638041	0.068368	0.025435	2.687916	0.008095	0.999985

cg0513359	0.373444	0.13895	2.687614	0.008102	0.999985
cg0494124	0.108731	0.040463	2.687146	0.008113	0.999985
cg1002772	0.129863	0.048331	2.686959	0.008117	0.999985
cg0033384	-0.09712	0.036152	-2.68649	0.008128	0.999985
cg1310522	0.066535	0.024767	2.686409	0.00813	0.999985
cg0992245	0.193387	0.071988	2.686381	0.008131	0.999985
cg0392815	-0.17474	0.065046	-2.68636	0.008131	0.999985
cg0319618	-0.07796	0.02902	-2.68636	0.008131	0.999985
cg0791584	-0.15844	0.058981	-2.6863	0.008132	0.999985
cg0677773	-0.61988	0.230765	-2.6862	0.008135	0.999985
cg2193655	-0.07641	0.02845	-2.68592	0.008141	0.999985
cg0675835	0.245353	0.091358	2.685625	0.008148	0.999985
cg2199225	-0.06451	0.024021	-2.68551	0.008151	0.999985
cg0045719	-0.12322	0.045884	-2.6855	0.008151	0.999985
cg0478939	0.109017	0.040609	2.684538	0.008173	0.999985
cg0679916	-0.09497	0.035378	-2.68453	0.008173	0.999985
cg0632872	-0.12282	0.045759	-2.68414	0.008182	0.999985
cg1287821	0.100848	0.037572	2.684108	0.008183	0.999985
cg1446338	0.079557	0.029641	2.684051	0.008185	0.999985
cg1345221	-0.09999	0.037255	-2.68401	0.008185	0.999985
cg1792911	-0.07596	0.028301	-2.684	0.008186	0.999985
cg1301364	0.124213	0.04628	2.68392	0.008188	0.999985
cg2541235	-0.123	0.045832	-2.68371	0.008192	0.999985
cg0346652	0.14461	0.053887	2.683596	0.008195	0.999985
cg0103345	0.169381	0.063127	2.683153	0.008205	0.999985
cg0951452	0.167234	0.062331	2.682997	0.008209	0.999985
cg2416806	-0.07068	0.026343	-2.6829	0.008211	0.999985
cg0153452	-0.09177	0.034206	-2.68275	0.008215	0.999985
cg1042475	-0.2414	0.089986	-2.68268	0.008216	0.999985
cg0199357	0.086035	0.032074	2.682426	0.008222	0.999985
cg1439392	0.130725	0.048738	2.682205	0.008227	0.999985
cg0868406	0.837189	0.312129	2.682187	0.008228	0.999985
cg1913017	-0.04845	0.018063	-2.68218	0.008228	0.999985
cg2613572	-0.0694	0.025875	-2.6821	0.00823	0.999985
cg0932071	-0.12547	0.046786	-2.68182	0.008236	0.999985
cg0546032	0.074882	0.027924	2.681658	0.00824	0.999985
cg2575275	-0.09019	0.033636	-2.6815	0.008244	0.999985
cg0092252	-0.10682	0.039838	-2.68143	0.008245	0.999985
cg1309570	0.107074	0.039933	2.681358	0.008247	0.999985
cg1860315	-0.10375	0.038693	-2.68125	0.00825	0.999985
cg1922659	0.077716	0.028988	2.680946	0.008257	0.999985
cg1809940	0.128534	0.047947	2.680757	0.008261	0.999985
cg0194237	-0.07424	0.027695	-2.68052	0.008267	0.999985
cg2153500	0.189619	0.070749	2.680144	0.008276	0.999985
cg0621346	-0.11093	0.041393	-2.67987	0.008282	0.999985
cg2433004	0.355299	0.132594	2.679598	0.008288	0.999985
cg1478964	-0.0528	0.019706	-2.67935	0.008294	0.999985
cg0513744	0.354657	0.132376	2.679162	0.008299	0.999985
cg2482772	-0.07997	0.029849	-2.67902	0.008302	0.999985
cg1005186	0.186901	0.069768	2.678897	0.008305	0.999985

cg0036384	-0.12621	0.047119	-2.67862	0.008312	0.999985
cg0206382	0.070847	0.026449	2.678581	0.008312	0.999985
cg0782549	-0.09244	0.034511	-2.67845	0.008316	0.999985
cg0954680	0.106713	0.039845	2.67822	0.008321	0.999985
cg0117905	0.104093	0.038867	2.678185	0.008322	0.999985
cg2505338	-0.17226	0.064319	-2.67818	0.008322	0.999985
cg1528789	-0.18112	0.067629	-2.67813	0.008323	0.999985
cg0796904	-0.07639	0.028523	-2.67805	0.008325	0.999985
cg2341263	-0.07796	0.029112	-2.67798	0.008327	0.999985
cg1508426	-0.11968	0.044691	-2.67794	0.008327	0.999985
cg1235367	0.176697	0.065985	2.677844	0.00833	0.999985
cg0034596	-0.09187	0.034306	-2.67783	0.00833	0.999985
cg0399115	-0.09176	0.034275	-2.67723	0.008344	0.999985
cg2550089	0.104862	0.039168	2.677218	0.008345	0.999985
cg2319870	0.076893	0.028722	2.677147	0.008346	0.999985
cg1058938	0.261316	0.097615	2.676995	0.00835	0.999985
cg0001319	0.104984	0.039218	2.676956	0.008351	0.999985
cg2378096	-0.07924	0.029604	-2.67662	0.008359	0.999985
cg2283643	0.149031	0.055679	2.676597	0.008359	0.999985
cg0722196	-0.14633	0.054671	-2.67656	0.00836	0.999985
cg2146263	0.189401	0.070764	2.676504	0.008361	0.999985
cg2632497	-0.10687	0.03993	-2.67647	0.008362	0.999985
cg0893777	-0.07265	0.027146	-2.67647	0.008362	0.999985
cg0855896	0.096696	0.036129	2.676397	0.008364	0.999985
cg1774519	0.326213	0.121901	2.676053	0.008372	0.999985
cg0575114	-0.0685	0.0256	-2.67599	0.008374	0.999985
cg2271086	0.086008	0.032141	2.675972	0.008374	0.999985
cg2355856	0.083771	0.031308	2.675677	0.008381	0.999985
cg0237498	-0.38823	0.145103	-2.67552	0.008385	0.999985
cg1404293	-0.12853	0.04804	-2.6755	0.008385	0.999985
cg1770563	-0.06377	0.023837	-2.67531	0.00839	0.999985
cg1371479	-0.13637	0.050977	-2.67511	0.008394	0.999985
cg2014738	-0.0804	0.030055	-2.67511	0.008394	0.999985
cg2509267	-0.1185	0.044298	-2.67509	0.008395	0.999985
cg0015002	-0.11848	0.044292	-2.67508	0.008395	0.999985
cg0153739	-0.10686	0.039948	-2.67496	0.008398	0.999985
cg0505705	-0.12915	0.048281	-2.67491	0.008399	0.999985
cg0367422	-0.11508	0.043026	-2.67473	0.008403	0.999985
cg2000363	-0.11739	0.043889	-2.67471	0.008404	0.999985
cg2391180	-0.0918	0.034323	-2.67469	0.008404	0.999985
cg1108054	-0.0756	0.028267	-2.67434	0.008413	0.999985
cg0217640	-0.11788	0.044079	-2.6743	0.008414	0.999985
cg2487837	-0.08897	0.033272	-2.67416	0.008417	0.999985
cg1555033	-0.17471	0.065338	-2.67393	0.008423	0.999985
cg1993152	-0.13335	0.049869	-2.67391	0.008423	0.999985
cg0076313	0.141807	0.053038	2.673659	0.008429	0.999985
cg2675135	0.108979	0.04076	2.673658	0.008429	0.999985
cg0066016	0.094141	0.035211	2.673626	0.00843	0.999985
cg2512544	0.10148	0.037958	2.673478	0.008433	0.999985
cg1711906	-0.17745	0.066379	-2.67327	0.008438	0.999985

cg0111391	0.097832	0.036597	2.673205	0.00844	0.999985
cg2637518	-0.08896	0.03328	-2.67303	0.008444	0.999985
cg1509174	0.216716	0.081084	2.67272	0.008451	0.999985
cg0779016	-0.11903	0.044541	-2.67247	0.008458	0.999985
cg2607772	-0.25374	0.094949	-2.67243	0.008458	0.999985
cg2222547	0.115169	0.043097	2.672335	0.008461	0.999985
cg2046629	-0.08551	0.031999	-2.67233	0.008461	0.999985
cg0070848	0.181513	0.067932	2.671996	0.008469	0.999985
cg0047244	-0.08066	0.030188	-2.67198	0.008469	0.999985
cg2105839	-0.10235	0.038307	-2.67191	0.008471	0.999985
cg2213809	0.080272	0.030043	2.671853	0.008472	0.999985
cg2486527	-0.06825	0.02555	-2.67118	0.008488	0.999985
cg2452174	0.086262	0.032295	2.671089	0.008491	0.999985
cg0341072	-0.09121	0.034148	-2.67103	0.008492	0.999985
cg0356323	0.116205	0.043506	2.671002	0.008493	0.999985
cg1898482	-0.05866	0.021961	-2.67088	0.008496	0.999985
cg0074996	0.147455	0.05521	2.670791	0.008498	0.999985
cg2219208	-0.09806	0.036716	-2.67074	0.008499	0.999985
cg2317653	-0.09264	0.034688	-2.67065	0.008501	0.999985
cg2657689	0.247666	0.09274	2.67055	0.008503	0.999985
cg1033613	0.064842	0.024281	2.670459	0.008506	0.999985
cg1109972	0.123471	0.046236	2.670444	0.008506	0.999985
cg2391453	-0.17423	0.065244	-2.67039	0.008507	0.999985
cg0420128	-0.13731	0.051421	-2.67025	0.008511	0.999985
cg1830324	0.070598	0.02644	2.670118	0.008514	0.999985
cg2456549	-0.08569	0.032093	-2.67008	0.008515	0.999985
cg1282142	-0.06842	0.02563	-2.66975	0.008523	0.999985
cg1395645	0.155553	0.058266	2.669699	0.008524	0.999985
cg1592677	-0.08122	0.030422	-2.66966	0.008525	0.999985
cg2268685	-0.17344	0.064972	-2.66946	0.00853	0.999985
cg2402328	0.12852	0.048148	2.669281	0.008534	0.999985
cg2051154	-0.19353	0.072502	-2.66926	0.008535	0.999985
cg1018128	0.23277	0.087209	2.669115	0.008538	0.999985
cg2478792	-0.10837	0.040602	-2.66896	0.008542	0.999985
cg0702768	0.1326	0.049683	2.668904	0.008543	0.999985
cg2075052	0.146822	0.055015	2.668787	0.008546	0.999985
cg1091199	-0.05985	0.02243	-2.66851	0.008553	0.999985
cg1181741	-0.09129	0.03421	-2.66835	0.008556	0.999985
cg1348073	-0.08665	0.032476	-2.66825	0.008559	0.999985
cg0210392	-0.073	0.027359	-2.66819	0.00856	0.999985
cg1806694	-0.09438	0.035376	-2.66796	0.008566	0.999985
cg2652109	0.115242	0.043196	2.667881	0.008568	0.999985
cg1748064	0.111095	0.041647	2.667556	0.008576	0.999985
cg1510153	-0.17527	0.065706	-2.66754	0.008576	0.999985
cg2437759	0.061147	0.022923	2.667532	0.008576	0.999985
cg0170324	-0.12613	0.047283	-2.6675	0.008577	0.999985
cg2369356	0.311552	0.116803	2.667328	0.008581	0.999985
cg0110844	-0.123	0.046113	-2.66733	0.008581	0.999985
cg0126834	-0.14979	0.05616	-2.66723	0.008584	0.999985
cg0229101	-0.07682	0.028802	-2.66721	0.008584	0.999985

cg2323873	0.108073	0.040519	2.667183	0.008585	0.999985
cg2088277	-0.0986	0.03697	-2.66712	0.008586	0.999985
cg0705744	-0.11707	0.043899	-2.66674	0.008595	0.999985
cg0767223	0.124428	0.04666	2.666699	0.008596	0.999985
cg0218128	-0.10412	0.039044	-2.66667	0.008597	0.999985
cg0104123	-0.09392	0.035221	-2.66662	0.008598	0.999985
cg1793672	0.112241	0.042093	2.666532	0.008601	0.999985
cg0016917	-0.10227	0.038352	-2.6665	0.008601	0.999985
cg2485012	0.133477	0.050058	2.666457	0.008602	0.999985
cg0107009	-0.10657	0.039967	-2.66639	0.008604	0.999985
cg2252307	-0.12899	0.048382	-2.66615	0.00861	0.999985
cg0194070	-0.05707	0.021406	-2.66612	0.008611	0.999985
cg1827288	0.159381	0.05978	2.66611	0.008611	0.999985
cg0788639	-0.07554	0.028335	-2.66598	0.008614	0.999985
cg0286955	0.324644	0.121774	2.665961	0.008614	0.999985
cg0003858	-0.13638	0.051162	-2.66569	0.008621	0.999985
cg1421339	0.097944	0.036747	2.665346	0.008629	0.999985
cg0351484	0.108261	0.040622	2.665097	0.008635	0.999985
cg2412439	-0.06284	0.023583	-2.66477	0.008643	0.999985
cg0354636	-0.12234	0.045913	-2.66465	0.008646	0.999985
cg0411587	-0.19742	0.074094	-2.66445	0.008651	0.999985
cg1260085	-0.1749	0.065649	-2.66415	0.008658	0.999985
cg0101609	1.341306	0.503476	2.664088	0.00866	0.999985
cg0894914	-0.0805	0.030218	-2.66393	0.008664	0.999985
cg0669747	-0.11391	0.042761	-2.66391	0.008664	0.999985
cg1058264	0.078958	0.029643	2.66366	0.00867	0.999985
cg1595296	-0.07546	0.028332	-2.66343	0.008676	0.999985
cg0070046	-0.09756	0.036631	-2.66341	0.008677	0.999985
cg1037519	-0.06604	0.024797	-2.66338	0.008677	0.999985
cg0613503	-0.07465	0.028031	-2.66324	0.008681	0.999985
cg2348423	-0.10904	0.040941	-2.66323	0.008681	0.999985
cg0241708	0.116034	0.043569	2.66321	0.008682	0.999985
cg0262826	-0.08213	0.030839	-2.66311	0.008684	0.999985
cg0555032	0.098816	0.037106	2.663054	0.008685	0.999985
cg1113443	0.076412	0.0287	2.662451	0.0087	0.999985
cg1794049	0.097715	0.036703	2.662272	0.008705	0.999985
cg0822229	-0.09791	0.036785	-2.66179	0.008716	0.999985
cg1364436	-0.07501	0.028179	-2.66175	0.008717	0.999985
cg2250520	0.847126	0.318273	2.661634	0.00872	0.999985
cg2291978	0.088965	0.033426	2.661524	0.008723	0.999985
cg0592310	-0.07582	0.028489	-2.66148	0.008724	0.999985
cg0577663	-0.2179	0.081874	-2.66145	0.008725	0.999985
cg2219860	-0.08552	0.032132	-2.66142	0.008726	0.999985
cg1644684	-0.09994	0.037554	-2.66123	0.00873	0.999985
cg0354680	-0.10521	0.039536	-2.66107	0.008734	0.999985
cg2689678	-0.04812	0.018085	-2.66084	0.00874	0.999985
cg1395200	0.092227	0.034662	2.660794	0.008741	0.999985
cg1337090	-0.08347	0.031375	-2.66057	0.008746	0.999985
cg1909183	0.084709	0.03184	2.660482	0.008749	0.999985
cg2228314	-0.0734	0.027594	-2.65996	0.008762	0.999985

cg2733147	0.162862	0.061227	2.659944	0.008762	0.999985
cg0318076	-0.09098	0.034205	-2.65993	0.008762	0.999985
cg2590465	0.14718	0.055339	2.659626	0.00877	0.999985
cg1195435	0.109831	0.041297	2.659538	0.008772	0.999985
cg0106093	0.0745	0.028013	2.659436	0.008774	0.999985
cg0362874	-0.0777	0.02922	-2.65926	0.008779	0.999985
cg0902164	0.077507	0.029147	2.659203	0.00878	0.999985
cg0064948	-0.08594	0.03232	-2.65918	0.008781	0.999985
cg1562204	0.066042	0.024837	2.659002	0.008785	0.999985
cg0612804	-0.09481	0.035655	-2.65898	0.008786	0.999985
cg1948880	0.144052	0.054176	2.658964	0.008786	0.999985
cg2742578	-0.14313	0.053828	-2.65895	0.008786	0.999985
cg1610190	-0.09244	0.034767	-2.6589	0.008788	0.999985
cg0571787	0.257105	0.096707	2.658596	0.008795	0.999985
cg2320399	-0.08537	0.032112	-2.65859	0.008795	0.999985
cg0894342	-0.13221	0.049729	-2.65858	0.008796	0.999985
cg1028919	-0.08366	0.031469	-2.65839	0.0088	0.999985
cg0590296	-0.09507	0.035763	-2.65834	0.008801	0.999985
cg0515265	0.119678	0.045021	2.658271	0.008803	0.999985
cg2303834	0.089187	0.033553	2.658122	0.008807	0.999985
cg0369942	-0.23689	0.089122	-2.65805	0.008809	0.999985
cg2059795	0.122389	0.046046	2.657978	0.00881	0.999985
cg1860493	0.183972	0.069222	2.65769	0.008818	0.999985
cg1392708	-0.11253	0.042341	-2.65764	0.008819	0.999985
cg2288613	-0.08229	0.030965	-2.65737	0.008826	0.999985
cg1779283	0.076119	0.028646	2.657242	0.008829	0.999985
cg2214285	-0.06718	0.025284	-2.65701	0.008834	0.999985
cg2250028	-0.08276	0.031149	-2.65699	0.008835	0.999985
cg1145864	-0.07217	0.027161	-2.65696	0.008836	0.999985
cg0523221	0.07368	0.027731	2.65692	0.008837	0.999985
cg1265629	0.166451	0.06265	2.656822	0.008839	0.999985
cg0894665	0.13044	0.049097	2.656757	0.008841	0.999985
cg1442177	-0.10396	0.039134	-2.65645	0.008849	0.999985
cg2670823	-0.0682	0.025676	-2.65628	0.008853	0.999985
cg0205894	-0.11557	0.043509	-2.65624	0.008854	0.999985
cg0905190	-0.13823	0.052039	-2.6562	0.008855	0.999985
cg0558948	0.300245	0.113036	2.656193	0.008855	0.999985
cg0066460	0.079436	0.029907	2.656125	0.008857	0.999985
cg1292465	-0.07864	0.029607	-2.65607	0.008858	0.999985
cg1266196	0.080005	0.030122	2.656013	0.008859	0.999985
cg1843308	0.111266	0.041894	2.655895	0.008862	0.999985
cg1416530	0.069186	0.02605	2.655872	0.008863	0.999985
cg1314405	0.145764	0.054885	2.655827	0.008864	0.999985
cg2565251	0.108142	0.040723	2.655571	0.00887	0.999985
cg0923256	-0.09669	0.036414	-2.6553	0.008877	0.999985
cg2059428	-0.14794	0.055717	-2.65527	0.008878	0.999985
cg0770500	-0.08611	0.03243	-2.65521	0.00888	0.999985
cg0699811	-0.17269	0.065039	-2.65518	0.00888	0.999985
cg2315624	0.082528	0.031083	2.655052	0.008883	0.999985
cg0661176	-0.16056	0.060474	-2.65499	0.008885	0.999985

cg2228439	-0.04216	0.015878	-2.65495	0.008886	0.999985
cg1531800	0.078186	0.029449	2.654935	0.008886	0.999985
cg1553427	-0.09708	0.036567	-2.65491	0.008887	0.999985
cg0637292	0.108954	0.041042	2.654684	0.008893	0.999985
cg1866040	-0.07128	0.026854	-2.6545	0.008897	0.999985
cg2704740	-0.13368	0.050359	-2.65446	0.008898	0.999985
cg2400666	-0.07524	0.028347	-2.65423	0.008904	0.999985
cg2298173	-0.08774	0.033056	-2.65413	0.008906	0.999985
cg0568380	-0.07953	0.029969	-2.65369	0.008917	0.999985
cg0105092	-0.22305	0.084055	-2.65366	0.008918	0.999985
cg1156997	0.086043	0.032425	2.65358	0.00892	0.999985
cg0878607	0.21175	0.079801	2.653464	0.008923	0.999985
cg1527894	0.08652	0.032607	2.653451	0.008923	0.999985
cg1273911	0.143196	0.053966	2.653443	0.008924	0.999985
cg1940603	-0.078	0.029398	-2.65342	0.008924	0.999985
cg0411621	-0.09284	0.034991	-2.65319	0.00893	0.999985
cg2358345	0.130569	0.049218	2.652881	0.008938	0.999985
cg0112932	0.091678	0.034558	2.652876	0.008938	0.999985
cg1759655	0.095531	0.036023	2.651938	0.008962	0.999985
cg2347437	0.080742	0.030452	2.651469	0.008973	0.999985
cg1958531	0.077549	0.029248	2.651433	0.008974	0.999985
cg0495414	0.318802	0.120248	2.651215	0.00898	0.999985
cg0029928	0.451421	0.170278	2.651077	0.008983	0.999985
cg2129632	0.08758	0.033036	2.651049	0.008984	0.999985
cg1176802	-0.0742	0.02799	-2.65093	0.008987	0.999985
cg1402027	0.070656	0.026654	2.650886	0.008988	0.999985
cg0022515	-0.05264	0.019858	-2.65088	0.008988	0.999985
cg0902374	-0.05279	0.019915	-2.65073	0.008992	0.999985
cg0122813	0.05762	0.021737	2.650725	0.008992	0.999985
cg0679555	-0.12252	0.046222	-2.65069	0.008993	0.999985
cg1926105	0.075123	0.028341	2.650678	0.008993	0.999985
cg0633852	0.11653	0.043966	2.650451	0.008999	0.999985
cg2726241	-0.08646	0.032624	-2.65036	0.009001	0.999985
cg1038144	-0.06606	0.024928	-2.65014	0.009007	0.999985
cg0145844	-0.10079	0.038031	-2.65012	0.009007	0.999985
cg0371903	0.084743	0.031977	2.65009	0.009008	0.999985
cg2350129	0.08075	0.030473	2.649884	0.009013	0.999985
cg2463864	0.083635	0.031567	2.649454	0.009024	0.999985
cg1514307	0.084954	0.032067	2.649228	0.00903	0.999985
cg1531165	-0.07426	0.028033	-2.64901	0.009036	0.999985
cg0026336	-0.08474	0.031994	-2.64863	0.009045	0.999985
cg1303109	0.187374	0.070745	2.648603	0.009046	0.999985
cg2656931	-0.11932	0.045051	-2.6485	0.009048	0.999985
cg0548313	0.170696	0.064472	2.647593	0.009072	0.999985
cg0114230	0.25122	0.09489	2.64749	0.009074	0.999985
cg1189312	0.207815	0.078496	2.647455	0.009075	0.999985
cg1883950	-0.06781	0.025615	-2.6474	0.009077	0.999985
cg0888101	-0.08081	0.030525	-2.64739	0.009077	0.999985
cg2468893	0.112461	0.042483	2.647177	0.009082	0.999985
cg1067842	0.093665	0.035386	2.646966	0.009088	0.999985

cg2062821	0.079	0.029847	2.646889	0.00909	0.999985
cg1239247	-0.08898	0.033618	-2.64688	0.00909	0.999985
cg0257846	0.088567	0.033463	2.646688	0.009095	0.999985
cg2003278	-0.14579	0.055089	-2.6465	0.0091	0.999985
cg1664252	0.117178	0.044277	2.646495	0.0091	0.999985
cg1556589	0.079252	0.029946	2.646483	0.0091	0.999985
cg0260473	-0.08261	0.03122	-2.64623	0.009106	0.999985
cg1844325	0.209452	0.079152	2.646186	0.009108	0.999985
cg2466635	0.140717	0.053178	2.646144	0.009109	0.999985
cg1082789	0.073466	0.027764	2.646071	0.00911	0.999985
cg2691161	-0.15754	0.059537	-2.64603	0.009111	0.999985
cg0486345	0.088917	0.033605	2.645983	0.009113	0.999985
cg1975011	0.442645	0.167292	2.645938	0.009114	0.999985
cg0712788	0.097905	0.037003	2.645837	0.009116	0.999985
cg0023821	-0.07156	0.027046	-2.6457	0.00912	0.999985
cg2569884	-0.10306	0.038952	-2.6457	0.00912	0.999985
cg0483035	-0.12957	0.048986	-2.645	0.009138	0.999985
cg1088122	0.300324	0.113544	2.644992	0.009138	0.999985
cg0810796	-0.13511	0.051084	-2.64481	0.009143	0.999985
cg0079417	0.334765	0.126576	2.644785	0.009143	0.999985
cg2256935	-0.07067	0.026722	-2.6446	0.009148	0.999985
cg0875230	-0.07668	0.028997	-2.64454	0.00915	0.999985
cg1721215	0.15701	0.059372	2.64451	0.00915	0.999985
cg0179166	0.102253	0.038668	2.644388	0.009154	0.999985
cg1465559	0.126847	0.047971	2.644251	0.009157	0.999985
cg0098923	0.115111	0.043535	2.6441	0.009161	0.999985
cg2720264	-0.09751	0.036887	-2.64359	0.009174	0.999985
cg1202044	-0.14006	0.052981	-2.64355	0.009175	0.999985
cg2086485	-0.06877	0.026018	-2.64319	0.009184	0.999985
cg0733021	-0.0984	0.037229	-2.64302	0.009189	0.999985
cg0409965	0.075358	0.028515	2.642785	0.009195	0.999985
cg1138976	-0.09864	0.037324	-2.64278	0.009195	0.999985
cg1822254	-0.05468	0.020689	-2.64272	0.009196	0.999985
cg0526967	-0.05241	0.019833	-2.64256	0.009201	0.999985
cg1879280	0.129742	0.049103	2.642251	0.009209	0.999985
cg0198882	-0.0896	0.03391	-2.64223	0.009209	0.999985
cg2241756	-0.28544	0.108032	-2.64219	0.00921	0.999985
cg2741202	0.091332	0.03457	2.641917	0.009217	0.999985
cg2362212	0.266394	0.100836	2.641856	0.009219	0.999985
cg0700847	-0.10252	0.038808	-2.64173	0.009222	0.999985
cg0721281	0.197967	0.074944	2.64153	0.009227	0.999985
cg1305674	0.159136	0.060248	2.641362	0.009232	0.999985
cg0289248	-0.08322	0.031507	-2.64131	0.009233	0.999985
cg1503056	-0.11435	0.043291	-2.64131	0.009233	0.999985
cg2278771	-0.04678	0.01771	-2.64118	0.009236	0.999985
cg0209016	0.113882	0.043119	2.641139	0.009237	0.999985
cg2602748	-0.08485	0.032125	-2.64106	0.009239	0.999985
cg0848466	0.156034	0.059084	2.640868	0.009244	0.999985
cg2706470	-0.07408	0.028052	-2.64084	0.009245	0.999985
cg1909603	-0.09842	0.037269	-2.64074	0.009248	0.999985

cg0117651	-0.19243	0.072873	-2.64056	0.009252	0.999985
cg1500174	-0.06618	0.025063	-2.64051	0.009254	0.999985
cg0365427	-0.09633	0.036482	-2.64051	0.009254	0.999985
cg0292192	0.054598	0.020677	2.640504	0.009254	0.999985
cg1885510	-0.04775	0.018082	-2.64048	0.009254	0.999985
cg0828487	0.130803	0.049544	2.640144	0.009263	0.999985
cg0840047	0.108125	0.040955	2.640108	0.009264	0.999985
cg0207522	0.09466	0.035856	2.640011	0.009267	0.999985
cg0380898	0.897815	0.34008	2.640007	0.009267	0.999985
cg0765118	0.192529	0.07293	2.639902	0.00927	0.999985
cg0416238	0.093483	0.035412	2.639899	0.00927	0.999985
cg0997526	-0.09823	0.037214	-2.63967	0.009276	0.999985
cg0251649	-0.08526	0.032301	-2.63951	0.00928	0.999985
cg0559240	-0.07231	0.027395	-2.63949	0.00928	0.999985
cg0832582	-0.1085	0.041106	-2.63942	0.009282	0.999985
cg0729898	-0.28802	0.109125	-2.63932	0.009285	0.999985
cg2074803	-0.09744	0.036921	-2.63915	0.009289	0.999985
cg1873433	0.099722	0.03779	2.638849	0.009297	0.999985
cg0917924	-0.10059	0.038122	-2.6386	0.009303	0.999985
cg1805254	0.874001	0.331254	2.638462	0.009307	0.999985
cg2430904	0.197302	0.074785	2.638254	0.009312	0.999985
cg1554245	-0.09882	0.037457	-2.63818	0.009314	0.999985
cg2453011	-0.19456	0.073749	-2.63814	0.009316	0.999985
cg0195771	0.357598	0.135553	2.638058	0.009318	0.999985
cg2270881	0.086584	0.032821	2.638042	0.009318	0.999985
cg0209065	-0.09651	0.036585	-2.63794	0.009321	0.999985
cg2032996	0.07573	0.028713	2.637527	0.009331	0.999985
cg2619588	0.15806	0.059928	2.637485	0.009333	0.999985
cg1733991	0.121391	0.046028	2.637315	0.009337	0.999985
cg2484444	0.078499	0.029765	2.637311	0.009337	0.999985
cg1738769	-0.12517	0.047463	-2.63725	0.009339	0.999985
cg1352364	0.12077	0.045801	2.636871	0.009349	0.999985
cg0621541	0.108312	0.041084	2.636368	0.009362	0.999985
cg0794162	0.455003	0.172612	2.635986	0.009372	0.999985
cg1315017	-0.09165	0.034769	-2.63589	0.009374	0.999985
cg0641887	-0.10045	0.03811	-2.63579	0.009377	0.999985
cg2607434	0.136909	0.051942	2.63579	0.009377	0.999985
cg0626975	-0.10534	0.039968	-2.63564	0.009381	0.999985
cg0019872	0.084232	0.031959	2.635613	0.009382	0.999985
cg0733844	0.109836	0.041676	2.63546	0.009386	0.999985
cg2365027	-0.10822	0.041069	-2.63505	0.009396	0.999985
cg0876291	0.086054	0.032658	2.635023	0.009397	0.999985
cg1974723	-0.13213	0.050146	-2.63498	0.009398	0.999985
cg0745668	0.114536	0.043473	2.63465	0.009407	0.999985
cg1765279	-0.21688	0.082325	-2.63444	0.009413	0.999985
cg1736641	0.092231	0.03501	2.634425	0.009413	0.999985
cg1142284	0.087361	0.033162	2.634365	0.009414	0.999985
cg0522998	-0.14078	0.053449	-2.63395	0.009425	0.999985
cg0093051	-0.10297	0.039093	-2.63392	0.009426	0.999985
cg1608487	-0.06027	0.022884	-2.6339	0.009427	0.999985

cg2273700	-0.13082	0.04967	-2.63385	0.009428	0.999985
cg2723600	-0.10156	0.038563	-2.63371	0.009432	0.999985
cg0652114	0.104285	0.039599	2.633557	0.009436	0.999985
cg0761430	-0.10053	0.038174	-2.63355	0.009436	0.999985
cg0395934	-0.06119	0.023237	-2.63323	0.009444	0.999985
cg2393500	0.10302	0.039127	2.632981	0.009451	0.999985
cg1556275	-0.13773	0.05231	-2.63297	0.009451	0.999985
cg0753823	-0.13867	0.052671	-2.63279	0.009456	0.999985
cg1164229	-0.07194	0.027328	-2.63248	0.009464	0.999985
cg2550513	-0.10749	0.040835	-2.63225	0.00947	0.999985
cg2295940	-0.10121	0.038449	-2.63225	0.00947	0.999985
cg0946280	0.161605	0.061396	2.632157	0.009473	0.999985
cg0824831	-0.09217	0.035018	-2.63198	0.009478	0.999985
cg1249159	0.15222	0.057837	2.631898	0.00948	0.999985
cg2555645	-0.10909	0.041451	-2.63183	0.009481	0.999985
cg0885502	-0.07132	0.0271	-2.63178	0.009483	0.999985
cg1850569	0.533035	0.202543	2.631718	0.009484	0.999985
cg0271915	-0.11895	0.0452	-2.63157	0.009488	0.999985
cg0652713	0.08642	0.032841	2.631447	0.009492	0.999985
cg0909298	-0.09943	0.037788	-2.63129	0.009496	0.999985
cg1275533	0.115751	0.043991	2.631242	0.009497	0.999985
cg1956156	-0.10417	0.039593	-2.6311	0.009501	0.999985
cg0731501	0.126533	0.048091	2.631095	0.009501	0.999985
cg1709939	0.091063	0.03461	2.631091	0.009501	0.999985
cg1016944	-0.14852	0.056451	-2.63103	0.009503	0.999985
cg2515786	-0.06383	0.024261	-2.63098	0.009504	0.999985
cg0010216	0.239411	0.090997	2.63096	0.009504	0.999985
cg0188177	0.099189	0.037702	2.630881	0.009507	0.999985
cg2088899	0.0928	0.035275	2.630757	0.00951	0.999985
cg1447570	0.131649	0.050046	2.630546	0.009516	0.999985
cg1488279	0.149578	0.056871	2.630128	0.009527	0.999985
cg0474835	0.104937	0.039899	2.630056	0.009529	0.999985
cg0248412	0.134807	0.051256	2.630047	0.009529	0.999985
cg2394845	0.087235	0.03317	2.629954	0.009531	0.999985
cg0924136	-0.07107	0.027025	-2.62995	0.009531	0.999985
cg1773904	0.139575	0.053072	2.62992	0.009532	0.999985
cg2136334	-0.14174	0.053895	-2.62985	0.009534	0.999985
cg1240369	-0.10659	0.040533	-2.62983	0.009535	0.999985
cg0104469	-0.07837	0.029801	-2.62975	0.009537	0.999985
cg0500744	-0.21666	0.08239	-2.62973	0.009537	0.999985
cg1910559	0.081606	0.031033	2.629628	0.00954	0.999985
cg0233614	-0.07546	0.028696	-2.62962	0.00954	0.999985
cg1191741	-0.11147	0.042392	-2.62953	0.009542	0.999985
cg1128267	0.155253	0.059043	2.629502	0.009543	0.999985
cg1482382	0.111588	0.04244	2.629308	0.009548	0.999985
cg1727514	-0.15697	0.059702	-2.62917	0.009552	0.999985
cg1577331	0.136447	0.0519	2.629015	0.009556	0.999985
cg1053820	-0.14646	0.055713	-2.62889	0.00956	0.999985
cg2088832	-0.10306	0.039205	-2.62864	0.009566	0.999985
cg0031977	-0.10185	0.038748	-2.62853	0.009569	0.999985

cg2272953	0.104511	0.039767	2.628106	0.009581	0.999985
cg2308263	0.100516	0.038247	2.628083	0.009581	0.999985
cg0732641	-0.09453	0.03597	-2.62801	0.009583	0.999985
cg0527068	-0.07837	0.029826	-2.62777	0.00959	0.999985
cg1766423	0.11024	0.041959	2.62733	0.009601	0.999985
cg0640804	0.120881	0.046016	2.626939	0.009612	0.999985
cg0610063	0.054367	0.020698	2.626608	0.009621	0.999985
cg0834455	0.085676	0.032618	2.626607	0.009621	0.999985
cg1823505	0.477607	0.181846	2.626436	0.009625	0.999985
cg1940796	0.167816	0.063896	2.626408	0.009626	0.999985
cg1310471	-0.07213	0.027464	-2.6263	0.009629	0.999985
cg0802426	0.718355	0.273539	2.626153	0.009633	0.999985
cg1429361	-0.12162	0.046312	-2.62614	0.009633	0.999985
cg2091040	0.120926	0.046048	2.626091	0.009635	0.999985
cg2415587	-0.12304	0.046853	-2.62603	0.009636	0.999985
cg0861713	-0.10578	0.04028	-2.62601	0.009637	0.999985
cg2007806	0.132438	0.050436	2.62588	0.00964	0.999985
cg1822426	-0.1192	0.045394	-2.62585	0.009641	0.999985
cg1830467	-0.06708	0.025548	-2.62573	0.009644	0.999985
cg0726616	-0.09537	0.036329	-2.6253	0.009656	0.999985
cg2744441	-0.09633	0.036693	-2.6252	0.009659	0.999985
cg1930182	-0.08307	0.031645	-2.62496	0.009665	0.999985
cg1011644	-0.09546	0.036366	-2.62489	0.009667	0.999985
cg1766208	-0.09743	0.03712	-2.62481	0.009669	0.999985
cg1921370	-0.10637	0.040526	-2.62466	0.009673	0.999985
cg0332155	-0.05578	0.021251	-2.62462	0.009674	0.999985
cg0947001	0.073324	0.027937	2.624581	0.009675	0.999985
cg1794243	0.068095	0.025949	2.624177	0.009686	0.999985
cg0563864	0.674742	0.257139	2.624031	0.00969	0.999985
cg0110199	0.157621	0.060071	2.623928	0.009693	0.999985
cg1959289	-0.12649	0.048212	-2.62364	0.009701	0.999985
cg2682724	0.245045	0.093401	2.623569	0.009703	0.999985
cg1528942	-0.22612	0.086198	-2.62331	0.00971	0.999985
cg0314905	-0.07764	0.0296	-2.6231	0.009715	0.999985
cg2599683	0.239948	0.09148	2.622964	0.009719	0.999985
cg2167939	0.252591	0.096301	2.622934	0.00972	0.999985
cg1780507	-0.10114	0.038562	-2.62288	0.009721	0.999985
cg0155445	-0.10454	0.039858	-2.6227	0.009726	0.999985
cg2144165	-0.07653	0.029182	-2.62261	0.009729	0.999985
cg0354623	-0.05568	0.021231	-2.62258	0.00973	0.999985
cg1356061	-0.10344	0.039446	-2.62222	0.009739	0.999985
cg0564349	-0.05482	0.020909	-2.62166	0.009754	0.999985
cg2751410	0.200758	0.076577	2.621642	0.009755	0.999985
cg1507316	-0.1463	0.055806	-2.62161	0.009756	0.999985
cg1206420	0.133915	0.051085	2.621389	0.009762	0.999985
cg1061381	0.191322	0.072985	2.621388	0.009762	0.999985
cg1102687	0.112392	0.042877	2.621281	0.009765	0.999985
cg1575524	-0.08453	0.03225	-2.62114	0.009769	0.999985
cg2022486	-0.08777	0.033487	-2.621	0.009772	0.999985
cg1407394	-0.0668	0.025487	-2.62073	0.00978	0.999985

cg1727286	-0.08172	0.031187	-2.62044	0.009788	0.999985
cg0936562	-0.0757	0.028891	-2.62022	0.009794	0.999985
cg0090980	-0.24037	0.091744	-2.62007	0.009798	0.999985
cg1713669	-0.09379	0.035797	-2.62005	0.009798	0.999985
cg0481891	0.136934	0.052264	2.620044	0.009799	0.999985
cg1902498	0.175863	0.067123	2.620034	0.009799	0.999985
cg1788484	-0.05789	0.022097	-2.61997	0.009801	0.999985
cg2027357	-0.82974	0.316704	-2.61992	0.009802	0.999985
cg1738624	0.684255	0.261179	2.619867	0.009803	0.999985
cg0966101	-0.08867	0.033851	-2.61936	0.009817	0.999985
cg2109494	-0.08207	0.031337	-2.61904	0.009826	0.999985
cg1421822	-0.09123	0.034836	-2.61899	0.009827	0.999985
cg1504669	-0.10311	0.03937	-2.61897	0.009828	0.999985
cg2493565	0.09682	0.03697	2.618863	0.009831	0.999985
cg1295112	-0.08688	0.033179	-2.61863	0.009837	0.999985
cg1610728	-0.05956	0.022748	-2.61838	0.009844	0.999985
cg1397613	-0.08709	0.033259	-2.61838	0.009844	0.999985
cg0693306	-0.08442	0.032241	-2.61835	0.009845	0.999985
cg2034278	-0.06488	0.024778	-2.61833	0.009846	0.999985
cg2618881	0.165664	0.063273	2.618253	0.009848	0.999985
cg2015312	-0.1317	0.050302	-2.6181	0.009852	0.999985
cg0272069	-0.0861	0.03289	-2.61779	0.00986	0.999985
cg0097441	0.084657	0.032339	2.617769	0.009861	0.999985
cg1425895	-0.13744	0.052513	-2.61721	0.009876	0.999985
cg0375360	-0.06373	0.024353	-2.61704	0.009881	0.999985
cg2311002	-0.12736	0.048665	-2.61702	0.009881	0.999985
cg1425850	0.074204	0.028359	2.61662	0.009893	0.999985
cg0129379	-0.0897	0.03428	-2.61659	0.009893	0.999985
cg1183009	-0.35619	0.136137	-2.6164	0.009899	0.999985
cg0562702	0.2242	0.085691	2.616364	0.0099	0.999985
cg1365709	0.112366	0.042947	2.616354	0.0099	0.999985
cg1889191	0.080278	0.030684	2.616255	0.009903	0.999985
cg0068365	0.085549	0.0327	2.616202	0.009904	0.999985
cg2419467	0.200955	0.07682	2.615906	0.009912	0.999985
cg1196880	-0.17011	0.065037	-2.61563	0.00992	0.999985
cg2180154	0.07206	0.02755	2.615627	0.00992	0.999985
cg1185558	-0.10861	0.041523	-2.61559	0.009921	0.999985
cg2526688	0.108667	0.041547	2.615548	0.009922	0.999985
cg1370235	-0.0831	0.031773	-2.61549	0.009924	0.999985
cg0868804	-0.07936	0.030342	-2.61548	0.009924	0.999985
cg1846158	-0.1096	0.041908	-2.61537	0.009927	0.999985
cg0236015	0.173604	0.06638	2.615322	0.009928	0.999985
cg0566458	-0.06007	0.022969	-2.61525	0.00993	0.999985
cg2653814	0.067771	0.025914	2.615195	0.009932	0.999985
cg1352750	-0.21061	0.080537	-2.6151	0.009934	0.999985
cg1861843	0.780422	0.298429	2.615104	0.009934	0.999985
cg1103627	-0.11133	0.042574	-2.61502	0.009937	0.999985
cg2701104	0.1141	0.043634	2.614944	0.009939	0.999985
cg2392659	-0.14373	0.054967	-2.61492	0.009939	0.999985
cg2286379	0.092742	0.035469	2.614752	0.009944	0.999985

cg0790367	-0.08092	0.030949	-2.61471	0.009945	0.999985
cg0991012	-0.05807	0.022209	-2.61462	0.009948	0.999985
cg2328805	0.075829	0.029003	2.61458	0.009949	0.999985
cg0442600	-0.07136	0.027293	-2.61449	0.009951	0.999985
cg0842487	-0.0832	0.03183	-2.61389	0.009968	0.999985
cg0621962	0.087551	0.033498	2.613645	0.009975	0.999985
cg0891123	-0.06435	0.024621	-2.61364	0.009975	0.999985
cg0745003	0.095894	0.036691	2.613553	0.009977	0.999985
cg0968677	0.163449	0.062558	2.612755	0.01	0.999985
