# Supplementary material for: Epigenetic signature of preterm birth in adult twins
Source: Clin Epigenetics. 2018 Jun 27;10:87. doi: 10.1186/s13148-018-0518-8 (PMC6020425; doi:10.1186/s13148-018-0518-8)
Supplement: Supplementary file 4 — Table S2. PTB DMR with p value lower than 5%. (PDF 991 kb) [file 13148_2018_518_MOESM4_ESM.pdf]

chr	start	end	value	area	cluster	indexStart	indexEnd	L	clusterL	p.value	fwer	p.valueAre	fwerArea
chr5	1594282	1594863	-0.50464	4.541791	139197	113545	113553	9	10	4.46E-07	0.009	6.14E-06	0.121
chr3	1.12E+08	1.12E+08	1.571018	1.571018	124830	83492	83492	1	1	7.92E-07	0.016	0.00038	0.998
chr22	24384159	24384573	0.455206	3.641647	116688	408912	408919	8	8	2.28E-06	0.042	1.77E-05	0.301
chr6	291687	292596	-0.45257	3.167987	148771	133459	133465	7	7	4.26E-06	0.08	3.19E-05	0.473
chr6	41068646	41068752	-0.51187	2.559362	153202	150498	150502	5	8	4.46E-06	0.084	7.14E-05	0.757
chr5	1.35E+08	1.35E+08	0.271204	4.068053	144990	125139	125153	15	17	4.90E-06	0.098	1.04E-05	0.2
chr10	1.32E+08	1.32E+08	0.620271	1.860813	27585	236214	236216	3	3	5.40E-06	0.102	0.00022	0.975
chr6	29894005	29894228	-0.49752	2.487597	151448	140249	140253	5	29	6.29E-06	0.11	7.98E-05	0.796
chr9	82286351	82286351	1.359326	1.359326	181976	211162	211162	1	1	7.63E-06	0.136	0.000593	1
chr22	24348549	24348715	-0.593	1.779008	116682	408891	408893	3	3	8.07E-06	0.144	0.000255	0.988
chr3	1.95E+08	1.95E+08	-0.36904	2.952301	129804	93472	93479	8	8	1.13E-05	0.19	4.13E-05	0.553
chr6	30039374	30039476	0.311907	2.807159	151504	140586	140594	9	43	1.33E-05	0.227	5.05E-05	0.622
chr6	31838544	31838544	-1.30275	1.302752	151934	144528	144528	1	10	1.34E-05	0.22	0.000677	1
chr22	24373054	24373618	0.439732	2.198662	116684	408903	408907	5	14	1.53E-05	0.25	0.000123	0.901
chr11	60608291	60608291	-1.2765	1.2765	32985	249590	249590	1	1	1.69E-05	0.256	0.000722	1
chr5	1.78E+08	1.78E+08	-1.26629	1.266291	148348	132454	132454	1	1	1.86E-05	0.278	0.000742	1
chr12	378771	378771	-1.25857	1.258567	39438	264330	264330	1	1	1.94E-05	0.287	0.000756	1
chr1	2.06E+08	2.06E+08	0.400686	2.404114	15672	34934	34939	6	8	1.94E-05	0.323	9.02E-05	0.823
chr20	60500578	60500578	-1.2349	1.234904	113518	401957	401957	1	1	2.30E-05	0.33	0.000802	1
chr8	697169	697377	-0.64721	1.294422	171739	190877	190878	2	3	2.75E-05	0.41	0.000691	1
chr6	30899145	30899162	-0.64579	1.291574	151693	142478	142479	2	12	2.79E-05	0.413	0.000696	1
chr4	967324	967327	-0.63448	1.268962	130371	94906	94907	2	10	3.12E-05	0.45	0.000736	1
chr16	70808233	70808233	-1.19836	1.198363	72188	336612	336612	1	1	3.24E-05	0.432	0.000885	1
chr2	26464058	26464058	-1.18121	1.181207	97683	45434	45434	1	1	3.78E-05	0.472	0.000927	1
chr1	2.49E+08	2.49E+08	-1.17235	1.172353	18724	41122	41122	1	2	4.04E-05	0.495	0.00095	1
chr2	20870087	20871401	0.271319	2.170553	97319	44683	44690	8	8	4.70E-05	0.605	0.000128	0.913
chr6	2888212	2888212	1.150124	1.150124	149071	134101	134101	1	1	4.83E-05	0.548	0.001005	1
chr12	678960	679287	0.459424	1.378272	39468	264402	264404	3	5	4.87E-05	0.56	0.000568	1
chr12	89748726	89749033	0.299259	2.094814	44875	276443	276449	7	12	4.93E-05	0.625	0.000146	0.937
chr21	35831871	35832141	0.32512	1.950721	114694	404619	404624	6	9	5.62E-05	0.674	0.000187	0.965
chr1	1.47E+08	1.47E+08	0.322798	1.936786	11228	25251	25256	6	13	5.79E-05	0.684	0.000192	0.966
chr5	1.24E+08	1.24E+08	1.126678	1.126678	144280	123598	123598	1	1	5.87E-05	0.615	0.001074	1
chr6	32489984	32490350	0.351808	1.759039	152134	146387	146391	5	12	6.07E-05	0.685	0.000265	0.989
chr7	2764129	2764246	-0.44196	1.325893	160991	167547	167549	3	12	6.10E-05	0.649	0.00064	1
chr2	30669597	30669863	-0.44125	1.323764	98102	46449	46451	3	8	6.14E-05	0.651	0.000643	1
chr18	43305784	43305784	1.115333	1.115333	85619	369366	369366	1	3	6.58E-05	0.65	0.001112	1
chr15	65272560	65272560	1.114749	1.114749	63068	316298	316298	1	1	6.62E-05	0.651	0.001114	1
chr6	47624115	47624117	0.544566	1.089131	153968	152170	152171	2	7	7.72E-05	0.755	0.001202	1
chr1	2.02E+08	2.02E+08	-0.18949	1.705385	15041	33636	33644	9	16	8.01E-05	0.795	0.000293	0.992
chr1	2.48E+08	2.48E+08	-0.29952	1.7971	18694	41072	41077	6	11	8.08E-05	0.795	0.000245	0.985
chr19	37466940	37466940	1.088903	1.088903	91878	384906	384906	1	1	8.30E-05	0.734	0.001203	1
chr11	5617812	5618408	0.370946	1.483785	29684	242680	242683	4	11	8.43E-05	0.793	0.000454	0.999
chr10	76996280	76996533	-0.41917	1.257502	23493	227342	227344	3	8	8.49E-05	0.773	0.000758	1
chr6	34524698	34524766	0.532317	1.064633	152555	149241	149242	2	9	8.85E-05	0.8	0.001297	1
chr10	1.3E+08	1.3E+08	1.073058	1.073058	27435	235775	235775	1	5	9.47E-05	0.775	0.001264	1
chr7	1209495	1209742	-0.41077	1.232319	160389	165729	165731	3	3	9.56E-05	0.815	0.000807	1
chr12	96188933	96188933	1.06767	1.06767	45217	277043	277043	1	1	9.88E-05	0.788	0.001285	1
chr10	72647652	72647652	1.059798	1.059798	23044	226432	226432	1	2	0.000106	0.81	0.001315	1
chr7	2802697	2802942	-0.51476	1.029527	161002	167593	167594	2	6	0.000107	0.849	0.001447	1
chr16	86112141	86112141	-1.04901	1.049014	73466	339250	339250	1	4	0.000115	0.837	0.00136	1
chr5	218153	218493	-0.34187	1.367489	138695	112177	112180	4	9	0.000124	0.898	0.000582	1
chr10	1.23E+08	1.23E+08	-0.23235	1.626446	26780	234404	234410	7	9	0.000126	0.914	0.00034	0.998
chr7	4244372	4244643	-0.49637	0.992745	161156	167924	167925	2	2	0.000131	0.909	0.001627	1
chr22	24372483	24372926	0.337627	1.350508	116684	408896	408899	4	14	0.000132	0.906	0.000605	1
chr5	1.8E+08	1.8E+08	-0.29602	1.480085	148684	133211	133215	5	6	0.000136	0.92	0.000457	0.999
chr1	42843232	42843232	1.027539	1.027539	6618	15211	15211	1	1	0.000138	0.883	0.001456	1
chr11	67383377	67384040	-0.19291	1.54327	34388	253693	253700	8	8	0.000149	0.935	0.000403	0.999
chr2	2.4E+08	2.4E+08	-1.01653	1.016531	109979	70298	70298	1	1	0.00015	0.903	0.001508	1
chr6	33096303	33096312	0.472469	0.944937	152266	147280	147281	2	7	0.000167	0.953	0.001911	1
chr5	1178961	1179328	-0.36882	1.106447	139030	113081	113083	3	3	0.000174	0.948	0.001141	1
chr19	2340235	2340235	-0.99553	0.99553	87711	374172	374172	1	3	0.000176	0.932	0.001613	1
chr5	36242508	36242542	0.466647	0.933295	140726	116584	116585	2	14	0.00018	0.962	0.001994	1
chr14	1.04E+08	1.04E+08	0.315135	1.26054	59185	307961	307964	4	12	0.000182	0.96	0.000753	1
chr8	22132714	22133076	-0.20822	1.457512	173558	194479	194485	7	12	0.000182	0.973	0.000478	0.999
chr6	29723301	29723407	0.45784	0.91568	151419	140113	140114	2	2	0.000195	0.968	0.002128	1
chr1	2.21E+08	2.21E+08	0.979737	0.979737	16614	36799	36799	1	1	0.0002	0.949	0.001697	1
chr5	2864736	2864736	-0.97908	0.97908	139423	114212	114212	1	2	0.000201	0.949	0.001701	1
chr14	69341603	69341905	-0.35691	1.070724	56430	302177	302179	3	5	0.000207	0.966	0.001274	1
chr6	29706048	29706048	0.974888	0.974888	151409	140023	140023	1	5	0.000207	0.954	0.001725	1
chr7	1.48E+08	1.48E+08	-0.97348	0.973481	169931	186424	186424	1	1	0.00021	0.955	0.001735	1
chr1	4725584	4725584	-0.97021	0.970207	1463	4065	4065	1	1	0.000215	0.96	0.001754	1
chr6	29911036	29911261	0.349286	1.047857	151451	140284	140286	3	11	0.00023	0.978	0.001366	1
chr12	21594328	21594328	0.955497	0.955497	41149	267980	267980	1	1	0.000242	0.971	0.001842	1

chr16	84520021	84520021	-0.95303	0.953026	73060	338355	338355	1	1	0.000246	0.973	0.001857	1
chr17	9546550	9546550	-0.9517	0.951705	76415	346770	346770	1	1	0.000248	0.975	0.001866	1
chr7	22481962	22481962	0.951187	0.951187	162212	170292	170292	1	1	0.00025	0.975	0.001869	1
chr16	49732224	49732623	0.292826	1.171303	70623	333061	333064	4	4	0.000254	0.983	0.000953	1
chr19	33622911	33622959	-0.34198	1.025943	91381	383531	383533	3	12	0.000254	0.982	0.001463	1
chr5	1.58E+08	1.58E+08	0.946294	0.946294	146843	129306	129306	1	1	0.00026	0.976	0.001902	1
chr16	419975	420230	0.336596	1.009788	66521	323476	323478	3	9	0.000273	0.986	0.001539	1
chr11	5617273	5617703	0.286205	1.14482	29684	242675	242678	4	11	0.000279	0.987	0.001021	1
chr5	1.77E+08	1.77E+08	-0.33476	1.004292	148046	131798	131800	3	3	0.00028	0.987	0.001565	1
chr11	6744048	6744442	-0.42516	0.850317	29827	243022	243023	2	2	0.000283	0.994	0.002731	1
chr3	60420416	60420416	0.934697	0.934697	123145	80556	80556	1	1	0.000286	0.982	0.001984	1
chr12	1.32E+08	1.32E+08	0.931463	0.931463	48282	284085	284085	1	2	0.000294	0.986	0.002007	1
chr6	32301513	32301513	-0.92813	0.928133	152085	146254	146254	1	6	0.000303	0.989	0.002033	1
chr8	2122276	2122339	0.419282	0.838563	172229	192064	192065	2	2	0.000305	0.994	0.002862	1
chr10	3178915	3178915	0.926472	0.926472	19299	218917	218917	1	1	0.000306	0.989	0.002044	1
chr11	59209777	59209777	-0.92046	0.920464	32852	249329	249329	1	2	0.000321	0.991	0.002089	1
chr6	25882463	25882590	0.324411	0.973232	150760	137288	137290	3	5	0.000324	0.993	0.001736	1
chr3	1.94E+08	1.94E+08	-0.23768	1.188406	129597	93069	93073	5	8	0.000331	0.997	0.000909	1
chr8	27522576	27522576	-0.91699	0.916985	174058	195580	195580	1	1	0.000332	0.991	0.002117	1
chr1	40226045	40226045	0.915572	0.915572	6323	14564	14564	1	2	0.000336	0.991	0.002129	1
chr16	8995926	8995926	0.913779	0.913779	68277	328097	328097	1	5	0.00034	0.992	0.002144	1
chr6	1.68E+08	1.68E+08	-0.91052	0.91052	159288	162877	162877	1	1	0.000349	0.993	0.00217	1
chr7	1.58E+08	1.58E+08	0.407313	0.814626	171189	189544	189545	2	2	0.000351	0.996	0.003156	1
chr8	1.45E+08	1.45E+08	0.316894	0.950681	180169	207797	207799	3	3	0.000361	0.997	0.001872	1
chr22	23489041	23489041	-0.90604	0.906041	116590	408692	408692	1	1	0.000361	0.994	0.002206	1
chr6	29701563	29701563	-0.90497	0.904974	151407	140016	140016	1	4	0.000364	0.994	0.002216	1
chr17	6558064	6558815	-0.26829	1.073148	75876	345105	345108	4	5	0.000365	0.998	0.001264	1
chr21	15352213	15352641	0.315763	0.947288	114275	403626	403628	3	8	0.000368	0.999	0.001896	1
chr3	1.96E+08	1.96E+08	-0.31543	0.946302	129826	93529	93531	3	5	0.00037	0.999	0.001902	1
chr1	42384002	42384564	0.161446	1.130119	6580	15125	15131	7	9	0.000371	1	0.001064	1
chr1	16345207	16345207	-0.90245	0.902452	3243	7636	7636	1	7	0.000371	0.994	0.002235	1
chr6	32822565	32822911	-0.31356	0.940679	152201	146790	146792	3	59	0.000379	0.999	0.00194	1
chr7	1616229	1616229	0.899389	0.899389	160563	166280	166280	1	1	0.00038	0.994	0.00226	1
chr11	66317822	66317822	0.898082	0.898082	34128	252956	252956	1	1	0.000383	0.994	0.002271	1
chr2	85811421	85812023	0.225796	1.128982	101224	52738	52742	5	8	0.000397	0.998	0.001067	1
chr5	1.04E+08	1.04E+08	-0.89312	0.893125	143489	122141	122141	1	1	0.000398	0.995	0.002315	1
chr2	74357527	74357872	0.261937	1.047749	100745	51690	51693	4	4	0.000403	0.999	0.001366	1
chr2	731073	731561	0.261714	1.046856	95762	41576	41579	4	4	0.000404	0.999	0.00137	1
chr2	8118122	8118122	-0.88927	0.889275	96404	42977	42977	1	3	0.000408	0.995	0.002346	1
chr7	1.28E+08	1.28E+08	0.887669	0.887669	168456	183538	183538	1	1	0.000414	0.995	0.002363	1
chr6	32297806	32297822	-0.39051	0.781025	152083	146244	146245	2	4	0.000424	0.997	0.003632	1
chr8	74282812	74282931	0.306223	0.918668	176324	200272	200274	3	3	0.000426	0.999	0.002103	1
chr2	3704501	3704773	0.220229	1.101145	96199	42619	42623	5	9	0.000432	0.999	0.001159	1
chr3	52869263	52869263	-0.8819	0.881904	122668	79659	79659	1	6	0.000433	0.995	0.002416	1
chr7	36124843	36124863	0.387177	0.774355	163255	172690	172691	2	5	0.000443	0.998	0.003741	1
chr1	1.1E+08	1.1E+08	-0.25583	1.023307	10198	22983	22986	4	6	0.000443	0.999	0.001476	1
chr19	523300	523360	-0.38684	0.773682	86946	372297	372298	2	2	0.000444	0.998	0.003752	1
chr6	3255870	3255870	-0.87592	0.875921	149155	134297	134297	1	1	0.000453	0.995	0.002471	1
chr4	7246127	7246127	-0.8746	0.874604	131561	97947	97947	1	2	0.000458	0.995	0.002484	1
chr13	1.12E+08	1.12E+08	0.873065	0.873065	53066	294248	294248	1	2	0.000464	0.996	0.002499	1
chr5	42949542	42950152	0.215651	1.078257	141013	117181	117185	5	5	0.000466	1	0.001242	1
chr11	970389	970664	0.382112	0.764224	28781	239906	239907	2	2	0.000471	0.998	0.003914	1
chr11	71238205	71238205	0.868878	0.868878	35030	255290	255290	1	3	0.000479	0.996	0.00254	1
chr22	42548356	42548792	0.296303	0.888908	118143	412442	412444	3	4	0.000492	1	0.00235	1
chr10	42863068	42863594	0.129732	0.908123	21475	223242	223248	7	8	0.000499	1	0.00219	1
chr12	25454990	25454990	0.860493	0.860493	41305	268331	268331	1	1	0.000509	0.997	0.002619	1
chr11	1413093	1413315	0.246746	0.986984	28940	240408	240411	4	4	0.00051	1	0.001657	1
chr15	60706369	60706369	0.859624	0.859624	62642	315487	315487	1	1	0.000512	0.997	0.002629	1
chr19	12876846	12877188	-0.375	0.749991	89762	379351	379352	2	2	0.000514	0.999	0.004168	1
chr21	36259241	36259797	0.244762	0.979049	114721	404688	404691	4	4	0.000527	1	0.001701	1
chr7	73245103	73245178	-0.3722	0.744395	165389	176853	176854	2	8	0.000532	0.999	0.004275	1
chr16	55866522	55867072	-0.24375	0.975007	70995	333861	333864	4	4	0.000535	1	0.001725	1
chr8	22266134	22266134	0.853855	0.853855	173570	194514	194514	1	4	0.000537	0.997	0.002692	1
chr15	67356310	67356942	-0.24306	0.972236	63333	316837	316840	4	5	0.00054	1	0.001741	1
chr16	857454	858205	-0.20574	1.028713	66731	324150	324154	5	6	0.000548	1	0.001451	1
chr1	17394374	17394374	0.847504	0.847504	3461	8103	8103	1	1	0.000563	0.998	0.002762	1
chr6	30039013	30039132	0.202904	1.014519	151504	140580	140584	5	43	0.000574	1	0.001517	1
chr3	1.68E+08	1.68E+08	-0.84478	0.844775	128033	90044	90044	1	1	0.000576	0.998	0.002792	1
chr8	1496732	1497307	-0.23839	0.953546	172027	191535	191538	4	11	0.000583	1	0.001854	1
chr8	1.45E+08	1.45E+08	0.842594	0.842594	180187	207848	207848	1	1	0.000585	0.998	0.002816	1
chr6	6894084	6894163	-0.35995	0.719902	149471	134911	134912	2	2	0.000618	1	0.004784	1
chr17	12562530	12562530	0.835718	0.835718	76612	347150	347150	1	1	0.000619	0.999	0.002896	1
chr1	95088560	95088560	0.833961	0.833961	9684	21854	21854	1	1	0.000627	0.999	0.002914	1
chr6	32526021	32526263	0.278798	0.836394	152144	146411	146413	3	6	0.000637	1	0.002887	1

chr12	7781004	7781431	0.196392	0.98196	40312	266361	266365	5	5	0.000638	1	0.001684	1
chr14	45431685	45432516	0.196287	0.981436	55050	299343	299347	5	15	0.000639	1	0.001687	1
chr3	1.97E+08	1.97E+08	-0.19525	0.976269	129989	93914	93918	5	5	0.000649	1	0.001717	1
chr2	97004840	97004840	0.828962	0.828962	101689	53758	53758	1	1	0.000651	0.999	0.002973	1
chr8	61197710	61197710	0.828286	0.828286	175743	199011	199011	1	1	0.000654	0.999	0.002981	1
chr2	2.41E+08	2.41E+08	-0.27691	0.830733	110134	70649	70651	3	24	0.000655	1	0.002951	1
chr5	74907694	74908170	-0.23112	0.924472	142331	119847	119850	4	7	0.000657	1	0.002058	1
chr16	89883212	89883248	0.274646	0.823937	74641	342076	342078	3	12	0.000678	1	0.003035	1
chr14	59947673	59947673	0.822511	0.822511	55846	300943	300943	1	1	0.000683	0.999	0.003053	1
chr14	21156471	21156471	-0.82187	0.821865	53955	296745	296745	1	2	0.000686	0.999	0.003062	1
chr6	32493873	32493994	0.272929	0.818786	152136	146395	146397	3	3	0.000696	1	0.003101	1
chr2	2.28E+08	2.28E+08	-0.81871	0.818709	108658	67528	67528	1	1	0.000702	0.999	0.003101	1
chr10	1.32E+08	1.32E+08	-0.81854	0.818539	27582	236205	236205	1	9	0.000703	0.999	0.003104	1
chr17	14936230	14936230	-0.81783	0.817834	76736	347404	347404	1	1	0.000706	0.999	0.003114	1
chr5	9547468	9548098	0.226303	0.905211	139786	114977	114980	4	4	0.000712	1	0.002214	1
chr7	16462463	16462463	0.815524	0.815524	161927	169733	169733	1	1	0.00072	0.999	0.003145	1
chr2	77235218	77235218	-0.81473	0.814726	100957	52232	52232	1	1	0.000724	0.999	0.003155	1
chr11	1.03E+08	1.03E+08	-0.81195	0.811953	36794	258744	258744	1	1	0.000739	0.999	0.003191	1
chr11	1.34E+08	1.34E+08	0.268784	0.806353	39237	263862	263864	3	9	0.000741	1	0.003264	1
chr14	1.06E+08	1.06E+08	0.344964	0.689928	59835	309516	309517	2	2	0.000742	1	0.005506	1
chr1	17309539	17309539	-0.80974	0.809737	3445	8065	8065	1	1	0.000753	0.999	0.003221	1
chr15	68594860	68594860	-0.80941	0.809408	63459	317105	317105	1	2	0.000755	0.999	0.003225	1
chr21	15352940	15352983	0.267338	0.802014	114275	403630	403632	3	8	0.000757	1	0.003324	1
chr10	4868328	4868398	-0.14259	0.855543	19470	219230	219235	6	13	0.000761	1	0.002673	1
chr4	125112	125504	0.342898	0.685796	130142	94235	94236	2	6	0.000761	1	0.005618	1
chr1	2.14E+08	2.14E+08	-0.80611	0.806107	16294	36225	36225	1	1	0.000773	1	0.003268	1
chr5	1.71E+08	1.71E+08	-0.22001	0.880042	147389	130385	130388	4	4	0.000788	1	0.002431	1
chr11	6592066	6592745	-0.21997	0.879861	29800	242947	242950	4	5	0.000788	1	0.002433	1
chr4	2403781	2404284	-0.26487	0.794618	130805	96169	96171	3	3	0.000789	1	0.003425	1
chr7	64450926	64451426	-0.33983	0.67965	164882	175848	175849	2	4	0.000791	1	0.005787	1
chr22	25202037	25202086	0.264175	0.792524	116772	409121	409123	3	10	0.000796	1	0.003455	1
chr2	2.39E+08	2.39E+08	-0.21906	0.876224	109763	69791	69794	4	5	0.000801	1	0.002468	1
chr4	1.84E+08	1.84E+08	0.799973	0.799973	138002	110762	110762	1	1	0.000808	1	0.00335	1
chr14	1.06E+08	1.06E+08	-0.13587	0.81521	59796	309445	309450	6	6	0.00081	1	0.003149	1
chr19	18699118	18699342	0.181063	0.905315	90776	382006	382010	5	12	0.000819	1	0.002213	1
chr6	32376066	32376095	-0.33572	0.671434	152114	146340	146341	2	10	0.000831	1	0.006016	1
chr13	42704154	42704154	0.795914	0.795914	50454	289096	289096	1	1	0.000833	1	0.003408	1
chr7	75372874	75372874	0.794849	0.794849	165595	177253	177253	1	1	0.000839	1	0.003422	1
chr7	3157722	3157722	0.794427	0.794427	161056	167719	167719	1	1	0.000841	1	0.003428	1
chr3	53032818	53033167	-0.1782	0.891008	122683	79690	79694	5	6	0.000858	1	0.002332	1
chr6	1.57E+08	1.57E+08	-0.79162	0.791617	158405	161022	161022	1	1	0.000858	1	0.003469	1
chr11	97844307	97844307	0.7915	0.7915	36620	258430	258430	1	1	0.000859	1	0.003471	1
chr4	96760945	96760945	-0.79132	0.791321	134971	104782	104782	1	10	0.00086	1	0.003473	1
chr1	67600547	67600835	0.214581	0.858324	8561	19433	19436	4	8	0.000864	1	0.002642	1
chr2	1.4E+08	1.4E+08	0.176679	0.883395	104346	58956	58960	5	13	0.000879	1	0.002402	1
chr21	38630234	38630728	0.213408	0.853634	114842	405008	405011	4	5	0.000881	1	0.002694	1
chr5	1.53E+08	1.53E+08	0.788114	0.788114	146550	128756	128756	1	1	0.000881	1	0.003522	1
chr1	1.56E+08	1.56E+08	0.787543	0.787543	12368	28196	28196	1	1	0.000884	1	0.00353	1
chr17	40819808	40819808	-0.78691	0.786914	79467	353776	353776	1	2	0.000888	1	0.003539	1
chr7	51293912	51293912	-0.78678	0.786778	164318	174757	174757	1	1	0.000889	1	0.003541	1
chr6	32305068	32305145	-0.25596	0.767884	152088	146260	146262	3	3	0.000907	1	0.003852	1
chr7	1588284	1588396	0.255903	0.767708	160548	166227	166229	3	4	0.000908	1	0.003855	1
chr17	79905236	79905263	0.255416	0.766248	84161	365517	365519	3	7	0.000916	1	0.003881	1
chr10	839609	839609	-0.78226	0.78226	18980	218160	218160	1	1	0.000919	1	0.003611	1
chr10	63240299	63240299	-0.78105	0.781052	22503	225278	225278	1	1	0.000928	1	0.003631	1
chr15	66947564	66947617	0.254445	0.763335	63280	316744	316746	3	3	0.00093	1	0.003931	1
chr7	63385990	63386815	-0.20979	0.839152	164788	175633	175636	4	6	0.000937	1	0.002855	1
chr17	4804374	4804838	-0.32565	0.651292	75695	344665	344666	2	2	0.000942	1	0.006651	1
chr14	50474530	50474530	0.778395	0.778395	55161	299622	299622	1	6	0.000946	1	0.003674	1
chr2	2.42E+08	2.42E+08	-0.20868	0.834725	110242	70905	70908	4	5	0.000954	1	0.002907	1
chr20	32856846	32857151	0.208476	0.833903	112032	398018	398021	4	5	0.000957	1	0.002915	1
chr1	1.53E+08	1.53E+08	-0.77673	0.776731	11861	26787	26787	1	2	0.000958	1	0.003702	1
chr14	73719302	73719302	-0.77658	0.776581	56810	302872	302872	1	1	0.000959	1	0.003705	1
chr22	17591088	17591088	0.774116	0.774116	115980	407306	407306	1	1	0.000976	1	0.003745	1
chr8	91681699	91681699	-0.77351	0.773513	176885	201360	201360	1	1	0.000981	1	0.003755	1
chr12	1.32E+08	1.32E+08	0.773082	0.773082	48249	284012	284012	1	2	0.000984	1	0.003763	1
chr13	31585928	31585928	-0.77272	0.772721	49903	287954	287954	1	1	0.000987	1	0.003768	1
chr5	164064	164172	-0.32185	0.643696	138672	112107	112108	2	3	0.00099	1	0.00691	1
chr7	94953956	94954202	-0.20645	0.825803	166454	179132	179135	4	11	0.000991	1	0.003012	1
chr17	75238703	75238948	-0.25027	0.750812	82975	362211	362213	3	3	0.000995	1	0.004152	1
chr6	32489203	32489555	0.250171	0.750514	152134	146382	146384	3	12	0.000996	1	0.004157	1
chr7	94023308	94023796	-0.20604	0.824155	166415	178947	178950	4	9	0.000999	1	0.003032	1
chr17	77706946	77707066	0.249339	0.748017	83439	363319	363321	3	4	0.001009	1	0.004205	1
chr3	3080327	3080338	-0.31895	0.63791	119171	71983	71984	2	4	0.001028	1	0.007114	1

chr15	28644585	28644585	0.766717	0.766717	60523	310999	310999	1	1	0.001032	1	0.003873	1
chr19	940724	941243	0.204068	0.816272	87135	372738	372741	4	4	0.001033	1	0.003134	1
chr11	1.18E+08	1.18E+08	-0.76521	0.765206	37627	260567	260567	1	2	0.001042	1	0.003898	1
chr10	50328538	50328538	-0.76517	0.765167	22044	224391	224391	1	2	0.001043	1	0.003899	1
chr2	1.09E+08	1.09E+08	-0.20348	0.813937	102523	55452	55455	4	5	0.001044	1	0.003165	1
chr2	2.33E+08	2.33E+08	0.316319	0.632638	109044	68326	68327	2	4	0.001064	1	0.007302	1
chr7	786861	786861	-0.76231	0.762315	160167	165040	165040	1	6	0.001064	1	0.003948	1
chr1	2120985	2121521	0.201604	0.806417	668	1886	1889	4	5	0.001077	1	0.003264	1
chr2	2.03E+08	2.03E+08	0.163571	0.817853	107118	64488	64492	5	5	0.001084	1	0.003113	1
chr17	7253189	7253720	-0.20066	0.802637	76003	345573	345576	4	4	0.001095	1	0.003315	1
chr17	75084281	75084281	0.757126	0.757126	82951	362136	362136	1	11	0.001103	1	0.004038	1
chr7	29605624	29606349	-0.16255	0.812759	162794	171840	171844	5	18	0.001103	1	0.003181	1
chr11	638076	639423	0.20004	0.800159	28653	239532	239535	4	4	0.001107	1	0.003348	1
chr17	17108846	17109678	0.162192	0.810961	76964	347861	347865	5	13	0.00111	1	0.003204	1
chr7	64043012	64043119	0.312769	0.625538	164841	175759	175760	2	2	0.001114	1	0.007569	1
chr18	77917615	77918142	-0.24281	0.728435	86842	372055	372057	3	3	0.001117	1	0.004601	1
chr19	39265241	39265241	0.754572	0.754572	92101	385470	385470	1	4	0.001122	1	0.004081	1
chr6	8435968	8436263	0.199134	0.796534	149630	135225	135228	4	14	0.001126	1	0.003399	1
chr5	1.8E+08	1.8E+08	0.198933	0.79573	148590	132979	132982	4	4	0.001129	1	0.00341	1
chr16	18478530	18478530	-0.75187	0.751869	68904	329217	329217	1	2	0.001144	1	0.004131	1
chr4	2402019	2402019	-0.75143	0.751428	130804	96167	96167	1	5	0.001148	1	0.00414	1
chr8	1.41E+08	1.41E+08	0.749407	0.749407	179281	205603	205603	1	4	0.001166	1	0.004178	1
chr1	86888585	86888629	-0.23968	0.719038	9213	20768	20770	3	4	0.001178	1	0.004804	1
chr2	1.01E+08	1.01E+08	0.747889	0.747889	102027	54529	54529	1	4	0.00118	1	0.004206	1
chr3	13555664	13555664	0.747155	0.747155	119917	73584	73584	1	1	0.001185	1	0.004219	1
chr5	65888284	65888284	-0.74695	0.746948	141856	118883	118883	1	1	0.001187	1	0.004222	1
chr12	9436616	9437009	-0.23904	0.717135	40490	266733	266735	3	6	0.001189	1	0.004848	1
chr8	8761750	8761750	-0.74647	0.746469	172612	192675	192675	1	1	0.001192	1	0.004232	1
chr9	96623032	96623674	0.195424	0.781697	182523	211980	211983	4	4	0.0012	1	0.00362	1
chr5	68710813	68710912	0.156798	0.783991	141989	119148	119152	5	10	0.001203	1	0.003584	1
chr2	98206702	98206733	-0.30684	0.613683	101798	54041	54042	2	4	0.00121	1	0.008036	1
chr5	54117761	54117761	-0.74157	0.741566	141307	117843	117843	1	1	0.00124	1	0.00433	1
chr15	1.01E+08	1.01E+08	0.304964	0.609927	66206	322797	322798	2	2	0.001242	1	0.008196	1
chr5	10091925	10091925	-0.73995	0.739954	139806	115001	115001	1	1	0.001254	1	0.00436	1
chr13	23412250	23412409	0.23578	0.707341	49235	286521	286523	3	4	0.001256	1	0.005072	1
chr15	81666528	81666528	0.738925	0.738925	64854	319904	319904	1	2	0.001264	1	0.004382	1
chr6	1.71E+08	1.71E+08	0.153317	0.766586	159897	164317	164321	5	5	0.001267	1	0.003875	1
chr1	46420220	46420220	0.738147	0.738147	7087	16384	16384	1	1	0.001271	1	0.004398	1
chr2	1165351	1165351	-0.73592	0.735923	95818	41700	41700	1	1	0.001293	1	0.004445	1
chr5	31106255	31106255	0.735689	0.735689	140412	115996	115996	1	1	0.001295	1	0.004449	1
chr5	421317	421733	0.190971	0.763882	138762	112335	112338	4	8	0.001302	1	0.003921	1
chr6	1.53E+08	1.53E+08	0.730773	0.730773	158215	160737	160737	1	1	0.001342	1	0.00455	1
chr10	1.32E+08	1.32E+08	-0.18922	0.756884	27639	236413	236416	4	8	0.001344	1	0.004043	1
chr3	32509018	32509105	0.189006	0.756026	120822	75203	75206	4	8	0.001349	1	0.004057	1
chr6	41374532	41374939	0.230674	0.692021	153236	150587	150589	3	4	0.001367	1	0.005454	1
chr3	1.95E+08	1.95E+08	-0.29733	0.59466	129704	93301	93302	2	3	0.001375	1	0.008871	1
chr14	99728984	99729088	-0.22998	0.689945	58509	306271	306273	3	3	0.001383	1	0.005506	1
chr2	10942687	10942687	-0.72648	0.72648	96751	43646	43646	1	2	0.001385	1	0.00464	1
chr12	2690385	2690385	-0.72627	0.726272	39704	264828	264828	1	1	0.001386	1	0.004644	1
chr8	1.31E+08	1.31E+08	0.723929	0.723929	178844	204924	204924	1	1	0.001412	1	0.004696	1
chr7	27187502	27187560	0.186101	0.744405	162556	171209	171212	4	15	0.001418	1	0.004275	1
chr17	5025970	5026574	0.144895	0.724476	75748	344812	344816	5	10	0.001424	1	0.004684	1
chr4	124232	124342	0.294244	0.588487	130142	94231	94232	2	6	0.001432	1	0.00916	1
chr8	2075469	2075777	0.294097	0.588194	172221	192049	192050	2	2	0.001435	1	0.009174	1
chr8	58055591	58056113	-0.18509	0.740341	175620	198781	198784	4	7	0.001444	1	0.004353	1
chr6	26195910	26195995	0.227517	0.682551	150820	137518	137520	3	4	0.001445	1	0.005708	1
chr11	18433554	18434015	0.143326	0.71663	30817	245001	245005	5	5	0.001451	1	0.004858	1
chr6	1.7E+08	1.7E+08	-0.18478	0.739135	159758	163970	163973	4	6	0.001452	1	0.004378	1
chr1	1.62E+08	1.62E+08	-0.71984	0.719844	13065	29788	29788	1	1	0.001457	1	0.004786	1
chr5	1.57E+08	1.57E+08	-0.22621	0.678641	146799	129221	129223	3	11	0.001476	1	0.005815	1
chr4	39531927	39531927	0.717522	0.717522	132891	100535	100535	1	1	0.001484	1	0.00484	1
chr8	914818	914818	0.716574	0.716574	171805	191038	191038	1	6	0.001494	1	0.004859	1
chr5	1.1E+08	1.1E+08	-0.14069	0.703456	143655	122383	122387	5	6	0.001497	1	0.005167	1
chr11	33097335	33097335	0.715945	0.715945	31394	246371	246371	1	3	0.001501	1	0.004873	1
chr5	28928346	28928735	-0.29055	0.581095	140388	115970	115971	2	3	0.001504	1	0.009519	1
chr18	34325827	34325827	0.714769	0.714769	85527	369156	369156	1	2	0.001513	1	0.0049	1
chr11	1.32E+08	1.32E+08	-0.22447	0.673424	39071	263569	263571	3	3	0.001517	1	0.005957	1
chr16	4560300	4560300	-0.71414	0.71414	67999	327521	327521	1	1	0.00152	1	0.004914	1
chr1	1.58E+08	1.58E+08	-0.7141	0.714101	12677	28904	28904	1	2	0.001521	1	0.004915	1
chr15	67228722	67228986	0.224191	0.672572	63319	316818	316820	3	3	0.001525	1	0.005983	1
chr16	55794456	55794910	-0.22414	0.67242	70991	333853	333855	3	4	0.001526	1	0.005987	1
chr7	1.43E+08	1.43E+08	0.289365	0.57873	169751	186090	186091	2	2	0.001529	1	0.009643	1
chr11	17825098	17825098	0.71239	0.71239	30741	244848	244848	1	1	0.001539	1	0.004954	1
chr12	740100	740338	-0.28852	0.577034	39478	264418	264419	2	2	0.001547	1	0.009727	1

chr3	1.6E+08	1.6E+08	0.223289	0.669868	127836	89646	89648	3	3	0.001549	1	0.006063	1
chr16	87682036	87682142	-0.28835	0.576693	73782	339941	339942	2	6	0.00155	1	0.009744	1
chr11	325915	325964	-0.2875	0.574997	28507	239076	239077	2	9	0.001567	1	0.009831	1
chr1	92012408	92012736	-0.18055	0.722185	9473	21348	21351	4	8	0.001568	1	0.004734	1
chr1	2.02E+08	2.02E+08	-0.18023	0.720925	15139	33848	33851	4	5	0.001575	1	0.004762	1
chr16	4506231	4506231	-0.70728	0.707282	67988	327494	327494	1	1	0.001595	1	0.005073	1
chr13	79234251	79234435	0.221279	0.663837	51785	291682	291684	3	5	0.001602	1	0.006244	1
chr11	93063662	93063684	-0.28522	0.570434	36339	257895	257896	2	13	0.001617	1	0.010071	1
chr7	1.59E+08	1.59E+08	0.178669	0.714677	171526	190383	190386	4	4	0.001621	1	0.004901	1
chr8	6913030	6913030	-0.70489	0.704886	172409	192386	192386	1	2	0.001624	1	0.005129	1
chr1	22191453	22191453	-0.70459	0.704586	4117	9407	9407	1	6	0.001627	1	0.005136	1
chr20	17680544	17680544	-0.70431	0.704314	111387	396359	396359	1	1	0.00163	1	0.005143	1
chr1	3659656	3659656	-0.70417	0.704166	1348	3774	3774	1	2	0.001633	1	0.005147	1
chr1	2.37E+08	2.37E+08	-0.13067	0.653337	17998	39686	39690	5	12	0.001645	1	0.006582	1
chr19	646890	646890	0.702723	0.702723	87008	372424	372424	1	7	0.001651	1	0.005184	1
chr9	34370835	34371380	-0.21934	0.658035	181219	209959	209961	3	5	0.001657	1	0.006427	1
chr5	36273196	36273196	0.702107	0.702107	140730	116589	116589	1	1	0.001659	1	0.0052	1
chr1	1.59E+08	1.59E+08	0.282895	0.565789	12748	29015	29016	2	2	0.001667	1	0.010317	1
chr11	1463541	1463662	-0.17708	0.708327	28956	240452	240455	4	8	0.001667	1	0.005047	1
chr7	63360775	63361361	-0.12877	0.643844	164784	175622	175626	5	7	0.001669	1	0.006905	1
chr1	1374310	1374601	-0.28247	0.564949	350	1044	1045	2	2	0.001677	1	0.010366	1
chr17	6904263	6904263	0.700544	0.700544	75903	345199	345199	1	1	0.001678	1	0.005238	1
chr2	1.98E+08	1.98E+08	-0.21863	0.655889	106823	63821	63823	3	15	0.001679	1	0.006496	1
chr6	1.58E+08	1.58E+08	0.126966	0.634832	158476	161136	161140	5	6	0.00169	1	0.007222	1
chr7	1.59E+08	1.59E+08	-0.28157	0.563136	171550	190452	190453	2	2	0.001698	1	0.010469	1
chr2	2.37E+08	2.37E+08	-0.28092	0.561846	109427	69101	69102	2	10	0.001714	1	0.010542	1
chr6	30882694	30883074	-0.1235	0.617493	151688	142445	142449	5	58	0.001725	1	0.007882	1
chr17	724273	724374	-0.27972	0.559443	74921	342737	342738	2	2	0.001744	1	0.010684	1
chr18	77905408	77905751	-0.12103	0.605163	86839	372047	372051	5	10	0.001745	1	0.008397	1
chr17	43065745	43065876	-0.21646	0.649369	79874	354934	354936	3	3	0.001745	1	0.006717	1
chr8	689908	690059	-0.21626	0.648791	171734	190868	190870	3	3	0.001752	1	0.006736	1
chr20	50418554	50419193	-0.11709	0.585441	113104	400870	400874	5	9	0.001765	1	0.009308	1
chr7	32109831	32110145	-0.21475	0.644239	163002	172234	172236	3	14	0.001798	1	0.006892	1
chr11	27224020	27224020	-0.68975	0.689754	31127	245688	245688	1	1	0.001815	1	0.005511	1
chr2	3675155	3675155	-0.68944	0.689435	96183	42571	42571	1	2	0.001819	1	0.00552	1
chr11	1.02E+08	1.02E+08	-0.68801	0.688013	36710	258588	258588	1	1	0.001837	1	0.005557	1
chr11	1.34E+08	1.34E+08	0.275745	0.55149	39213	263814	263815	2	3	0.001841	1	0.011153	1
chr17	79108139	79108139	-0.68756	0.687557	83887	364607	364607	1	6	0.001844	1	0.005571	1
chr10	1.17E+08	1.17E+08	0.686245	0.686245	26334	233457	233457	1	2	0.001861	1	0.005605	1
chr5	308268	308268	-0.68537	0.685371	138723	112246	112246	1	11	0.001875	1	0.005629	1
chr20	46415320	46415320	0.684818	0.684818	112888	400355	400355	1	13	0.001883	1	0.005645	1
chr10	1E+08	1E+08	-0.68458	0.684583	25108	230514	230514	1	2	0.001886	1	0.005652	1
chr8	1905131	1905131	-0.68348	0.683479	172158	191896	191896	1	3	0.001901	1	0.00568	1
chr21	15645988	15646187	-0.27314	0.546287	114297	403674	403675	2	6	0.001912	1	0.01148	1
chr5	4230769	4230872	-0.2104	0.631187	139540	114479	114481	3	6	0.001941	1	0.007356	1
chr3	1.1E+08	1.1E+08	-0.68071	0.680713	124755	83361	83361	1	1	0.001941	1	0.005759	1
chr6	32525960	32525979	-0.27133	0.542655	152144	146409	146410	2	6	0.001961	1	0.011713	1
chr14	24475906	24476082	0.271155	0.542309	54389	297702	297703	2	4	0.001966	1	0.011736	1
chr16	14380714	14380714	-0.67875	0.67875	68657	328814	328814	1	1	0.00197	1	0.005812	1
chr2	1.32E+08	1.32E+08	-0.27039	0.54079	104008	58375	58376	2	4	0.001989	1	0.011835	1
chr4	1.2E+08	1.2E+08	-0.67692	0.676917	135776	106413	106413	1	1	0.001996	1	0.005861	1
chr17	171187	171434	-0.16636	0.66542	74802	342460	342463	4	5	0.002015	1	0.006196	1
chr11	1858572	1858572	0.675611	0.675611	29081	240781	240781	1	6	0.002016	1	0.005897	1
chr21	38362420	38362727	0.208264	0.624793	114825	404958	404960	3	6	0.002017	1	0.007597	1
chr11	65363274	65363274	-0.67548	0.675476	33904	252200	252200	1	7	0.002018	1	0.0059	1
chr7	32526065	32526065	0.674399	0.674399	163018	172275	172275	1	1	0.002033	1	0.00593	1
chr20	37064059	37064062	0.268659	0.537318	112367	398964	398965	2	11	0.002037	1	0.012058	1
chr6	1.28E+08	1.28E+08	-0.16571	0.662826	156763	157897	157900	4	7	0.002038	1	0.006275	1
chr13	1.13E+08	1.13E+08	0.207206	0.621618	53281	294869	294871	3	3	0.002053	1	0.007718	1
chr10	1.01E+08	1.01E+08	0.165129	0.660517	25158	230642	230645	4	9	0.002058	1	0.006347	1
chr19	39056084	39056216	-0.20705	0.621141	92070	385399	385401	3	6	0.002058	1	0.007735	1
chr7	94953653	94953810	-0.16511	0.66043	166454	179127	179130	4	11	0.002059	1	0.006349	1
chr14	1.07E+08	1.07E+08	-0.26788	0.53575	59930	309685	309686	2	2	0.00206	1	0.012162	1
chr2	2.37E+08	2.37E+08	-0.67149	0.671488	109504	69312	69312	1	21	0.002077	1	0.006015	1
chr2	71787431	71787615	0.26728	0.53456	100523	51202	51203	2	6	0.002079	1	0.012244	1
chr21	43528734	43528868	0.266715	0.533431	115112	405566	405567	2	2	0.002097	1	0.012321	1
chr17	21281318	21281507	-0.26626	0.532522	77526	349090	349091	2	16	0.002111	1	0.012382	1
chr11	60049097	60049097	0.668999	0.668999	32930	249495	249495	1	3	0.002115	1	0.006091	1
chr1	1.81E+08	1.81E+08	0.163579	0.654317	14169	32016	32019	4	6	0.002117	1	0.00655	1
chr22	24388327	24388327	-0.66795	0.667955	116689	408920	408920	1	1	0.00213	1	0.00612	1
chr17	40169518	40169520	0.265664	0.531328	79346	353405	353406	2	15	0.00213	1	0.012465	1
chr20	25281332	25281332	0.667465	0.667465	111688	397188	397188	1	1	0.002136	1	0.006134	1
chr1	2.35E+08	2.35E+08	-0.66703	0.667035	17885	39423	39423	1	9	0.002144	1	0.006148	1
chr4	1.2E+08	1.2E+08	-0.66625	0.666252	135750	106378	106378	1	2	0.002156	1	0.006171	1

chr1	92946700	92947588	-0.16248	0.649925	9535	21480	21483	4	5	0.00216	1	0.006699	1
chr19	2583601	2583601	0.665725	0.665725	87777	374321	374321	1	1	0.002165	1	0.006187	1
chr1	61479999	61479999	0.665223	0.665223	8182	18656	18656	1	1	0.002173	1	0.006202	1
chr1	12606996	12606996	0.664051	0.664051	2859	6904	6904	1	1	0.002193	1	0.006238	1
chr11	4565489	4565489	-0.66293	0.662926	29577	242511	242511	1	3	0.002209	1	0.006271	1
chr19	18234711	18234911	0.202966	0.608897	90656	381692	381694	3	3	0.002218	1	0.00824	1
chr10	1.35E+08	1.35E+08	0.661782	0.661782	28177	238050	238050	1	2	0.002227	1	0.006307	1
chr21	45161359	45161415	0.262012	0.524023	115327	406018	406019	2	2	0.002244	1	0.012986	1
chr16	58704495	58704495	-0.65956	0.659559	71358	334715	334715	1	1	0.002265	1	0.006378	1
chr10	47083342	47083398	0.159539	0.638158	21857	224059	224062	4	9	0.002272	1	0.007104	1
chr1	1.08E+08	1.08E+08	-0.65898	0.658976	10010	22509	22509	1	1	0.002275	1	0.006397	1
chr10	1.35E+08	1.35E+08	-0.20146	0.604389	28425	238873	238875	3	8	0.002276	1	0.008432	1
chr8	1897969	1898438	-0.20139	0.604165	172156	191883	191885	3	3	0.002279	1	0.008442	1
chr1	31732891	31732891	0.657889	0.657889	5335	12233	12233	1	1	0.002294	1	0.006431	1
chr1	85686316	85686316	-0.6576	0.657601	9141	20615	20615	1	1	0.002298	1	0.00644	1
chr12	1.31E+08	1.31E+08	0.260369	0.520737	48199	283880	283881	2	2	0.002299	1	0.01323	1
chr22	24376252	24376252	-0.65738	0.657383	116685	408909	408909	1	1	0.002302	1	0.006446	1
chr10	2147217	2147217	-0.65703	0.657035	19215	218716	218716	1	5	0.002308	1	0.006458	1
chr8	703701	703870	-0.26002	0.520048	171741	190887	190888	2	6	0.002312	1	0.013283	1
chr2	2.02E+08	2.02E+08	0.656686	0.656686	107050	64352	64352	1	1	0.002315	1	0.00647	1
chr2	96874307	96874307	0.656651	0.656651	101664	53680	53680	1	13	0.002315	1	0.006471	1
chr19	29217858	29218774	-0.1583	0.633205	91204	383115	383118	4	4	0.002323	1	0.007282	1
chr17	40838983	40839022	-0.25936	0.518727	79479	353827	353828	2	8	0.002333	1	0.013378	1
chr13	1.14E+08	1.14E+08	0.655399	0.655399	53458	295385	295385	1	3	0.002336	1	0.006514	1
chr10	94136	94136	0.654092	0.654092	18783	217614	217614	1	9	0.00236	1	0.006558	1
chr17	1645410	1645410	-0.65397	0.653973	75173	343371	343371	1	4	0.002362	1	0.006562	1
chr6	40145993	40145993	-0.65148	0.651479	153150	150386	150386	1	1	0.002408	1	0.006645	1
chr15	30861172	30861172	0.651342	0.651342	60674	311267	311267	1	1	0.002411	1	0.006649	1
chr19	49340574	49340765	0.198175	0.594524	93822	389735	389737	3	4	0.002421	1	0.008877	1
chr3	52569097	52569097	0.650164	0.650164	122626	79552	79552	1	5	0.002431	1	0.00669	1
chr11	1.02E+08	1.02E+08	-0.25646	0.512928	36701	258577	258578	2	13	0.00244	1	0.013823	1
chr8	11303828	11303828	0.64857	0.64857	172848	193176	193176	1	1	0.002459	1	0.006744	1
chr13	1.15E+08	1.15E+08	0.255841	0.511682	53795	296435	296436	2	2	0.002464	1	0.013921	1
chr2	3680222	3680295	0.255836	0.511672	96184	42573	42574	2	3	0.002464	1	0.013922	1
chr15	30336915	30337018	0.197134	0.591401	60647	311231	311233	3	3	0.002467	1	0.009022	1
chr17	31867	32503	0.154639	0.618558	74753	342348	342351	4	9	0.00247	1	0.00784	1
chr19	18549689	18549723	-0.25529	0.51057	90750	381940	381941	2	12	0.002485	1	0.014007	1
chr1	2.48E+08	2.48E+08	0.196651	0.589953	18634	40930	40932	3	3	0.00249	1	0.00909	1
chr19	310296	310512	-0.19659	0.58976	86865	372125	372127	3	3	0.002492	1	0.009099	1
chr1	38156462	38156652	0.153826	0.615304	6077	13983	13986	4	22	0.002505	1	0.007968	1
chr4	89299173	89299173	0.645348	0.645348	134783	104389	104389	1	10	0.002519	1	0.006855	1
chr17	78725375	78725470	-0.25421	0.508415	83722	364087	364088	2	5	0.002528	1	0.014181	1
chr9	1.4E+08	1.4E+08	-0.64403	0.644032	185829	217193	217193	1	1	0.002543	1	0.006899	1
chr14	1.02E+08	1.02E+08	-0.25374	0.507472	58888	307244	307245	2	2	0.002544	1	0.014256	1
chr19	38876666	38877134	-0.19525	0.585748	92024	385297	385299	3	4	0.002556	1	0.009294	1
chr13	1.13E+08	1.13E+08	-0.25329	0.506589	53205	294650	294651	2	3	0.002561	1	0.014327	1
chr11	33061845	33061907	0.253129	0.506259	31389	246363	246364	2	13	0.002567	1	0.014354	1
chr7	1E+08	1E+08	0.252643	0.505287	167069	180836	180837	2	8	0.002588	1	0.014434	1
chr10	49659559	49659559	-0.63944	0.639443	21985	224267	224267	1	1	0.002629	1	0.007058	1
chr22	23744717	23744725	0.251275	0.50255	116610	408723	408724	2	6	0.002644	1	0.014665	1
chr11	17032483	17032483	0.638606	0.638606	30658	244638	244638	1	1	0.002645	1	0.007088	1
chr4	69435250	69435601	-0.19316	0.579488	133809	102453	102455	3	3	0.002659	1	0.009601	1
chr21	36262896	36263808	0.149944	0.599777	114724	404701	404704	4	4	0.002672	1	0.008636	1
chr18	72167187	72167213	0.250606	0.501211	86269	370960	370961	2	2	0.002674	1	0.014779	1
chr6	31275881	31276418	0.149858	0.599431	151779	142883	142886	4	17	0.002676	1	0.008651	1
chr6	32795582	32795627	0.250376	0.500753	152190	146558	146559	2	9	0.002683	1	0.014818	1
chr7	1596103	1596217	-0.14963	0.59852	160553	166246	166249	4	7	0.002686	1	0.008694	1
chr6	32634316	32634344	-0.24925	0.498491	152162	146450	146451	2	3	0.002729	1	0.01501	1
chr5	1.16E+08	1.16E+08	-0.63422	0.634217	143995	123063	123063	1	1	0.002729	1	0.007245	1
chr6	33280400	33280436	0.148614	0.594456	152322	148197	148200	4	168	0.00273	1	0.00888	1
chr11	1.02E+08	1.02E+08	-0.63353	0.633526	36722	258613	258613	1	1	0.002743	1	0.007271	1
chr6	1.35E+08	1.35E+08	0.633066	0.633066	157160	158639	158639	1	1	0.00275	1	0.007286	1
chr5	1.81E+08	1.81E+08	-0.63291	0.632912	148725	133317	133317	1	1	0.002754	1	0.007292	1
chr3	1.95E+08	1.95E+08	-0.19134	0.57401	129778	93432	93434	3	7	0.002756	1	0.009882	1
chr9	1.39E+08	1.39E+08	0.632166	0.632166	185515	216632	216632	1	4	0.002768	1	0.00732	1
chr7	1096374	1096387	0.247326	0.494651	160334	165552	165553	2	2	0.002808	1	0.015344	1
chr17	71739339	71739385	-0.24709	0.494176	82341	360509	360510	2	2	0.002818	1	0.015386	1
chr4	46126261	46126448	0.146636	0.586544	133133	101107	101110	4	6	0.002819	1	0.009255	1
chr1	1687481	1687481	-0.62959	0.629594	482	1386	1386	1	12	0.002821	1	0.007418	1
chr2	89065319	89065330	-0.24687	0.49373	101462	53297	53298	2	10	0.002827	1	0.015425	1
chr9	1.4E+08	1.4E+08	0.628631	0.628631	185638	216826	216826	1	2	0.002839	1	0.007453	1
chr2	11430093	11430093	0.628623	0.628623	96808	43770	43770	1	1	0.00284	1	0.007453	1
chr7	1980808	1981254	0.246299	0.492597	160694	166669	166670	2	7	0.002851	1	0.015521	1
chr6	30074080	30074162	-0.24612	0.492238	151513	140699	140700	2	11	0.002859	1	0.015554	1

chr12	1.33E+08	1.33E+08	-0.62759	0.627588	48771	285396	285396	1	4	0.002861	1	0.007492	1
chr1	1229351	1229589	0.189288	0.567865	270	800	802	3	9	0.002866	1	0.010207	1
chr6	99842241	99842353	-0.14551	0.582024	155541	155350	155353	4	8	0.002869	1	0.009471	1
chr16	32857551	32857872	-0.24575	0.491504	70282	332419	332420	2	6	0.002876	1	0.015624	1
chr2	3486749	3487412	-0.14533	0.581309	96146	42459	42462	4	7	0.002878	1	0.009508	1
chr11	1.11E+08	1.11E+08	0.626721	0.626721	37081	259381	259381	1	1	0.002878	1	0.007523	1
chr8	8679450	8679450	-0.62671	0.626707	172597	192645	192645	1	1	0.002878	1	0.007524	1
chr4	1513089	1513259	-0.18898	0.566925	130560	95538	95540	3	5	0.002883	1	0.010258	1
chr15	59950012	59950097	-0.18855	0.565663	62586	315364	315366	3	10	0.002905	1	0.010324	1
chr17	35161825	35161825	-0.62481	0.624812	78555	351477	351477	1	1	0.002916	1	0.007596	1
chr6	26242929	26242929	0.623376	0.623376	150837	137596	137596	1	2	0.002946	1	0.00765	1
chr2	99771531	99771627	0.187848	0.563543	101931	54373	54375	3	12	0.002946	1	0.010446	1
chr7	1.06E+08	1.06E+08	0.623128	0.623128	167631	182011	182011	1	1	0.00295	1	0.00766	1
chr8	1.27E+08	1.27E+08	0.623023	0.623023	178596	204582	204582	1	1	0.002952	1	0.007664	1
chr6	30899568	30900149	-0.18759	0.562764	151693	142482	142484	3	12	0.00296	1	0.01049	1
chr13	28554842	28555387	-0.18735	0.562048	49672	287517	287519	3	4	0.002974	1	0.010531	1
chr18	77836355	77836355	0.621686	0.621686	86833	372031	372031	1	3	0.002981	1	0.007715	1
chr22	45809646	45810043	0.142954	0.571816	118427	413154	413157	4	14	0.002981	1	0.009997	1
chr11	14495049	14495049	-0.6201	0.620104	30512	244309	244309	1	1	0.003016	1	0.007778	1
chr7	1080558	1081061	0.186547	0.559642	160328	165521	165523	3	8	0.00302	1	0.010673	1
chr5	49708521	49708521	-0.61963	0.619628	141130	117494	117494	1	2	0.003026	1	0.007796	1
chr7	44079020	44079036	-0.24245	0.484891	163782	173632	173633	2	3	0.003031	1	0.016249	1
chr12	49319263	49319428	0.141553	0.566212	42346	270451	270454	4	16	0.003046	1	0.010296	1
chr17	41438351	41438402	-0.24147	0.482937	79580	354134	354135	2	4	0.003079	1	0.016438	1
chr10	1.32E+08	1.32E+08	0.140616	0.562464	27606	236281	236284	4	11	0.003088	1	0.010507	1
chr19	58570419	58570466	0.140343	0.561371	95498	394148	394151	4	9	0.0031	1	0.010571	1
chr11	2365581	2365581	0.615718	0.615718	29245	241422	241422	1	3	0.003111	1	0.007952	1
chr6	29856426	29856477	-0.1849	0.554705	151437	140223	140225	3	35	0.003119	1	0.010963	1
chr22	49579968	49579968	-0.6152	0.615197	118749	413814	413814	1	4	0.003121	1	0.007973	1
chr1	2.22E+08	2.22E+08	0.613447	0.613447	16687	36958	36958	1	1	0.00316	1	0.008047	1
chr8	1.45E+08	1.45E+08	-0.2398	0.479597	180234	207961	207962	2	3	0.003163	1	0.016759	1
chr17	213675	213743	-0.23968	0.47937	74824	342504	342505	2	2	0.003168	1	0.016781	1
chr6	30421275	30421376	-0.18409	0.552257	151580	141370	141372	3	11	0.003169	1	0.011108	1
chr5	1.26E+08	1.26E+08	0.138666	0.554663	144385	123765	123768	4	12	0.003171	1	0.010966	1
chr6	32145342	32145470	-0.13857	0.554285	152033	145876	145879	4	38	0.003175	1	0.010989	1
chr6	29690766	29690893	0.183694	0.551081	151401	139959	139961	3	39	0.003192	1	0.011178	1
chr16	7703812	7703947	0.183641	0.550922	68216	327960	327962	3	3	0.003194	1	0.011187	1
chr20	43935222	43935291	-0.18344	0.550334	112643	399724	399726	3	10	0.003208	1	0.011225	1
chr1	1.54E+08	1.54E+08	0.137663	0.55065	12009	27185	27188	4	8	0.003213	1	0.011204	1
chr11	68658793	68658836	-0.23845	0.476906	34621	254244	254245	2	2	0.003229	1	0.01703	1
chr14	1.06E+08	1.06E+08	0.609905	0.609905	59864	309560	309560	1	1	0.003241	1	0.008197	1
chr19	33623072	33623091	-0.23807	0.476144	91381	383535	383536	2	12	0.00325	1	0.017108	1
chr11	636460	636659	0.237831	0.475662	28652	239522	239523	2	10	0.003264	1	0.017157	1
chr18	77376639	77376689	0.237413	0.474826	86691	371767	371768	2	5	0.003286	1	0.017244	1
chr19	11199851	11199944	0.135571	0.542285	89459	378453	378456	4	5	0.003304	1	0.011737	1
chr12	1.33E+08	1.33E+08	0.606003	0.606003	48694	285193	285193	1	1	0.003332	1	0.00836	1
chr8	65640259	65640259	-0.60579	0.605787	175928	199418	199418	1	1	0.003337	1	0.008369	1
chr7	16457583	16457583	0.60558	0.60558	161924	169725	169725	1	1	0.003343	1	0.008378	1
chr11	5959923	5960081	0.133848	0.535393	29722	242745	242748	4	7	0.003376	1	0.012186	1
chr2	1.8E+08	1.8E+08	0.235689	0.471378	106202	62675	62676	2	2	0.003383	1	0.017602	1
chr15	46521595	46521595	-0.60341	0.603412	61882	313967	313967	1	1	0.003395	1	0.008473	1
chr4	74847646	74847761	0.133329	0.533315	134017	102808	102811	4	4	0.003396	1	0.01233	1
chr22	42524984	42525206	-0.23541	0.470828	118134	412424	412425	2	5	0.003397	1	0.017659	1
chr7	1.05E+08	1.05E+08	-0.60309	0.603095	167566	181914	181914	1	1	0.003402	1	0.008486	1
chr2	91847976	91847996	-0.18037	0.541105	101491	53342	53344	3	5	0.003404	1	0.011815	1
chr14	56979854	56979854	-0.60287	0.602872	55679	300574	300574	1	2	0.003408	1	0.008497	1
chr18	29205358	29205358	-0.60279	0.602791	85393	368770	368770	1	1	0.00341	1	0.0085	1
chr3	1.84E+08	1.84E+08	0.180089	0.540266	128929	91820	91822	3	4	0.003422	1	0.01187	1
chr19	57741988	57742260	0.132434	0.529738	95343	393678	393681	4	8	0.00343	1	0.012574	1
chr19	52074470	52074470	0.60181	0.60181	94424	391301	391301	1	5	0.003435	1	0.008543	1
chr1	1.62E+08	1.62E+08	-0.23467	0.469341	13052	29766	29767	2	3	0.003437	1	0.017813	1
chr19	36801408	36801408	-0.60136	0.601363	91809	384705	384705	1	3	0.003445	1	0.008563	1
chr1	2529359	2529359	-0.60085	0.600851	887	2530	2530	1	1	0.003458	1	0.008587	1
chr12	52798363	52798363	0.600849	0.600849	42852	271747	271747	1	1	0.003458	1	0.008587	1
chr15	91500009	91500193	0.234234	0.468468	65606	321549	321550	2	4	0.003462	1	0.017903	1
chr9	1.29E+08	1.29E+08	-0.59814	0.598135	183953	214225	214225	1	2	0.003525	1	0.008713	1
chr1	1.61E+08	1.61E+08	0.178505	0.535515	12937	29455	29457	3	10	0.003528	1	0.012178	1
chr19	34621907	34621907	0.597652	0.597652	91470	383745	383745	1	1	0.003537	1	0.008734	1
chr8	52321814	52322134	-0.1783	0.534911	175346	198177	198179	3	7	0.003542	1	0.01222	1
chr4	1.89E+08	1.89E+08	-0.23281	0.465622	138545	111894	111895	2	2	0.003547	1	0.018209	1
chr19	23945743	23946205	0.129177	0.516709	91176	383053	383056	4	8	0.003552	1	0.013529	1
chr10	81320623	81320623	-0.59685	0.596847	23902	227966	227966	1	1	0.003558	1	0.00877	1
chr10	50649666	50650021	0.128943	0.515771	22077	224463	224466	4	5	0.00356	1	0.013603	1
chr3	52828628	52828628	0.596467	0.596467	122659	79642	79642	1	6	0.003567	1	0.008788	1

chr19	56002240	56002446	-0.17784	0.533511	95111	393061	393063	3	3	0.003575	1	0.012316	1
chr6	31583915	31584223	-0.12793	0.511718	151840	143342	143345	4	6	0.003597	1	0.013918	1
chr7	12443880	12443886	-0.23133	0.462654	161845	169560	169561	2	11	0.003635	1	0.01853	1
chr15	57668539	57668812	0.126792	0.507166	62441	315073	315076	4	9	0.003635	1	0.014282	1
chr7	57071676	57071676	0.59365	0.59365	164654	175403	175403	1	1	0.003638	1	0.008917	1
chr6	32632000	32632036	0.231214	0.462429	152161	146439	146440	2	10	0.003642	1	0.018555	1
chr3	1.28E+08	1.28E+08	-0.59307	0.593068	126096	85990	85990	1	1	0.003654	1	0.008944	1
chr16	1022650	1022650	0.59306	0.59306	66822	324393	324393	1	2	0.003654	1	0.008945	1
chr6	8435883	8435919	0.176625	0.529875	149630	135221	135223	3	14	0.003662	1	0.012564	1
chr12	34371784	34372490	-0.12595	0.503817	41737	269164	269167	4	4	0.003663	1	0.014554	1
chr17	48858534	48858873	0.1252	0.500799	80798	357260	357263	4	4	0.003687	1	0.014815	1
chr4	1.86E+08	1.86E+08	0.590999	0.590999	138232	111232	111232	1	1	0.003707	1	0.00904	1
chr13	46291973	46291973	0.590988	0.590988	50719	289588	289588	1	1	0.003707	1	0.009041	1
chr15	26874098	26874788	0.124555	0.498219	60382	310686	310689	4	6	0.003708	1	0.015032	1
chr5	1.41E+08	1.41E+08	-0.12444	0.497774	145540	126547	126550	4	12	0.003711	1	0.015072	1
chr16	49518414	49518536	0.230018	0.460037	70591	333011	333012	2	3	0.003712	1	0.018817	1
chr3	1.39E+08	1.39E+08	-0.12425	0.497016	126828	87568	87571	4	8	0.003717	1	0.015141	1
chr6	34499314	34499504	-0.12425	0.497011	152546	149222	149225	4	6	0.003717	1	0.015141	1
chr19	46807119	46807466	-0.17587	0.527618	93343	388599	388601	3	6	0.003718	1	0.012722	1
chr6	1.1E+08	1.1E+08	-0.17586	0.527575	156001	156387	156389	3	3	0.003719	1	0.012725	1
chr12	1.33E+08	1.33E+08	0.229538	0.459075	48659	285072	285073	2	2	0.003744	1	0.018928	1
chr16	423420	423420	0.589173	0.589173	66523	323487	323487	1	3	0.003755	1	0.009127	1
chr13	1.14E+08	1.14E+08	-0.12259	0.490351	53389	295190	295193	4	6	0.003765	1	0.015733	1
chr10	45406644	45406847	-0.12248	0.489933	21728	223799	223802	4	6	0.003767	1	0.015769	1
chr1	2.47E+08	2.47E+08	0.588773	0.588773	18554	40736	40736	1	1	0.003767	1	0.009147	1
chr6	1.69E+08	1.69E+08	0.229155	0.458309	159454	163263	163264	2	4	0.003769	1	0.019015	1
chr10	37106999	37106999	0.588517	0.588517	21396	223067	223067	1	3	0.003774	1	0.009159	1
chr22	50753068	50753068	-0.58751	0.587506	118997	414343	414343	1	1	0.0038	1	0.009206	1
chr8	96085385	96085689	0.228386	0.456772	177141	201823	201824	2	5	0.003815	1	0.01919	1
chr7	1.58E+08	1.58E+08	-0.58662	0.586618	171469	190265	190265	1	3	0.003825	1	0.009251	1
chr19	3480508	3480672	0.119784	0.479135	88021	374846	374849	4	5	0.003833	1	0.016805	1
chr16	32290281	32290371	-0.22795	0.455897	70261	332384	332385	2	2	0.003844	1	0.019295	1
chr17	39597601	39597601	-0.58585	0.585853	79222	353092	353092	1	5	0.003845	1	0.009288	1
chr6	1.61E+08	1.61E+08	-0.11922	0.476868	158792	161801	161804	4	5	0.003846	1	0.017035	1
chr4	1.85E+08	1.85E+08	0.22779	0.455579	138109	110980	110981	2	2	0.003854	1	0.019333	1
chr21	27372387	27372461	0.118782	0.475127	114382	403877	403880	4	4	0.003854	1	0.01721	1
chr3	1.28E+08	1.28E+08	0.227752	0.455505	126066	85916	85917	2	2	0.003856	1	0.01934	1
chr4	2401542	2401542	-0.58443	0.584427	130804	96164	96164	1	5	0.003885	1	0.009355	1
chr20	10412596	10412596	0.584134	0.584134	111270	396029	396029	1	3	0.003892	1	0.009369	1
chr21	39644332	39644354	0.227168	0.454335	114886	405108	405109	2	6	0.003894	1	0.019474	1
chr19	52134289	52134296	0.226964	0.453929	94436	391319	391320	2	2	0.003908	1	0.019522	1
chr7	1.41E+08	1.41E+08	0.583417	0.583417	169623	185839	185839	1	1	0.003912	1	0.009403	1
chr7	6653510	6653510	0.583092	0.583092	161622	169123	169123	1	1	0.003921	1	0.009419	1
chr18	72837531	72837700	0.11395	0.4558	86290	371013	371016	4	6	0.003929	1	0.019307	1
chr8	37699239	37699923	0.112721	0.450883	174585	196596	196599	4	7	0.003942	1	0.01988	1
chr6	1.58E+08	1.58E+08	-0.58194	0.581945	158468	161123	161123	1	1	0.003951	1	0.009476	1
chr2	1.36E+08	1.36E+08	-0.58172	0.581718	104251	58781	58781	1	1	0.003957	1	0.009487	1
chr9	1.37E+08	1.37E+08	-0.58096	0.580961	185014	215876	215876	1	1	0.003977	1	0.009525	1
chr10	4868690	4868892	-0.22571	0.451421	19470	219237	219238	2	13	0.003991	1	0.019816	1
chr5	1.12E+08	1.12E+08	-0.58034	0.580344	143775	122642	122642	1	1	0.003994	1	0.009556	1
chr7	1.08E+08	1.08E+08	-0.58029	0.580288	167728	182204	182204	1	1	0.003996	1	0.009559	1
chr17	76141068	76141068	0.579588	0.579588	83128	362559	362559	1	3	0.004016	1	0.009596	1
chr8	47529015	47529280	-0.22506	0.450125	175096	197729	197730	2	3	0.004032	1	0.019973	1
chr9	1.31E+08	1.31E+08	-0.17181	0.51544	184188	214586	214588	3	3	0.004035	1	0.013629	1
chr17	406249	406501	0.171766	0.515299	74851	342565	342567	3	3	0.004039	1	0.01364	1
chr8	599963	600233	-0.17168	0.515043	171687	190749	190751	3	8	0.004045	1	0.013659	1
chr1	43425156	43425412	0.171678	0.515034	6693	15414	15416	3	15	0.004045	1	0.013659	1
chr15	25123287	25123731	-0.17113	0.513404	60165	310147	310149	3	4	0.004087	1	0.013787	1
chr10	855060	855060	-0.57705	0.577051	18988	218170	218170	1	1	0.004089	1	0.009726	1
chr3	1.95E+08	1.95E+08	-0.57679	0.576786	129705	93303	93303	1	1	0.004097	1	0.009738	1
chr17	78851149	78851262	0.1709	0.5127	83769	364223	364225	3	4	0.004107	1	0.013841	1
chr8	1.46E+08	1.46E+08	0.170675	0.512025	180379	208422	208424	3	8	0.004125	1	0.013893	1
chr17	8792092	8792092	-0.57557	0.575571	76352	346642	346642	1	4	0.004132	1	0.0098	1
chr1	18902165	18902165	-0.57549	0.575491	3655	8433	8433	1	1	0.004134	1	0.009804	1
chr1	18767776	18767776	-0.5749	0.574898	3646	8412	8412	1	1	0.004152	1	0.009837	1
chr11	4389638	4389638	0.57413	0.57413	29567	242492	242492	1	7	0.004174	1	0.009876	1
chr1	29102029	29102153	-0.16998	0.509948	5128	11860	11862	3	6	0.004181	1	0.014058	1
chr12	1.33E+08	1.33E+08	0.169457	0.508372	48680	285134	285136	3	5	0.004224	1	0.014183	1
chr5	1.35E+08	1.35E+08	0.571737	0.571737	144982	125129	125129	1	1	0.004244	1	0.01	1
chr9	1.25E+08	1.25E+08	-0.16906	0.507193	183645	213743	213745	3	3	0.004257	1	0.01428	1
chr14	1.02E+08	1.02E+08	0.169057	0.50717	58885	307237	307239	3	3	0.004257	1	0.014281	1
chr10	1.35E+08	1.35E+08	0.571193	0.571193	28113	237856	237856	1	7	0.004258	1	0.010028	1
chr11	65631880	65631880	0.570915	0.570915	33978	252425	252425	1	1	0.004267	1	0.010043	1
chr13	26590097	26590097	0.570083	0.570083	49502	287076	287076	1	1	0.004292	1	0.01009	1

chr13	24788196	24788196	0.569342	0.569342	49369	286784	286784	1	1	0.004315	1	0.010131	1
chr2	1.1E+08	1.1E+08	-0.16823	0.504688	102580	55584	55586	3	17	0.004327	1	0.014482	1
chr1	1562535	1562535	0.567976	0.567976	432	1258	1258	1	6	0.004355	1	0.010201	1
chr13	1.11E+08	1.11E+08	0.567342	0.567342	52843	293755	293755	1	1	0.004375	1	0.010237	1
chr16	594329	594329	-0.56726	0.567255	66584	323646	323646	1	1	0.004378	1	0.010241	1
chr11	93063208	93063302	0.219984	0.439967	36339	257892	257893	2	13	0.004401	1	0.021254	1
chr2	1.37E+08	1.37E+08	-0.56582	0.565825	104280	58855	58855	1	1	0.00442	1	0.010315	1
chr8	1365049	1365175	0.219638	0.439277	171977	191419	191420	2	6	0.004428	1	0.021343	1
chr8	685830	686594	-0.16694	0.500833	171731	190856	190858	3	6	0.004443	1	0.014812	1
chr22	46762943	46763229	-0.21928	0.438557	118548	413458	413459	2	2	0.004457	1	0.021439	1
chr11	1457153	1457346	-0.21883	0.437658	28952	240444	240445	2	4	0.004494	1	0.021559	1
chr13	1.01E+08	1.01E+08	0.562986	0.562986	52382	292834	292834	1	3	0.004512	1	0.010478	1
chr2	64839958	64839958	-0.56201	0.562007	99936	49981	49981	1	1	0.004544	1	0.010533	1
chr2	2.25E+08	2.25E+08	-0.56158	0.561578	108589	67437	67437	1	1	0.004558	1	0.010559	1
chr7	1.38E+08	1.38E+08	-0.16563	0.496899	169357	185306	185308	3	7	0.004561	1	0.01515	1
chr13	27295928	27296010	-0.1651	0.495313	49546	287172	287174	3	3	0.004611	1	0.015286	1
chr17	1133546	1133706	-0.16491	0.494723	75049	343042	343044	3	3	0.004629	1	0.015337	1
chr22	50483499	50483545	-0.21692	0.433844	118903	414100	414101	2	5	0.004655	1	0.022076	1
chr6	24910720	24911001	-0.21676	0.433511	150676	137123	137124	2	13	0.004669	1	0.022119	1
chr6	35765093	35765176	0.216726	0.433453	152719	149577	149578	2	4	0.004671	1	0.022127	1
chr6	46871130	46871130	0.558216	0.558216	153916	152085	152085	1	2	0.004672	1	0.010757	1
chr19	21265264	21265421	-0.16433	0.492997	91053	382751	382753	3	5	0.004684	1	0.015489	1
chr7	1.48E+08	1.48E+08	-0.55768	0.557683	169935	186435	186435	1	1	0.00469	1	0.010788	1
chr10	81680307	81680307	0.557667	0.557667	23926	228006	228006	1	4	0.00469	1	0.010789	1
chr21	31709690	31709690	-0.55712	0.557121	114465	404063	404063	1	3	0.00471	1	0.010823	1
chr21	44105265	44105474	0.216255	0.432511	115193	405723	405724	2	3	0.00471	1	0.022253	1
chr15	23378564	23378571	-0.21624	0.43248	60105	310011	310012	2	4	0.004712	1	0.022258	1
chr11	44881786	44881786	0.557002	0.557002	31926	247431	247431	1	5	0.004714	1	0.010829	1
chr1	9341141	9341141	0.55659	0.55659	2261	5673	5673	1	1	0.004727	1	0.010853	1
chr7	73442480	73442531	-0.16374	0.49123	165417	176919	176921	3	5	0.00474	1	0.01565	1
chr19	8464538	8464538	-0.55605	0.556049	89052	377354	377354	1	3	0.004746	1	0.010884	1
chr1	39281613	39282003	0.163659	0.490978	6207	14285	14287	3	4	0.004748	1	0.015672	1
chr17	44847220	44847427	0.163535	0.490604	80093	355417	355419	3	3	0.004761	1	0.015709	1
chr5	1.4E+08	1.4E+08	-0.55512	0.555119	145437	126185	126185	1	10	0.004778	1	0.01094	1
chr1	28826586	28826586	0.553644	0.553644	5083	11723	11723	1	1	0.004828	1	0.011026	1
chr10	1.32E+08	1.32E+08	0.214776	0.429552	27587	236224	236225	2	5	0.004835	1	0.022669	1
chr8	641466	641497	0.162643	0.487928	171709	190791	190793	3	3	0.004849	1	0.015956	1
chr7	1.49E+08	1.49E+08	-0.55303	0.553035	170039	186659	186659	1	1	0.004849	1	0.011062	1
chr8	1327546	1327546	-0.55299	0.552988	171961	191383	191383	1	6	0.004851	1	0.011065	1
chr6	21916568	21916568	0.550835	0.550835	150533	136872	136872	1	1	0.004923	1	0.011191	1
chr16	77469710	77469710	-0.54997	0.549966	72624	337544	337544	1	12	0.004955	1	0.011249	1
chr19	725168	725168	-0.54961	0.549614	87043	372510	372510	1	1	0.004968	1	0.011271	1
chr6	37618009	37618123	-0.21324	0.426481	152959	150054	150055	2	3	0.004973	1	0.023113	1
chr2	2.32E+08	2.32E+08	-0.2132	0.426406	108949	68118	68119	2	10	0.004976	1	0.023123	1
chr8	1847999	1848143	-0.16131	0.483931	172135	191824	191826	3	4	0.004983	1	0.016342	1
chr14	37445440	37445440	0.548443	0.548443	54890	298978	298978	1	1	0.005011	1	0.011343	1
chr1	39249425	39249604	0.160794	0.482382	6203	14271	14273	3	5	0.005036	1	0.016491	1
chr19	10679729	10679729	0.547671	0.547671	89370	378214	378214	1	10	0.005038	1	0.01139	1
chr3	1.34E+08	1.34E+08	0.54718	0.54718	126583	87007	87007	1	15	0.005056	1	0.011421	1
chr3	1.84E+08	1.84E+08	-0.54669	0.546688	128943	91857	91857	1	2	0.005075	1	0.011454	1
chr6	30850581	30850581	-0.54647	0.546465	151675	142294	142294	1	49	0.005083	1	0.011469	1
chr7	1.03E+08	1.03E+08	0.546425	0.546425	167444	181680	181680	1	1	0.005085	1	0.011472	1
chr12	1.33E+08	1.33E+08	-0.21175	0.423496	48560	284746	284747	2	2	0.005112	1	0.023551	1
chr14	62035431	62035585	0.15994	0.47982	55996	301290	301292	3	3	0.005124	1	0.016739	1
chr3	1.19E+08	1.19E+08	-0.54492	0.544917	125260	84271	84271	1	1	0.005141	1	0.01157	1
chr2	1.05E+08	1.05E+08	0.159438	0.478314	102302	54995	54997	3	3	0.005176	1	0.016888	1
chr7	15651770	15651770	0.543822	0.543822	161911	169697	169697	1	1	0.005181	1	0.011636	1
chr8	674434	674560	-0.15842	0.475258	171726	190836	190838	3	6	0.005285	1	0.017198	1
chr17	154499	155045	-0.15809	0.474267	74797	342451	342453	3	5	0.00532	1	0.017301	1
chr16	80231787	80231787	0.540169	0.540169	72743	337731	337731	1	1	0.00532	1	0.011877	1
chr12	1.33E+08	1.33E+08	0.209588	0.419176	48706	285228	285229	2	5	0.005321	1	0.024212	1
chr5	1.41E+08	1.41E+08	-0.15796	0.473868	145656	127030	127032	3	9	0.005333	1	0.017343	1
chr16	12058616	12058616	-0.53972	0.53972	68542	328614	328614	1	1	0.005338	1	0.011906	1
chr11	1.23E+08	1.23E+08	-0.53941	0.53941	38294	262015	262015	1	1	0.00535	1	0.011926	1
chr17	63926873	63926873	0.539171	0.539171	81864	359612	359612	1	1	0.00536	1	0.011942	1
chr1	1.1E+08	1.1E+08	0.538608	0.538608	10102	22729	22729	1	1	0.005379	1	0.011975	1
chr10	54632322	54632322	0.538065	0.538065	22278	224870	224870	1	1	0.005398	1	0.012009	1
chr10	81904244	81904331	-0.20873	0.417455	23946	228055	228056	2	3	0.005405	1	0.024478	1
chr5	1.69E+08	1.69E+08	-0.53754	0.537538	147262	130104	130104	1	2	0.005419	1	0.012043	1
chr2	2.07E+08	2.07E+08	0.537073	0.537073	107352	64872	64872	1	1	0.005437	1	0.012075	1
chr11	1.03E+08	1.03E+08	-0.53675	0.536751	36751	258661	258661	1	3	0.00545	1	0.012096	1
chr12	1.31E+08	1.31E+08	0.208225	0.416451	48189	283849	283850	2	3	0.005457	1	0.024636	1
chr7	1.4E+08	1.4E+08	-0.53651	0.53651	169591	185754	185754	1	1	0.00546	1	0.012112	1
chr12	1.23E+08	1.23E+08	0.208191	0.416381	47349	281990	281991	2	2	0.00546	1	0.024646	1

chr6	32485396	32485396	0.536255	0.536255	152132	146380	146380	1	1	0.005469	1	0.012128	1
chr1	2E+08	2E+08	-0.20791	0.415818	14839	33216	33217	2	15	0.005485	1	0.024737	1
chr6	6606065	6606132	-0.2079	0.4158	149445	134870	134871	2	3	0.005486	1	0.024739	1
chr13	1.14E+08	1.14E+08	0.207678	0.415356	53408	295232	295233	2	2	0.00551	1	0.024808	1
chr12	66579675	66579675	0.535137	0.535137	44093	274957	274957	1	1	0.005512	1	0.012204	1
chr8	43147211	43147397	0.207632	0.415263	175052	197629	197630	2	9	0.005515	1	0.024822	1
chr9	35646325	35646566	0.20751	0.41502	181344	210196	210197	2	4	0.005526	1	0.024861	1
chr6	33422476	33422521	0.156163	0.46849	152355	148750	148752	3	54	0.005527	1	0.017901	1
chr6	32498493	32498493	0.534147	0.534147	152139	146400	146400	1	1	0.005553	1	0.012273	1
chr1	43770523	43770707	0.15573	0.46719	6737	15505	15507	3	5	0.005574	1	0.01804	1
chr16	412168	412393	0.206443	0.412885	66518	323470	323471	2	3	0.005636	1	0.025197	1
chr7	1.5E+08	1.5E+08	-0.53194	0.531941	170171	186931	186931	1	1	0.005639	1	0.012424	1
chr11	14431708	14431708	-0.53193	0.531929	30507	244304	244304	1	1	0.005639	1	0.012425	1
chr7	1.58E+08	1.58E+08	-0.15511	0.465322	171442	190195	190197	3	3	0.005642	1	0.018242	1
chr22	49812267	49812410	-0.20579	0.411573	118776	413859	413860	2	2	0.005702	1	0.025406	1
chr11	45868449	45868501	0.154516	0.463549	32059	247738	247740	3	15	0.005708	1	0.018432	1
chr6	33033176	33033312	0.205324	0.410648	152238	147158	147159	2	3	0.005751	1	0.025557	1
chr22	19162276	19162276	-0.52905	0.529051	116150	407682	407682	1	1	0.005756	1	0.012623	1
chr16	15858790	15858790	0.528405	0.528405	68781	329060	329060	1	1	0.005781	1	0.012667	1
chr6	16962712	16962712	-0.52824	0.528239	150279	136406	136406	1	1	0.005788	1	0.012678	1
chr2	2.1E+08	2.1E+08	0.204776	0.409552	107564	65318	65319	2	3	0.005812	1	0.025735	1
chr6	41376604	41376993	0.15347	0.46041	153237	150593	150595	3	6	0.005824	1	0.018776	1
chr4	6728936	6729110	-0.20458	0.409166	131462	97741	97742	2	3	0.005834	1	0.025799	1
chr11	85378422	85378422	-0.52714	0.527135	36038	257353	257353	1	1	0.005835	1	0.012757	1
chr14	80858152	80858152	0.526906	0.526906	57418	304166	304166	1	1	0.005845	1	0.012774	1
chr19	3910932	3910932	-0.52682	0.526816	88179	375212	375212	1	1	0.005848	1	0.012781	1
chr1	2.05E+08	2.05E+08	0.526728	0.526728	15463	34521	34521	1	1	0.005852	1	0.012788	1
chr10	1.35E+08	1.35E+08	0.204334	0.408668	28201	238151	238152	2	7	0.005863	1	0.02588	1
chr17	61602292	61602292	-0.52539	0.525386	81619	359038	359038	1	1	0.005912	1	0.012888	1
chr4	23729861	23729861	-0.52517	0.525166	132382	99605	99605	1	1	0.005922	1	0.012905	1
chr13	1.14E+08	1.14E+08	-0.52282	0.522818	53615	295847	295847	1	8	0.006019	1	0.013075	1
chr4	1366463	1366463	0.522778	0.522778	130526	95443	95443	1	6	0.006021	1	0.013078	1
chr5	1809712	1809712	-0.52262	0.522623	139261	113738	113738	1	5	0.006027	1	0.013088	1
chr6	1.39E+08	1.39E+08	0.151762	0.455286	157419	159129	159131	3	3	0.006027	1	0.019366	1
chr8	1.27E+08	1.27E+08	0.522617	0.522617	178607	204593	204593	1	1	0.006027	1	0.013089	1
chr19	35720295	35720295	-0.52253	0.522526	91593	384071	384071	1	5	0.006031	1	0.013096	1
chr7	1.3E+08	1.3E+08	0.521996	0.521996	168778	184242	184242	1	1	0.006054	1	0.013136	1
chr2	2.4E+08	2.4E+08	-0.20263	0.405268	109968	70276	70277	2	3	0.00606	1	0.026464	1
chr20	11869467	11869467	0.521676	0.521676	111290	396085	396085	1	1	0.006068	1	0.013161	1
chr4	1.3E+08	1.3E+08	-0.52153	0.52153	136143	107127	107127	1	1	0.006076	1	0.013173	1
chr1	1.08E+08	1.08E+08	0.151176	0.453528	10007	22504	22506	3	4	0.006094	1	0.019572	1
chr7	149976	150084	0.151175	0.453524	160007	164606	164608	3	4	0.006094	1	0.019572	1
chr19	38229377	38229377	-0.52101	0.521012	91950	385114	385114	1	1	0.006098	1	0.01321	1
chr7	190334	190423	-0.20229	0.40458	160025	164652	164653	2	2	0.0061	1	0.026579	1
chr12	1.33E+08	1.33E+08	-0.15088	0.452642	48674	285116	285118	3	3	0.006129	1	0.019674	1
chr6	29894322	29894341	-0.20199	0.403977	151448	140257	140258	2	29	0.006133	1	0.026681	1
chr12	53359155	53359506	0.150786	0.452359	42940	271952	271954	3	4	0.00614	1	0.019707	1
chr8	96619989	96619989	-0.51981	0.519813	177168	201866	201866	1	1	0.006152	1	0.013301	1
chr13	44453233	44453235	0.201613	0.403225	50560	289267	289268	2	17	0.006176	1	0.026814	1
chr7	1595067	1595067	-0.51883	0.518827	160552	166243	166243	1	2	0.006194	1	0.013371	1
chr3	1.91E+08	1.91E+08	-0.51871	0.518709	129449	92799	92799	1	4	0.006199	1	0.01338	1
chr11	74026371	74026371	-0.51848	0.51848	35372	256051	256051	1	1	0.006209	1	0.013398	1
chr17	81025461	81025461	-0.51842	0.518415	84607	366831	366831	1	2	0.006211	1	0.013403	1
chr22	50250283	50250283	-0.51804	0.518036	118855	413993	413993	1	1	0.006229	1	0.01343	1
chr11	71164098	71164112	-0.15005	0.450161	35008	255247	255249	3	13	0.006232	1	0.019968	1
chr22	49447845	49448123	-0.20109	0.402172	118735	413793	413794	2	4	0.006242	1	0.026999	1
chr11	1.23E+08	1.23E+08	-0.51757	0.517574	38242	261920	261920	1	1	0.006249	1	0.013464	1
chr2	2.42E+08	2.42E+08	0.5173	0.5173	110251	70930	70930	1	4	0.006261	1	0.013484	1
chr5	1.25E+08	1.25E+08	-0.51718	0.517179	144315	123634	123634	1	1	0.006266	1	0.013493	1
chr18	47016218	47016218	0.517171	0.517171	85806	369804	369804	1	3	0.006266	1	0.013494	1
chr12	1.23E+08	1.23E+08	0.517164	0.517164	47292	281849	281849	1	5	0.006266	1	0.013494	1
chr6	32063895	32063991	0.149591	0.448773	152005	145416	145418	3	54	0.006289	1	0.020136	1
chr5	71852464	71852862	0.149554	0.448661	142120	119374	119376	3	4	0.006293	1	0.020148	1
chr11	1.33E+08	1.33E+08	0.149437	0.44831	39146	263691	263693	3	3	0.006307	1	0.02019	1
chr2	2.33E+08	2.33E+08	0.516136	0.516136	109047	68333	68333	1	1	0.006313	1	0.013574	1
chr17	77924582	77924733	-0.14938	0.448134	83515	363564	363566	3	15	0.006314	1	0.020212	1
chr10	45720040	45720134	-0.20034	0.400679	21769	223895	223896	2	4	0.00633	1	0.027261	1
chr16	33948806	33948885	-0.20014	0.400274	70330	332494	332495	2	2	0.006355	1	0.027337	1
chr3	52931532	52931657	-0.14903	0.447089	122677	79675	79677	3	10	0.006358	1	0.020342	1
chr19	56915855	56915855	-0.5151	0.515096	95264	393442	393442	1	12	0.00636	1	0.013655	1
chr10	1.18E+08	1.18E+08	-0.51466	0.514662	26404	233614	233614	1	1	0.006378	1	0.013687	1
chr6	1.14E+08	1.14E+08	0.199886	0.399772	156277	156938	156939	2	3	0.006388	1	0.027427	1
chr11	1.01E+08	1.01E+08	-0.51445	0.514448	36658	258485	258485	1	1	0.006388	1	0.013704	1
chr5	1.71E+08	1.71E+08	0.513423	0.513423	147387	130383	130383	1	1	0.006435	1	0.013785	1

chr15	33023237	33023586	0.199459	0.398917	60835	311569	311570	2	2	0.00644	1	0.027578	1
chr7	1.34E+08	1.34E+08	0.199406	0.398811	169091	184823	184824	2	9	0.006447	1	0.027596	1
chr12	50523625	50523625	0.512043	0.512043	42558	271077	271077	1	1	0.006497	1	0.013891	1
chr1	2.47E+08	2.47E+08	-0.51201	0.512008	18543	40699	40699	1	1	0.006499	1	0.013894	1
chr10	1.35E+08	1.35E+08	0.147793	0.443379	28166	238017	238019	3	4	0.006516	1	0.020817	1
chr3	1.39E+08	1.39E+08	-0.51111	0.511108	126877	87687	87687	1	2	0.006539	1	0.013965	1
chr8	1.45E+08	1.45E+08	-0.51083	0.510833	180070	207476	207476	1	1	0.006552	1	0.013986	1
chr16	53543985	53544321	0.147194	0.441583	70861	333542	333544	3	3	0.006588	1	0.021047	1
chr17	79503859	79503877	-0.19817	0.396344	84050	365086	365087	2	7	0.006609	1	0.028049	1
chr17	34067305	34067305	0.509565	0.509565	78420	351188	351188	1	1	0.006611	1	0.014089	1
chr20	32255491	32256071	-0.14697	0.440909	111977	397902	397904	3	6	0.006616	1	0.021131	1
chr6	33092130	33092130	0.509367	0.509367	152262	147271	147271	1	13	0.00662	1	0.014103	1
chr7	1.05E+08	1.05E+08	0.509294	0.509294	167535	181849	181849	1	1	0.006623	1	0.01411	1
chr16	788035	788184	-0.14683	0.440504	66699	324035	324037	3	3	0.006634	1	0.021185	1
chr15	1.02E+08	1.02E+08	-0.50894	0.508942	66277	322944	322944	1	4	0.006639	1	0.014137	1
chr6	1.52E+08	1.52E+08	-0.14662	0.439873	158142	160590	160592	3	6	0.00666	1	0.021268	1
chr17	78233839	78233937	0.197698	0.395396	83612	363828	363829	2	5	0.006672	1	0.028226	1
chr14	1.07E+08	1.07E+08	-0.19758	0.395153	59933	309689	309690	2	3	0.006688	1	0.028271	1
chr12	1.33E+08	1.33E+08	-0.50759	0.507593	48828	285556	285556	1	7	0.006702	1	0.014245	1
chr19	54960355	54960853	-0.14622	0.438646	94885	392518	392520	3	7	0.006715	1	0.021427	1
chr5	1.27E+08	1.27E+08	-0.14614	0.43841	144405	123807	123809	3	16	0.006724	1	0.021458	1
chr7	44184403	44184403	0.50611	0.50611	163815	173723	173723	1	9	0.006774	1	0.014365	1
chr20	1757570	1757780	0.196851	0.393701	110838	394964	394965	2	3	0.006786	1	0.028545	1
chr12	9886905	9886905	0.505659	0.505659	40526	266805	266805	1	1	0.006797	1	0.014403	1
chr21	38349937	38350069	0.19672	0.393439	114820	404950	404951	2	2	0.006803	1	0.028593	1
chr15	97311928	97311928	0.505115	0.505115	65943	322258	322258	1	3	0.006822	1	0.014446	1
chr17	72462559	72463080	0.145359	0.436076	82425	360727	360729	3	5	0.006826	1	0.021769	1
chr12	1.32E+08	1.32E+08	-0.50498	0.504985	48429	284390	284390	1	3	0.006829	1	0.014458	1
chr22	39784769	39784982	0.145316	0.435949	117840	411680	411682	3	6	0.006831	1	0.021785	1
chr5	667968	668501	-0.14529	0.435863	138869	112639	112641	3	4	0.006835	1	0.021798	1
chr5	1.33E+08	1.33E+08	-0.5045	0.504498	144739	124526	124526	1	1	0.006851	1	0.014497	1
chr6	30431699	30431724	0.145061	0.435183	151583	141386	141388	3	34	0.006865	1	0.021892	1
chr8	1.44E+08	1.44E+08	-0.50421	0.504207	179987	207248	207248	1	1	0.006865	1	0.014522	1
chr9	90273181	90273181	-0.50404	0.504043	182166	211440	211440	1	1	0.006872	1	0.014535	1
chr11	69982916	69982941	-0.19612	0.392232	34833	254800	254801	2	3	0.006884	1	0.028823	1
chr6	33253607	33253607	-0.50356	0.503564	152312	147981	147981	1	2	0.006897	1	0.014576	1
chr3	1.15E+08	1.15E+08	-0.50322	0.50322	125080	83961	83961	1	1	0.006915	1	0.014606	1
chr10	1.35E+08	1.35E+08	0.195812	0.391624	28137	237923	237924	2	2	0.006929	1	0.028942	1
chr7	39875276	39875276	-0.50288	0.502882	163520	173157	173157	1	1	0.006933	1	0.014636	1
chr21	36258423	36258497	0.195751	0.391503	114720	404686	404687	2	2	0.006938	1	0.028966	1
chr3	36986992	36986992	0.502764	0.502764	120997	75573	75573	1	13	0.00694	1	0.014647	1
chr15	22833335	22833400	-0.19555	0.391105	60040	309847	309848	2	11	0.006964	1	0.029041	1
chr16	89315104	89315186	-0.1954	0.390794	74439	341572	341573	2	4	0.006988	1	0.029102	1
chr2	1.28E+08	1.28E+08	0.144042	0.432125	103484	57306	57308	3	8	0.006995	1	0.022309	1
chr6	1.69E+08	1.69E+08	-0.50157	0.501569	159567	163543	163543	1	4	0.007	1	0.014749	1
chr1	25655526	25655526	0.501568	0.501568	4564	10422	10422	1	1	0.007	1	0.014749	1
chr13	1.15E+08	1.15E+08	-0.50151	0.501509	53772	296360	296360	1	13	0.007002	1	0.014753	1
chr19	17905589	17905626	0.195205	0.39041	90581	381494	381495	2	6	0.007015	1	0.029175	1
chr1	2.1E+08	2.1E+08	0.143694	0.431083	15971	35538	35540	3	8	0.007041	1	0.022452	1
chr12	1.32E+08	1.32E+08	-0.195	0.390005	48256	284026	284027	2	2	0.007043	1	0.029254	1
chr3	9988144	9988590	0.194988	0.389975	119517	72701	72702	2	2	0.007045	1	0.02926	1
chr5	75955929	75955929	0.500054	0.500054	142384	119946	119946	1	1	0.007077	1	0.014877	1
chr16	11603344	11603344	-0.49979	0.499789	68492	328508	328508	1	2	0.007089	1	0.014899	1
chr17	948956	948956	-0.49953	0.499533	74995	342905	342905	1	1	0.007102	1	0.014921	1
chr13	95652307	95652307	0.499461	0.499461	52058	292194	292194	1	1	0.007105	1	0.014926	1
chr6	32312351	32312351	0.49934	0.49934	152092	146269	146269	1	2	0.007111	1	0.014936	1
chr1	2.1E+08	2.1E+08	-0.19454	0.389071	15991	35589	35590	2	9	0.007111	1	0.02944	1
chr8	57351067	57351185	0.194461	0.388922	175598	198727	198728	2	6	0.007121	1	0.02947	1
chr17	14201680	14201744	-0.19441	0.388829	76704	347325	347326	2	6	0.007127	1	0.029487	1
chr22	50482925	50483123	-0.19438	0.388751	118903	414097	414098	2	5	0.007134	1	0.029505	1
chr17	30822561	30822961	-0.19437	0.388738	78207	350707	350708	2	5	0.007135	1	0.029507	1
chr4	99851156	99851281	0.142902	0.428705	135035	104896	104898	3	18	0.007147	1	0.022787	1
chr17	79459563	79459563	-0.4983	0.498304	84037	365033	365033	1	2	0.007164	1	0.015026	1
chr20	62421310	62421310	-0.49777	0.497775	114077	403205	403205	1	1	0.00719	1	0.015072	1
chr5	10639679	10639679	0.497723	0.497723	139874	115132	115132	1	1	0.007193	1	0.015077	1
chr2	2.19E+08	2.19E+08	-0.4976	0.497604	107965	65993	65993	1	1	0.007199	1	0.015088	1
chr14	24422837	24422872	-0.19387	0.387736	54379	297674	297675	2	12	0.007209	1	0.029703	1
chr17	37123767	37123843	0.14239	0.427171	78791	352074	352076	3	9	0.007215	1	0.023008	1
chr8	1144375	1144375	0.496964	0.496964	171890	191228	191228	1	1	0.007234	1	0.015145	1
chr6	1.7E+08	1.7E+08	0.496739	0.496739	159865	164240	164240	1	2	0.007245	1	0.015164	1
chr16	818807	819064	-0.19354	0.387075	66711	324082	324083	2	4	0.007259	1	0.029834	1
chr3	48335857	48335857	0.496441	0.496441	121994	77766	77766	1	1	0.00726	1	0.015189	1
chr1	1.67E+08	1.67E+08	0.496344	0.496344	13404	30463	30463	1	1	0.007264	1	0.015198	1
chr12	1.25E+08	1.25E+08	0.496204	0.496204	47586	282557	282557	1	7	0.007271	1	0.015208	1

chr14	96180576	96180643	-0.14198	0.425954	58312	305895	305897	3	11	0.007271	1	0.023185	1
chr10	70319645	70319774	0.193308	0.386615	22777	225804	225805	2	11	0.007293	1	0.029922	1
chr17	80899280	80899280	-0.49533	0.495334	84550	366676	366676	1	8	0.007317	1	0.015284	1
chr7	1.01E+08	1.01E+08	-0.14153	0.424597	167198	181175	181177	3	3	0.007333	1	0.023386	1
chr4	2439700	2439700	-0.49489	0.494888	130817	96197	96197	1	4	0.00734	1	0.015323	1
chr16	66098650	66098650	0.494651	0.494651	71510	334923	334923	1	1	0.007354	1	0.015344	1
chr7	1.59E+08	1.59E+08	0.192827	0.385653	171539	190414	190415	2	6	0.007369	1	0.030121	1
chr1	1.58E+08	1.58E+08	0.141222	0.423666	12648	28849	28851	3	6	0.007375	1	0.023525	1
chr16	10855738	10855738	-0.49399	0.493987	68389	328310	328310	1	4	0.007387	1	0.015402	1
chr19	54515169	54515274	-0.14107	0.423223	94803	392296	392298	3	5	0.007394	1	0.023591	1
chr4	39448653	39449053	0.141059	0.423178	132878	100475	100477	3	9	0.007397	1	0.023597	1
chr16	86593603	86593603	0.493663	0.493663	73568	339482	339482	1	1	0.007404	1	0.015429	1
chr6	31846996	31847028	0.140772	0.422317	151938	144556	144558	3	10	0.007437	1	0.023728	1
chr19	12305854	12305869	0.19213	0.38426	89666	379052	379053	2	6	0.007476	1	0.0304	1
chr6	1619094	1619162	-0.14044	0.421332	148955	133888	133890	3	6	0.007481	1	0.023876	1
chr1	26670609	26670609	-0.49147	0.491471	4740	10850	10850	1	2	0.007521	1	0.015627	1
chr14	74001118	74001118	-0.49128	0.491276	56829	302908	302908	1	1	0.007533	1	0.015646	1
chr13	98002933	98002933	-0.49065	0.490655	52154	292403	292403	1	1	0.007566	1	0.015704	1
chr17	40706682	40706718	0.191502	0.383004	79445	353690	353691	2	8	0.007575	1	0.030655	1
chr4	13549355	13549496	0.139669	0.419008	132053	99001	99003	3	12	0.007589	1	0.024239	1
chr6	99817927	99817927	0.489642	0.489642	155539	155345	155345	1	1	0.007623	1	0.015797	1
chr21	46677854	46677854	0.489413	0.489413	115614	406618	406618	1	5	0.007636	1	0.015819	1
chr16	86029348	86029348	-0.48917	0.489173	73462	339238	339238	1	2	0.007649	1	0.01584	1
chr17	48994958	48994958	0.489083	0.489083	80821	357317	357317	1	1	0.007654	1	0.015849	1
chr11	65547886	65547924	0.139064	0.417192	33951	252329	252331	3	7	0.007672	1	0.024521	1
chr14	35032169	35032169	0.488241	0.488241	54760	298595	298595	1	1	0.007699	1	0.015927	1
chr15	41625136	41625234	-0.19058	0.381168	61467	312914	312915	2	16	0.007719	1	0.031036	1
chr7	171613	171613	-0.4877	0.487695	160015	164629	164629	1	6	0.007731	1	0.01598	1
chr4	1.09E+08	1.09E+08	-0.48768	0.487683	135353	105535	105535	1	8	0.007732	1	0.015981	1
chr14	93389102	93389246	0.138506	0.415518	58009	305280	305282	3	9	0.007746	1	0.024783	1
chr10	7517765	7517833	0.138201	0.414603	19716	219714	219716	3	3	0.00779	1	0.024927	1
chr2	1.6E+08	1.6E+08	-0.18993	0.379857	105025	60106	60107	2	2	0.007827	1	0.031312	1
chr1	2.13E+08	2.13E+08	-0.13777	0.413303	16175	35966	35968	3	5	0.00785	1	0.025132	1
chr6	29868207	29868221	0.189728	0.379456	151443	140236	140237	2	3	0.00786	1	0.031397	1
chr6	17600994	17600994	-0.48537	0.485369	150312	136488	136488	1	8	0.007862	1	0.016203	1
chr4	1.47E+08	1.47E+08	-0.48513	0.48513	136689	108163	108163	1	1	0.007876	1	0.016226	1
chr15	1.03E+08	1.03E+08	-0.48478	0.484785	66413	323192	323192	1	1	0.007897	1	0.016261	1
chr7	4118583	4118583	0.484693	0.484693	161127	167841	167841	1	4	0.007902	1	0.016269	1
chr5	1.44E+08	1.44E+08	0.137315	0.411944	145956	127557	127559	3	3	0.007913	1	0.025348	1
chr6	1.71E+08	1.71E+08	0.136939	0.410818	159915	164387	164389	3	3	0.007964	1	0.025528	1
chr6	29895116	29895187	-0.13693	0.410792	151448	140270	140272	3	29	0.007966	1	0.025533	1
chr6	1410215	1410436	0.188898	0.377797	148915	133778	133779	2	2	0.00799	1	0.031751	1
chr8	23423757	23423757	0.483107	0.483107	173755	194949	194949	1	2	0.007993	1	0.016422	1
chr4	37246827	37247022	-0.18885	0.37769	132728	100194	100195	2	13	0.007999	1	0.031774	1
chr13	24901961	24902376	-0.18873	0.377451	49388	286817	286818	2	4	0.008019	1	0.031826	1
chr19	23945472	23945659	0.136307	0.408921	91176	383049	383051	3	8	0.008051	1	0.025838	1
chr14	47872288	47872288	0.482084	0.482084	55092	299432	299432	1	1	0.008052	1	0.016519	1
chr7	1.11E+08	1.11E+08	-0.13624	0.408712	167847	182389	182391	3	5	0.008062	1	0.025873	1
chr12	77417567	77417738	-0.18845	0.376908	44572	275885	275886	2	2	0.008065	1	0.031944	1
chr6	1.12E+08	1.12E+08	0.481828	0.481828	156117	156662	156662	1	1	0.008066	1	0.016542	1
chr17	71361857	71361901	-0.13618	0.408534	82305	360446	360448	3	3	0.00807	1	0.025904	1
chr6	27831894	27832098	0.136164	0.408491	151079	138325	138327	3	3	0.008072	1	0.025912	1
chr10	1.3E+08	1.3E+08	-0.13607	0.408195	27367	235601	235603	3	7	0.008085	1	0.02596	1
chr8	48397680	48397680	-0.48144	0.481437	175128	197790	197790	1	1	0.008091	1	0.016582	1
chr10	30663455	30663547	0.188235	0.376471	20964	222367	222368	2	2	0.008103	1	0.032034	1
chr7	2050401	2050401	0.480816	0.480816	160728	166798	166798	1	5	0.008127	1	0.016643	1
chr17	40489569	40489721	0.135694	0.407081	79411	353589	353591	3	5	0.008138	1	0.026153	1
chr7	1.49E+08	1.49E+08	-0.48046	0.480455	169995	186549	186549	1	1	0.008148	1	0.016679	1
chr2	1.5E+08	1.5E+08	-0.18788	0.375761	104645	59408	59409	2	9	0.008163	1	0.032187	1
chr5	1.12E+08	1.12E+08	-0.4801	0.4801	143774	122641	122641	1	1	0.008166	1	0.016711	1
chr4	4117213	4117213	0.480096	0.480096	131166	97011	97011	1	1	0.008166	1	0.016712	1
chr1	1666808	1666808	-0.47993	0.479929	474	1358	1358	1	1	0.008176	1	0.016728	1
chr18	34917603	34917603	0.479833	0.479833	85554	369212	369212	1	1	0.008182	1	0.016737	1
chr6	1.68E+08	1.68E+08	-0.47981	0.479809	159280	162866	162866	1	2	0.008183	1	0.01674	1
chr3	14339534	14339700	0.135319	0.405957	120004	73754	73756	3	3	0.008189	1	0.026345	1
chr3	1.88E+08	1.88E+08	0.479507	0.479507	129274	92536	92536	1	4	0.0082	1	0.016768	1
chr21	38081100	38081100	-0.47944	0.479435	114808	404918	404918	1	9	0.008205	1	0.016775	1
chr17	74023630	74023633	-0.18764	0.375272	82754	361632	361633	2	4	0.008205	1	0.032293	1
chr6	31731881	31731881	0.479354	0.479354	151896	144075	144075	1	9	0.00821	1	0.016783	1
chr3	1.25E+08	1.25E+08	0.479112	0.479112	125682	85096	85096	1	1	0.008224	1	0.016807	1
chr8	26727369	26727369	0.479094	0.479094	173987	195433	195433	1	1	0.008225	1	0.016809	1
chr1	1.56E+08	1.56E+08	0.187421	0.374843	12359	28172	28173	2	8	0.008243	1	0.032386	1
chr5	67383930	67383930	-0.47878	0.478776	141918	118979	118979	1	1	0.008243	1	0.016841	1
chr11	45671015	45671369	-0.18739	0.374779	32002	247578	247579	2	2	0.008248	1	0.0324	1

chr10	1.17E+08	1.17E+08	0.13481	0.40443	26336	233459	233461	3	3	0.008261	1	0.026604	1
chr10	14620934	14620934	-0.47798	0.477985	20155	220664	220664	1	1	0.008292	1	0.016922	1
chr4	3516637	3516692	0.187108	0.374217	131050	96756	96757	2	3	0.008296	1	0.032525	1
chr17	48585216	48585635	0.134251	0.402754	80729	357095	357097	3	10	0.008336	1	0.026899	1
chr7	2883003	2883003	-0.47682	0.476824	161020	167624	167624	1	1	0.008361	1	0.017039	1
chr6	36076933	36076933	-0.47655	0.476549	152743	149643	149643	1	1	0.008378	1	0.017067	1
chr1	1886501	1886543	0.18661	0.373219	544	1540	1541	2	3	0.008384	1	0.032747	1
chr12	3384898	3384898	-0.47553	0.475528	39807	265055	265055	1	3	0.008438	1	0.017168	1
chr7	1886391	1886902	0.133463	0.400389	160642	166518	166520	3	5	0.00845	1	0.027317	1
chr1	6009857	6009857	-0.47514	0.475138	1582	4293	4293	1	1	0.008463	1	0.017209	1
chr11	1.19E+08	1.19E+08	0.474936	0.474936	37903	261304	261304	1	1	0.008475	1	0.017231	1
chr12	25054873	25055108	0.13305	0.39915	41272	268241	268243	3	11	0.008508	1	0.027538	1
chr11	3073408	3073488	-0.13287	0.398608	29429	242128	242130	3	3	0.008532	1	0.027632	1
chr7	1.57E+08	1.57E+08	-0.47383	0.473834	171080	189184	189184	1	2	0.008545	1	0.017346	1
chr11	2286079	2286079	-0.47354	0.473535	29219	241301	241301	1	4	0.008563	1	0.017376	1
chr14	1.03E+08	1.03E+08	-0.47345	0.47345	59030	307597	307597	1	3	0.008568	1	0.017385	1
chr8	74903761	74903801	0.185527	0.371054	176362	200359	200360	2	2	0.008583	1	0.033233	1
chr9	90621637	90622100	-0.18535	0.370707	182191	211476	211477	2	3	0.008616	1	0.033314	1
chr22	50482250	50482250	-0.47249	0.472489	118902	414096	414096	1	2	0.008626	1	0.017484	1
chr13	28553783	28553805	-0.13206	0.396179	49671	287513	287515	3	5	0.008641	1	0.028078	1
chr14	1E+08	1E+08	0.47179	0.47179	58558	306363	306363	1	1	0.008672	1	0.017557	1
chr13	1.11E+08	1.11E+08	-0.47101	0.471014	52880	293818	293818	1	1	0.008721	1	0.01764	1
chr8	43132256	43132507	-0.13144	0.394324	175049	197620	197622	3	9	0.008727	1	0.028428	1
chr19	55735946	55735946	0.470659	0.470659	95023	392813	392813	1	1	0.008744	1	0.017677	1
chr18	77508326	77508326	0.47061	0.47061	86745	371861	371861	1	1	0.008747	1	0.017682	1
chr15	78556940	78556945	0.184653	0.369306	64521	319275	319276	2	14	0.008747	1	0.033633	1
chr8	1.09E+08	1.09E+08	-0.47046	0.470455	177874	203258	203258	1	1	0.008757	1	0.017698	1
chr11	6804983	6804983	-0.47036	0.470362	29830	243029	243029	1	1	0.008764	1	0.017709	1
chr1	40257195	40257195	-0.4701	0.4701	6330	14589	14589	1	1	0.00878	1	0.017737	1
chr17	27346926	27347092	0.130734	0.392202	77881	349966	349968	3	5	0.008824	1	0.028829	1
chr11	1.24E+08	1.24E+08	0.469159	0.469159	38351	262125	262125	1	1	0.008839	1	0.017831	1
chr1	2801984	2801984	0.468438	0.468438	946	2665	2665	1	5	0.008884	1	0.017907	1
chr7	1.55E+08	1.55E+08	0.130242	0.390726	170744	188271	188273	3	5	0.008891	1	0.029116	1
chr10	2357283	2357363	-0.18388	0.367768	19223	218734	218735	2	3	0.008895	1	0.033992	1
chr4	1.4E+08	1.4E+08	0.183773	0.367545	136342	107438	107439	2	2	0.008916	1	0.034047	1
chr17	45401733	45401833	-0.18369	0.367386	80159	355559	355560	2	9	0.00893	1	0.034082	1
chr10	1.32E+08	1.32E+08	-0.46756	0.467556	27631	236386	236386	1	1	0.008943	1	0.018003	1
chr18	58996785	58996785	0.467465	0.467465	86082	370484	370484	1	1	0.008949	1	0.018012	1
chr11	47235900	47236131	0.129623	0.388869	32266	248172	248174	3	10	0.008977	1	0.029479	1
chr1	16678832	16678847	0.183381	0.366762	3316	7791	7792	2	13	0.008993	1	0.034229	1
chr1	1.11E+08	1.11E+08	-0.12942	0.388257	10329	23292	23294	3	12	0.009005	1	0.029601	1
chr1	1.55E+08	1.55E+08	-0.18327	0.366543	12148	27567	27568	2	10	0.009013	1	0.034279	1
chr22	18268062	18268249	0.129243	0.387729	116045	407439	407441	3	5	0.009028	1	0.029704	1
chr6	1.7E+08	1.7E+08	0.466163	0.466163	159767	164001	164001	1	1	0.009032	1	0.018151	1
chr7	63505768	63505791	-0.18318	0.366361	164799	175663	175664	2	8	0.009033	1	0.034325	1
chr6	32116853	32116875	-0.12913	0.38738	152022	145634	145636	3	49	0.009045	1	0.029773	1
chr15	49716247	49716645	-0.12901	0.38704	62032	314245	314247	3	3	0.009061	1	0.029841	1
chr10	13424911	13425201	0.12874	0.386219	20043	220467	220469	3	5	0.009098	1	0.030004	1
chr12	1.26E+08	1.26E+08	-0.46492	0.464922	47842	283108	283108	1	5	0.009112	1	0.018285	1
chr19	49713828	49714044	-0.12859	0.385785	93926	390018	390020	3	5	0.009118	1	0.030095	1
chr10	77542488	77542585	0.128519	0.385558	23544	227462	227464	3	9	0.009128	1	0.03014	1
chr16	1121907	1122047	0.18269	0.36538	66879	324551	324552	2	2	0.009133	1	0.03456	1
chr1	29190137	29190137	0.464269	0.464269	5134	11883	11883	1	7	0.009154	1	0.018356	1
chr6	1.69E+08	1.69E+08	-0.46419	0.464191	159632	163712	163712	1	3	0.009159	1	0.018363	1
chr12	214194	214224	0.182515	0.365029	39403	264255	264256	2	4	0.009167	1	0.034644	1
chr6	1.57E+08	1.57E+08	0.464053	0.464053	158377	160974	160974	1	2	0.009168	1	0.018378	1
chr6	1.71E+08	1.71E+08	0.182417	0.364835	159884	164290	164291	2	2	0.009187	1	0.034692	1
chr1	1555396	1555396	-0.46356	0.463563	428	1248	1248	1	1	0.0092	1	0.01843	1
chr2	1.69E+08	1.69E+08	0.463085	0.463085	105397	60790	60790	1	5	0.009233	1	0.018484	1
chr4	3495242	3495341	0.182098	0.364196	131036	96733	96734	2	2	0.009251	1	0.034843	1
chr11	4600034	4600034	-0.46273	0.462733	29581	242518	242518	1	3	0.009254	1	0.018521	1
chr19	40995921	40996118	-0.12749	0.38247	92409	386247	386249	3	4	0.009265	1	0.030767	1
chr11	69919554	69920054	0.12704	0.381119	34815	254746	254748	3	5	0.009323	1	0.031047	1
chr5	1.78E+08	1.78E+08	0.461182	0.461182	148321	132393	132393	1	4	0.009359	1	0.01869	1
chr18	34327498	34327498	-0.46114	0.461139	85528	369157	369157	1	1	0.009362	1	0.018695	1
chr12	1.26E+08	1.26E+08	0.461126	0.461126	47799	283029	283029	1	1	0.009363	1	0.018696	1
chr3	1.26E+08	1.26E+08	-0.1267	0.380089	125752	85236	85238	3	6	0.009369	1	0.031265	1
chr2	47115827	47115827	0.460814	0.460814	99133	48362	48362	1	1	0.009383	1	0.01873	1
chr20	44420225	44420276	-0.18142	0.362848	112707	399880	399881	2	15	0.009384	1	0.035164	1
chr15	93208459	93208920	-0.1814	0.36281	65721	321768	321769	2	2	0.009388	1	0.035174	1
chr2	1.82E+08	1.82E+08	0.460554	0.460554	106277	62788	62788	1	1	0.009403	1	0.01876	1
chr8	1.44E+08	1.44E+08	0.180902	0.361804	179824	206843	206844	2	11	0.009494	1	0.035421	1
chr10	695859	695900	0.125659	0.376976	18941	218056	218058	3	11	0.009508	1	0.031931	1
chr10	1.34E+08	1.34E+08	-0.45897	0.458966	27936	237265	237265	1	5	0.009513	1	0.01894	1

chr3	1.01E+08	1.01E+08	0.125613	0.376838	124508	82894	82896	3	15	0.009514	1	0.031959	1
chr5	14230166	14230166	0.458842	0.458842	140003	115323	115323	1	1	0.009523	1	0.018955	1
chr1	1.57E+08	1.57E+08	0.180757	0.361514	12448	28416	28417	2	2	0.009524	1	0.035491	1
chr17	7387573	7387703	0.125457	0.37637	76053	345755	345757	3	12	0.009533	1	0.032056	1
chr1	47900630	47900630	0.458448	0.458448	7252	16812	16812	1	5	0.009549	1	0.018999	1
chr2	1.28E+08	1.28E+08	0.180623	0.361246	103604	57574	57575	2	4	0.009552	1	0.035555	1
chr1	1.09E+08	1.09E+08	-0.45836	0.458357	10038	22559	22559	1	1	0.009557	1	0.01901	1
chr8	6633067	6633067	-0.45806	0.45806	172380	192322	192322	1	2	0.009578	1	0.019042	1
chr4	16077807	16077807	-0.45798	0.457977	132171	99219	99219	1	4	0.009583	1	0.019052	1
chr11	1.32E+08	1.32E+08	-0.45795	0.457951	39076	263578	263578	1	1	0.009585	1	0.019055	1
chr11	1.34E+08	1.34E+08	0.457676	0.457676	39229	263844	263844	1	1	0.009606	1	0.019086	1
chr6	32547167	32547167	0.457589	0.457589	152146	146417	146417	1	1	0.009612	1	0.019096	1
chr2	95454741	95454741	0.457535	0.457535	101532	53405	53405	1	2	0.009616	1	0.019103	1
chr5	1.4E+08	1.4E+08	-0.45743	0.457434	145443	126224	126224	1	1	0.009623	1	0.019114	1
chr19	37894644	37894856	-0.1247	0.374095	91922	385014	385016	3	3	0.009629	1	0.032552	1
chr7	91764530	91764605	-0.12465	0.373941	166268	178616	178618	3	20	0.009635	1	0.032582	1
chr10	1.32E+08	1.32E+08	0.180114	0.360227	27607	236292	236293	2	2	0.009658	1	0.035805	1
chr1	2.46E+08	2.46E+08	0.456774	0.456774	18478	40604	40604	1	3	0.009667	1	0.01919	1
chr2	37571729	37571732	0.180027	0.360054	98398	46990	46991	2	10	0.009675	1	0.035846	1
chr12	66459807	66459807	-0.45645	0.456447	44082	274926	274926	1	1	0.009693	1	0.019231	1
chr20	61751933	61751933	-0.45641	0.456411	113835	402670	402670	1	1	0.009696	1	0.019235	1
chr8	1.22E+08	1.22E+08	0.456363	0.456363	178288	203997	203997	1	14	0.009699	1	0.019241	1
chr5	1.79E+08	1.79E+08	0.124036	0.372107	148360	132484	132486	3	6	0.009711	1	0.032997	1
chr19	44493005	44493005	0.455841	0.455841	92928	387530	387530	1	1	0.009737	1	0.019302	1
chr4	88755207	88755207	0.455628	0.455628	134747	104328	104328	1	1	0.009751	1	0.019326	1
chr7	1.29E+08	1.29E+08	-0.45479	0.454785	168723	184109	184109	1	1	0.009811	1	0.019424	1
chr7	56551524	56551640	-0.12298	0.368941	164616	175337	175339	3	8	0.00984	1	0.033718	1
chr17	58179869	58180026	0.1789	0.3578	81361	358481	358482	2	5	0.009918	1	0.03641	1
chr8	2002012	2002012	0.453269	0.453269	172198	191994	191994	1	1	0.00992	1	0.019603	1
chr17	146670	146670	-0.45322	0.453221	74794	342436	342436	1	5	0.009923	1	0.019609	1
chr21	44782434	44782497	0.122273	0.36682	115264	405881	405883	3	4	0.009926	1	0.034215	1
chr10	79622515	79622515	-0.45311	0.453115	23677	227636	227636	1	1	0.00993	1	0.01962	1
chr19	12750806	12751042	-0.12216	0.366489	89713	379200	379202	3	5	0.009939	1	0.034292	1
chr7	1.59E+08	1.59E+08	-0.12209	0.366265	171549	190449	190451	3	7	0.009946	1	0.034349	1
chr20	61162099	61162099	0.452011	0.452011	113689	402320	402320	1	6	0.010006	1	0.019748	1
chr7	187333	187686	0.121337	0.364011	160024	164649	164651	3	4	0.010036	1	0.034887	1
chr17	6899359	6899380	-0.12127	0.36382	75902	345190	345192	3	15	0.010043	1	0.034934	1
chr1	49242519	49242757	-0.12126	0.363781	7339	16995	16997	3	6	0.010045	1	0.034943	1
chr6	1.71E+08	1.71E+08	-0.45098	0.450977	159945	164472	164472	1	1	0.010079	1	0.019868	1
chr11	1.34E+08	1.34E+08	0.178122	0.356243	39232	263847	263848	2	2	0.010093	1	0.036814	1
chr2	39470725	39470750	0.120813	0.362438	98563	47285	47287	3	5	0.010095	1	0.035265	1
chr8	68624190	68624190	0.450533	0.450533	176088	199780	199780	1	1	0.010111	1	0.019922	1
chr1	2.1E+08	2.1E+08	0.450242	0.450242	15992	35594	35594	1	1	0.010134	1	0.019958	1
chr10	49765381	49765381	-0.45017	0.450169	22006	224300	224300	1	1	0.010138	1	0.019967	1
chr6	32765109	32765117	-0.17788	0.355766	152177	146519	146520	2	13	0.010148	1	0.036939	1
chr16	83967545	83967808	-0.17787	0.355741	72957	338143	338144	2	3	0.010151	1	0.036946	1
chr9	25677495	25678015	0.120261	0.360784	181055	209698	209700	3	5	0.010152	1	0.035669	1
chr1	8120055	8120055	0.449232	0.449232	2027	5273	5273	1	1	0.010208	1	0.02008	1
chr1	43472312	43472312	-0.44923	0.449232	6701	15424	15424	1	2	0.010208	1	0.02008	1
chr6	52690013	52690013	0.449208	0.449208	154180	152584	152584	1	1	0.010209	1	0.020084	1
chr3	1.4E+08	1.4E+08	0.448792	0.448792	126891	87711	87711	1	1	0.010239	1	0.020133	1
chr16	2334163	2334163	-0.44861	0.448609	67430	326129	326129	1	4	0.010252	1	0.020155	1
chr18	11147146	11147785	-0.11928	0.35785	84937	367691	367693	3	3	0.010257	1	0.036398	1
chr11	70672740	70672841	0.119158	0.357473	34949	255113	255115	3	10	0.010271	1	0.036494	1
chr2	69812277	69812277	0.448227	0.448227	100311	50642	50642	1	1	0.01028	1	0.020201	1
chr6	33290873	33290873	0.447927	0.447927	152323	148370	148370	1	44	0.010302	1	0.020237	1
chr6	1.64E+08	1.64E+08	-0.11885	0.356546	158973	162134	162136	3	3	0.010304	1	0.036735	1
chr9	2234921	2234921	-0.4478	0.447801	180624	209059	209059	1	1	0.010311	1	0.020253	1
chr17	36457564	36457564	-0.44773	0.447725	78661	351740	351740	1	1	0.010317	1	0.020263	1
chr8	11327014	11327014	0.447497	0.447497	172853	193184	193184	1	1	0.010333	1	0.020291	1
chr8	1.44E+08	1.44E+08	0.447394	0.447394	180008	207321	207321	1	4	0.010342	1	0.020304	1
chr19	58879022	58879059	-0.11846	0.355377	95554	394315	394317	3	5	0.010345	1	0.037045	1
chr1	2.46E+08	2.46E+08	0.447274	0.447274	18489	40629	40629	1	1	0.010351	1	0.020319	1
chr4	4763360	4763596	-0.11838	0.355146	131255	97199	97201	3	6	0.010354	1	0.037108	1
chr1	567206	567348	0.118378	0.355134	14	17	19	3	9	0.010354	1	0.037111	1
chr19	17015427	17015427	-0.44718	0.447175	90406	381030	381030	1	2	0.010358	1	0.020331	1
chr3	45077649	45077765	-0.17688	0.353769	121614	76952	76953	2	9	0.010386	1	0.037472	1
chr15	99709980	99709980	0.446766	0.446766	66109	322576	322576	1	1	0.01039	1	0.020383	1
chr6	1.67E+08	1.67E+08	-0.44663	0.446635	159246	162779	162779	1	3	0.010399	1	0.020398	1
chr3	75704498	75704824	-0.11772	0.353164	123997	81952	81954	3	5	0.010419	1	0.037636	1
chr6	1.71E+08	1.71E+08	0.117244	0.351733	159904	164352	164354	3	9	0.010464	1	0.038014	1
chr6	424126	424272	-0.17645	0.352905	148795	133526	133527	2	4	0.01049	1	0.037702	1
chr1	1.55E+08	1.55E+08	0.176437	0.352874	12104	27426	27427	2	3	0.010493	1	0.03771	1
chr17	36665844	36666462	-0.11669	0.350066	78697	351817	351819	3	8	0.010518	1	0.038467	1

chr2	2.42E+08	2.42E+08	-0.44512	0.445125	110250	70924	70924	1	4	0.010519	1	0.020593	1
chr21	43547872	43547872	-0.44489	0.444886	115115	405570	405570	1	4	0.010537	1	0.020623	1
chr6	1620687	1621093	-0.11647	0.349409	148956	133893	133895	3	3	0.010538	1	0.038648	1
chr10	1.35E+08	1.35E+08	-0.44479	0.444794	28409	238827	238827	1	4	0.010544	1	0.020635	1
chr3	1.19E+08	1.19E+08	0.444561	0.444561	125237	84215	84215	1	1	0.010563	1	0.020664	1
chr19	30363203	30363526	0.116037	0.348112	91264	383247	383249	3	4	0.010576	1	0.039001	1
chr6	67741651	67741714	-0.17594	0.351881	154573	153251	153252	2	2	0.010614	1	0.037976	1
chr15	1E+08	1E+08	-0.44369	0.443688	66158	322687	322687	1	1	0.010631	1	0.020777	1
chr6	32336160	32336160	0.443024	0.443024	152100	146280	146280	1	1	0.010685	1	0.020862	1
chr4	68622534	68622534	0.442967	0.442967	133773	102389	102389	1	2	0.01069	1	0.02087	1
chr19	19977659	19977733	0.175614	0.351227	91021	382662	382663	2	2	0.010696	1	0.038152	1
chr4	81111177	81111527	-0.11445	0.343349	134378	103522	103524	3	3	0.010705	1	0.040348	1
chr16	32127656	32127659	-0.17554	0.351078	70246	332363	332364	2	4	0.010715	1	0.03819	1
chr6	29794657	29794657	0.442587	0.442587	151430	140167	140167	1	5	0.010721	1	0.02092	1
chr11	1.2E+08	1.2E+08	0.442515	0.442515	37967	261413	261413	1	13	0.010727	1	0.02093	1
chr15	67361773	67361773	-0.44251	0.442511	63337	316849	316849	1	1	0.010727	1	0.020931	1
chr1	1.8E+08	1.8E+08	0.114116	0.342349	14076	31808	31810	3	14	0.010732	1	0.040638	1
chr20	62948037	62948134	-0.17544	0.350877	114221	403534	403535	2	3	0.010741	1	0.038245	1
chr6	1.5E+08	1.5E+08	-0.11364	0.340905	157945	160091	160093	3	10	0.010769	1	0.041063	1
chr2	2.33E+08	2.33E+08	0.441718	0.441718	109042	68322	68322	1	4	0.010789	1	0.021031	1
chr1	2.42E+08	2.42E+08	-0.44155	0.441548	18202	40069	40069	1	1	0.010802	1	0.021052	1
chr10	99338074	99338188	-0.17516	0.350326	25005	230279	230280	2	4	0.010809	1	0.038396	1
chr17	12927897	12927897	0.441434	0.441434	76647	347228	347228	1	3	0.01081	1	0.021066	1
chr5	1.4E+08	1.4E+08	-0.17511	0.350219	145486	126366	126367	2	5	0.010823	1	0.038427	1
chr2	8530521	8530521	0.440631	0.440631	96438	43021	43021	1	1	0.010872	1	0.021167	1
chr16	930179	930179	-0.44052	0.44052	66771	324255	324255	1	9	0.010882	1	0.021183	1
chr22	42016856	42016984	-0.17483	0.349661	118057	412189	412190	2	14	0.010893	1	0.038578	1
chr10	1.35E+08	1.35E+08	-0.44034	0.440339	28241	238296	238296	1	3	0.010897	1	0.021206	1
chr19	1523917	1524449	0.111564	0.334693	87389	373415	373417	3	6	0.010908	1	0.042952	1
chr1	26233376	26233435	0.111159	0.333477	4661	10622	10624	3	16	0.010932	1	0.043336	1
chr7	1.57E+08	1.57E+08	0.111007	0.333022	171170	189490	189492	3	3	0.01094	1	0.043485	1
chr20	62053198	62053198	0.439724	0.439724	113932	402890	402890	1	3	0.010946	1	0.021287	1
chr20	47253042	47253042	0.439626	0.439626	112911	400396	400396	1	3	0.010953	1	0.021299	1
chr3	1.34E+08	1.34E+08	-0.11079	0.332363	126552	86920	86922	3	5	0.010954	1	0.043699	1
chr3	1.18E+08	1.18E+08	0.439491	0.439491	125159	84061	84061	1	1	0.010963	1	0.021315	1
chr10	1.27E+08	1.27E+08	0.174531	0.349061	27182	235292	235293	2	11	0.01097	1	0.038745	1
chr18	54306261	54306437	0.174479	0.348957	85940	370151	370152	2	12	0.010982	1	0.038772	1
chr20	61160996	61160996	-0.43891	0.438914	113688	402315	402315	1	3	0.011007	1	0.021391	1
chr10	6205688	6205688	0.438883	0.438883	19616	219523	219523	1	2	0.01101	1	0.021395	1
chr2	263559	264120	-0.10936	0.328067	95645	41298	41300	3	5	0.011021	1	0.045105	1
chr11	46321235	46321235	0.438482	0.438482	32133	247886	247886	1	1	0.011043	1	0.021449	1
chr1	1.81E+08	1.81E+08	-0.43836	0.438361	14223	32131	32131	1	1	0.011053	1	0.021465	1
chr6	32145830	32145904	-0.10779	0.323362	152033	145891	145893	3	38	0.011081	1	0.046699	1
chr10	1.03E+08	1.03E+08	-0.17406	0.348113	25388	231279	231280	2	7	0.01109	1	0.039001	1
chr13	1.11E+08	1.11E+08	0.105232	0.315695	52925	293926	293928	3	3	0.011141	1	0.049478	1
chr4	1.84E+08	1.84E+08	-0.43726	0.437256	138034	110802	110802	1	1	0.011144	1	0.021612	1
chr1	16942481	16942566	0.173838	0.347675	3366	7892	7893	2	2	0.011147	1	0.039124	1
chr7	1.5E+08	1.5E+08	-0.43721	0.437207	170176	186952	186952	1	6	0.011148	1	0.021618	1
chr1	1015392	1015447	0.173813	0.347627	155	425	426	2	3	0.011154	1	0.039138	1
chr4	40267141	40267141	-0.43699	0.436989	132931	100641	100641	1	1	0.011164	1	0.021646	1
chr21	45753273	45753273	-0.43697	0.436966	115443	406260	406260	1	5	0.011166	1	0.021649	1
chr6	10837647	10837710	0.173717	0.347433	149747	135505	135506	2	2	0.011178	1	0.039192	1
chr3	99595033	99595145	0.173693	0.347386	124393	82666	82667	2	5	0.011185	1	0.039206	1
chr17	39094308	39094308	0.435616	0.435616	79129	352937	352937	1	3	0.011275	1	0.021832	1
chr11	1.24E+08	1.24E+08	0.435298	0.435298	38372	262159	262159	1	2	0.011302	1	0.021878	1
chr19	2858854	2858944	-0.17324	0.346471	87858	374502	374503	2	4	0.011306	1	0.039463	1
chr5	1.8E+08	1.8E+08	-0.43511	0.435114	148686	133218	133218	1	1	0.011318	1	0.021902	1
chr16	2848577	2848797	0.173176	0.346352	67577	326473	326474	2	11	0.011323	1	0.039499	1
chr11	1874291	1874291	0.434892	0.434892	29087	240809	240809	1	7	0.011336	1	0.021932	1
chr6	32289357	32289357	0.434798	0.434798	152077	146228	146228	1	2	0.011344	1	0.021946	1
chr11	66579670	66579670	0.434603	0.434603	34198	253123	253123	1	1	0.011361	1	0.021973	1
chr6	29442830	29442830	-0.43429	0.434292	151337	139601	139601	1	1	0.011386	1	0.022015	1
chr2	2.01E+08	2.01E+08	0.434198	0.434198	106929	64085	64085	1	1	0.011394	1	0.022028	1
chr10	1.32E+08	1.32E+08	0.433385	0.433385	27642	236420	236420	1	3	0.011461	1	0.022136	1
chr19	4277322	4277333	-0.17262	0.345247	88273	375458	375459	2	2	0.011464	1	0.039809	1
chr1	2.33E+08	2.33E+08	-0.43313	0.433129	17684	39056	39056	1	1	0.011481	1	0.022171	1
chr19	51107558	51107560	0.172562	0.345124	94228	390815	390816	2	4	0.011481	1	0.039842	1
chr10	1.35E+08	1.35E+08	-0.17254	0.345085	28425	238877	238878	2	8	0.011485	1	0.039853	1
chr8	1.33E+08	1.33E+08	0.432825	0.432825	178952	205077	205077	1	2	0.011508	1	0.022213	1
chr2	9188970	9188970	-0.4323	0.432304	96512	43174	43174	1	1	0.011552	1	0.022284	1
chr4	24796803	24796803	0.432139	0.432139	132461	99714	99714	1	8	0.011566	1	0.022307	1
chr7	36655667	36655667	-0.43138	0.431385	163307	172774	172774	1	1	0.011628	1	0.02241	1
chr2	2.36E+08	2.36E+08	-0.43071	0.430707	109385	69019	69019	1	1	0.011687	1	0.022506	1
chr2	11485552	11485561	0.171772	0.343543	96815	43782	43783	2	4	0.011687	1	0.040289	1

chr12	1.26E+08	1.26E+08	0.430208	0.430208	47806	283046	283046	1	3	0.01173	1	0.022575	1
chr15	40572137	40572326	-0.17159	0.343178	61298	312457	312458	2	5	0.011735	1	0.040398	1
chr5	759353	759740	-0.17159	0.343172	138884	112673	112674	2	8	0.011736	1	0.040399	1
chr18	713085	713200	-0.17158	0.343153	84668	367011	367012	2	8	0.011739	1	0.040405	1
chr1	41350014	41350183	0.171523	0.343046	6465	14924	14925	2	3	0.011753	1	0.040435	1
chr12	1.33E+08	1.33E+08	0.429379	0.429379	48713	285257	285257	1	3	0.011803	1	0.022694	1
chr14	1.02E+08	1.02E+08	-0.42904	0.429037	58905	307280	307280	1	17	0.01183	1	0.02274	1
chr5	1.4E+08	1.4E+08	-0.17121	0.342418	145463	126294	126295	2	4	0.011837	1	0.040617	1
chr6	28185726	28185726	0.428757	0.428757	151123	138495	138495	1	1	0.011854	1	0.02278	1
chr19	289902	289902	-0.42868	0.428683	86858	372093	372093	1	1	0.011861	1	0.02279	1
chr19	11517079	11517152	-0.1709	0.341803	89535	378644	378645	2	5	0.011924	1	0.040798	1
chr5	1.41E+08	1.41E+08	-0.17068	0.34136	145546	126576	126577	2	7	0.011987	1	0.040931	1
chr5	1.41E+08	1.41E+08	-0.17068	0.341358	145565	126636	126637	2	4	0.011987	1	0.040931	1
chr4	1.9E+08	1.9E+08	-0.42689	0.426893	138582	111953	111953	1	1	0.012021	1	0.023049	1
chr2	97778927	97778927	-0.4265	0.4265	101795	54033	54033	1	11	0.012056	1	0.023109	1
chr6	29648736	29648901	-0.17029	0.340576	151396	139951	139952	2	11	0.012096	1	0.041161	1
chr1	1.59E+08	1.59E+08	0.42584	0.42584	12715	28964	28964	1	1	0.012114	1	0.023202	1
chr21	15353879	15354232	0.170214	0.340427	114276	403633	403634	2	2	0.012117	1	0.041204	1
chr3	1.13E+08	1.13E+08	-0.42572	0.425715	124932	83662	83662	1	1	0.012125	1	0.02322	1
chr1	2.29E+08	2.29E+08	0.170174	0.340348	17349	38429	38430	2	3	0.012127	1	0.041228	1
chr10	1.31E+08	1.31E+08	0.425645	0.425645	27539	236048	236048	1	5	0.012131	1	0.02323	1
chr19	50391354	50391554	-0.17011	0.340226	94097	390505	390506	2	4	0.012143	1	0.041263	1
chr12	1.14E+08	1.14E+08	0.425294	0.425294	46542	279956	279956	1	3	0.012163	1	0.023283	1
chr15	29980500	29980500	-0.42529	0.425291	60620	311184	311184	1	3	0.012163	1	0.023284	1
chr7	3134557	3134592	0.169996	0.339992	161055	167717	167718	2	2	0.012177	1	0.041334	1
chr7	1.58E+08	1.58E+08	-0.42496	0.424956	171453	190228	190228	1	3	0.012194	1	0.023334	1
chr14	1.06E+08	1.06E+08	0.424729	0.424729	59811	309471	309471	1	1	0.012216	1	0.023367	1
chr12	12225262	12225262	-0.42472	0.424718	40720	267131	267131	1	1	0.012217	1	0.023369	1
chr17	12922839	12922839	-0.42439	0.42439	76646	347226	347226	1	1	0.012246	1	0.023417	1
chr11	17034644	17034644	0.424275	0.424275	30659	244639	244639	1	2	0.012256	1	0.023434	1
chr22	17516125	17516125	0.424148	0.424148	115970	407277	407277	1	1	0.012269	1	0.023454	1
chr15	62899159	62899159	0.424011	0.424011	62793	315768	315768	1	1	0.012282	1	0.023475	1
chr13	1.12E+08	1.12E+08	0.423493	0.423493	53048	294208	294208	1	6	0.012326	1	0.023551	1
chr15	67323243	67323243	0.423482	0.423482	63327	316829	316829	1	1	0.012327	1	0.023552	1
chr6	33083457	33083457	0.42335	0.42335	152255	147227	147227	1	4	0.012339	1	0.023572	1
chr13	27050512	27050512	0.423313	0.423313	49529	287144	287144	1	1	0.012342	1	0.023577	1
chr19	43383986	43383986	0.422702	0.422702	92797	387195	387195	1	3	0.0124	1	0.02367	1
chr19	626119	626119	0.422178	0.422178	86999	372392	372392	1	1	0.01245	1	0.023748	1
chr16	90143751	90143788	-0.16886	0.33773	74732	342307	342308	2	5	0.012504	1	0.04202	1
chr4	720809	720870	-0.16883	0.337666	130240	94554	94555	2	2	0.012513	1	0.042038	1
chr21	45393541	45393541	-0.42136	0.421363	115367	406098	406098	1	3	0.012524	1	0.02387	1
chr2	214148	214171	-0.16879	0.337582	95636	41287	41288	2	4	0.012525	1	0.042063	1
chr5	1.1E+08	1.1E+08	0.421175	0.421175	143669	122430	122430	1	11	0.012542	1	0.0239	1
chr16	66613273	66613278	0.16862	0.337241	71578	335047	335048	2	13	0.012574	1	0.042168	1
chr14	58762197	58762197	0.420713	0.420713	55761	300785	300785	1	1	0.012585	1	0.02397	1
chr3	49170599	49170668	0.168461	0.336921	122129	78215	78216	2	10	0.012621	1	0.042267	1
chr2	2.2E+08	2.2E+08	0.168449	0.336897	108293	66826	66827	2	2	0.012625	1	0.042274	1
chr7	21301723	21301723	-0.42008	0.420081	162148	170190	170190	1	1	0.012643	1	0.024068	1
chr20	60958108	60958108	-0.41992	0.419917	113650	402194	402194	1	1	0.01266	1	0.024093	1
chr11	33797161	33797161	0.419611	0.419611	31458	246499	246499	1	2	0.012689	1	0.024143	1
chr17	54678114	54678114	-0.41884	0.418842	80990	357666	357666	1	1	0.012766	1	0.024265	1
chr10	35504007	35504007	-0.41816	0.418156	21306	222941	222941	1	4	0.01283	1	0.024369	1
chr20	170641	170641	-0.418	0.418004	110659	394565	394565	1	5	0.012844	1	0.024391	1
chr16	5482346	5482346	0.417995	0.417995	68143	327854	327854	1	1	0.012844	1	0.024393	1
chr4	1.55E+08	1.55E+08	0.417623	0.417623	137117	108957	108957	1	1	0.01288	1	0.024449	1
chr6	29942706	29942706	-0.41749	0.417489	151460	140302	140302	1	26	0.012893	1	0.024472	1
chr10	27608703	27608719	-0.16754	0.33509	20762	221998	221999	2	2	0.012899	1	0.042827	1
chr10	1.32E+08	1.32E+08	0.167512	0.335023	27622	236364	236365	2	9	0.01291	1	0.042848	1
chr10	45374841	45374906	0.167461	0.334923	21725	223793	223794	2	5	0.012924	1	0.042878	1
chr8	1.34E+08	1.34E+08	0.416946	0.416946	179042	205219	205219	1	1	0.012946	1	0.024557	1
chr3	1.96E+08	1.96E+08	0.167164	0.334329	129933	93778	93779	2	4	0.013017	1	0.043066	1
chr8	1.26E+08	1.26E+08	-0.41614	0.416136	178534	204475	204475	1	1	0.013025	1	0.024684	1
chr12	1.33E+08	1.33E+08	0.415826	0.415826	48649	285037	285037	1	4	0.013057	1	0.024735	1
chr2	1625268	1625268	-0.41554	0.415545	95896	41871	41871	1	5	0.013084	1	0.024779	1
chr1	37732046	37732046	0.41537	0.41537	6017	13839	13839	1	1	0.013099	1	0.024806	1
chr13	1.11E+08	1.11E+08	-0.41517	0.415169	52878	293816	293816	1	1	0.013117	1	0.024836	1
chr7	1.57E+08	1.57E+08	-0.41516	0.41516	171167	189481	189481	1	12	0.013117	1	0.024837	1
chr5	1.41E+08	1.41E+08	-0.1668	0.333607	145635	126944	126945	2	4	0.01313	1	0.043295	1
chr6	31324921	31324941	0.166786	0.333572	151783	142902	142903	2	9	0.013135	1	0.043307	1
chr12	1.33E+08	1.33E+08	-0.41497	0.414972	48549	284713	284713	1	3	0.013136	1	0.024868	1
chr6	1.43E+08	1.43E+08	-0.16674	0.33349	157561	159378	159379	2	2	0.013147	1	0.043332	1
chr1	10702712	10702712	-0.41472	0.414721	2496	6168	6168	1	1	0.013162	1	0.024908	1
chr12	1.31E+08	1.31E+08	0.414714	0.414714	48197	283874	283874	1	4	0.013162	1	0.024909	1
chr3	1.96E+08	1.96E+08	-0.16639	0.332777	129814	93506	93507	2	2	0.013261	1	0.043566	1

chr2	2.34E+08	2.34E+08	0.41365	0.41365	109143	68572	68572	1	12	0.013264	1	0.025076	1
chr19	55667663	55668066	0.165677	0.331355	95004	392759	392760	2	6	0.013487	1	0.044022	1
chr2	1.76E+08	1.76E+08	0.165664	0.331328	105954	61925	61926	2	9	0.013491	1	0.04403	1
chr7	1986301	1986334	0.165629	0.331259	160698	166679	166680	2	7	0.013502	1	0.044053	1
chr11	6502655	6502690	-0.16555	0.331109	29790	242923	242924	2	11	0.013526	1	0.044101	1
chr1	11046185	11046185	0.410826	0.410826	2600	6364	6364	1	1	0.013542	1	0.025527	1
chr6	32605704	32605704	0.410013	0.410013	152155	146430	146430	1	1	0.013622	1	0.025657	1
chr7	1.34E+08	1.34E+08	-0.40962	0.409623	169105	184846	184846	1	1	0.013663	1	0.025722	1
chr13	28562900	28562900	0.409484	0.409484	49674	287527	287527	1	7	0.013678	1	0.025747	1
chr2	16484904	16484904	-0.40935	0.409354	97058	44212	44212	1	1	0.013691	1	0.025768	1
chr14	1.04E+08	1.04E+08	0.409277	0.409277	59225	308066	308066	1	1	0.0137	1	0.025781	1
chr10	1251771	1251771	-0.40906	0.409057	19078	218398	218398	1	6	0.013722	1	0.025817	1
chr3	1.25E+08	1.25E+08	0.16496	0.32992	125700	85126	85127	2	3	0.013725	1	0.044496	1
chr3	1.05E+08	1.05E+08	0.408938	0.408938	124577	83043	83043	1	1	0.013733	1	0.025836	1
chr11	28642652	28642652	0.408821	0.408821	31213	245873	245873	1	1	0.013745	1	0.025855	1
chr1	2710376	2710376	-0.40882	0.408815	907	2579	2579	1	1	0.013746	1	0.025856	1
chr12	1.31E+08	1.31E+08	-0.16489	0.32978	48177	283818	283819	2	4	0.013748	1	0.044543	1
chr6	1.11E+08	1.11E+08	0.164765	0.329529	156064	156525	156526	2	2	0.013787	1	0.044622	1
chr15	29034942	29034950	-0.16475	0.329498	60540	311028	311029	2	2	0.013792	1	0.044631	1
chr3	14258049	14258049	-0.40813	0.408125	119991	73741	73741	1	1	0.013815	1	0.025971	1
chr10	1.22E+08	1.22E+08	-0.40812	0.408118	26716	234306	234306	1	2	0.013816	1	0.025972	1
chr16	66458043	66458043	-0.40727	0.407272	71549	334977	334977	1	1	0.013906	1	0.026119	1
chr19	427162	427263	0.164388	0.328777	86903	372201	372202	2	3	0.013913	1	0.04487	1
chr3	52728804	52728804	0.407162	0.407162	122643	79588	79588	1	1	0.013918	1	0.026138	1
chr12	1.15E+08	1.15E+08	-0.40714	0.407135	46653	280296	280296	1	1	0.013921	1	0.026144	1
chr16	34257170	34257432	-0.16414	0.328279	70344	332523	332524	2	7	0.013997	1	0.045035	1
chr3	1.72E+08	1.72E+08	-0.40631	0.406308	128312	90560	90560	1	1	0.014006	1	0.026281	1
chr1	27481531	27481531	0.406005	0.406005	4878	11208	11208	1	6	0.014038	1	0.026336	1
chr11	10373718	10373718	-0.40585	0.40585	30170	243729	243729	1	1	0.014056	1	0.026364	1
chr16	2007559	2007559	-0.40547	0.405474	67262	325657	325657	1	2	0.014096	1	0.02643	1
chr5	83017553	83017644	0.163685	0.32737	142835	120820	120821	2	12	0.014148	1	0.045335	1
chr4	1.44E+08	1.44E+08	-0.40495	0.404954	136599	107952	107952	1	2	0.014148	1	0.026517	1
chr6	1.11E+08	1.11E+08	0.404933	0.404933	156071	156537	156537	1	4	0.01415	1	0.02652	1
chr7	2962240	2962240	0.404883	0.404883	161037	167664	167664	1	3	0.014156	1	0.026529	1
chr10	1.24E+08	1.24E+08	-0.40447	0.404469	26802	234458	234458	1	1	0.014199	1	0.026598	1
chr2	11757139	11757139	0.404449	0.404449	96857	43860	43860	1	1	0.014201	1	0.026601	1
chr22	43811071	43811071	0.404302	0.404302	118281	412780	412780	1	1	0.014217	1	0.026628	1
chr3	52030037	52030057	0.163479	0.326957	122514	79294	79295	2	8	0.014217	1	0.045471	1
chr10	14051821	14051838	-0.16343	0.326868	20109	220603	220604	2	2	0.014232	1	0.045501	1
chr10	1.31E+08	1.31E+08	-0.40398	0.403979	27542	236054	236054	1	2	0.01425	1	0.026681	1
chr21	46077562	46077582	0.163354	0.326709	115507	406391	406392	2	7	0.01426	1	0.045556	1
chr16	81254051	81254051	-0.40367	0.403671	72787	337836	337836	1	4	0.014281	1	0.026734	1
chr5	1.68E+08	1.68E+08	-0.40358	0.403579	147220	130021	130021	1	1	0.014292	1	0.026752	1
chr17	61920407	61920421	0.163207	0.326414	81672	359181	359182	2	11	0.014314	1	0.045657	1
chr11	61594965	61594997	-0.1631	0.326194	33157	250055	250056	2	12	0.014353	1	0.045732	1
chr3	1.19E+08	1.19E+08	-0.16308	0.326156	125233	84195	84196	2	8	0.01436	1	0.045745	1
chr12	1.26E+08	1.26E+08	0.402928	0.402928	47862	283146	283146	1	2	0.014363	1	0.026867	1
chr16	54225800	54225800	0.402926	0.402926	70896	333598	333598	1	1	0.014364	1	0.026868	1
chr3	10436869	10436869	0.402845	0.402845	119602	72917	72917	1	9	0.014373	1	0.026882	1
chr1	2785382	2785382	0.40265	0.40265	938	2641	2641	1	1	0.014395	1	0.026918	1
chr11	1078956	1079219	-0.16292	0.32585	28834	240082	240083	2	2	0.014411	1	0.045848	1
chr21	43528205	43528205	-0.40242	0.402423	115111	405565	405565	1	1	0.014417	1	0.026956	1
chr6	29796985	29796985	0.402414	0.402414	151431	140183	140183	1	21	0.014418	1	0.026957	1
chr4	1.9E+08	1.9E+08	0.162837	0.325674	138585	111956	111957	2	2	0.01444	1	0.045907	1
chr10	1.04E+08	1.04E+08	0.401732	0.401732	25568	231853	231853	1	9	0.014492	1	0.027076	1
chr5	404713	404713	-0.40155	0.401554	138757	112323	112323	1	8	0.014511	1	0.027107	1
chr11	1.22E+08	1.22E+08	-0.40128	0.401276	38123	261699	261699	1	1	0.014541	1	0.027156	1
chr7	11568529	11568529	0.401275	0.401275	161824	169517	169517	1	1	0.014541	1	0.027156	1
chr12	670974	670974	0.401203	0.401203	39466	264399	264399	1	6	0.014549	1	0.027169	1
chr12	57559808	57559808	-0.40116	0.401163	43568	273762	273762	1	3	0.014553	1	0.027176	1
chr10	1.15E+08	1.15E+08	-0.40105	0.401049	26242	233246	233246	1	1	0.014566	1	0.027196	1
chr17	59543726	59543726	0.401004	0.401004	81478	358748	358748	1	1	0.01457	1	0.027204	1
chr4	1772062	1772151	-0.16233	0.32467	130661	95807	95808	2	2	0.014622	1	0.046248	1
chr6	34724739	34724741	-0.16232	0.324648	152576	149274	149275	2	11	0.014625	1	0.046255	1
chr16	11410036	11410036	0.400506	0.400506	68472	328480	328480	1	1	0.014627	1	0.027295	1
chr1	1.54E+08	1.54E+08	-0.16228	0.324566	11910	26882	26883	2	10	0.01464	1	0.046283	1
chr19	35329641	35329860	-0.16225	0.324492	91532	383916	383917	2	4	0.014652	1	0.046306	1
chr20	32700235	32700246	-0.16218	0.324355	112022	398001	398002	2	9	0.014675	1	0.046354	1
chr6	1.7E+08	1.7E+08	-0.39982	0.399819	159692	163839	163839	1	2	0.0147	1	0.027418	1
chr9	5040816	5040816	-0.39975	0.399752	180709	209196	209196	1	4	0.014709	1	0.027431	1
chr8	1.42E+08	1.42E+08	-0.39961	0.399612	179466	205947	205947	1	4	0.014725	1	0.027456	1
chr16	70143703	70143703	0.399419	0.399419	72086	336402	336402	1	1	0.014745	1	0.027489	1
chr17	212481	212481	-0.39927	0.399266	74823	342503	342503	1	1	0.014761	1	0.027517	1
chr8	6534897	6534897	-0.39897	0.398973	172371	192298	192298	1	1	0.014793	1	0.027568	1

chr6	1.69E+08	1.69E+08	-0.39895	0.398953	159617	163674	163674	1	3	0.014795	1	0.027571	1
chr10	1.34E+08	1.34E+08	-0.39886	0.398856	27926	237232	237232	1	5	0.014805	1	0.027588	1
chr2	1.07E+08	1.07E+08	-0.39866	0.398664	102458	55329	55329	1	1	0.014826	1	0.027621	1
chr3	1.91E+08	1.91E+08	0.398437	0.398437	129442	92790	92790	1	1	0.014851	1	0.027662	1
chr2	1.73E+08	1.73E+08	-0.39833	0.398331	105715	61461	61461	1	1	0.014864	1	0.027682	1
chr2	1481492	1481492	-0.39793	0.397934	95855	41786	41786	1	4	0.014909	1	0.027755	1
chr10	79492099	79492099	0.397618	0.397618	23664	227617	227617	1	2	0.014942	1	0.027812	1
chr1	78602941	78602941	0.397471	0.397471	8923	20216	20216	1	1	0.014959	1	0.027839	1
chr12	9600060	9600060	-0.39732	0.397321	40512	266765	266765	1	12	0.014976	1	0.027867	1
chr15	26915732	26915752	-0.16118	0.322366	60387	310697	310698	2	4	0.015028	1	0.047047	1
chr3	38033516	38033516	-0.39663	0.396626	121077	75728	75728	1	4	0.015055	1	0.027995	1
chr3	1.6E+08	1.6E+08	-0.39662	0.396619	127842	89654	89654	1	1	0.015056	1	0.027997	1
chr2	2.43E+08	2.43E+08	0.161002	0.322003	110594	71748	71749	2	7	0.015097	1	0.047173	1
chr6	1.68E+08	1.68E+08	0.160843	0.321685	159372	163056	163057	2	2	0.015154	1	0.047286	1
chr19	41319790	41319790	-0.3957	0.395697	92492	386472	386472	1	1	0.015159	1	0.028169	1
chr22	27152698	27152963	0.16081	0.321621	116886	409387	409388	2	4	0.015165	1	0.047308	1
chr10	1.19E+08	1.19E+08	0.395577	0.395577	26458	233766	233766	1	1	0.015173	1	0.028192	1
chr22	43168851	43168851	0.395572	0.395572	118215	412617	412617	1	1	0.015173	1	0.028193	1
chr1	2.36E+08	2.36E+08	0.1607	0.321399	17909	39487	39488	2	10	0.015207	1	0.04739	1
chr6	73973719	73973719	0.395263	0.395263	154733	153597	153597	1	1	0.015209	1	0.028252	1
chr17	78652902	78652902	0.394985	0.394985	83701	364046	364046	1	1	0.015242	1	0.028305	1
chr11	1.33E+08	1.33E+08	-0.39454	0.394543	39173	263740	263740	1	6	0.01529	1	0.028386	1
chr17	30244184	30244370	0.160389	0.320778	78153	350565	350566	2	6	0.015321	1	0.047611	1
chr5	63933908	63933908	-0.39401	0.394013	141772	118704	118704	1	1	0.015352	1	0.028485	1
chr5	1.4E+08	1.4E+08	0.393855	0.393855	145434	126171	126171	1	12	0.01537	1	0.028514	1
chr7	1.53E+08	1.53E+08	0.393776	0.393776	170601	187979	187979	1	2	0.01538	1	0.02853	1
chr6	1.58E+08	1.58E+08	-0.39372	0.393718	158550	161272	161272	1	2	0.015387	1	0.028542	1
chr2	382237	382356	-0.16021	0.32042	95669	41356	41357	2	3	0.01539	1	0.04774	1
chr2	33951647	33951647	0.393599	0.393599	98281	46777	46777	1	1	0.015402	1	0.028564	1
chr10	5454459	5454459	0.393594	0.393594	19502	219294	219294	1	9	0.015402	1	0.028565	1
chr17	57187728	57187728	0.393559	0.393559	81281	358267	358267	1	1	0.015407	1	0.028572	1
chr6	32309323	32309323	-0.39356	0.393558	152090	146267	146267	1	3	0.015407	1	0.028573	1
chr10	21799314	21799395	0.159992	0.319984	20469	221308	221309	2	9	0.015471	1	0.047893	1
chr10	14439215	14439215	0.392775	0.392775	20131	220634	220634	1	1	0.015494	1	0.028718	1
chr1	1.45E+08	1.45E+08	0.392702	0.392702	11144	25024	25024	1	2	0.015503	1	0.028732	1
chr19	53194695	53194699	0.159781	0.319562	94570	391691	391692	2	2	0.015548	1	0.048045	1
chr11	2222912	2222912	-0.39211	0.392108	29208	241265	241265	1	2	0.015575	1	0.028848	1
chr14	1.04E+08	1.04E+08	-0.15969	0.319379	59249	308118	308119	2	2	0.015581	1	0.048111	1
chr6	31164921	31164924	-0.15964	0.319288	151767	142820	142821	2	31	0.015597	1	0.048144	1
chr6	32451079	32451079	-0.39154	0.391542	152129	146377	146377	1	1	0.015642	1	0.028958	1
chr5	344218	344218	0.391336	0.391336	138735	112276	112276	1	4	0.015665	1	0.028996	1
chr7	34911385	34911385	0.391212	0.391212	163172	172519	172519	1	3	0.015681	1	0.029021	1
chr3	61237223	61237223	0.391113	0.391113	123164	80586	80586	1	14	0.015692	1	0.029039	1
chr1	3077798	3077835	0.159349	0.318699	1060	2987	2988	2	6	0.015713	1	0.048362	1
chr2	2.22E+08	2.22E+08	-0.15935	0.318693	108411	67065	67066	2	18	0.015714	1	0.048364	1
chr13	1.13E+08	1.13E+08	0.159078	0.318156	53151	294474	294475	2	3	0.015821	1	0.04856	1
chr1	2.3E+08	2.3E+08	-0.38995	0.389946	17440	38603	38603	1	1	0.015832	1	0.029267	1
chr14	81405846	81405846	0.389703	0.389703	57431	304179	304179	1	1	0.015861	1	0.029315	1
chr11	68637568	68637592	-0.15891	0.317827	34620	254242	254243	2	2	0.015885	1	0.048682	1
chr7	1.17E+08	1.17E+08	-0.38944	0.389436	168088	182831	182831	1	1	0.015894	1	0.02937	1
chr2	2.35E+08	2.35E+08	-0.15883	0.317666	109243	68792	68793	2	5	0.015916	1	0.048742	1
chr12	2881762	2881762	-0.38916	0.389156	39728	264883	264883	1	1	0.015927	1	0.029424	1
chr5	1180364	1180364	-0.38827	0.388266	139031	113084	113084	1	1	0.016033	1	0.029599	1
chr3	1.93E+08	1.93E+08	-0.38819	0.388194	129491	92876	92876	1	1	0.016042	1	0.029614	1
chr19	597635	597635	0.388147	0.388147	86980	372361	372361	1	1	0.016047	1	0.029622	1
chr14	24505537	24505547	-0.15843	0.316862	54391	297707	297708	2	8	0.016072	1	0.049042	1
chr7	2719090	2719090	0.387844	0.387844	160970	167487	167487	1	5	0.016084	1	0.02968	1
chr9	97810617	97810769	0.158368	0.316736	182602	212107	212108	2	4	0.016097	1	0.049088	1
chr1	28763330	28763330	0.38742	0.38742	5079	11717	11717	1	1	0.016135	1	0.029765	1
chr17	7613764	7613764	0.387025	0.387025	76111	345967	345967	1	1	0.016181	1	0.029843	1
chr16	11374865	11374865	0.386554	0.386554	68469	328472	328472	1	7	0.016237	1	0.029936	1
chr20	61153997	61153997	-0.38586	0.385858	113686	402312	402312	1	2	0.016325	1	0.030079	1
chr14	63568796	63568796	0.385722	0.385722	56058	301407	301407	1	6	0.016343	1	0.030108	1
chr1	3047462	3047462	0.385607	0.385607	1046	2938	2938	1	3	0.016357	1	0.03013	1
chr2	2.19E+08	2.19E+08	-0.38554	0.385541	108018	66083	66083	1	6	0.016365	1	0.030143	1
chr2	80281335	80281335	0.385361	0.385361	101024	52329	52329	1	1	0.016387	1	0.03018	1
chr19	55362650	55362650	0.385204	0.385204	94944	392641	392641	1	1	0.016405	1	0.030211	1
chr1	2.47E+08	2.47E+08	-0.38508	0.38508	18556	40738	40738	1	1	0.016421	1	0.030235	1
chr10	49654342	49654342	0.385047	0.385047	21982	224262	224262	1	1	0.016424	1	0.030241	1
chr16	57484979	57484979	-0.38487	0.384872	71180	334312	334312	1	1	0.016446	1	0.030275	1
chr11	1.13E+08	1.13E+08	-0.15742	0.314849	37237	259789	259790	2	2	0.016472	1	0.049805	1
chr16	2746544	2746544	0.384103	0.384103	67532	326367	326367	1	2	0.01654	1	0.030433	1
chr3	1.29E+08	1.29E+08	0.157145	0.314289	126245	86325	86326	2	2	0.016584	1	0.050029	1
chr15	72567956	72567956	0.383757	0.383757	63858	317781	317781	1	1	0.016585	1	0.030504	1

chr5	1.8E+08	1.8E+08	0.383145	0.383145	148696	133233	133233	1	1	0.016662	1	0.030627	1
chr17	79011140	79011140	0.382868	0.382868	83836	364457	364457	1	7	0.016698	1	0.030684	1
chr1	2.15E+08	2.15E+08	0.156818	0.313637	16383	36374	36375	2	9	0.016716	1	0.050281	1
chr5	32223673	32223673	0.382528	0.382528	140477	116115	116115	1	1	0.016741	1	0.030755	1
chr3	1.31E+08	1.31E+08	-0.38234	0.382345	126365	86560	86560	1	1	0.016765	1	0.030793	1
chr10	1.35E+08	1.35E+08	0.382175	0.382175	28300	238468	238468	1	4	0.016786	1	0.030827	1
chr18	74961078	74961138	-0.15662	0.313246	86416	371264	371265	2	28	0.016795	1	0.050429	1
chr17	41738893	41738893	-0.38144	0.38144	79629	354249	354249	1	6	0.016882	1	0.030981	1
chr8	884647	884716	-0.1564	0.312798	171798	191016	191017	2	7	0.016887	1	0.050605	1
chr16	83171155	83171299	0.156212	0.312425	72919	338081	338082	2	2	0.016966	1	0.05075	1
chr5	1.75E+08	1.75E+08	0.156131	0.312262	147796	131220	131221	2	3	0.016999	1	0.050811	1
chr7	30725669	30725669	0.380483	0.380483	162907	172083	172083	1	1	0.017005	1	0.031181	1
chr19	46801642	46801672	0.156114	0.312228	93340	388592	388593	2	3	0.017006	1	0.050824	1
chr10	60456715	60456715	-0.38018	0.380183	22366	225033	225033	1	1	0.017044	1	0.031246	1
chr2	2.4E+08	2.4E+08	-0.38015	0.380151	109911	70124	70124	1	1	0.017049	1	0.031253	1
chr19	2901147	2901147	0.380002	0.380002	87869	374535	374535	1	13	0.017067	1	0.031282	1
chr12	57622963	57623095	-0.15589	0.31179	43604	273856	273857	2	16	0.017103	1	0.050999	1
chr11	1.25E+08	1.25E+08	0.379245	0.379245	38399	262213	262213	1	1	0.017164	1	0.031442	1
chr4	77172836	77172841	0.155738	0.311476	134147	103114	103115	2	10	0.017168	1	0.051128	1
chr15	76016056	76016056	0.379022	0.379022	64279	318811	318811	1	5	0.017192	1	0.031488	1
chr14	1.05E+08	1.05E+08	-0.1556	0.311204	59414	308508	308509	2	3	0.017226	1	0.051234	1
chr20	61047376	61047376	0.378636	0.378636	113676	402251	402251	1	1	0.017243	1	0.03157	1
chr20	4804131	4804131	-0.3785	0.378499	111131	395707	395707	1	8	0.017261	1	0.0316	1
chr19	54210518	54210518	0.378305	0.378305	94715	392060	392060	1	4	0.017288	1	0.031642	1
chr16	57094643	57094643	-0.3782	0.378196	71127	334189	334189	1	1	0.017302	1	0.031665	1
chr16	30572739	30572815	0.155392	0.310785	69951	331566	331567	2	5	0.017312	1	0.051398	1
chr12	9065171	9065171	-0.37805	0.378047	40465	266668	266668	1	2	0.017323	1	0.031698	1
chr1	68962614	68962753	-0.15535	0.310708	8668	19635	19636	2	14	0.017329	1	0.051429	1
chr19	4788146	4788146	-0.3777	0.377697	88407	375788	375788	1	1	0.01737	1	0.031773	1
chr14	22370643	22370643	-0.37768	0.377679	54112	297081	297081	1	2	0.017372	1	0.031776	1
chr6	79788076	79788099	-0.15523	0.310468	154929	154011	154012	2	15	0.017381	1	0.051525	1
chr6	32330188	32330188	-0.37747	0.377473	152098	146276	146276	1	2	0.0174	1	0.031822	1
chr3	1.6E+08	1.6E+08	0.155163	0.310326	127848	89665	89666	2	8	0.017412	1	0.051581	1
chr1	12120304	12120304	-0.37711	0.377105	2771	6764	6764	1	1	0.017448	1	0.031902	1
chr3	57945651	57945695	-0.15507	0.310149	123004	80324	80325	2	2	0.01745	1	0.051654	1
chr10	49348564	49348564	0.376744	0.376744	21962	224226	224226	1	1	0.017495	1	0.031978	1
chr18	55919633	55919633	-0.37659	0.376587	86008	370312	370312	1	1	0.017515	1	0.032011	1
chr11	85873778	85873778	0.376587	0.376587	36090	257445	257445	1	1	0.017515	1	0.032011	1
chr16	3519514	3519514	0.376566	0.376566	67785	327046	327046	1	1	0.017517	1	0.032014	1
chr10	2543474	2543513	-0.15483	0.309669	19235	218759	218760	2	2	0.017553	1	0.051848	1
chr7	1.4E+08	1.4E+08	-0.37587	0.375873	169573	185727	185727	1	1	0.01761	1	0.032163	1
chr2	1.31E+08	1.31E+08	-0.37548	0.375483	103777	57883	57883	1	1	0.017663	1	0.032248	1
chr16	1939295	1939295	0.375295	0.375295	67242	325602	325602	1	2	0.017687	1	0.032288	1
chr5	1039552	1039552	0.37445	0.37445	138963	112870	112870	1	2	0.017803	1	0.032475	1
chr22	46262041	46262041	-0.37431	0.374313	118444	413192	413192	1	2	0.01782	1	0.032504	1
chr19	58545290	58545333	0.154164	0.308327	95490	394126	394127	2	11	0.017845	1	0.052394	1
chr5	1.41E+08	1.41E+08	0.154164	0.308327	145670	127062	127063	2	5	0.017845	1	0.052394	1
chr11	2323056	2323059	-0.15416	0.30832	29236	241395	241396	2	30	0.017847	1	0.052397	1
chr16	10281150	10281150	-0.37398	0.373983	68348	328227	328227	1	1	0.017863	1	0.032574	1
chr1	1.18E+08	1.18E+08	-0.37388	0.373882	10876	24508	24508	1	1	0.017876	1	0.032596	1
chr7	76624761	76624761	0.373799	0.373799	165755	177598	177598	1	1	0.017888	1	0.032613	1
chr20	61443715	61443751	0.154061	0.308122	113739	402431	402432	2	2	0.017892	1	0.052478	1
chr2	8596908	8597159	-0.15406	0.308111	96443	43026	43027	2	4	0.017894	1	0.052483	1
chr22	43623455	43623494	0.153963	0.307926	118268	412753	412754	2	2	0.017936	1	0.052559	1
chr20	259898	259925	0.153907	0.307815	110667	394578	394579	2	2	0.017962	1	0.052607	1
chr6	37662649	37662649	-0.37327	0.373273	152969	150071	150071	1	1	0.017966	1	0.032734	1
chr7	64894861	64894861	0.37306	0.37306	164920	175926	175926	1	2	0.017994	1	0.032781	1
chr8	38782090	38782090	0.372646	0.372646	174716	196926	196926	1	1	0.018053	1	0.032875	1
chr19	12984170	12984645	-0.15369	0.307376	89806	379483	379484	2	5	0.018056	1	0.052785	1
chr17	57053	57053	-0.37252	0.37252	74760	342363	342363	1	3	0.018071	1	0.032905	1
chr12	1.34E+08	1.34E+08	-0.37246	0.372462	48875	285745	285745	1	2	0.018079	1	0.032919	1
chr10	1.27E+08	1.27E+08	-0.15362	0.307238	27194	235306	235307	2	3	0.018088	1	0.052845	1
chr17	75378036	75378036	-0.37233	0.372333	83008	362302	362302	1	1	0.018096	1	0.032948	1
chr1	17287501	17287670	0.153567	0.307135	3430	8035	8036	2	4	0.018112	1	0.052889	1
chr13	1.15E+08	1.15E+08	0.15345	0.306901	53719	296183	296184	2	5	0.018162	1	0.052985	1
chr1	1.45E+08	1.45E+08	-0.15344	0.306876	11100	24944	24945	2	9	0.018167	1	0.052995	1
chr16	14403004	14403022	-0.15342	0.306836	68667	328832	328833	2	7	0.018176	1	0.05301	1
chr1	8152878	8152878	0.371552	0.371552	2032	5278	5278	1	1	0.018202	1	0.033122	1
chr10	48185197	48185200	0.153352	0.306705	21900	224124	224125	2	2	0.018208	1	0.053069	1
chr1	2981840	2981840	0.370801	0.370801	1015	2821	2821	1	1	0.018305	1	0.033292	1
chr1	33502512	33502579	0.153107	0.306215	5603	12903	12904	2	11	0.018318	1	0.053274	1
chr16	33483591	33483591	-0.37066	0.370664	70305	332462	332462	1	5	0.018325	1	0.033324	1
chr4	79282758	79282758	0.370638	0.370638	134308	103380	103380	1	1	0.018328	1	0.033329	1
chr17	11503508	11503508	-0.3704	0.370401	76553	347067	347067	1	1	0.018364	1	0.033388	1

chr2	62535081	62535081	-0.37	0.369998	99796	49653	49653	1	1	0.01842	1	0.033478	1
chr13	91827542	91827600	0.152872	0.305744	51940	291961	291962	2	8	0.018424	1	0.05347	1
chr16	81898406	81898406	-0.36993	0.369932	72867	337984	337984	1	1	0.018429	1	0.033494	1
chr3	1.14E+08	1.14E+08	-0.36941	0.369411	125031	83884	83884	1	2	0.018499	1	0.033608	1
chr21	46825222	46825222	-0.36935	0.36935	115655	406698	406698	1	6	0.018507	1	0.033622	1
chr5	766938	766938	0.369308	0.369308	138885	112680	112680	1	4	0.018514	1	0.033633	1
chr1	2.26E+08	2.26E+08	-0.15266	0.305321	17005	37569	37570	2	6	0.018518	1	0.05365	1
chr5	32445482	32445482	0.368969	0.368969	140495	116146	116146	1	2	0.018564	1	0.033712	1
chr3	1.19E+08	1.19E+08	-0.36894	0.368944	125251	84246	84246	1	1	0.018567	1	0.033717	1
chr5	1.41E+08	1.41E+08	-0.15255	0.305101	145599	126746	126747	2	9	0.018568	1	0.053744	1
chr7	50132653	50132903	0.152445	0.30489	164228	174544	174545	2	11	0.018619	1	0.053834	1
chr19	18170383	18170383	-0.36856	0.368555	90640	381660	381660	1	1	0.018621	1	0.033805	1
chr2	2.41E+08	2.41E+08	-0.15221	0.304419	110134	70654	70655	2	24	0.018731	1	0.054036	1
chr16	10772249	10772249	-0.3676	0.367604	68377	328277	328277	1	1	0.018763	1	0.034033	1
chr21	30391383	30391563	-0.15214	0.304278	114428	403969	403970	2	8	0.018766	1	0.054099	1
chr7	1121190	1121190	-0.36754	0.367543	160349	165603	165603	1	12	0.018772	1	0.034048	1
chr6	33090304	33090304	0.367406	0.367406	152261	147258	147258	1	3	0.018791	1	0.034078	1
chr4	1.89E+08	1.89E+08	-0.36702	0.36702	138511	111837	111837	1	3	0.018849	1	0.034171	1
chr2	75874389	75874389	-0.36699	0.366994	100931	52189	52189	1	12	0.018852	1	0.034177	1
chr6	75775380	75775380	-0.36675	0.366745	154817	153777	153777	1	1	0.018888	1	0.034234	1
chr6	1.23E+08	1.23E+08	-0.15187	0.303735	156591	157553	157554	2	14	0.018891	1	0.054333	1
chr13	1.13E+08	1.13E+08	0.151846	0.303691	53209	294665	294666	2	3	0.018902	1	0.054353	1
chr1	1.18E+08	1.18E+08	0.151815	0.303629	10812	24390	24391	2	3	0.018916	1	0.054378	1
chr2	2.06E+08	2.06E+08	0.366548	0.366548	107298	64792	64792	1	1	0.018917	1	0.034279	1
chr2	1.99E+08	1.99E+08	-0.15167	0.303348	106846	63882	63883	2	16	0.018979	1	0.054501	1
chr13	1.12E+08	1.12E+08	0.15167	0.303341	53107	294344	294345	2	3	0.018981	1	0.054505	1
chr16	85531414	85531414	0.366122	0.366122	73325	338878	338878	1	1	0.018981	1	0.034382	1
chr14	1.05E+08	1.05E+08	0.366006	0.366006	59361	308375	308375	1	5	0.018998	1	0.034409	1
chr10	3164740	3164740	0.365999	0.365999	19292	218897	218897	1	4	0.018999	1	0.034411	1
chr12	1.25E+08	1.25E+08	-0.36599	0.365992	47649	282717	282717	1	6	0.019	1	0.034412	1
chr4	7735312	7735312	0.365742	0.365742	131629	98089	98089	1	1	0.019035	1	0.034474	1
chr2	2726437	2726437	0.365642	0.365642	96029	42168	42168	1	1	0.01905	1	0.034499	1
chr17	34509482	34509488	-0.1515	0.303008	78485	351322	351323	2	2	0.019056	1	0.054649	1
chr3	23782847	23782847	0.365593	0.365593	120460	74521	74521	1	1	0.019058	1	0.034511	1
chr11	60611862	60611862	0.365201	0.365201	32987	249603	249603	1	1	0.019113	1	0.034602	1
chr7	39170539	39170763	-0.15135	0.302691	163460	173047	173048	2	5	0.019131	1	0.054789	1
chr3	75657183	75657183	-0.36504	0.365043	123984	81926	81926	1	1	0.019136	1	0.03464	1
chr15	79107452	79107452	-0.36461	0.364611	64594	319452	319452	1	1	0.0192	1	0.034743	1
chr11	96076288	96076288	-0.36442	0.364417	36603	258390	258390	1	11	0.019229	1	0.03479	1
chr9	1.33E+08	1.33E+08	0.364348	0.364348	184472	215064	215064	1	1	0.019238	1	0.034806	1
chr22	24382663	24382663	-0.36427	0.364271	116687	408911	408911	1	1	0.019249	1	0.034824	1
chr1	1.53E+08	1.53E+08	-0.15109	0.30218	11877	26818	26819	2	3	0.019252	1	0.055016	1
chr13	96204870	96204873	0.151086	0.302172	52078	292237	292238	2	16	0.019254	1	0.055019	1
chr7	2121155	2121155	-0.36416	0.36416	160764	166905	166905	1	1	0.019266	1	0.034852	1
chr3	15902307	15902359	0.15105	0.302099	120180	74072	74073	2	8	0.019271	1	0.055051	1
chr3	1.18E+08	1.18E+08	-0.36407	0.364068	125169	84074	84074	1	1	0.019279	1	0.034874	1
chr1	1.61E+08	1.61E+08	-0.15099	0.301989	13020	29702	29703	2	9	0.019296	1	0.0551	1
chr4	3683268	3683268	0.363831	0.363831	131099	96862	96862	1	1	0.019315	1	0.03493	1
chr17	40700314	40700509	0.150909	0.301817	79442	353679	353680	2	5	0.01934	1	0.055178	1
chr4	82521413	82521413	0.36356	0.36356	134447	103684	103684	1	2	0.019357	1	0.034996	1
chr8	98271949	98271949	0.363113	0.363113	177252	202040	202040	1	1	0.019422	1	0.0351	1
chr6	33091567	33091591	0.150586	0.301172	152262	147265	147266	2	13	0.019497	1	0.055469	1
chr4	1.22E+08	1.22E+08	-0.3626	0.362604	135834	106511	106511	1	1	0.019497	1	0.035222	1
chr8	1.33E+08	1.33E+08	0.362237	0.362237	178953	205078	205078	1	1	0.019554	1	0.035315	1
chr6	30980847	30980847	-0.36215	0.36215	151712	142579	142579	1	1	0.019566	1	0.035336	1
chr1	75591029	75591353	-0.15036	0.300718	8781	19892	19893	2	5	0.019608	1	0.05567	1
chr5	506439	506439	-0.3618	0.361804	138811	112471	112471	1	5	0.01962	1	0.035421	1
chr11	49229833	49229833	-0.36165	0.361651	32453	248563	248563	1	4	0.019644	1	0.035459	1
chr6	43197544	43197574	0.150274	0.300548	153531	151285	151286	2	9	0.019649	1	0.055744	1
chr5	79647805	79647805	0.361617	0.361617	142663	120515	120515	1	4	0.019649	1	0.035467	1
chr7	1.55E+08	1.55E+08	-0.15022	0.300448	170704	188191	188192	2	14	0.019674	1	0.05579	1
chr2	2.35E+08	2.35E+08	0.150222	0.300445	109282	68844	68845	2	2	0.019675	1	0.055792	1
chr17	19620263	19620263	-0.36123	0.361233	77350	348698	348698	1	4	0.019706	1	0.035559	1
chr10	1.35E+08	1.35E+08	0.150102	0.300204	28269	238368	238369	2	10	0.019733	1	0.055904	1
chr1	1.54E+08	1.54E+08	-0.15006	0.300124	11947	26992	26993	2	11	0.019754	1	0.05594	1
chr2	1.28E+08	1.28E+08	0.360921	0.360921	103568	57502	57502	1	3	0.019754	1	0.035636	1
chr2	2.41E+08	2.41E+08	-0.36064	0.360643	110176	70750	70750	1	1	0.019795	1	0.035703	1
chr1	59008471	59008471	-0.36048	0.360483	8052	18425	18425	1	1	0.01982	1	0.035742	1
chr1	1.09E+08	1.09E+08	-0.36037	0.360369	10033	22540	22540	1	4	0.019838	1	0.03577	1
chr19	55582635	55582635	0.360346	0.360346	94980	392711	392711	1	1	0.019842	1	0.035775	1
chr3	1.74E+08	1.74E+08	-0.36032	0.360315	128393	90695	90695	1	1	0.019846	1	0.035783	1
chr12	1.06E+08	1.06E+08	-0.36017	0.36017	45732	278102	278102	1	1	0.019867	1	0.035819	1
chr20	43371550	43371550	-0.36016	0.36016	112592	399584	399584	1	1	0.019869	1	0.035822	1
chr11	1.21E+08	1.21E+08	-0.14982	0.299641	38048	261560	261561	2	4	0.019871	1	0.05616	1

chr15	68177161	68177161	-0.35985	0.359846	63424	317027	317027	1	4	0.019914	1	0.035896	1
chr5	1.63E+08	1.63E+08	0.149661	0.299322	147030	129700	129701	2	13	0.019951	1	0.056309	1
chr19	49375674	49375674	0.359407	0.359407	93828	389749	389749	1	7	0.019982	1	0.036005	1
chr11	8012913	8012913	-0.35937	0.359371	29925	243227	243227	1	1	0.019987	1	0.036014	1
chr10	1.23E+08	1.23E+08	-0.35932	0.359319	26757	234369	234369	1	1	0.019995	1	0.036027	1
chr16	5116160	5116160	-0.35928	0.359283	68117	327810	327810	1	5	0.020001	1	0.036035	1
chr2	1.11E+08	1.11E+08	0.149492	0.298985	102660	55701	55702	2	7	0.020035	1	0.056461	1
chr14	38724648	38724675	0.149357	0.298713	54944	299122	299123	2	11	0.020101	1	0.056584	1
chr10	1.26E+08	1.26E+08	0.358653	0.358653	27048	235020	235020	1	1	0.020101	1	0.036196	1
chr2	60712421	60712421	-0.35796	0.357963	99678	49349	49349	1	1	0.020206	1	0.036367	1
chr17	1040653	1040653	0.357905	0.357905	75021	342965	342965	1	4	0.020216	1	0.036383	1
chr10	82189478	82189478	-0.35789	0.357894	23981	228132	228132	1	1	0.020218	1	0.036386	1
chr6	1.68E+08	1.68E+08	-0.35769	0.357695	159317	162937	162937	1	2	0.02025	1	0.036439	1
chr19	843940	843995	0.149055	0.29811	87086	372612	372613	2	4	0.020255	1	0.056873	1
chr5	1.8E+08	1.8E+08	-0.3575	0.357503	148681	133208	133208	1	1	0.02028	1	0.036486	1
chr19	49202858	49202858	-0.35746	0.357463	93781	389639	389639	1	1	0.020286	1	0.036496	1
chr7	27153655	27153663	-0.14896	0.297919	162541	171068	171069	2	26	0.020306	1	0.056963	1
chr3	44803129	44803129	-0.35718	0.357178	121590	76884	76884	1	18	0.020331	1	0.036567	1
chr5	1.8E+08	1.8E+08	-0.3566	0.356599	148682	133209	133209	1	1	0.020425	1	0.036722	1
chr19	52514445	52514445	-0.35658	0.356585	94488	391457	391457	1	1	0.020427	1	0.036726	1
chr15	76552574	76552574	-0.35653	0.35653	64333	318917	318917	1	4	0.020435	1	0.036739	1
chr1	1.62E+08	1.62E+08	-0.35641	0.356414	13059	29778	29778	1	1	0.020454	1	0.036768	1
chr7	98029266	98029285	-0.14864	0.297286	166648	179671	179672	2	2	0.020468	1	0.057266	1
chr1	1.49E+08	1.49E+08	-0.14864	0.297272	11395	25609	25610	2	2	0.020473	1	0.057273	1
chr7	1.43E+08	1.43E+08	0.356287	0.356287	169814	186233	186233	1	4	0.020475	1	0.036802	1
chr1	1.53E+08	1.53E+08	-0.35598	0.355982	11795	26648	26648	1	3	0.020527	1	0.036884	1
chr14	70317228	70317239	0.148535	0.29707	56553	302425	302426	2	7	0.020527	1	0.05737	1
chr17	8777762	8777762	-0.35588	0.355882	76349	346636	346636	1	1	0.020543	1	0.036909	1
chr10	1.16E+08	1.16E+08	0.355534	0.355534	26245	233249	233249	1	1	0.020599	1	0.037002	1
chr1	1.97E+08	1.97E+08	0.148369	0.296738	14737	33024	33025	2	8	0.020611	1	0.057526	1
chr3	1.12E+08	1.12E+08	-0.35525	0.355252	124849	83528	83528	1	2	0.020647	1	0.037078	1
chr4	4136918	4136918	0.354926	0.354926	131172	97024	97024	1	6	0.0207	1	0.037163	1
chr11	1.25E+08	1.25E+08	-0.35485	0.354845	38492	262431	262431	1	1	0.020713	1	0.037185	1
chr6	32186244	32186244	-0.35484	0.35484	152052	146154	146154	1	41	0.020713	1	0.037186	1
chr5	565486	565934	0.148164	0.296328	138834	112531	112532	2	4	0.020715	1	0.057722	1
chr9	1.02E+08	1.02E+08	-0.35456	0.354559	182886	212574	212574	1	1	0.020761	1	0.037261	1
chr1	2373387	2373387	0.354514	0.354514	798	2287	2287	1	2	0.020768	1	0.037273	1
chr5	1.8E+08	1.8E+08	-0.35433	0.354327	148683	133210	133210	1	1	0.020799	1	0.037322	1
chr2	55450737	55450823	0.147976	0.295952	99529	49049	49050	2	4	0.020813	1	0.057901	1
chr2	1.97E+08	1.97E+08	-0.14792	0.295837	106734	63649	63650	2	10	0.020842	1	0.057955	1
chr7	6836436	6836436	0.353793	0.353793	161663	169209	169209	1	1	0.020887	1	0.037467	1
chr14	88793634	88793741	0.147828	0.295655	57597	304470	304471	2	7	0.02089	1	0.058045	1
chr15	61972967	61972967	0.353705	0.353705	62720	315609	315609	1	1	0.0209	1	0.037488	1
chr5	79366549	79366549	0.353637	0.353637	142631	120463	120463	1	1	0.020911	1	0.037508	1
chr17	66597372	66597378	0.147782	0.295564	82076	360065	360066	2	5	0.020914	1	0.058093	1
chr17	81047721	81047721	0.353443	0.353443	84622	366879	366879	1	5	0.020944	1	0.03756	1
chr1	2.27E+08	2.27E+08	-0.353	0.353004	17132	37843	37843	1	1	0.021016	1	0.037678	1
chr2	28605224	28605224	-0.35294	0.352939	97952	46190	46190	1	1	0.021026	1	0.037694	1
chr13	25322170	25322170	0.352823	0.352823	49423	286895	286895	1	4	0.021042	1	0.037723	1
chr11	15180922	15180922	-0.35279	0.352792	30555	244466	244466	1	1	0.021048	1	0.037731	1
chr3	1.77E+08	1.77E+08	-0.35278	0.352781	128494	90821	90821	1	1	0.021049	1	0.037734	1
chr10	1.27E+08	1.27E+08	0.352758	0.352758	27136	235216	235216	1	2	0.021053	1	0.03774	1
chr15	90927939	90927939	0.352632	0.352632	65541	321390	321390	1	1	0.021074	1	0.037774	1
chr5	2387924	2387924	0.352366	0.352366	139372	114071	114071	1	3	0.021118	1	0.037847	1
chr6	1.49E+08	1.49E+08	0.352196	0.352196	157848	159923	159923	1	1	0.021147	1	0.037891	1
chr10	1.21E+08	1.21E+08	-0.35198	0.351983	26617	234125	234125	1	3	0.021183	1	0.03795	1
chr6	1.42E+08	1.42E+08	0.351901	0.351901	157537	159332	159332	1	1	0.021196	1	0.037971	1
chr6	33131893	33131893	0.351894	0.351894	152271	147326	147326	1	70	0.021196	1	0.037973	1
chr14	1.05E+08	1.05E+08	-0.35188	0.351879	59566	308853	308853	1	3	0.021199	1	0.037977	1
chr4	81122687	81122726	-0.14709	0.29417	134383	103543	103544	2	3	0.021271	1	0.058767	1
chr11	464665	464665	0.351115	0.351115	28569	239273	239273	1	4	0.021324	1	0.038181	1
chr2	2.42E+08	2.42E+08	-0.3508	0.350803	110249	70922	70922	1	3	0.021374	1	0.038264	1
chr4	1.58E+08	1.58E+08	-0.35073	0.350726	137238	109217	109217	1	1	0.021387	1	0.038285	1
chr9	90531866	90531866	-0.3507	0.3507	182186	211469	211469	1	1	0.021391	1	0.038292	1
chr11	1001560	1001560	0.350528	0.350528	28798	239955	239955	1	11	0.021421	1	0.03834	1
chr5	392364	392364	0.350509	0.350509	138751	112307	112307	1	1	0.021424	1	0.038345	1
chr2	10184444	10184457	-0.14671	0.293415	96651	43406	43407	2	3	0.021472	1	0.059147	1
chr9	1.21E+08	1.21E+08	0.350211	0.350211	183470	213474	213474	1	1	0.021476	1	0.038429	1
chr7	22122872	22122872	0.350209	0.350209	162188	170259	170259	1	5	0.021477	1	0.038429	1
chr16	34809318	34809677	-0.14665	0.293307	70387	332616	332617	2	5	0.0215	1	0.059199	1
chr19	36198177	36198177	-0.34996	0.349965	91674	384325	384325	1	1	0.021518	1	0.038494	1
chr7	1018969	1018969	-0.34993	0.349927	160291	165402	165402	1	6	0.021524	1	0.038505	1
chr2	71033156	71033156	-0.34974	0.34974	100431	50945	50945	1	1	0.021555	1	0.038556	1
chr6	2953027	2953123	0.146533	0.293065	149087	134128	134129	2	3	0.021565	1	0.059318	1

chr8	15807734	15807734	-0.34938	0.349382	173128	193698	193698	1	1	0.021618	1	0.038656	1
chr8	637909	638330	0.146434	0.292868	171707	190788	190789	2	3	0.02162	1	0.059417	1
chr13	1.13E+08	1.13E+08	0.349275	0.349275	53243	294777	294777	1	4	0.021635	1	0.038685	1
chr5	1951028	1951028	0.349061	0.349061	139312	113919	113919	1	5	0.021672	1	0.038745	1
chr10	1.35E+08	1.35E+08	0.146254	0.292508	28248	238307	238308	2	2	0.021718	1	0.059599	1
chr1	957119	957119	0.348766	0.348766	113	314	314	1	14	0.021721	1	0.038823	1
chr12	58970138	58970138	-0.3487	0.348697	43769	274366	274366	1	1	0.021733	1	0.038842	1
chr14	55617843	55617843	-0.34869	0.348689	55573	300412	300412	1	1	0.021733	1	0.038844	1
chr11	2295852	2295852	-0.34862	0.348618	29226	241359	241359	1	2	0.021745	1	0.038863	1
chr14	1.03E+08	1.03E+08	-0.1462	0.292391	59079	307715	307716	2	3	0.021748	1	0.059658	1
chr3	1.86E+08	1.86E+08	-0.14618	0.292356	129102	92180	92181	2	9	0.021758	1	0.059676	1
chr19	12547760	12547760	0.348397	0.348397	89691	379121	379121	1	1	0.021782	1	0.038924	1
chr10	1.27E+08	1.27E+08	0.348333	0.348333	27130	235205	235205	1	2	0.021792	1	0.03894	1
chr15	79788143	79788143	-0.34829	0.348295	64674	319614	319614	1	1	0.021798	1	0.03895	1
chr5	1.5E+08	1.5E+08	-0.1461	0.292192	146280	128207	128208	2	10	0.021804	1	0.059759	1
chr11	89610012	89610012	-0.34794	0.34794	36232	257717	257717	1	1	0.021861	1	0.039053	1
chr19	519609	519611	-0.14598	0.291956	86944	372293	372294	2	3	0.021867	1	0.059877	1
chr20	34638489	34638493	-0.14588	0.291756	112190	398441	398442	2	10	0.021925	1	0.059982	1
chr2	36771024	36771024	0.347317	0.347317	98334	46849	46849	1	1	0.021969	1	0.039224	1
chr2	2.2E+08	2.2E+08	0.347244	0.347244	108222	66644	66644	1	1	0.021983	1	0.039247	1
chr20	42574362	42574362	-0.34687	0.346867	112529	399430	399430	1	3	0.022049	1	0.039353	1
chr7	1955432	1955738	0.145652	0.291303	160674	166622	166623	2	6	0.022049	1	0.060213	1
chr19	55995271	55995271	0.346768	0.346768	95107	393047	393047	1	13	0.022066	1	0.039381	1
chr19	911815	911965	0.145604	0.291207	87119	372686	372687	2	3	0.022076	1	0.060266	1
chr2	2.22E+08	2.22E+08	-0.1456	0.291204	108411	67078	67079	2	18	0.022077	1	0.060267	1
chr3	1.2E+08	1.2E+08	-0.34663	0.346631	125273	84293	84293	1	1	0.02209	1	0.039419	1
chr2	1.14E+08	1.14E+08	-0.34652	0.346524	102876	56111	56111	1	1	0.022108	1	0.039448	1
chr1	63249793	63249836	0.145474	0.290949	8279	18875	18876	2	10	0.022146	1	0.060396	1
chr17	16593613	16593885	-0.14542	0.290835	76912	347776	347777	2	6	0.022179	1	0.060454	1
chr12	1.29E+08	1.29E+08	0.345992	0.345992	47982	283360	283360	1	1	0.022204	1	0.039601	1
chr6	1.06E+08	1.06E+08	0.145372	0.290745	155746	155826	155827	2	10	0.022204	1	0.060501	1
chr17	19280571	19280802	0.145292	0.290584	77300	348578	348579	2	17	0.022249	1	0.060581	1
chr2	2.42E+08	2.42E+08	-0.3457	0.345697	110323	71084	71084	1	2	0.022256	1	0.039683	1
chr1	38062058	38062058	-0.34567	0.345675	6063	13946	13946	1	7	0.022259	1	0.03969	1
chr1	3606550	3606550	0.345659	0.345659	1325	3708	3708	1	14	0.022262	1	0.039694	1
chr2	1.6E+08	1.6E+08	-0.34551	0.345512	105064	60161	60161	1	1	0.022289	1	0.039737	1
chr19	48614733	48614773	-0.1452	0.29041	93626	389272	389273	2	2	0.022297	1	0.060672	1
chr14	1.03E+08	1.03E+08	-0.34529	0.345294	59085	307730	307730	1	1	0.022328	1	0.039797	1
chr19	53142106	53142146	0.145115	0.290229	94566	391682	391683	2	9	0.022345	1	0.060766	1
chr4	39460490	39460490	-0.34506	0.345056	132883	100489	100489	1	18	0.022367	1	0.039862	1
chr5	649884	650104	-0.145	0.290003	138861	112619	112620	2	2	0.022406	1	0.060882	1
chr1	2844356	2844356	0.344832	0.344832	967	2717	2717	1	2	0.022408	1	0.039926	1
chr17	77096009	77096009	-0.34465	0.344655	83350	363114	363114	1	1	0.022441	1	0.039977	1
chr5	33515546	33515546	0.344467	0.344467	140555	116261	116261	1	2	0.022474	1	0.040031	1
chr8	961961	961961	-0.34409	0.34409	171821	191056	191056	1	4	0.022542	1	0.040137	1
chr11	3168455	3168455	-0.34382	0.343819	29451	242178	242178	1	4	0.02259	1	0.040213	1
chr5	1.81E+08	1.81E+08	-0.34363	0.343633	148711	133277	133277	1	6	0.022626	1	0.040267	1
chr17	63770753	63770753	-0.34359	0.343592	81859	359603	359603	1	1	0.022632	1	0.040278	1
chr11	33760479	33760479	0.343435	0.343435	31454	246485	246485	1	1	0.022661	1	0.040322	1
chr5	77930038	77930038	-0.34336	0.343364	142547	120296	120296	1	1	0.022673	1	0.040343	1
chr16	57023022	57023191	0.144485	0.28897	71121	334172	334173	2	12	0.022694	1	0.061425	1
chr2	61153802	61153802	-0.34304	0.343044	99719	49457	49457	1	1	0.022731	1	0.040436	1
chr3	1.13E+08	1.13E+08	-0.34279	0.342787	124960	83737	83737	1	1	0.022778	1	0.040512	1
chr16	57562740	57562841	-0.14428	0.288558	71195	334346	334347	2	6	0.022813	1	0.061644	1
chr17	73559562	73559562	-0.34241	0.342415	82633	361301	361301	1	4	0.022847	1	0.040618	1
chr17	22193895	22193895	0.341697	0.341697	77601	349262	349262	1	3	0.022977	1	0.04083	1
chr15	30930499	30930499	0.3416	0.3416	60680	311283	311283	1	1	0.022996	1	0.04086	1
chr6	26189355	26189355	0.341587	0.341587	150818	137512	137512	1	8	0.022998	1	0.040864	1
chr15	74266972	74267168	-0.14395	0.287894	64013	318078	318079	2	2	0.023	1	0.062	1
chr21	15436260	15436419	-0.14394	0.287884	114284	403647	403648	2	2	0.023003	1	0.062005	1
chr12	76938908	76938908	0.341551	0.341551	44544	275829	275829	1	1	0.023003	1	0.040873	1
chr10	1.19E+08	1.19E+08	-0.14392	0.287833	26464	233786	233787	2	6	0.023017	1	0.062033	1
chr21	37437565	37437565	0.34136	0.34136	114750	404753	404753	1	2	0.02304	1	0.040931	1
chr1	1.48E+08	1.48E+08	-0.14384	0.287685	11321	25473	25474	2	5	0.023059	1	0.06211	1
chr2	1.75E+08	1.75E+08	-0.14383	0.287655	105898	61812	61813	2	2	0.023066	1	0.062127	1
chr6	30457562	30457724	-0.14381	0.287622	151588	141442	141443	2	49	0.023077	1	0.062146	1
chr7	1.28E+08	1.28E+08	0.143782	0.287563	168506	183616	183617	2	9	0.023096	1	0.06218	1
chr3	1.96E+08	1.96E+08	-0.34093	0.340931	129858	93603	93603	1	3	0.023117	1	0.041054	1
chr21	43185674	43185803	-0.14368	0.287353	115049	405433	405434	2	6	0.023152	1	0.062289	1
chr22	31503709	31503747	-0.14367	0.287342	117167	410096	410097	2	8	0.023156	1	0.062296	1
chr2	2.03E+08	2.03E+08	0.340715	0.340715	107144	64521	64521	1	1	0.023157	1	0.04112	1
chr7	2643340	2643340	-0.34069	0.340692	160939	167391	167391	1	3	0.023162	1	0.041127	1
chr6	28831858	28831873	-0.14354	0.287079	151252	139105	139106	2	78	0.023228	1	0.062436	1
chr3	64127184	64127184	-0.34021	0.340215	123355	80938	80938	1	1	0.02325	1	0.041266	1

chr7	1.29E+08	1.29E+08	0.340105	0.340105	168713	184092	184092	1	1	0.023271	1	0.041299	1
chr14	1.07E+08	1.07E+08	-0.33994	0.339944	59929	309683	309683	1	2	0.023301	1	0.041348	1
chr19	53697534	53697534	-0.33986	0.339861	94633	391853	391853	1	11	0.023317	1	0.041373	1
chr5	81402204	81402204	0.339587	0.339587	142763	120706	120706	1	1	0.023371	1	0.041459	1
chr19	17008650	17008740	-0.14325	0.286494	90403	381025	381026	2	8	0.023399	1	0.062751	1
chr4	1.77E+08	1.77E+08	-0.33912	0.339125	137791	110368	110368	1	1	0.02346	1	0.041602	1
chr13	46357647	46357904	-0.14314	0.28627	50723	289592	289593	2	3	0.023461	1	0.062869	1
chr6	1.53E+08	1.53E+08	0.338538	0.338538	158199	160695	160695	1	19	0.023569	1	0.041776	1
chr1	3052501	3052501	0.338397	0.338397	1048	2946	2946	1	6	0.023595	1	0.041818	1
chr7	72722760	72722923	0.142897	0.285794	165288	176587	176588	2	13	0.023597	1	0.063128	1
chr1	39174395	39174395	0.338047	0.338047	6197	14258	14258	1	7	0.02366	1	0.041923	1
chr5	1.12E+08	1.12E+08	0.337999	0.337999	143737	122555	122555	1	1	0.023669	1	0.041936	1
chr6	1.68E+08	1.68E+08	0.337963	0.337963	159395	163102	163102	1	1	0.023676	1	0.041948	1
chr10	75674740	75674740	-0.33774	0.337744	23385	227135	227135	1	2	0.023717	1	0.042015	1
chr16	2155961	2155961	0.337714	0.337714	67347	325908	325908	1	1	0.023723	1	0.042024	1
chr11	18034626	18034626	0.337712	0.337712	30763	244873	244873	1	12	0.023723	1	0.042025	1
chr19	34749411	34749411	-0.33766	0.337659	91480	383775	383775	1	1	0.023733	1	0.04204	1
chr5	1.8E+08	1.8E+08	0.337356	0.337356	148700	133246	133246	1	5	0.023791	1	0.042133	1
chr13	1.15E+08	1.15E+08	-0.33728	0.33728	53807	296464	296464	1	4	0.023807	1	0.042156	1
chr11	12845864	12845864	-0.33725	0.337246	30409	244127	244127	1	1	0.023813	1	0.042166	1
chr7	675570	676060	-0.14253	0.285064	160133	164948	164949	2	4	0.023814	1	0.063531	1
chr3	12045449	12045459	-0.1425	0.284998	119746	73212	73213	2	9	0.023833	1	0.063569	1
chr6	1.7E+08	1.7E+08	0.142433	0.284865	159815	164122	164123	2	2	0.02387	1	0.06364	1
chr15	93655850	93655850	-0.33692	0.336922	65777	321911	321911	1	1	0.023877	1	0.042267	1
chr7	27185393	27185393	-0.33674	0.336739	162555	171202	171202	1	51	0.023909	1	0.042322	1
chr2	8829827	8829827	0.336688	0.336688	96481	43113	43113	1	1	0.02392	1	0.042339	1
chr16	20750043	20750043	-0.33654	0.336535	69067	329532	329532	1	1	0.02395	1	0.042389	1
chr8	1.17E+08	1.17E+08	0.336286	0.336286	178008	203528	203528	1	1	0.023999	1	0.042465	1
chr8	1.43E+08	1.43E+08	0.336207	0.336207	179690	206518	206518	1	2	0.024014	1	0.04249	1
chr13	1.12E+08	1.12E+08	0.336041	0.336041	53102	294329	294329	1	1	0.024045	1	0.042539	1
chr1	2.15E+08	2.15E+08	-0.33597	0.335973	16329	36279	36279	1	1	0.024057	1	0.042559	1
chr15	21940895	21941178	-0.14203	0.284059	60001	309793	309794	2	3	0.024109	1	0.064091	1
chr2	83812230	83812230	0.335667	0.335667	101080	52428	52428	1	1	0.024116	1	0.042653	1
chr5	28927606	28927606	-0.33537	0.335368	140387	115968	115968	1	1	0.024173	1	0.042743	1
chr12	1.31E+08	1.31E+08	0.335181	0.335181	48158	283781	283781	1	3	0.024209	1	0.0428	1
chr19	55385405	55385405	0.335168	0.335168	94947	392646	392646	1	3	0.024211	1	0.042804	1
chr17	74270190	74270190	-0.33504	0.335038	82803	361761	361761	1	1	0.024236	1	0.042844	1
chr22	47026467	47026467	0.334981	0.334981	118592	413547	413547	1	1	0.024247	1	0.042861	1
chr6	31698058	31698089	-0.14176	0.283527	151887	143978	143979	2	72	0.024271	1	0.064391	1
chr1	1.47E+08	1.47E+08	0.334451	0.334451	11226	25247	25247	1	4	0.024353	1	0.043029	1
chr8	1.29E+08	1.29E+08	0.334419	0.334419	178720	204770	204770	1	1	0.02436	1	0.043039	1
chr4	10020882	10021025	-0.14154	0.283083	131919	98712	98713	2	6	0.024404	1	0.064645	1
chr6	32630856	32630856	0.334134	0.334134	152160	146438	146438	1	1	0.024415	1	0.043127	1
chr6	1.31E+08	1.31E+08	0.333385	0.333385	156878	158112	158112	1	1	0.024568	1	0.043368	1
chr16	33357426	33357426	-0.33334	0.333344	70301	332452	332452	1	3	0.024576	1	0.04338	1
chr16	1139733	1139733	0.333018	0.333018	66889	324585	324585	1	1	0.024645	1	0.043487	1
chr22	46508451	46508451	0.332969	0.332969	118505	413336	413336	1	6	0.024656	1	0.043503	1
chr9	1.36E+08	1.36E+08	0.141053	0.282106	184862	215656	215657	2	3	0.024705	1	0.065209	1
chr12	1.33E+08	1.33E+08	-0.3327	0.332704	48592	284857	284857	1	5	0.024708	1	0.043589	1
chr11	2322507	2322517	-0.14105	0.282093	29236	241386	241387	2	30	0.02471	1	0.065216	1
chr8	1792758	1792758	0.332637	0.332637	172113	191756	191756	1	2	0.024723	1	0.043612	1
chr9	1.08E+08	1.08E+08	0.332489	0.332489	183000	212753	212753	1	2	0.024753	1	0.043658	1
chr16	86457593	86457593	-0.33238	0.332382	73522	339350	339350	1	4	0.024774	1	0.043693	1
chr17	43578911	43578911	-0.33227	0.332272	79986	355229	355229	1	3	0.024795	1	0.043728	1
chr1	19291857	19291857	0.332132	0.332132	3735	8614	8614	1	1	0.024822	1	0.043771	1
chr1	12600225	12600529	-0.14086	0.281726	2857	6900	6901	2	3	0.024824	1	0.065433	1
chr5	1.8E+08	1.8E+08	-0.33196	0.331962	148689	133221	133221	1	1	0.024857	1	0.043827	1
chr11	660455	660455	-0.33188	0.331875	28661	239550	239550	1	3	0.024875	1	0.043855	1
chr10	22292468	22292568	0.140781	0.281562	20500	221376	221377	2	14	0.024876	1	0.065529	1
chr6	31164804	31164914	-0.14077	0.281541	151767	142817	142818	2	31	0.024881	1	0.065541	1
chr11	1.34E+08	1.34E+08	0.140763	0.281526	39233	263849	263850	2	4	0.024886	1	0.065549	1
chr4	1062280	1062280	0.33176	0.33176	130421	95083	95083	1	1	0.024898	1	0.043892	1
chr18	12777645	12777786	0.140675	0.28135	85040	367939	367940	2	4	0.024937	1	0.065649	1
chr17	61518241	61518241	-0.3314	0.3314	81599	358992	358992	1	6	0.024973	1	0.04401	1
chr5	1.1E+08	1.1E+08	-0.33136	0.331358	143659	122402	122402	1	1	0.02498	1	0.044021	1
chr20	37230326	37230386	0.140585	0.28117	112385	399014	399015	2	7	0.024993	1	0.065755	1
chr6	30079203	30079256	-0.14047	0.280947	151516	140724	140725	2	23	0.025062	1	0.065885	1
chr12	34457254	34457254	0.330961	0.330961	41752	269195	269195	1	4	0.025063	1	0.044149	1
chr12	76414534	76414534	-0.33096	0.330958	44511	275771	275771	1	2	0.025063	1	0.04415	1
chr1	1.55E+08	1.55E+08	0.330899	0.330899	12220	27842	27842	1	13	0.025076	1	0.04417	1
chr19	844589	844589	0.330853	0.330853	87087	372614	372614	1	1	0.025086	1	0.044184	1
chr11	58944103	58944103	0.330793	0.330793	32839	249304	249304	1	1	0.025099	1	0.044204	1
chr7	1.5E+08	1.5E+08	-0.3303	0.330302	170245	187102	187102	1	1	0.0252	1	0.044367	1
chr11	67568593	67568593	-0.33021	0.330214	34420	253768	253768	1	1	0.025218	1	0.044397	1

chr5	2754240	2754240	-0.33016	0.330155	139414	114185	114185	1	34	0.02523	1	0.044415	1
chr6	30711805	30711805	0.330072	0.330072	151658	142226	142226	1	72	0.025248	1	0.044445	1
chr16	1722957	1722957	0.329635	0.329635	67173	325400	325400	1	1	0.02534	1	0.04459	1
chr12	1973367	1973367	0.329577	0.329577	39610	264672	264672	1	7	0.025351	1	0.044608	1
chr6	1.71E+08	1.71E+08	0.139963	0.279926	159895	164313	164314	2	4	0.025382	1	0.066486	1
chr17	54858770	54858770	-0.32931	0.329313	81005	357687	357687	1	1	0.025407	1	0.044694	1
chr10	43632967	43633030	0.139872	0.279744	21549	223416	223417	2	15	0.02544	1	0.066597	1
chr8	1.41E+08	1.41E+08	-0.32912	0.329122	179328	205697	205697	1	6	0.025448	1	0.044756	1
chr7	47990680	47990680	0.329115	0.329115	164145	174402	174402	1	1	0.025449	1	0.044758	1
chr22	49765138	49765229	0.139844	0.279687	118766	413845	413846	2	3	0.025458	1	0.066631	1
chr16	8806531	8806569	-0.13983	0.279657	68248	328023	328024	2	11	0.025469	1	0.066651	1
chr11	1.35E+08	1.35E+08	0.328878	0.328878	39357	264179	264179	1	1	0.025499	1	0.044837	1
chr8	496327	496440	-0.13974	0.279471	171673	190714	190715	2	4	0.025528	1	0.066758	1
chr17	74585455	74585455	-0.3286	0.328603	82878	361968	361968	1	1	0.025555	1	0.044928	1
chr4	38872591	38872591	-0.32855	0.328545	132838	100399	100399	1	1	0.025566	1	0.044945	1
chr8	1893887	1893887	-0.3285	0.328501	172153	191871	191871	1	4	0.025576	1	0.04496	1
chr10	73136913	73136972	0.139629	0.279259	23090	226508	226509	2	5	0.025592	1	0.066881	1
chr13	27832751	27832751	0.3283	0.3283	49586	287258	287258	1	1	0.025618	1	0.045028	1
chr6	1.54E+08	1.54E+08	0.328239	0.328239	158233	160760	160760	1	1	0.025631	1	0.045048	1
chr2	2.33E+08	2.33E+08	-0.13953	0.27907	109029	68294	68295	2	5	0.025651	1	0.066992	1
chr3	22422855	22422855	0.328097	0.328097	120420	74466	74466	1	2	0.025661	1	0.045095	1
chr6	21136536	21136536	0.328064	0.328064	150484	136783	136783	1	1	0.025668	1	0.045106	1
chr1	1.17E+08	1.17E+08	0.328057	0.328057	10737	24223	24223	1	9	0.02567	1	0.045109	1
chr7	533963	533963	0.327969	0.327969	160064	164749	164749	1	1	0.025689	1	0.045138	1
chr10	1.35E+08	1.35E+08	0.327941	0.327941	28095	237780	237780	1	7	0.025694	1	0.045146	1
chr11	32605279	32605281	-0.13943	0.278851	31360	246285	246286	2	12	0.025721	1	0.067122	1
chr8	980822	980822	0.327761	0.327761	171832	191079	191079	1	1	0.025734	1	0.045208	1
chr16	33070551	33070632	-0.13936	0.27872	70293	332439	332440	2	2	0.025764	1	0.067202	1
chr12	57040199	57040204	-0.13935	0.278692	43502	273606	273607	2	13	0.025774	1	0.06722	1
chr10	22768167	22768167	0.327531	0.327531	20538	221514	221514	1	3	0.025782	1	0.045284	1
chr3	52869266	52869266	0.327442	0.327442	122668	79660	79660	1	6	0.0258	1	0.045312	1
chr17	4502244	4502244	-0.32736	0.327359	75623	344475	344475	1	1	0.025817	1	0.045339	1
chr12	1.25E+08	1.25E+08	-0.32722	0.327223	47593	282576	282576	1	4	0.025847	1	0.045386	1
chr17	40808068	40808068	0.327148	0.327148	79464	353766	353766	1	1	0.025863	1	0.04541	1
chr17	38120485	38120485	-0.32706	0.327056	78938	352511	352511	1	1	0.025883	1	0.04544	1
chr12	4381803	4381882	-0.13905	0.278102	39905	265247	265248	2	53	0.025958	1	0.067572	1
chr20	50384822	50384822	0.326581	0.326581	113100	400859	400859	1	6	0.025981	1	0.0456	1
chr11	65360123	65360327	0.138965	0.27793	33903	252194	252195	2	4	0.026012	1	0.067678	1
chr15	72668543	72668568	-0.13893	0.277868	63872	317810	317811	2	10	0.026032	1	0.067715	1
chr6	1.68E+08	1.68E+08	0.326331	0.326331	159265	162830	162830	1	2	0.026033	1	0.045685	1
chr11	27883440	27883440	0.326331	0.326331	31187	245829	245829	1	1	0.026033	1	0.045685	1
chr6	20032755	20032841	0.138904	0.277809	150423	136688	136689	2	3	0.026052	1	0.06775	1
chr18	52258182	52258234	-0.1389	0.277794	85908	370076	370077	2	2	0.026056	1	0.067759	1
chr7	4855406	4855406	-0.32593	0.325934	161263	168245	168245	1	4	0.026118	1	0.045819	1
chr9	1.4E+08	1.4E+08	0.325889	0.325889	185599	216770	216770	1	1	0.026128	1	0.045835	1
chr8	28745972	28745972	-0.32587	0.325868	174162	195800	195800	1	2	0.026134	1	0.045843	1
chr6	32805748	32805759	0.138695	0.27739	152195	146618	146619	2	38	0.026183	1	0.068005	1
chr19	48698419	48698632	-0.13869	0.277385	93643	389302	389303	2	2	0.026185	1	0.068009	1
chr15	81426347	81426360	0.138667	0.277333	64820	319846	319847	2	10	0.026202	1	0.068042	1
chr1	1.61E+08	1.61E+08	0.138659	0.277317	13015	29690	29691	2	3	0.026208	1	0.068052	1
chr4	56660143	56660328	-0.1386	0.277205	133541	101940	101941	2	7	0.026244	1	0.068119	1
chr2	2.41E+08	2.41E+08	-0.32524	0.325235	110108	70582	70582	1	1	0.026267	1	0.046055	1
chr15	71747687	71747687	0.325104	0.325104	63796	317669	317669	1	1	0.026294	1	0.046097	1
chr1	1.82E+08	1.82E+08	0.325095	0.325095	14247	32162	32162	1	1	0.026295	1	0.0461	1
chr1	2E+08	2E+08	-0.13852	0.277033	14843	33243	33244	2	7	0.026296	1	0.068223	1
chr20	388473	388473	0.324934	0.324934	110697	394646	394646	1	10	0.026329	1	0.046154	1
chr7	807596	807608	-0.13843	0.276859	160180	165074	165075	2	5	0.026353	1	0.068331	1
chr3	1.34E+08	1.34E+08	-0.13843	0.276858	126583	86994	86995	2	15	0.026354	1	0.068331	1
chr20	18478185	18478185	-0.32477	0.324773	111424	396465	396465	1	9	0.026366	1	0.04621	1
chr7	766100	766104	0.138406	0.276813	160156	165004	165005	2	8	0.02637	1	0.068359	1
chr10	95463028	95463124	0.138343	0.276686	24699	229626	229627	2	13	0.026411	1	0.068437	1
chr10	1.35E+08	1.35E+08	0.324515	0.324515	28197	238136	238136	1	17	0.026423	1	0.046299	1
chr12	8995965	8995965	-0.32444	0.324437	40460	266663	266663	1	4	0.02644	1	0.046325	1
chr7	39649706	39649706	-0.3243	0.324295	163492	173108	173108	1	5	0.026472	1	0.046374	1
chr16	1797050	1797050	0.324269	0.324269	67199	325466	325466	1	6	0.026477	1	0.046383	1
chr1	2.2E+08	2.2E+08	0.324132	0.324132	16540	36641	36641	1	1	0.026507	1	0.046429	1
chr5	1.8E+08	1.8E+08	-0.324	0.324	148645	133118	133118	1	3	0.026535	1	0.046474	1
chr7	1.57E+08	1.57E+08	-0.32388	0.323882	171055	189127	189127	1	7	0.026561	1	0.046513	1
chr1	45672383	45672383	0.323802	0.323802	7014	16195	16195	1	12	0.026579	1	0.046542	1
chr2	1843120	1843136	0.138043	0.276086	95953	41998	41999	2	6	0.026604	1	0.068816	1
chr17	75033233	75033339	0.138013	0.276026	82946	362126	362127	2	2	0.026623	1	0.068854	1
chr14	1.04E+08	1.04E+08	-0.32346	0.323462	59264	308152	308152	1	9	0.026657	1	0.046662	1
chr18	7117321	7117680	0.137921	0.275842	84821	367426	367427	2	9	0.026683	1	0.068972	1
chr7	884056	884056	0.323016	0.323016	160213	165174	165174	1	9	0.026758	1	0.046818	1

chr8	1.26E+08	1.26E+08	0.322983	0.322983	178537	204479	204479	1	4	0.026765	1	0.046829	1
chr17	47207658	47207658	0.322846	0.322846	80465	356475	356475	1	1	0.026794	1	0.046875	1
chr5	1.41E+08	1.41E+08	-0.13768	0.275362	145638	126956	126957	2	3	0.026835	1	0.069269	1
chr6	28584167	28584172	-0.13766	0.275313	151208	138872	138873	2	19	0.026852	1	0.069301	1
chr11	300286	300404	-0.13763	0.275259	28493	239024	239025	2	4	0.026869	1	0.069336	1
chr8	11212812	11212812	-0.3225	0.322503	172836	193160	193160	1	2	0.02687	1	0.046997	1
chr3	1.94E+08	1.94E+08	0.137603	0.275206	129612	93099	93100	2	6	0.026886	1	0.069369	1
chr13	27787332	27787332	0.322397	0.322397	49580	287238	287238	1	1	0.026894	1	0.047034	1
chr14	63671356	63671737	0.137588	0.275176	56059	301413	301414	2	6	0.026897	1	0.06939	1
chr16	57318697	57318709	-0.13754	0.275085	71153	334255	334256	2	14	0.026928	1	0.069449	1
chr13	1.11E+08	1.11E+08	-0.32217	0.322168	52916	293894	293894	1	7	0.026945	1	0.047116	1
chr12	64080144	64080144	0.322023	0.322023	43894	274605	274605	1	2	0.026977	1	0.047167	1
chr11	61781116	61781116	-0.32202	0.32202	33188	250128	250128	1	1	0.026978	1	0.047168	1
chr6	1.57E+08	1.57E+08	-0.32199	0.321989	158423	161040	161040	1	3	0.026984	1	0.047178	1
chr12	1.3E+08	1.3E+08	0.137441	0.274883	48018	283441	283442	2	2	0.026993	1	0.069576	1
chr6	55778951	55778951	0.321855	0.321855	154344	152865	152865	1	1	0.027017	1	0.047228	1
chr2	99952887	99952887	-0.32169	0.321692	101948	54414	54414	1	17	0.027052	1	0.047284	1
chr2	65955222	65955240	0.137223	0.274446	100061	50195	50196	2	2	0.027142	1	0.06986	1
chr1	2.01E+08	2.01E+08	0.321275	0.321275	14918	33397	33397	1	2	0.027149	1	0.047433	1
chr6	29714059	29714059	-0.32127	0.321272	151414	140040	140040	1	1	0.02715	1	0.047434	1
chr6	1.33E+08	1.33E+08	-0.32103	0.321034	156997	158303	158303	1	1	0.027201	1	0.047518	1
chr4	1.44E+08	1.44E+08	0.320941	0.320941	136583	107919	107919	1	1	0.027223	1	0.047551	1
chr2	2757161	2757161	-0.32071	0.320715	96035	42179	42179	1	1	0.027276	1	0.047634	1
chr5	1.41E+08	1.41E+08	-0.32068	0.320678	145659	127046	127046	1	1	0.027284	1	0.047648	1
chr10	4069749	4069749	0.32048	0.32048	19432	219173	219173	1	1	0.02733	1	0.047719	1
chr11	73661250	73661250	-0.32025	0.320254	35338	255980	255980	1	1	0.02738	1	0.047797	1
chr1	55260950	55260950	-0.32018	0.320179	7810	18063	18063	1	1	0.027397	1	0.047823	1
chr6	32449961	32449961	0.320178	0.320178	152128	146376	146376	1	1	0.027397	1	0.047823	1
chr2	1.33E+08	1.33E+08	-0.1367	0.273399	104066	58491	58492	2	9	0.027491	1	0.070536	1
chr11	66362959	66362959	-0.31905	0.319047	34141	252996	252996	1	1	0.027657	1	0.04823	1
chr12	1.25E+08	1.25E+08	-0.31862	0.318625	47613	282621	282621	1	4	0.027759	1	0.048388	1
chr19	1035450	1035450	0.318572	0.318572	87170	372822	372822	1	2	0.027771	1	0.048408	1
chr5	3182057	3182108	0.136248	0.272495	139450	114264	114265	2	3	0.027798	1	0.071125	1
chr7	64035417	64035529	-0.13624	0.272481	164838	175755	175756	2	3	0.027802	1	0.071133	1
chr12	28343237	28343237	0.318213	0.318213	41482	268688	268688	1	5	0.027853	1	0.048539	1
chr8	19616280	19616280	-0.31795	0.317946	173355	194082	194082	1	1	0.027914	1	0.048637	1
chr21	43372922	43372922	-0.3178	0.317799	115095	405519	405519	1	9	0.02795	1	0.048693	1
chr2	46043	46465	0.135927	0.271855	95618	41256	41257	2	3	0.02801	1	0.071544	1
chr20	4721766	4721766	-0.31754	0.317543	111122	395684	395684	1	5	0.028012	1	0.048789	1
chr12	1.31E+08	1.31E+08	-0.31752	0.317518	48121	283657	283657	1	1	0.028018	1	0.048798	1
chr7	1.53E+08	1.53E+08	-0.31747	0.317465	170588	187949	187949	1	3	0.02803	1	0.048817	1
chr7	1.59E+08	1.59E+08	-0.13583	0.271663	171599	190577	190578	2	3	0.028075	1	0.071671	1
chr7	1.37E+08	1.37E+08	0.317265	0.317265	169249	185140	185140	1	1	0.028077	1	0.04889	1
chr6	32141882	32141882	-0.31709	0.317089	152032	145862	145862	1	13	0.028119	1	0.048956	1
chr13	28540727	28540727	0.317008	0.317008	49663	287481	287481	1	5	0.028138	1	0.048986	1
chr1	2.21E+08	2.21E+08	0.31691	0.31691	16626	36851	36851	1	1	0.028162	1	0.049024	1
chr16	57334839	57335017	0.13567	0.27134	71157	334269	334270	2	6	0.028184	1	0.071888	1
chr17	17109800	17109817	0.135569	0.271137	76964	347867	347868	2	13	0.02825	1	0.072023	1
chr1	6011302	6011302	-0.31641	0.316409	1583	4294	4294	1	1	0.028283	1	0.049211	1
chr22	27053402	27053413	0.135516	0.271031	116883	409371	409372	2	15	0.028288	1	0.072098	1
chr17	77435199	77435263	-0.13546	0.270917	83409	363252	363253	2	2	0.028329	1	0.072178	1
chr9	1.25E+08	1.25E+08	-0.31584	0.31584	183644	213742	213742	1	1	0.02842	1	0.049424	1
chr6	31997059	31997067	0.135313	0.270626	151978	145008	145009	2	5	0.028429	1	0.072376	1
chr6	37616410	37616482	-0.13528	0.270565	152958	150049	150050	2	5	0.028448	1	0.072415	1
chr6	31588707	31588727	0.135281	0.270562	151841	143371	143372	2	41	0.028449	1	0.072418	1
chr8	1.02E+08	1.02E+08	-0.13522	0.270437	177523	202626	202627	2	6	0.028493	1	0.072502	1
chr2	1.69E+08	1.69E+08	0.315545	0.315545	105396	60786	60786	1	1	0.028493	1	0.049537	1
chr12	97304436	97304436	-0.31546	0.315455	45296	277203	277203	1	1	0.028512	1	0.04957	1
chr4	25090298	25090491	-0.13519	0.270373	132491	99777	99778	2	6	0.028514	1	0.072545	1
chr2	1.61E+08	1.61E+08	-0.31533	0.315326	105091	60225	60225	1	1	0.028547	1	0.049622	1
chr6	29759078	29759078	-0.31518	0.315181	151424	140120	140120	1	1	0.02858	1	0.049677	1
chr7	91808500	91808500	-0.31508	0.31508	166274	178629	178629	1	5	0.028605	1	0.049715	1
chr13	41240516	41240761	-0.13499	0.269973	50358	288868	288869	2	8	0.028652	1	0.072816	1
chr1	1.63E+08	1.63E+08	-0.31483	0.31483	13200	30046	30046	1	1	0.028667	1	0.049813	1
chr19	38308263	38308458	0.134963	0.269926	91956	385135	385136	2	5	0.028667	1	0.072848	1
chr2	24367828	24367828	-0.31456	0.314557	97489	45005	45005	1	3	0.028738	1	0.049921	1
chr16	1116010	1116106	0.134842	0.269684	66875	324541	324542	2	7	0.028749	1	0.07301	1
chr1	16472728	16473143	-0.13484	0.269675	3278	7700	7701	2	3	0.028752	1	0.073017	1
chr19	34789667	34789667	-0.31442	0.314418	91481	383776	383776	1	1	0.028776	1	0.049978	1
chr7	3227262	3227277	-0.13479	0.269582	161064	167732	167733	2	2	0.028783	1	0.073078	1
chr1	97050305	97050305	-0.31415	0.314151	9785	22015	22015	1	1	0.028843	1	0.050081	1
chr1	1.09E+08	1.09E+08	-0.31398	0.313984	10057	22602	22602	1	1	0.028884	1	0.050146	1
chr2	1.32E+08	1.32E+08	-0.13455	0.269097	103947	58256	58257	2	4	0.028951	1	0.073406	1
chr1	2E+08	2E+08	-0.13453	0.269062	14839	33229	33230	2	15	0.028964	1	0.073431	1

chr7	1.4E+08	1.4E+08	-0.31363	0.313632	169521	185624	185624	1	4	0.028972	1	0.050283	1
chr10	1.34E+08	1.34E+08	0.134376	0.268752	27760	236714	236715	2	3	0.029071	1	0.073647	1
chr3	66002958	66002958	-0.31322	0.313219	123487	81118	81118	1	2	0.029072	1	0.050441	1
chr19	4047785	4048075	0.134369	0.268739	88219	375328	375329	2	4	0.029075	1	0.073655	1
chr12	9446913	9446913	-0.31311	0.313111	40493	266740	266740	1	3	0.029098	1	0.050481	1
chr9	1.25E+08	1.25E+08	-0.1343	0.268598	183646	213746	213747	2	8	0.029124	1	0.073751	1
chr1	89829871	89829871	-0.31301	0.313008	9345	21028	21028	1	3	0.029126	1	0.050523	1
chr1	10508703	10508703	0.312886	0.312886	2452	6078	6078	1	2	0.029156	1	0.05057	1
chr12	1.17E+08	1.17E+08	0.3127	0.3127	46766	280491	280491	1	1	0.029202	1	0.050643	1
chr6	10413233	10413394	-0.13419	0.268378	149704	135367	135368	2	18	0.029202	1	0.073906	1
chr8	1.25E+08	1.25E+08	0.134172	0.268345	178497	204395	204396	2	6	0.029215	1	0.073931	1
chr2	2.43E+08	2.43E+08	-0.1341	0.268205	110535	71573	71574	2	5	0.029262	1	0.074027	1
chr5	1.51E+08	1.51E+08	0.312349	0.312349	146452	128583	128583	1	1	0.029287	1	0.050779	1
chr19	932759	932813	0.134056	0.268112	87130	372729	372730	2	2	0.029297	1	0.074093	1
chr1	5937169	5937169	-0.31208	0.312081	1562	4244	4244	1	2	0.029351	1	0.050882	1
chr7	65375514	65375514	0.311859	0.311859	164944	175980	175980	1	1	0.029406	1	0.050971	1
chr2	1.53E+08	1.53E+08	-0.31178	0.311778	104789	59638	59638	1	1	0.029426	1	0.051004	1
chr12	4918391	4918848	0.13387	0.26774	39955	265400	265401	2	10	0.029427	1	0.074352	1
chr19	15582687	15582775	0.133822	0.267645	90239	380642	380643	2	3	0.029461	1	0.074421	1
chr9	91997686	91997686	-0.31143	0.311429	182263	211574	211574	1	1	0.02952	1	0.051146	1
chr2	2.38E+08	2.38E+08	-0.13367	0.267347	109567	69434	69435	2	2	0.029567	1	0.074635	1
chr17	71898817	71898904	-0.13365	0.267293	82351	360533	360534	2	7	0.029585	1	0.074674	1
chr19	37760583	37760594	-0.13363	0.267265	91900	384966	384967	2	2	0.029595	1	0.074693	1
chr2	2.4E+08	2.4E+08	-0.3111	0.311096	109928	70170	70170	1	3	0.0296	1	0.051276	1
chr2	2.39E+08	2.39E+08	0.310786	0.310786	109799	69887	69887	1	1	0.029679	1	0.051397	1
chr3	1.96E+08	1.96E+08	-0.31072	0.310725	129855	93597	93597	1	3	0.029694	1	0.051422	1
chr7	1.5E+08	1.5E+08	-0.31051	0.310513	170248	187106	187106	1	7	0.029746	1	0.051505	1
chr8	6722002	6722002	0.310465	0.310465	172391	192361	192361	1	1	0.02976	1	0.051526	1
chr12	1.31E+08	1.31E+08	-0.13336	0.266726	48135	283700	283701	2	3	0.029787	1	0.07507	1
chr11	1.17E+08	1.17E+08	0.31028	0.31028	37513	260311	260311	1	3	0.029806	1	0.0516	1
chr2	1.01E+08	1.01E+08	-0.31006	0.310061	102053	54578	54578	1	1	0.029864	1	0.051689	1
chr14	94943234	94943234	-0.31004	0.310041	58184	305637	305637	1	2	0.029869	1	0.051697	1
chr1	2.36E+08	2.36E+08	-0.13321	0.266415	17920	39509	39510	2	6	0.029897	1	0.075288	1
chr3	1.69E+08	1.69E+08	-0.30983	0.309829	128040	90052	90052	1	1	0.029923	1	0.051782	1
chr6	1615843	1615992	-0.13316	0.266327	148952	133881	133882	2	3	0.029929	1	0.075351	1
chr11	10326565	10326570	0.133149	0.266298	30162	243708	243709	2	16	0.029941	1	0.075373	1
chr3	1.7E+08	1.7E+08	0.133119	0.266238	128113	90217	90218	2	6	0.029962	1	0.075415	1
chr1	31983220	31983220	-0.30962	0.309621	5354	12290	12290	1	3	0.029978	1	0.051868	1
chr5	78282669	78282669	-0.30957	0.309565	142579	120347	120347	1	2	0.029992	1	0.05189	1
chr13	23499332	23499332	0.309547	0.309547	49246	286550	286550	1	11	0.029997	1	0.051897	1
chr20	60527061	60527061	0.309512	0.309512	113532	401977	401977	1	1	0.030006	1	0.051912	1
chr7	23695266	23695323	0.132957	0.265913	162337	170562	170563	2	3	0.030073	1	0.075644	1
chr2	75785554	75785554	-0.30921	0.309206	100919	52146	52146	1	2	0.030085	1	0.052037	1
chr1	13111908	13111908	0.309147	0.309147	2916	6994	6994	1	1	0.030099	1	0.052061	1
chr9	46862104	46862104	-0.30893	0.308932	181636	210655	210655	1	1	0.030153	1	0.052146	1
chr14	92410260	92410260	-0.30881	0.308811	57926	305107	305107	1	1	0.030184	1	0.052195	1
chr7	1.59E+08	1.59E+08	-0.30878	0.308779	171519	190362	190362	1	1	0.030191	1	0.052207	1
chr19	47987374	47987460	-0.13277	0.265547	93530	389078	389079	2	7	0.030204	1	0.075906	1
chr1	81878937	81878937	0.308728	0.308728	8983	20303	20303	1	1	0.030205	1	0.052228	1
chr17	47092156	47092178	-0.13256	0.265119	80451	356451	356452	2	9	0.030352	1	0.07621	1
chr11	364752	364752	-0.30816	0.30816	28519	239105	239105	1	4	0.030354	1	0.052463	1
chr2	1.35E+08	1.35E+08	0.30801	0.30801	104201	58683	58683	1	1	0.030393	1	0.052523	1
chr14	1.02E+08	1.02E+08	0.307859	0.307859	58897	307260	307260	1	4	0.030433	1	0.052587	1
chr7	2744621	2744621	0.307658	0.307658	160983	167514	167514	1	1	0.030487	1	0.052672	1
chr7	1133730	1133730	-0.30764	0.30764	160356	165634	165634	1	3	0.030492	1	0.05268	1
chr1	24306751	24306765	-0.13236	0.264711	4418	10060	10061	2	10	0.030496	1	0.076508	1
chr16	19179928	19179931	0.132347	0.264693	68957	329328	329329	2	8	0.030504	1	0.076522	1
chr14	55151579	55151579	0.307469	0.307469	55524	300322	300322	1	1	0.030536	1	0.052746	1
chr5	1.31E+08	1.31E+08	0.307293	0.307293	144543	124088	124088	1	4	0.030582	1	0.052821	1
chr16	8806663	8806690	-0.13223	0.264466	68248	328026	328027	2	11	0.030585	1	0.076687	1
chr17	77906735	77906735	-0.30728	0.307281	83506	363530	363530	1	1	0.030586	1	0.052827	1
chr19	53099796	53099796	-0.30722	0.307215	94562	391669	391669	1	1	0.030604	1	0.052856	1
chr11	62138599	62138845	-0.13218	0.264369	33224	250196	250197	2	2	0.030621	1	0.07676	1
chr10	1.35E+08	1.35E+08	0.307111	0.307111	28102	237810	237810	1	4	0.030632	1	0.052899	1
chr2	7342228	7342228	0.306946	0.306946	96375	42933	42933	1	1	0.030675	1	0.052966	1
chr1	19777949	19777949	-0.30686	0.30686	3825	8771	8771	1	1	0.030697	1	0.053	1
chr2	1.74E+08	1.74E+08	-0.13206	0.264115	105742	61511	61512	2	4	0.030712	1	0.076942	1
chr19	54567123	54567279	0.131968	0.263937	94806	392304	392305	2	3	0.030775	1	0.077071	1
chr2	2.19E+08	2.19E+08	-0.30649	0.306495	107977	66015	66015	1	1	0.030795	1	0.053154	1
chr1	2.15E+08	2.15E+08	-0.30649	0.306493	16335	36287	36287	1	1	0.030795	1	0.053155	1
chr1	2.08E+08	2.08E+08	0.306442	0.306442	15820	35261	35261	1	1	0.030809	1	0.053177	1
chr8	85787158	85787158	0.306351	0.306351	176689	200983	200983	1	1	0.030832	1	0.053214	1
chr7	1245797	1245797	0.306343	0.306343	160399	165752	165752	1	1	0.030835	1	0.053218	1
chr5	1.41E+08	1.41E+08	-0.13178	0.26355	145603	126776	126777	2	11	0.030919	1	0.077359	1

chr1	90309410	90309410	0.306021	0.306021	9385	21108	21108	1	5	0.030921	1	0.053355	1
chr16	787266	787266	-0.30595	0.305946	66698	324034	324034	1	3	0.030939	1	0.053386	1
chr1	31988382	31988382	-0.30584	0.305845	5355	12293	12293	1	3	0.030965	1	0.053427	1
chr19	39926777	39926786	0.131637	0.263275	92225	385790	385791	2	10	0.031022	1	0.077563	1
chr8	49333003	49333003	0.305523	0.305523	175214	197949	197949	1	1	0.031056	1	0.053564	1
chr19	44306947	44306947	0.30547	0.30547	92905	387458	387458	1	3	0.03107	1	0.053587	1
chr14	45722711	45722721	0.131525	0.263051	55066	299393	299394	2	12	0.031104	1	0.077735	1
chr8	47108510	47108770	-0.13149	0.262971	175073	197681	197682	2	7	0.031133	1	0.077798	1
chr6	1.7E+08	1.7E+08	-0.30518	0.30518	159770	164006	164006	1	2	0.031151	1	0.05371	1
chr14	74704714	74704714	-0.30514	0.305141	56902	303109	303109	1	2	0.031162	1	0.053728	1
chr11	55417018	55417018	-0.30513	0.305126	32518	248671	248671	1	1	0.031165	1	0.053733	1
chr3	1.94E+08	1.94E+08	0.131295	0.26259	129607	93086	93087	2	4	0.031266	1	0.078076	1
chr2	1.73E+08	1.73E+08	-0.13123	0.262452	105681	61331	61332	2	2	0.031317	1	0.078183	1
chr9	33749216	33749416	0.131184	0.262368	181182	209896	209897	2	2	0.031348	1	0.078244	1
chr6	1.53E+08	1.53E+08	0.303942	0.303942	158170	160627	160627	1	3	0.031489	1	0.054245	1
chr7	6616423	6616919	0.130988	0.261977	161613	169104	169105	2	7	0.031491	1	0.078536	1
chr3	58398729	58398729	-0.30392	0.303923	123059	80429	80429	1	1	0.031493	1	0.054252	1
chr3	8813493	8813493	-0.30387	0.30387	119382	72383	72383	1	1	0.031508	1	0.054275	1
chr6	29720425	29720425	-0.30377	0.303771	151418	140085	140085	1	29	0.031535	1	0.054317	1
chr11	888956	888956	0.30375	0.30375	28760	239847	239847	1	6	0.031541	1	0.054326	1
chr6	1.67E+08	1.67E+08	-0.13088	0.261763	159225	162724	162725	2	2	0.031574	1	0.078702	1
chr1	17023008	17023203	-0.13087	0.261738	3384	7933	7934	2	3	0.031584	1	0.078721	1
chr19	4033718	4033718	-0.30356	0.303562	88213	375310	375310	1	2	0.031593	1	0.054407	1
chr6	2876395	2876395	0.303403	0.303403	149068	134097	134097	1	2	0.031638	1	0.054476	1
chr5	1.79E+08	1.79E+08	0.303388	0.303388	148510	132822	132822	1	2	0.031642	1	0.054484	1
chr1	57037591	57037591	-0.30319	0.303187	7956	18278	18278	1	1	0.031701	1	0.054573	1
chr14	1.02E+08	1.02E+08	-0.30301	0.30301	58861	307113	307113	1	5	0.031749	1	0.054648	1
chr6	30073259	30073259	-0.30301	0.303009	151512	140697	140697	1	4	0.03175	1	0.054649	1
chr16	34659664	34660044	-0.13063	0.261257	70373	332588	332589	2	2	0.031763	1	0.079092	1
chr11	19081590	19081590	0.302941	0.302941	30862	245121	245121	1	1	0.03177	1	0.054679	1
chr11	1.17E+08	1.17E+08	0.302935	0.302935	37520	260342	260342	1	4	0.031772	1	0.054683	1
chr4	1.29E+08	1.29E+08	0.302875	0.302875	136122	107102	107102	1	1	0.031788	1	0.054709	1
chr9	8858574	8858644	-0.13056	0.261126	180806	209346	209347	2	3	0.03181	1	0.079192	1
chr17	19277143	19277143	-0.30278	0.302782	77298	348576	348576	1	1	0.031814	1	0.05475	1
chr7	1.42E+08	1.42E+08	-0.30245	0.302455	169698	185974	185974	1	4	0.031906	1	0.054893	1
chr6	31239320	31239320	0.302398	0.302398	151775	142870	142870	1	8	0.031923	1	0.054919	1
chr6	32073700	32073700	-0.30203	0.302031	152009	145470	145470	1	11	0.032025	1	0.055081	1
chr14	72490712	72490712	-0.30195	0.301951	56713	302697	302697	1	1	0.032047	1	0.055118	1
chr2	1.99E+08	1.99E+08	-0.13015	0.260297	106846	63887	63888	2	16	0.032117	1	0.079834	1
chr16	28634766	28634844	-0.13014	0.260271	69623	330660	330661	2	6	0.032126	1	0.079853	1
chr7	1.01E+08	1.01E+08	0.301668	0.301668	167180	181151	181151	1	16	0.032128	1	0.055246	1
chr8	1301014	1301014	-0.30157	0.301572	171944	191338	191338	1	1	0.032155	1	0.05529	1
chr15	59063195	59063195	0.301488	0.301488	62517	315207	315207	1	13	0.032179	1	0.055327	1
chr10	3121320	3121320	-0.30141	0.301413	19275	218851	218851	1	1	0.0322	1	0.055361	1
chr4	1.16E+08	1.16E+08	-0.30122	0.30122	135675	106232	106232	1	1	0.032255	1	0.055447	1
chr5	1.47E+08	1.47E+08	0.301199	0.301199	146051	127773	127773	1	1	0.032261	1	0.055457	1
chr7	985839	985839	0.300783	0.300783	160275	165352	165352	1	5	0.032375	1	0.05564	1
chr8	99165228	99165228	-0.30073	0.300729	177326	202201	202201	1	1	0.03239	1	0.055664	1
chr18	45456587	45456743	0.129615	0.25923	85737	369648	369649	2	4	0.032515	1	0.080672	1
chr12	1.31E+08	1.31E+08	0.129612	0.259225	48208	283900	283901	2	11	0.032518	1	0.080676	1
chr16	85242503	85242503	-0.30017	0.300166	73225	338678	338678	1	9	0.032554	1	0.055919	1
chr5	1641593	1641593	-0.29983	0.299834	139204	113577	113577	1	11	0.032649	1	0.05607	1
chr19	46389533	46389533	0.299792	0.299792	93280	388449	388449	1	8	0.032661	1	0.05609	1
chr3	1.7E+08	1.7E+08	0.299743	0.299743	128148	90310	90310	1	17	0.032674	1	0.056112	1
chr4	1.91E+08	1.91E+08	-0.2995	0.2995	138599	111974	111974	1	1	0.032747	1	0.056226	1
chr6	30431274	30431563	0.129278	0.258556	151583	141379	141380	2	34	0.032766	1	0.081198	1
chr2	75059013	75059013	0.299341	0.299341	100861	52047	52047	1	6	0.032795	1	0.0563	1
chr3	23713441	23713773	0.129235	0.258471	120456	74516	74517	2	3	0.032797	1	0.081267	1
chr11	69448444	69448444	-0.29923	0.299228	34745	254518	254518	1	1	0.032826	1	0.05635	1
chr12	11002403	11002411	0.129189	0.258378	40626	266992	266993	2	6	0.032832	1	0.081338	1
chr22	29707585	29707782	-0.12917	0.258341	116988	409620	409621	2	3	0.032846	1	0.081366	1
chr4	99700350	99700350	-0.29905	0.299055	135027	104873	104873	1	1	0.032875	1	0.056426	1
chr2	2.33E+08	2.33E+08	0.299	0.299	109043	68324	68324	1	2	0.032891	1	0.056453	1
chr12	1.25E+08	1.25E+08	0.29897	0.29897	47785	282984	282984	1	3	0.0329	1	0.056469	1
chr6	32633157	32633163	-0.12909	0.258181	152161	146446	146447	2	10	0.032907	1	0.081495	1
chr7	1198977	1198977	0.298856	0.298856	160386	165719	165719	1	4	0.032932	1	0.056518	1
chr2	1.29E+08	1.29E+08	-0.12904	0.258083	103627	57621	57622	2	15	0.032943	1	0.081571	1
chr11	76381800	76381800	-0.2987	0.2987	35615	256564	256564	1	16	0.032979	1	0.056591	1
chr10	37414506	37414597	-0.12894	0.257872	21397	223074	223075	2	12	0.033025	1	0.081738	1
chr19	38794814	38794845	-0.12893	0.257864	92000	385229	385230	2	5	0.033027	1	0.081744	1
chr8	33342441	33342441	-0.2985	0.298504	174407	196287	196287	1	14	0.033039	1	0.056685	1
chr9	98637884	98638211	-0.1289	0.257798	182677	212223	212224	2	7	0.033053	1	0.081797	1
chr11	1643842	1643842	-0.29845	0.298448	29021	240618	240618	1	3	0.033056	1	0.056711	1
chr8	19333253	19333253	0.298447	0.298447	173326	194033	194033	1	1	0.033056	1	0.056711	1

chr1	1.69E+08	1.69E+08	0.128895	0.257789	13548	30777	30778	2	3	0.033056	1	0.081805	1
chr1	37271822	37271960	-0.12885	0.25771	5991	13796	13797	2	3	0.033087	1	0.081867	1
chr6	28945338	28945341	0.128807	0.257614	151278	139344	139345	2	4	0.033122	1	0.08194	1
chr6	31381779	31382065	0.128774	0.257548	151793	142980	142981	2	6	0.033148	1	0.081995	1
chr2	11734257	11734257	0.29809	0.29809	96849	43850	43850	1	1	0.033164	1	0.056882	1
chr20	52821599	52821599	-0.29807	0.298065	113160	400993	400993	1	1	0.033171	1	0.056892	1
chr3	43465503	43465503	0.298033	0.298033	121485	76602	76602	1	1	0.03318	1	0.056909	1
chr11	1.19E+08	1.19E+08	0.128625	0.257249	37820	261102	261103	2	3	0.033257	1	0.082232	1
chr7	63506148	63506261	-0.12861	0.257219	164799	175666	175667	2	8	0.033269	1	0.082256	1
chr1	25069938	25069938	0.297555	0.297555	4495	10246	10246	1	2	0.033326	1	0.05714	1
chr11	1.34E+08	1.34E+08	0.297501	0.297501	39313	264073	264073	1	5	0.033342	1	0.057166	1
chr2	10183140	10183227	-0.1285	0.257	96650	43401	43402	2	10	0.03335	1	0.08243	1
chr13	23458333	23458333	0.297435	0.297435	49241	286534	286534	1	1	0.033362	1	0.057197	1
chr15	69325340	69325449	0.12838	0.256761	63550	317258	317259	2	3	0.033442	1	0.082631	1
chr13	1.11E+08	1.11E+08	0.297136	0.297136	52832	293721	293721	1	1	0.033451	1	0.057338	1
chr12	95897447	95897447	-0.29705	0.297051	45192	276995	276995	1	1	0.033477	1	0.05738	1
chr2	863938	863946	0.128221	0.256442	95780	41620	41621	2	3	0.033562	1	0.082888	1
chr10	51549499	51549499	0.296717	0.296717	22130	224599	224599	1	4	0.033578	1	0.057536	1
chr10	1.3E+08	1.3E+08	-0.12816	0.256313	27369	235619	235620	2	4	0.033613	1	0.082993	1
chr6	1.53E+08	1.53E+08	-0.29659	0.296589	158188	160672	160672	1	1	0.033616	1	0.057598	1
chr1	38279984	38279984	-0.29642	0.296422	6107	14079	14079	1	1	0.033667	1	0.057677	1
chr5	1.41E+08	1.41E+08	0.296392	0.296392	145773	127268	127268	1	1	0.033677	1	0.057692	1
chr5	25190672	25190672	0.296241	0.296241	140346	115899	115899	1	4	0.033721	1	0.057763	1
chr4	1.87E+08	1.87E+08	-0.12802	0.256036	138339	111482	111483	2	8	0.033722	1	0.083225	1
chr2	2.4E+08	2.4E+08	-0.29595	0.295952	109929	70172	70172	1	2	0.033807	1	0.057901	1
chr3	1.96E+08	1.96E+08	0.295886	0.295886	129897	93676	93676	1	3	0.033827	1	0.057932	1
chr6	1449399	1449399	0.295813	0.295813	148921	133786	133786	1	1	0.033849	1	0.057968	1
chr7	1.28E+08	1.28E+08	0.295767	0.295767	168487	183585	183585	1	2	0.033864	1	0.057991	1
chr8	8580928	8580960	0.127791	0.255581	172588	192632	192633	2	7	0.033893	1	0.083596	1
chr1	1.7E+08	1.7E+08	0.295666	0.295666	13563	30813	30813	1	1	0.033896	1	0.05804	1
chr14	93064986	93064986	0.295586	0.295586	57980	305221	305221	1	1	0.033922	1	0.058081	1
chr9	74061171	74061323	0.127716	0.255432	181812	210896	210897	2	4	0.033948	1	0.083717	1
chr3	44450272	44450272	-0.29546	0.295461	121551	76750	76750	1	1	0.033964	1	0.058146	1
chr15	94147555	94147555	0.295452	0.295452	65808	321954	321954	1	1	0.033967	1	0.058149	1
chr1	7432614	7432614	0.295341	0.295341	1904	5047	5047	1	2	0.034	1	0.058204	1
chr7	1.31E+08	1.31E+08	0.12761	0.255219	168859	184466	184467	2	2	0.03403	1	0.083891	1
chr13	1.12E+08	1.12E+08	-0.29515	0.295152	52992	294105	294105	1	1	0.034058	1	0.058293	1
chr16	90144006	90144006	-0.29511	0.295115	74732	342311	342311	1	5	0.034068	1	0.058311	1
chr1	42248998	42248998	-0.29504	0.295036	6567	15110	15110	1	1	0.034092	1	0.058349	1
chr1	40420675	40420689	0.127526	0.255053	6348	14637	14638	2	16	0.034094	1	0.084031	1
chr10	11343618	11344086	-0.12753	0.255052	19876	220108	220109	2	6	0.034094	1	0.084032	1
chr8	25107121	25107121	0.294963	0.294963	173860	195162	195162	1	1	0.034113	1	0.058383	1
chr7	1233469	1233637	0.127437	0.254874	160396	165745	165746	2	3	0.034162	1	0.084182	1
chr5	346695	346987	-0.1274	0.254801	138736	112278	112279	2	3	0.034189	1	0.084242	1
chr2	1.67E+08	1.67E+08	-0.29444	0.29444	105339	60699	60699	1	2	0.034275	1	0.058636	1
chr22	24381773	24381773	-0.29442	0.294416	116686	408910	408910	1	1	0.034282	1	0.058648	1
chr8	1.04E+08	1.04E+08	-0.12726	0.254526	177677	202903	202904	2	13	0.034296	1	0.084468	1
chr1	33608053	33608053	-0.29425	0.294251	5618	12945	12945	1	1	0.034335	1	0.058728	1
chr10	34151199	34151199	-0.29414	0.294144	21205	222785	222785	1	2	0.034366	1	0.058779	1
chr16	5007834	5007972	0.127168	0.254335	68101	327769	327770	2	9	0.034372	1	0.084629	1
chr21	46075092	46075092	0.294103	0.294103	115505	406388	406388	1	4	0.034379	1	0.0588	1
chr7	90896572	90896701	0.127148	0.254297	166240	178550	178551	2	21	0.034386	1	0.084662	1
chr20	61878356	61878356	0.294065	0.294065	113864	402748	402748	1	2	0.034392	1	0.05882	1
chr11	60161999	60161999	-0.29406	0.294059	32942	249510	249510	1	2	0.034395	1	0.058823	1
chr15	74345103	74345103	0.294054	0.294054	64035	318120	318120	1	2	0.034396	1	0.058825	1
chr1	23280069	23280477	-0.12711	0.254227	4285	9703	9704	2	6	0.03441	1	0.084718	1
chr19	1792217	1792217	-0.29366	0.293664	87493	373623	373623	1	1	0.03452	1	0.059022	1
chr17	16593582	16593589	-0.12691	0.253814	76912	347773	347774	2	6	0.034572	1	0.085072	1
chr11	1.11E+08	1.11E+08	-0.29345	0.293454	37092	259396	259396	1	1	0.034586	1	0.059127	1
chr13	1.14E+08	1.14E+08	0.126884	0.253767	53452	295370	295371	2	7	0.03459	1	0.085112	1
chr1	92203667	92203667	0.293396	0.293396	9492	21371	21371	1	1	0.034605	1	0.059156	1
chr1	62499140	62499140	0.293159	0.293159	8239	18785	18785	1	1	0.03468	1	0.059274	1
chr6	31529897	31529897	-0.29303	0.293032	151822	143250	143250	1	2	0.03472	1	0.059335	1
chr2	11680057	11680086	-0.12668	0.253358	96837	43830	43831	2	8	0.03475	1	0.085455	1
chr12	1.09E+08	1.09E+08	0.126673	0.253347	45940	278550	278551	2	12	0.034754	1	0.085463	1
chr1	2.01E+08	2.01E+08	0.292864	0.292864	14927	33407	33407	1	1	0.034771	1	0.059418	1
chr7	1.31E+08	1.31E+08	0.292854	0.292854	168919	184568	184568	1	1	0.034775	1	0.059423	1
chr19	39523840	39523840	-0.29284	0.292845	92154	385604	385604	1	7	0.034778	1	0.059428	1
chr17	45813433	45813433	0.292838	0.292838	80197	355649	355649	1	1	0.03478	1	0.05943	1
chr15	1.01E+08	1.01E+08	0.292765	0.292765	66183	322735	322735	1	1	0.034803	1	0.059468	1
chr7	1.12E+08	1.12E+08	-0.29275	0.29275	167887	182446	182446	1	6	0.034807	1	0.059476	1
chr6	1.7E+08	1.7E+08	-0.29248	0.292476	159704	163861	163861	1	2	0.034894	1	0.059614	1
chr11	66108893	66108893	0.292364	0.292364	34084	252809	252809	1	3	0.034929	1	0.059671	1
chr9	44420179	44420179	-0.2923	0.292301	181606	210622	210622	1	1	0.03495	1	0.059703	1

chr10	1.35E+08	1.35E+08	-0.12634	0.252682	28403	238811	238812	2	3	0.035014	1	0.086028	1
chr9	1.4E+08	1.4E+08	-0.29191	0.291905	185639	216828	216828	1	1	0.035073	1	0.059902	1
chr16	46602737	46603015	-0.12623	0.252459	70400	332641	332642	2	7	0.035101	1	0.086218	1
chr2	71723690	71723690	0.291812	0.291812	100519	51198	51198	1	1	0.035103	1	0.059952	1
chr19	49573012	49573012	0.291492	0.291492	93882	389896	389896	1	1	0.035206	1	0.060118	1
chr19	35645620	35646039	0.126039	0.252078	91584	384054	384055	2	6	0.035249	1	0.086547	1
chr16	9185470	9185479	0.125972	0.251943	68300	328141	328142	2	11	0.035303	1	0.086663	1
chr17	37123669	37123671	0.125951	0.251901	78791	352071	352072	2	9	0.035321	1	0.086701	1
chr21	30391784	30391795	-0.12594	0.251873	114428	403972	403973	2	8	0.035332	1	0.086726	1
chr6	32629955	32629955	0.291063	0.291063	152159	146437	146437	1	1	0.035345	1	0.060339	1
chr12	1.1E+08	1.1E+08	-0.29094	0.290941	46157	279038	279038	1	6	0.035385	1	0.0604	1
chr14	1.01E+08	1.01E+08	0.125835	0.25167	58665	306577	306578	2	2	0.035409	1	0.086899	1
chr11	62187186	62187186	-0.29081	0.290807	33231	250207	250207	1	1	0.035426	1	0.060468	1
chr1	75590483	75590912	-0.12573	0.251467	8781	19889	19890	2	5	0.035492	1	0.087079	1
chr6	29635507	29635507	-0.29059	0.290586	151389	139920	139920	1	8	0.035496	1	0.06058	1
chr7	24323675	24323764	-0.12568	0.251367	162353	170608	170609	2	11	0.035534	1	0.087166	1
chr2	46890238	46890238	-0.29044	0.290444	99111	48325	48325	1	1	0.035544	1	0.060655	1
chr10	1.03E+08	1.03E+08	-0.12567	0.251334	25375	231248	231249	2	5	0.035547	1	0.087196	1
chr11	57159174	57159174	-0.29043	0.290427	32646	248872	248872	1	1	0.035549	1	0.060664	1
chr16	27741555	27741555	-0.2903	0.290298	69532	330476	330476	1	1	0.035592	1	0.060731	1
chr10	1.11E+08	1.11E+08	-0.29029	0.290292	25940	232679	232679	1	1	0.035594	1	0.060733	1
chr13	21893289	21893420	0.125554	0.251108	49149	286360	286361	2	4	0.035634	1	0.087396	1
chr6	43200203	43200203	0.290151	0.290151	153532	151288	151288	1	1	0.035641	1	0.060806	1
chr6	26022062	26022062	-0.29011	0.290112	150779	137346	137346	1	7	0.035653	1	0.060825	1
chr16	90016004	90016020	0.125454	0.250908	74694	342215	342216	2	8	0.035711	1	0.08757	1
chr16	89593170	89593170	-0.28982	0.289816	74542	341829	341829	1	4	0.03575	1	0.060978	1
chr6	1.68E+08	1.68E+08	-0.28979	0.289786	159315	162933	162933	1	1	0.035761	1	0.060994	1
chr1	2265881	2265881	0.289765	0.289765	750	2116	2116	1	8	0.035767	1	0.061005	1
chr2	91758369	91758369	-0.28972	0.289719	101481	53329	53329	1	1	0.035783	1	0.061031	1
chr5	1.42E+08	1.42E+08	-0.28966	0.28966	145839	127392	127392	1	1	0.035803	1	0.061063	1
chr17	40837037	40837037	-0.28958	0.289582	79477	353821	353821	1	3	0.035829	1	0.061103	1
chr8	1.45E+08	1.45E+08	0.125263	0.250527	180230	207955	207956	2	2	0.035855	1	0.087902	1
chr16	1272275	1272275	0.289021	0.289021	66967	324804	324804	1	8	0.036016	1	0.061399	1
chr8	1.45E+08	1.45E+08	0.288917	0.288917	180216	207923	207923	1	2	0.03605	1	0.061453	1
chr7	55980652	55980652	-0.28891	0.288912	164556	175174	175174	1	5	0.036052	1	0.061456	1
chr5	28810402	28810645	-0.12499	0.249978	140385	115962	115963	2	4	0.036066	1	0.088385	1
chr14	1.04E+08	1.04E+08	-0.28886	0.288861	59183	307947	307947	1	2	0.03607	1	0.061484	1
chr11	1.33E+08	1.33E+08	0.288658	0.288658	39180	263753	263753	1	1	0.036137	1	0.061591	1
chr6	1.17E+08	1.17E+08	-0.28859	0.288595	156365	157098	157098	1	2	0.036157	1	0.061625	1
chr6	1.5E+08	1.5E+08	0.124826	0.249651	157996	160238	160239	2	11	0.036196	1	0.088672	1
chr1	1.66E+08	1.66E+08	-0.12478	0.249563	13313	30247	30248	2	6	0.036231	1	0.088753	1
chr9	1.39E+08	1.39E+08	0.124762	0.249524	185373	216404	216405	2	3	0.036246	1	0.088787	1
chr12	1.32E+08	1.32E+08	0.124697	0.249395	48410	284327	284328	2	5	0.036294	1	0.0889	1
chr20	44574847	44575021	-0.12468	0.249356	112744	400019	400020	2	2	0.036308	1	0.088933	1
chr14	69261160	69261160	0.288027	0.288027	56418	302142	302142	1	14	0.036349	1	0.061929	1
chr5	25190850	25191097	-0.12462	0.249249	140346	115900	115901	2	4	0.03635	1	0.089025	1
chr17	80436296	80436296	0.287994	0.287994	84373	366177	366177	1	2	0.03636	1	0.061946	1
chr19	11071743	11071746	-0.12459	0.249184	89437	378414	378415	2	4	0.036376	1	0.089083	1
chr11	93754223	93754223	-0.28777	0.28777	36384	258007	258007	1	6	0.036435	1	0.062067	1
chr11	1.18E+08	1.18E+08	-0.28776	0.287764	37635	260581	260581	1	1	0.036437	1	0.06207	1
chr11	1.18E+08	1.18E+08	0.124486	0.248972	37726	260827	260828	2	10	0.036457	1	0.08927	1
chr10	1.31E+08	1.31E+08	0.28764	0.28764	27523	236003	236003	1	3	0.036479	1	0.062136	1
chr19	17393354	17393744	0.124449	0.248899	90454	381160	381161	2	11	0.036487	1	0.089337	1
chr3	10277547	10277547	0.287519	0.287519	119564	72832	72832	1	1	0.03652	1	0.062203	1
chr2	2.06E+08	2.06E+08	-0.28734	0.287339	107280	64774	64774	1	1	0.036582	1	0.062297	1
chr9	1.14E+08	1.14E+08	-0.28724	0.287244	183226	213091	213091	1	1	0.036613	1	0.062347	1
chr12	48598048	48598048	0.287179	0.287179	42267	270263	270263	1	2	0.036634	1	0.062381	1
chr2	1.09E+08	1.09E+08	0.287139	0.287139	102548	55503	55503	1	1	0.036649	1	0.062402	1
chr6	29718093	29718093	-0.28712	0.287115	151416	140076	140076	1	34	0.036657	1	0.062416	1
chr2	26628105	26628105	0.287115	0.287115	97703	45509	45509	1	1	0.036658	1	0.062417	1
chr1	2.33E+08	2.33E+08	-0.28679	0.286785	17714	39107	39107	1	1	0.036772	1	0.062594	1
chr8	1950112	1950120	0.124076	0.248153	172179	191939	191940	2	2	0.036784	1	0.090013	1
chr17	9677572	9677572	-0.28666	0.286664	76427	346808	346808	1	1	0.03681	1	0.062659	1
chr9	1.39E+08	1.39E+08	-0.28653	0.286526	185543	216683	216683	1	1	0.036857	1	0.062734	1
chr11	1.34E+08	1.34E+08	0.286447	0.286447	39217	263819	263819	1	1	0.036882	1	0.062776	1
chr4	1.2E+08	1.2E+08	0.286161	0.286161	135749	106370	106370	1	12	0.03698	1	0.062928	1
chr17	76421442	76421442	0.286064	0.286064	83192	362711	362711	1	5	0.037014	1	0.062981	1
chr4	1.03E+08	1.03E+08	-0.28575	0.285748	135176	105144	105144	1	1	0.037123	1	0.063152	1
chr8	81806219	81806286	0.123628	0.247255	176587	200795	200796	2	4	0.037146	1	0.090836	1
chr7	1.39E+08	1.39E+08	0.285667	0.285667	169415	185423	185423	1	1	0.037151	1	0.063197	1
chr6	32193107	32193235	-0.12353	0.24707	152055	146191	146192	2	2	0.037224	1	0.091012	1
chr21	44513500	44513500	-0.2853	0.2853	115238	405825	405825	1	1	0.03728	1	0.063402	1
chr13	79694063	79694063	-0.28513	0.285127	51790	291691	291691	1	1	0.037339	1	0.063496	1
chr5	1.41E+08	1.41E+08	-0.12338	0.246762	145617	126850	126851	2	12	0.037345	1	0.091295	1

chr10	1.35E+08	1.35E+08	0.284893	0.284893	28055	237613	237613	1	5	0.037421	1	0.063625	1
chr3	1.58E+08	1.58E+08	0.123176	0.246353	127794	89574	89575	2	9	0.037507	1	0.091677	1
chr12	1.22E+08	1.22E+08	0.123153	0.246306	47252	281786	281787	2	8	0.037526	1	0.091721	1
chr12	1.33E+08	1.33E+08	0.284554	0.284554	48854	285646	285646	1	3	0.037541	1	0.063814	1
chr6	32844032	32844032	0.284522	0.284522	152206	146822	146822	1	1	0.037552	1	0.063831	1
chr5	1.41E+08	1.41E+08	-0.12307	0.246132	145588	126704	126705	2	13	0.037596	1	0.091884	1
chr7	76221076	76221076	-0.28438	0.284377	165742	177577	177577	1	1	0.037606	1	0.063915	1
chr12	54073597	54073597	-0.28437	0.28437	43077	272296	272296	1	1	0.037608	1	0.063918	1
chr13	25872067	25872067	0.284284	0.284284	49471	287014	287014	1	1	0.037638	1	0.063965	1
chr12	1.14E+08	1.14E+08	0.284223	0.284223	46448	279732	279732	1	5	0.037659	1	0.063999	1
chr1	1.6E+08	1.6E+08	-0.28416	0.284164	12831	29221	29221	1	5	0.037678	1	0.06403	1
chr17	14198368	14198392	-0.12295	0.245893	76702	347318	347319	2	2	0.03769	1	0.092106	1
chr1	44435428	44435433	-0.12281	0.245617	6852	15776	15777	2	14	0.0378	1	0.092365	1
chr6	1.09E+08	1.09E+08	0.283801	0.283801	155995	156379	156379	1	3	0.037805	1	0.064236	1
chr1	1.56E+08	1.56E+08	-0.28357	0.283575	12376	28222	28222	1	6	0.037886	1	0.064364	1
chr11	19790796	19790796	-0.28352	0.28352	30918	245247	245247	1	1	0.037905	1	0.064395	1
chr18	45660777	45660777	-0.28347	0.283473	85751	369681	369681	1	1	0.037923	1	0.064423	1
chr8	1.01E+08	1.01E+08	-0.12265	0.2453	177429	202436	202437	2	5	0.037926	1	0.092662	1
chr14	1.07E+08	1.07E+08	0.283456	0.283456	59907	309646	309646	1	3	0.037929	1	0.064434	1
chr1	2.41E+08	2.41E+08	0.283328	0.283328	18137	39945	39945	1	2	0.037974	1	0.064505	1
chr7	1948947	1948947	0.28321	0.28321	160671	166604	166604	1	13	0.038017	1	0.064572	1
chr15	1.02E+08	1.02E+08	0.283147	0.283147	66367	323108	323108	1	1	0.03804	1	0.064609	1
chr14	1.06E+08	1.06E+08	-0.28314	0.283141	59728	309243	309243	1	5	0.038042	1	0.064612	1
chr7	35078082	35078097	0.122437	0.244875	163183	172543	172544	2	12	0.038096	1	0.093071	1
chr1	6341230	6341287	0.122407	0.244815	1712	4594	4595	2	4	0.038122	1	0.093127	1
chr1	912055	912055	0.282762	0.282762	88	222	222	1	3	0.038179	1	0.064832	1
chr1	27902069	27902555	0.122229	0.244458	4957	11425	11426	2	6	0.038264	1	0.093466	1
chr21	45246164	45246164	-0.28246	0.282464	115349	406067	406067	1	5	0.038284	1	0.065003	1
chr6	29716554	29716597	0.122179	0.244358	151416	140050	140051	2	34	0.038305	1	0.093565	1
chr17	78912765	78912765	-0.28238	0.28238	83798	364327	364327	1	2	0.038315	1	0.065053	1
chr7	1.49E+08	1.49E+08	-0.28213	0.282131	170016	186600	186600	1	1	0.038403	1	0.065194	1
chr12	1.21E+08	1.21E+08	-0.12201	0.24403	47017	281120	281121	2	8	0.038438	1	0.093877	1
chr19	7688798	7688798	0.282023	0.282023	88907	376968	376968	1	3	0.038444	1	0.065258	1
chr17	77979405	77979890	0.12194	0.243879	83529	363610	363611	2	3	0.038499	1	0.094023	1
chr16	57836395	57836397	0.121913	0.243826	71242	334456	334457	2	8	0.038523	1	0.094076	1
chr20	30584710	30584710	-0.28163	0.281631	111837	397561	397561	1	3	0.038588	1	0.065488	1
chr2	2.19E+08	2.19E+08	-0.28156	0.281565	108051	66165	66165	1	1	0.038613	1	0.065528	1
chr2	2.38E+08	2.38E+08	0.281479	0.281479	109660	69573	69573	1	1	0.038642	1	0.065575	1
chr5	373299	373299	0.281459	0.281459	138744	112294	112294	1	2	0.03865	1	0.065587	1
chr4	48175353	48175353	0.281457	0.281457	133202	101271	101271	1	1	0.038651	1	0.065588	1
chr17	7320581	7320581	-0.28146	0.281457	76024	345652	345652	1	4	0.038651	1	0.065588	1
chr22	42896249	42896249	-0.2814	0.281396	118176	412504	412504	1	10	0.038674	1	0.065623	1
chr12	89920650	89920650	0.281306	0.281306	44885	276476	276476	1	1	0.038708	1	0.065676	1
chr17	74679597	74679597	0.28129	0.28129	82889	361990	361990	1	1	0.038713	1	0.065684	1
chr2	2.41E+08	2.41E+08	-0.28118	0.281185	110088	70550	70550	1	1	0.038752	1	0.065746	1
chr5	843766	844184	0.121599	0.243197	138900	112706	112707	2	3	0.038778	1	0.094691	1
chr5	1.54E+08	1.54E+08	0.281099	0.281099	146572	128800	128800	1	1	0.038784	1	0.065798	1
chr14	71374784	71374784	-0.28109	0.281094	56644	302607	302607	1	8	0.038785	1	0.0658	1
chr14	23352295	23352488	0.121463	0.242926	54206	297265	297266	2	6	0.038885	1	0.094954	1
chr11	1.35E+08	1.35E+08	-0.28079	0.280793	39374	264204	264204	1	2	0.038892	1	0.065972	1
chr14	1.03E+08	1.03E+08	-0.28073	0.280732	59122	307795	307795	1	5	0.038913	1	0.066006	1
chr8	1651128	1651128	-0.28063	0.280628	172068	191649	191649	1	2	0.03895	1	0.066068	1
chr22	20192371	20192485	0.121266	0.242531	116299	408042	408043	2	2	0.039045	1	0.095338	1
chr5	669397	669733	0.121237	0.242474	138870	112642	112643	2	4	0.039068	1	0.095396	1
chr20	60628389	60628389	0.280292	0.280292	113560	402017	402017	1	1	0.039075	1	0.066265	1
chr13	1.13E+08	1.13E+08	0.121228	0.242455	53233	294748	294749	2	14	0.039077	1	0.095416	1
chr6	30038712	30038712	-0.28027	0.28027	151504	140565	140565	1	43	0.039084	1	0.066278	1
chr1	65886182	65886266	0.121208	0.242416	8478	19267	19268	2	12	0.039093	1	0.095456	1
chr10	47062881	47062881	0.280207	0.280207	21855	224055	224055	1	1	0.039105	1	0.066312	1
chr5	1.34E+08	1.34E+08	0.121162	0.242323	144817	124719	124720	2	5	0.039129	1	0.095543	1
chr7	5812747	5812747	-0.28014	0.280135	161473	168801	168801	1	1	0.039132	1	0.066355	1
chr11	78470286	78470286	0.279883	0.279883	35829	256982	256982	1	1	0.039233	1	0.066512	1
chr19	5478473	5478484	0.120964	0.241928	88559	376100	376101	2	2	0.039286	1	0.095925	1
chr17	1835482	1835482	0.279469	0.279469	75212	343452	343452	1	2	0.039387	1	0.066759	1
chr2	73215647	73215647	0.279455	0.279455	100613	51386	51386	1	3	0.039392	1	0.066767	1
chr11	57283055	57283055	-0.2794	0.279399	32679	248929	248929	1	12	0.039413	1	0.0668	1
chr8	6419483	6419570	-0.12079	0.241577	172351	192257	192258	2	4	0.039428	1	0.096266	1
chr12	13041108	13041108	-0.27936	0.279357	40799	267316	267316	1	1	0.039428	1	0.066823	1
chr6	30071572	30071601	0.120732	0.241464	151511	140691	140692	2	53	0.039473	1	0.096378	1
chr15	22442000	22442048	-0.12073	0.24146	60022	309816	309817	2	2	0.039474	1	0.096382	1
chr19	19639596	19639596	-0.27918	0.279182	90968	382491	382491	1	6	0.039491	1	0.066927	1
chr8	1.43E+08	1.43E+08	0.120705	0.241409	179658	206447	206448	2	2	0.039494	1	0.096432	1
chr21	47604986	47605072	-0.1207	0.241393	115826	407005	407006	2	3	0.039499	1	0.096448	1
chr7	86782435	86782435	0.279129	0.279129	166094	178174	178174	1	16	0.039511	1	0.066958	1

chr7	563891	563891	0.279008	0.279008	160080	164799	164799	1	5	0.039556	1	0.067028	1
chr20	61455419	61455419	0.278892	0.278892	113745	402478	402478	1	5	0.039601	1	0.067099	1
chr20	29534074	29534074	0.278878	0.278878	111731	397279	397279	1	1	0.039605	1	0.067106	1
chr5	37723683	37723683	0.278761	0.278761	140777	116707	116707	1	1	0.039648	1	0.067177	1
chr9	71627524	71627524	-0.27862	0.278616	181719	210760	210760	1	2	0.039704	1	0.067265	1
chr2	1.79E+08	1.79E+08	0.278571	0.278571	106154	62572	62572	1	1	0.039721	1	0.067293	1
chr16	32858241	32858241	-0.27841	0.278407	70282	332422	332422	1	6	0.039781	1	0.067388	1
chr2	2.37E+08	2.37E+08	-0.27838	0.278385	109428	69109	69109	1	2	0.039789	1	0.067403	1
chr10	1.3E+08	1.3E+08	-0.1203	0.240592	27407	235698	235699	2	2	0.039813	1	0.097251	1
chr12	98117821	98117821	-0.27829	0.278288	45331	277252	277252	1	1	0.039826	1	0.067461	1
chr15	93286499	93286499	0.278067	0.278067	65729	321784	321784	1	1	0.03991	1	0.067594	1
chr4	675827	675936	0.120175	0.24035	130219	94482	94483	2	2	0.039913	1	0.097502	1
chr7	1097952	1097952	0.278057	0.278057	160335	165562	165562	1	9	0.039914	1	0.0676	1
chr7	1961968	1962212	0.120151	0.240302	160678	166631	166632	2	3	0.039932	1	0.097552	1
chr4	1580172	1580193	0.120132	0.240264	130592	95629	95630	2	8	0.039949	1	0.09759	1
chr19	49168736	49168736	-0.27785	0.277852	93766	389611	389611	1	1	0.039991	1	0.067724	1
chr2	1.99E+08	1.99E+08	0.277839	0.277839	106841	63870	63870	1	12	0.039996	1	0.067732	1
chr19	52456033	52456033	-0.2778	0.277798	94481	391430	391430	1	1	0.040011	1	0.067756	1
chr9	72132995	72132995	0.277649	0.277649	181764	210824	210824	1	1	0.040068	1	0.067848	1
chr6	29856938	29856938	-0.27763	0.277633	151437	140229	140229	1	35	0.040074	1	0.067857	1
chr18	77377538	77377589	-0.11993	0.239867	86691	371770	371771	2	5	0.040106	1	0.097994	1
chr5	1.21E+08	1.21E+08	-0.11991	0.239817	144112	123298	123299	2	23	0.040125	1	0.098043	1
chr2	2.39E+08	2.39E+08	0.119895	0.239789	109721	69698	69699	2	4	0.040137	1	0.098071	1
chr11	1.17E+08	1.17E+08	-0.27745	0.27745	37505	260286	260286	1	1	0.040147	1	0.067971	1
chr14	1.04E+08	1.04E+08	0.277444	0.277444	59163	307895	307895	1	1	0.040149	1	0.067975	1
chr16	88267466	88267466	-0.27741	0.277415	74002	340458	340458	1	6	0.04016	1	0.067993	1
chr5	34929231	34929231	0.277399	0.277399	140666	116446	116446	1	10	0.040166	1	0.068001	1
chr1	84543374	84543374	-0.27738	0.277379	9046	20405	20405	1	10	0.040173	1	0.068013	1
chr11	47236405	47236405	-0.27734	0.277339	32266	248176	248176	1	10	0.040189	1	0.068039	1
chr19	56652683	56653147	-0.11982	0.239648	95225	393341	393342	2	10	0.040193	1	0.098217	1
chr3	1.81E+08	1.81E+08	-0.11968	0.239364	128681	91206	91207	2	8	0.040306	1	0.098503	1
chr16	73102243	73102243	-0.27694	0.276945	72383	337037	337037	1	1	0.040338	1	0.068275	1
chr20	62387416	62387416	-0.27684	0.276845	114062	403182	403182	1	1	0.040379	1	0.06834	1
chr5	80493	80493	0.276609	0.276609	138653	112067	112067	1	3	0.04047	1	0.068486	1
chr15	90030046	90030153	0.119463	0.238927	65406	321067	321068	2	5	0.040474	1	0.098946	1
chr7	95181501	95181501	0.276566	0.276566	166473	179200	179200	1	1	0.040488	1	0.068513	1
chr7	1.58E+08	1.58E+08	-0.27653	0.276532	171362	190002	190002	1	7	0.040501	1	0.068535	1
chr10	1.31E+08	1.31E+08	-0.11942	0.238843	27472	235853	235854	2	3	0.040506	1	0.09903	1
chr6	25279718	25279741	-0.11942	0.23884	150711	137199	137200	2	3	0.040507	1	0.099032	1
chr2	1.19E+08	1.19E+08	-0.1194	0.238793	103023	56370	56371	2	7	0.040527	1	0.099083	1
chr3	1.95E+08	1.95E+08	0.2763	0.2763	129781	93438	93438	1	1	0.040596	1	0.068682	1
chr16	84693148	84693148	0.276245	0.276245	73092	338416	338416	1	3	0.040617	1	0.068714	1
chr5	1.76E+08	1.76E+08	-0.27623	0.27623	147990	131637	131637	1	1	0.040623	1	0.068724	1
chr6	31276504	31276504	0.276224	0.276224	151779	142889	142889	1	17	0.040626	1	0.068729	1
chr22	30792916	30792924	0.11924	0.238481	117090	409889	409890	2	8	0.040653	1	0.0994	1
chr5	73980966	73980968	0.119195	0.23839	142277	119721	119722	2	13	0.040688	1	0.099492	1
chr22	19141030	19141030	-0.27604	0.276038	116144	407674	407674	1	1	0.040702	1	0.068846	1
chr6	1.7E+08	1.7E+08	0.275991	0.275991	159860	164229	164229	1	4	0.04072	1	0.068876	1
chr2	2.41E+08	2.41E+08	-0.27599	0.27599	110161	70717	70717	1	1	0.040721	1	0.068877	1
chr17	77773337	77773554	-0.11915	0.238293	83465	363406	363407	2	11	0.040726	1	0.099594	1
chr4	1.33E+08	1.33E+08	-0.27576	0.275755	136188	107211	107211	1	1	0.040817	1	0.069024	1
chr17	33307476	33307483	-0.11901	0.238027	78334	350954	350955	2	9	0.040834	1	0.099871	1
chr17	25289961	25290204	-0.11898	0.237954	77606	349272	349273	2	4	0.040863	1	0.099946	1
chr2	2.4E+08	2.4E+08	-0.27549	0.275494	110027	70399	70399	1	1	0.040923	1	0.069189	1
chr20	61979272	61979433	-0.11888	0.237768	113903	402836	402837	2	3	0.040939	1	0.100138	1
chr8	1797886	1797886	0.275397	0.275397	172116	191763	191763	1	3	0.04096	1	0.069247	1
chr3	31494586	31494586	0.275384	0.275384	120749	75084	75084	1	9	0.040965	1	0.069255	1
chr5	35015682	35015682	-0.27538	0.275382	140672	116461	116461	1	1	0.040966	1	0.069256	1
chr8	1.44E+08	1.44E+08	-0.27516	0.275165	179805	206794	206794	1	7	0.041056	1	0.069399	1
chr3	1.94E+08	1.94E+08	0.118712	0.237424	129612	93096	93097	2	6	0.041074	1	0.100494	1
chr11	71189514	71189514	0.274985	0.274985	35018	255266	255266	1	2	0.041129	1	0.069513	1
chr17	71810497	71810497	-0.27496	0.274965	82346	360520	360520	1	1	0.041136	1	0.069525	1
chr3	1.94E+08	1.94E+08	0.118538	0.237076	129625	93130	93131	2	7	0.041214	1	0.100857	1
chr18	77410113	77410113	-0.27474	0.274739	86707	371794	371794	1	1	0.041228	1	0.069669	1
chr2	792213	792213	0.274691	0.274691	95772	41601	41601	1	1	0.041247	1	0.069699	1
chr14	21199099	21199099	0.274689	0.274689	53966	296760	296760	1	1	0.041248	1	0.069701	1
chr19	2343629	2343629	0.274676	0.274676	87713	374174	374174	1	1	0.041253	1	0.069708	1
chr15	98196234	98196247	-0.11847	0.236946	65965	322305	322306	2	4	0.041267	1	0.100998	1
chr5	1.03E+08	1.03E+08	-0.11845	0.236909	143477	122126	122127	2	5	0.041281	1	0.101035	1
chr4	15429967	15429967	-0.27459	0.274594	132121	99123	99123	1	4	0.041285	1	0.069761	1
chr4	55143285	55143285	0.274572	0.274572	133466	101807	101807	1	2	0.041295	1	0.069776	1
chr14	39573919	39573919	-0.27453	0.274525	54958	299154	299154	1	1	0.041316	1	0.069809	1
chr5	1.47E+08	1.47E+08	-0.27451	0.274508	146103	127860	127860	1	1	0.041323	1	0.06982	1
chr12	1.11E+08	1.11E+08	0.118385	0.236769	46161	279044	279045	2	2	0.041335	1	0.101182	1

chr15	25684837	25684849	0.118383	0.236766	60299	310523	310524	2	16	0.041337	1	0.101186	1
chr1	92951594	92951596	0.118378	0.236756	9538	21514	21515	2	22	0.041339	1	0.101196	1
chr8	1.43E+08	1.43E+08	0.274466	0.274466	179707	206552	206552	1	1	0.04134	1	0.069847	1
chr11	7110074	7110083	-0.11833	0.236664	29861	243093	243094	2	5	0.041377	1	0.101291	1
chr9	1.35E+08	1.35E+08	-0.27437	0.27437	184676	215372	215372	1	1	0.041377	1	0.069908	1
chr1	1565856	1565931	-0.11832	0.236639	434	1270	1271	2	9	0.041386	1	0.101317	1
chr5	1667205	1667258	-0.11831	0.236621	139218	113612	113613	2	9	0.041392	1	0.101335	1
chr19	33719749	33719749	-0.27432	0.274325	91405	383572	383572	1	1	0.041396	1	0.06994	1
chr22	36635583	36635674	-0.11826	0.236518	117386	410616	410617	2	8	0.041436	1	0.101445	1
chr1	1.55E+08	1.55E+08	-0.11824	0.236471	12118	27469	27470	2	7	0.041453	1	0.101492	1
chr21	45759431	45759439	-0.11822	0.236439	115446	406269	406270	2	6	0.041465	1	0.101526	1
chr12	53715494	53715500	0.118144	0.236289	43016	272143	272144	2	11	0.041523	1	0.101678	1
chr11	70261959	70262058	-0.11813	0.236251	34874	254903	254904	2	2	0.041538	1	0.101716	1
chr13	19202724	19202724	-0.27394	0.27394	48913	285830	285830	1	4	0.041553	1	0.070188	1
chr7	77672208	77672208	-0.27394	0.273935	165822	177753	177753	1	1	0.041555	1	0.070191	1
chr1	65609418	65609418	-0.27391	0.27391	8450	19191	19191	1	1	0.041565	1	0.070207	1
chr9	91361323	91361323	-0.27389	0.273886	182222	211515	211515	1	1	0.041574	1	0.070222	1
chr4	1.46E+08	1.46E+08	0.118029	0.236058	136673	108122	108123	2	15	0.041612	1	0.10192	1
chr15	83654517	83654524	-0.11801	0.236025	64963	320112	320113	2	14	0.041625	1	0.101954	1
chr7	1581522	1581522	-0.27371	0.273714	160545	166211	166211	1	10	0.041642	1	0.070332	1
chr6	1.7E+08	1.7E+08	0.117988	0.235976	159852	164208	164209	2	2	0.041644	1	0.102006	1
chr3	15377670	15377670	0.273597	0.273597	120138	73987	73987	1	1	0.041688	1	0.070404	1
chr12	31405369	31405369	0.273546	0.273546	41608	268900	268900	1	2	0.041709	1	0.070435	1
chr5	6378806	6378806	-0.27348	0.273477	139627	114658	114658	1	8	0.041739	1	0.070482	1
chr10	37414768	37414802	-0.11787	0.235733	21397	223079	223080	2	12	0.04174	1	0.102265	1
chr7	69858080	69858080	-0.27337	0.273372	165134	176337	176337	1	1	0.041785	1	0.070554	1
chr9	1.17E+08	1.17E+08	-0.27336	0.273359	183392	213356	213356	1	1	0.04179	1	0.070562	1
chr19	835147	835345	0.117732	0.235463	87081	372599	372600	2	2	0.041844	1	0.10255	1
chr20	55665224	55665224	-0.27314	0.273137	113223	401171	401171	1	1	0.041882	1	0.07071	1
chr19	58566643	58566643	0.273103	0.273103	95497	394145	394145	1	1	0.041895	1	0.070732	1
chr1	17004764	17004764	0.273025	0.273025	3379	7922	7922	1	4	0.04193	1	0.070784	1
chr10	1.06E+08	1.06E+08	0.273024	0.273024	25868	232579	232579	1	4	0.041931	1	0.070785	1
chr12	93859987	93859987	-0.27301	0.273006	45048	276709	276709	1	1	0.041937	1	0.070796	1
chr6	32522683	32522683	0.272998	0.272998	152143	146408	146408	1	1	0.04194	1	0.070801	1
chr5	1.45E+08	1.45E+08	0.272989	0.272989	145981	127600	127600	1	1	0.041944	1	0.070805	1
chr8	1.44E+08	1.44E+08	0.117571	0.235142	179916	207089	207090	2	5	0.04197	1	0.102891	1
chr5	7540955	7540955	-0.27255	0.272546	139707	114840	114840	1	1	0.042126	1	0.071094	1
chr19	58220657	58220662	0.117363	0.234727	95434	393965	393966	2	11	0.042132	1	0.103336	1
chr21	47806332	47806332	0.272482	0.272482	115854	407067	407067	1	1	0.04215	1	0.071133	1
chr5	1.77E+08	1.77E+08	0.27248	0.27248	148154	132054	132054	1	2	0.042151	1	0.071134	1
chr6	1.44E+08	1.44E+08	-0.27242	0.272415	157639	159507	159507	1	1	0.042176	1	0.071175	1
chr14	87857656	87857656	-0.27228	0.272285	57560	304396	304396	1	1	0.04223	1	0.071261	1
chr15	41096921	41096921	0.272181	0.272181	61405	312721	312721	1	1	0.042274	1	0.07133	1
chr20	62405407	62405407	0.272151	0.272151	114072	403196	403196	1	1	0.042287	1	0.071349	1
chr9	1.24E+08	1.24E+08	-0.2721	0.272097	183511	213532	213532	1	1	0.04231	1	0.071384	1
chr8	1.35E+08	1.35E+08	-0.27209	0.272087	179123	205336	205336	1	1	0.042314	1	0.071391	1
chr16	67062934	67062946	0.117113	0.234226	71653	335251	335252	2	10	0.042328	1	0.103881	1
chr1	15853252	15853263	0.117111	0.234222	3141	7410	7411	2	7	0.04233	1	0.103886	1
chr11	75946452	75946680	0.117071	0.234142	35572	256473	256474	2	10	0.042362	1	0.103974	1
chr1	6531727	6531813	-0.11706	0.234127	1767	4749	4750	2	9	0.042367	1	0.103989	1
chr2	27301649	27301943	0.117045	0.23409	97793	45724	45725	2	6	0.042381	1	0.104027	1
chr15	23158338	23158623	-0.11703	0.234067	60097	309997	309998	2	3	0.04239	1	0.104051	1
chr2	62521325	62521430	-0.11702	0.234047	99794	49650	49651	2	2	0.042398	1	0.104072	1
chr12	1.31E+08	1.31E+08	-0.11701	0.234023	48167	283800	283801	2	3	0.042409	1	0.104099	1
chr1	1.48E+08	1.48E+08	-0.27183	0.271835	11334	25490	25490	1	1	0.042419	1	0.071556	1
chr1	58898672	58898793	-0.11685	0.233698	8047	18418	18419	2	2	0.042532	1	0.104447	1
chr1	3335858	3335858	-0.27151	0.271507	1188	3358	3358	1	1	0.042558	1	0.071774	1
chr6	1.7E+08	1.7E+08	0.27146	0.27146	159682	163809	163809	1	6	0.042582	1	0.071808	1
chr13	52027382	52027388	0.116764	0.233529	51105	290379	290380	2	13	0.042596	1	0.104631	1
chr3	1.81E+08	1.81E+08	-0.27136	0.271362	128641	91098	91098	1	5	0.042622	1	0.071872	1
chr3	1.84E+08	1.84E+08	-0.27127	0.271268	128814	91481	91481	1	1	0.042664	1	0.071935	1
chr11	96074944	96074944	0.271249	0.271249	36602	258387	258387	1	1	0.042672	1	0.071947	1
chr1	19536541	19536960	0.116561	0.233121	3767	8652	8653	2	8	0.042758	1	0.10508	1
chr6	1.17E+08	1.17E+08	0.116556	0.233112	156376	157114	157115	2	4	0.042763	1	0.105092	1
chr2	2.31E+08	2.31E+08	0.271029	0.271029	108799	67785	67785	1	10	0.042768	1	0.0721	1
chr2	1.5E+08	1.5E+08	-0.11653	0.23306	104627	59362	59363	2	17	0.042784	1	0.105148	1
chr2	1.73E+08	1.73E+08	-0.27096	0.270958	105710	61456	61456	1	1	0.042799	1	0.07215	1
chr20	42544792	42544794	0.116486	0.232973	112526	399422	399423	2	20	0.042819	1	0.105245	1
chr19	2819672	2819672	0.270784	0.270784	87851	374477	374477	1	13	0.042877	1	0.072269	1
chr1	1.77E+08	1.77E+08	-0.27077	0.270774	13917	31484	31484	1	1	0.042882	1	0.072276	1
chr10	1.28E+08	1.28E+08	0.270678	0.270678	27251	235429	235429	1	1	0.042923	1	0.07234	1
chr5	1.7E+08	1.7E+08	-0.11635	0.232694	147343	130251	130252	2	10	0.042925	1	0.10555	1
chr3	52739927	52739975	-0.11624	0.232481	122646	79600	79601	2	14	0.043008	1	0.105784	1
chr18	44547161	44547161	-0.27048	0.27048	85699	369560	369560	1	1	0.043008	1	0.072473	1

chr12	1.31E+08	1.31E+08	-0.11618	0.232351	48145	283740	283741	2	10	0.043058	1	0.105929	1
chr2	2.01E+08	2.01E+08	0.270351	0.270351	106955	64125	64125	1	1	0.043064	1	0.07256	1
chr11	35611044	35611044	-0.27033	0.270327	31615	246788	246788	1	1	0.043073	1	0.072575	1
chr10	1.02E+08	1.02E+08	-0.27031	0.270308	25256	230899	230899	1	1	0.043082	1	0.072589	1
chr7	43151725	43151725	0.270304	0.270304	163697	173444	173444	1	15	0.043084	1	0.072592	1
chr3	1.84E+08	1.84E+08	-0.27028	0.270285	128942	91856	91856	1	2	0.043093	1	0.072605	1
chr12	1.31E+08	1.31E+08	-0.27027	0.270268	48169	283804	283804	1	1	0.043101	1	0.072616	1
chr19	8619925	8619925	-0.27024	0.270237	89092	377456	377456	1	1	0.043114	1	0.072637	1
chr5	434998	434998	0.270167	0.270167	138768	112354	112354	1	2	0.043145	1	0.072685	1
chr11	66311334	66311348	0.116059	0.232119	34124	252936	252937	2	3	0.043147	1	0.106194	1
chr1	1.09E+08	1.09E+08	-0.27012	0.270125	10040	22561	22561	1	1	0.043165	1	0.072715	1
chr4	94759825	94759825	-0.26999	0.269985	134917	104671	104671	1	1	0.043224	1	0.072808	1
chr1	1.52E+08	1.52E+08	0.115916	0.231832	11794	26645	26646	2	7	0.043258	1	0.106517	1
chr10	71087979	71088038	0.115913	0.231826	22838	225991	225992	2	5	0.043261	1	0.106525	1
chr19	35759696	35759698	-0.11589	0.23178	91598	384089	384090	2	4	0.043278	1	0.106576	1
chr12	1.31E+08	1.31E+08	-0.11584	0.231674	48155	283771	283772	2	7	0.043318	1	0.106695	1
chr2	1.03E+08	1.03E+08	0.26976	0.26976	102241	54892	54892	1	6	0.043321	1	0.072959	1
chr1	1.8E+08	1.8E+08	-0.26975	0.269748	14068	31784	31784	1	15	0.043326	1	0.072966	1
chr4	6050440	6050460	0.115791	0.231582	131358	97477	97478	2	2	0.043353	1	0.1068	1
chr12	4919138	4919230	0.115784	0.231568	39955	265404	265405	2	10	0.043358	1	0.106816	1
chr5	1.45E+08	1.45E+08	0.115748	0.231496	145974	127588	127589	2	13	0.043385	1	0.106896	1
chr7	27170388	27170394	0.115737	0.231473	162548	171121	171122	2	27	0.043393	1	0.106921	1
chr1	19971790	19971792	0.115673	0.231346	3848	8819	8820	2	3	0.04344	1	0.10706	1
chr11	2293171	2293173	-0.11553	0.231053	29224	241340	241341	2	44	0.043552	1	0.107394	1
chr7	2071723	2071723	-0.26913	0.269129	160737	166824	166824	1	1	0.043591	1	0.073384	1
chr3	1.3E+08	1.3E+08	-0.26891	0.268913	126295	86409	86409	1	1	0.043686	1	0.073533	1
chr2	2.42E+08	2.42E+08	-0.11532	0.230632	110336	71111	71112	2	10	0.043711	1	0.107875	1
chr1	1.15E+08	1.15E+08	-0.11528	0.230557	10606	23934	23935	2	3	0.043738	1	0.107957	1
chr7	1.56E+08	1.56E+08	0.115267	0.230534	170930	188761	188762	2	2	0.043746	1	0.10798	1
chr2	2.39E+08	2.39E+08	0.268727	0.268727	109797	69869	69869	1	17	0.043766	1	0.073662	1
chr17	80735406	80735406	0.268664	0.268664	84466	366392	366392	1	2	0.043792	1	0.073703	1
chr6	29943414	29943425	0.115207	0.230413	151460	140306	140307	2	26	0.043793	1	0.108119	1
chr2	2.21E+08	2.21E+08	-0.11516	0.230323	108360	67012	67013	2	2	0.043829	1	0.108225	1
chr13	21750649	21750649	0.268541	0.268541	49136	286332	286332	1	12	0.04385	1	0.073793	1
chr10	1.35E+08	1.35E+08	-0.11511	0.230216	28405	238817	238818	2	4	0.04387	1	0.108347	1
chr10	82368399	82368399	-0.26849	0.268492	24008	228181	228181	1	1	0.043872	1	0.073828	1
chr11	75273205	75273341	0.115103	0.230207	35504	256338	256339	2	5	0.043873	1	0.108357	1
chr1	54763942	54763942	0.268462	0.268462	7727	17897	17897	1	1	0.043886	1	0.073848	1
chr8	38032157	38032157	-0.26845	0.268454	174634	196717	196717	1	1	0.043889	1	0.073853	1
chr22	39527583	39527583	0.268368	0.268368	117787	411559	411559	1	1	0.043927	1	0.073913	1
chr1	1.56E+08	1.56E+08	0.268181	0.268181	12385	28243	28243	1	1	0.044012	1	0.074045	1
chr1	44114346	44114355	0.114919	0.229838	6809	15672	15673	2	2	0.044015	1	0.108778	1
chr5	1.26E+08	1.26E+08	0.268093	0.268093	144361	123721	123721	1	4	0.04405	1	0.074107	1
chr2	27529788	27530153	0.114857	0.229715	97849	45879	45880	2	16	0.04406	1	0.108917	1
chr6	14524587	14524587	0.268059	0.268059	150053	136055	136055	1	1	0.044064	1	0.074129	1
chr1	8021749	8021811	-0.1148	0.229596	2007	5241	5242	2	9	0.044104	1	0.109051	1
chr3	27674461	27674472	-0.1148	0.229592	120603	74814	74815	2	4	0.044106	1	0.109056	1
chr12	29778034	29778034	-0.26796	0.26796	41533	268767	268767	1	1	0.044108	1	0.0742	1
chr6	27782628	27782658	0.114709	0.229419	151064	138281	138282	2	10	0.044171	1	0.109255	1
chr5	1868261	1868279	0.114654	0.229307	139286	113820	113821	2	6	0.044212	1	0.109382	1
chr12	1.34E+08	1.34E+08	0.267727	0.267727	48883	285762	285762	1	9	0.044214	1	0.074362	1
chr6	32362744	32362744	0.267681	0.267681	152104	146294	146294	1	14	0.044236	1	0.074395	1
chr14	1.06E+08	1.06E+08	0.267677	0.267677	59834	309509	309509	1	10	0.044238	1	0.074398	1
chr2	1.72E+08	1.72E+08	0.114572	0.229145	105609	61192	61193	2	11	0.044274	1	0.109566	1
chr10	1.02E+08	1.02E+08	0.114556	0.229112	25219	230793	230794	2	4	0.044286	1	0.109603	1
chr16	48644408	48644449	0.114473	0.228947	70552	332932	332933	2	9	0.044348	1	0.109794	1
chr7	6568678	6568678	-0.26741	0.267407	161600	169078	169078	1	2	0.044363	1	0.07459	1
chr6	74007703	74007703	0.267401	0.267401	154736	153600	153600	1	1	0.044365	1	0.074594	1
chr12	1.14E+08	1.14E+08	0.267361	0.267361	46511	279910	279910	1	1	0.044384	1	0.074624	1
chr8	1.45E+08	1.45E+08	0.267302	0.267302	180104	207609	207609	1	1	0.044413	1	0.074667	1
chr8	1.41E+08	1.41E+08	-0.26726	0.267264	179264	205568	205568	1	4	0.04443	1	0.074694	1
chr16	88296994	88296994	0.267259	0.267259	74007	340476	340476	1	3	0.044432	1	0.074697	1
chr6	30432583	30433036	-0.11435	0.228707	151583	141393	141394	2	34	0.044441	1	0.110081	1
chr7	193262	193580	0.114342	0.228684	160028	164657	164658	2	6	0.044449	1	0.110108	1
chr4	7812988	7812988	0.267189	0.267189	131660	98161	98161	1	6	0.044462	1	0.074745	1
chr7	1.56E+08	1.56E+08	-0.11432	0.22864	170849	188580	188581	2	3	0.044466	1	0.11016	1
chr16	33965322	33965435	-0.11431	0.228621	70337	332505	332506	2	3	0.044474	1	0.110182	1
chr20	34297200	34297200	-0.26713	0.267135	112172	398397	398397	1	1	0.044486	1	0.074783	1
chr19	1009642	1009642	-0.26709	0.267088	87160	372793	372793	1	5	0.044506	1	0.074816	1
chr19	45147056	45147078	-0.11424	0.228483	92992	387728	387729	2	9	0.044525	1	0.11035	1
chr12	77174623	77174623	0.267009	0.267009	44558	275861	275861	1	1	0.044543	1	0.074873	1
chr7	1.58E+08	1.58E+08	-0.1142	0.228408	171378	190037	190038	2	5	0.044551	1	0.110436	1
chr8	1938530	1938530	0.266886	0.266886	172174	191930	191930	1	1	0.044599	1	0.07496	1
chr14	94491763	94491763	-0.26686	0.266858	58133	305528	305528	1	2	0.044611	1	0.074979	1

chr20	48526714	48526714	0.266831	0.266831	112980	400581	400581	1	1	0.044621	1	0.074996	1
chr12	3865073	3865073	-0.26682	0.266822	39868	265171	265171	1	1	0.044625	1	0.075003	1
chr1	28560936	28560936	-0.26677	0.266772	5054	11658	11658	1	1	0.044648	1	0.075038	1
chr7	64328759	64328759	-0.26671	0.266709	164860	175797	175797	1	2	0.044677	1	0.075083	1
chr13	1.03E+08	1.03E+08	-0.26656	0.266565	52531	293176	293176	1	10	0.044744	1	0.075187	1
chr5	1.41E+08	1.41E+08	-0.11389	0.227783	145641	126978	126979	2	6	0.04478	1	0.11117	1
chr5	1.33E+08	1.33E+08	-0.26647	0.266473	144726	124499	124499	1	12	0.044783	1	0.075248	1
chr12	1.14E+08	1.14E+08	0.266472	0.266472	46561	279984	279984	1	4	0.044784	1	0.075249	1
chr8	1957168	1957168	0.26643	0.26643	172183	191949	191949	1	2	0.044802	1	0.075277	1
chr4	10042842	10042842	-0.26631	0.266306	131924	98723	98723	1	4	0.044859	1	0.075367	1
chr19	10405083	10405226	-0.11371	0.227425	89297	378010	378011	2	2	0.044916	1	0.111593	1
chr12	1.31E+08	1.31E+08	-0.26616	0.26616	48160	283785	283785	1	1	0.044927	1	0.075471	1
chr6	1.07E+08	1.07E+08	-0.26616	0.266159	155796	155952	155952	1	1	0.044927	1	0.075472	1
chr8	38759261	38759261	0.266134	0.266134	174713	196921	196921	1	9	0.044939	1	0.07549	1
chr6	1.26E+08	1.26E+08	-0.26611	0.266113	156701	157768	157768	1	1	0.044949	1	0.075505	1
chr12	6165816	6166028	0.113626	0.227252	40042	265571	265572	2	4	0.044981	1	0.111803	1
chr12	57472555	57472580	0.113375	0.226751	43545	273702	273703	2	11	0.04516	1	0.112392	1
chr1	3828901	3828901	-0.26564	0.265642	1406	3950	3950	1	2	0.045162	1	0.075837	1
chr13	1.14E+08	1.14E+08	0.113324	0.226647	53524	295584	295585	2	5	0.045197	1	0.112516	1
chr8	30890980	30891002	-0.11331	0.226623	174347	196168	196169	2	31	0.045206	1	0.112547	1
chr13	1.15E+08	1.15E+08	0.265523	0.265523	53791	296427	296427	1	8	0.045216	1	0.075922	1
chr3	1.83E+08	1.83E+08	-0.11329	0.226575	128758	91354	91355	2	2	0.045223	1	0.112602	1
chr2	43328122	43328266	0.113282	0.226564	98793	47657	47658	2	6	0.045228	1	0.112616	1
chr12	69324962	69324962	0.265463	0.265463	44230	275219	275219	1	1	0.045245	1	0.075966	1
chr17	79140481	79140486	-0.11324	0.226475	83901	364648	364649	2	4	0.04526	1	0.112718	1
chr17	59940892	59940900	0.113228	0.226455	81495	358784	358785	2	8	0.045266	1	0.11274	1
chr5	1.4E+08	1.4E+08	-0.11322	0.226437	145488	126373	126374	2	7	0.045273	1	0.112761	1
chr6	32608095	32608095	0.265332	0.265332	152157	146435	146435	1	1	0.045307	1	0.07606	1
chr11	1.08E+08	1.08E+08	0.11316	0.22632	36960	259105	259106	2	44	0.045316	1	0.112904	1
chr6	1.67E+08	1.67E+08	0.265274	0.265274	159232	162744	162744	1	1	0.045334	1	0.076101	1
chr2	98340425	98340511	-0.11311	0.226224	101814	54098	54099	2	5	0.045348	1	0.113016	1
chr15	40268687	40268777	0.1131	0.2262	61256	312344	312345	2	5	0.045356	1	0.113046	1
chr7	66022277	66022277	0.26518	0.26518	165012	176128	176128	1	5	0.045377	1	0.076168	1
chr1	40204285	40204330	-0.11304	0.226089	6322	14556	14557	2	7	0.045396	1	0.113178	1
chr6	33037690	33037690	0.265016	0.265016	152241	147166	147166	1	3	0.045455	1	0.076289	1
chr19	12605925	12605925	0.264989	0.264989	89697	379144	379144	1	5	0.045468	1	0.076309	1
chr4	1.74E+08	1.74E+08	-0.26496	0.264958	137726	110153	110153	1	10	0.045482	1	0.076331	1
chr19	7895127	7895133	-0.11292	0.225846	88950	377093	377094	2	10	0.045482	1	0.113468	1
chr20	13200929	13200931	0.112889	0.225779	111305	396121	396122	2	31	0.045507	1	0.113551	1
chr6	17016226	17016303	0.11287	0.225741	150283	136410	136411	2	3	0.045522	1	0.1136	1
chr11	1.24E+08	1.24E+08	0.112842	0.225684	38379	262176	262177	2	10	0.045542	1	0.113666	1
chr2	2.41E+08	2.41E+08	0.11263	0.225259	110167	70734	70735	2	4	0.045692	1	0.114185	1
chr6	31148606	31148606	-0.26449	0.264492	151763	142808	142808	1	13	0.045698	1	0.076668	1
chr14	78108523	78108822	0.11254	0.225079	57316	303992	303993	2	3	0.045755	1	0.114401	1
chr18	10488229	10488229	-0.26426	0.264256	84919	367655	367655	1	1	0.045812	1	0.076841	1
chr17	7255624	7255736	-0.11244	0.224878	76004	345583	345584	2	8	0.045827	1	0.114647	1
chr6	41888715	41888869	-0.11237	0.224734	153345	150818	150819	2	12	0.04588	1	0.114821	1
chr10	3150898	3150898	0.264076	0.264076	19285	218879	218879	1	2	0.045893	1	0.076972	1
chr19	44576272	44576295	-0.11233	0.224668	92941	387572	387573	2	12	0.045902	1	0.114902	1
chr6	52454298	52454298	0.264054	0.264054	154162	152551	152551	1	1	0.045903	1	0.076987	1
chr16	30960279	30960301	0.112269	0.224539	70064	331877	331878	2	9	0.045948	1	0.115064	1
chr4	1.69E+08	1.69E+08	-0.11223	0.224453	137511	109762	109763	2	11	0.045977	1	0.115167	1
chr17	80043349	80043349	0.263855	0.263855	84225	365726	365726	1	2	0.045998	1	0.077133	1
chr2	3381687	3381687	0.263804	0.263804	96112	42365	42365	1	14	0.046021	1	0.07717	1
chr20	57617431	57617431	-0.26372	0.263719	113398	401736	401736	1	8	0.046061	1	0.077232	1
chr13	1.14E+08	1.14E+08	-0.26351	0.263515	53648	295930	295930	1	1	0.046157	1	0.077386	1
chr10	1.34E+08	1.34E+08	-0.2635	0.263502	27991	237422	237422	1	4	0.046163	1	0.077395	1
chr6	28601440	28601443	0.111932	0.223863	151211	138886	138887	2	12	0.046183	1	0.115892	1
chr5	1.34E+08	1.34E+08	-0.26339	0.263385	144846	124813	124813	1	1	0.046216	1	0.07748	1
chr8	70855057	70855146	0.11186	0.223719	176167	199936	199937	2	3	0.046232	1	0.116067	1
chr3	1.08E+08	1.08E+08	0.263255	0.263255	124707	83260	83260	1	1	0.046279	1	0.077578	1
chr19	33793141	33793146	0.111661	0.223322	91418	383594	383595	2	6	0.046369	1	0.116557	1
chr7	27143717	27143806	-0.11166	0.223319	162536	171039	171040	2	35	0.04637	1	0.116561	1
chr13	27999177	27999177	0.263043	0.263043	49601	287289	287289	1	10	0.046386	1	0.077741	1
chr6	1.39E+08	1.39E+08	0.262708	0.262708	157422	159137	159137	1	1	0.046549	1	0.077988	1
chr5	1.41E+08	1.41E+08	0.262408	0.262408	145671	127067	127067	1	1	0.046697	1	0.078215	1
chr11	1.07E+08	1.07E+08	-0.26239	0.262395	36920	258987	258987	1	17	0.046704	1	0.078225	1
chr5	1.42E+08	1.42E+08	0.262234	0.262234	145807	127337	127337	1	1	0.046785	1	0.078349	1
chr1	1.6E+08	1.6E+08	0.110913	0.221826	12772	29068	29069	2	11	0.046874	1	0.118414	1
chr16	2908219	2908245	-0.11086	0.221728	67595	326527	326528	2	11	0.046907	1	0.118536	1
chr2	27434617	27434710	0.110785	0.22157	97830	45806	45807	2	19	0.04696	1	0.118734	1
chr19	55587193	55587193	0.2617	0.2617	94982	392717	392717	1	5	0.04704	1	0.078751	1
chr19	53326091	53326091	-0.26169	0.26169	94585	391724	391724	1	3	0.047045	1	0.078758	1
chr4	1.64E+08	1.64E+08	-0.26167	0.26167	137385	109474	109474	1	1	0.047055	1	0.078772	1

chr8	1400221	1400221	0.261637	0.261637	171990	191447	191447	1	1	0.047072	1	0.078799	1
chr16	29625216	29625225	0.110565	0.22113	69763	330989	330990	2	6	0.047107	1	0.119293	1
chr5	1.41E+08	1.41E+08	-0.11052	0.221036	145643	126982	126983	2	4	0.047138	1	0.119411	1
chr3	64080488	64080488	-0.26146	0.261463	123349	80930	80930	1	1	0.04716	1	0.078934	1
chr11	1.34E+08	1.34E+08	0.261421	0.261421	39244	263882	263882	1	2	0.047182	1	0.078967	1
chr17	21220141	21220411	-0.11043	0.220861	77518	349058	349059	2	4	0.047196	1	0.119639	1
chr2	1.59E+08	1.59E+08	-0.26133	0.261327	104996	60062	60062	1	1	0.047228	1	0.079037	1
chr20	61953935	61954258	0.110313	0.220626	113894	402820	402821	2	3	0.047272	1	0.119936	1
chr3	44803293	44803341	-0.11029	0.220575	121590	76891	76892	2	18	0.047286	1	0.119999	1
chr19	53541186	53541287	0.110272	0.220544	94611	391796	391797	2	4	0.047296	1	0.120037	1
chr12	1.08E+08	1.08E+08	0.261091	0.261091	45856	278377	278377	1	9	0.047344	1	0.079218	1
chr17	80542006	80542118	-0.1101	0.2202	84396	366224	366225	2	6	0.047407	1	0.120476	1
chr11	1.18E+08	1.18E+08	0.26096	0.26096	37667	260648	260648	1	1	0.047411	1	0.079318	1
chr15	69745373	69745373	0.260941	0.260941	63594	317351	317351	1	15	0.047421	1	0.079333	1
chr1	2391317	2391317	0.260918	0.260918	812	2321	2321	1	5	0.047432	1	0.079351	1
chr2	10572946	10572946	-0.26091	0.260913	96709	43544	43544	1	1	0.047434	1	0.079355	1
chr19	54211900	54211900	0.260796	0.260796	94717	392062	392062	1	2	0.047493	1	0.079443	1
chr19	47017048	47017050	-0.10993	0.219859	93372	388675	388676	2	4	0.047514	1	0.12091	1
chr17	2207236	2207236	0.260743	0.260743	75275	343639	343639	1	11	0.04752	1	0.079484	1
chr12	43203955	43203955	0.260728	0.260728	41967	269637	269637	1	1	0.047527	1	0.079494	1
chr10	52434778	52434778	-0.26064	0.260642	22201	224724	224724	1	3	0.047569	1	0.079562	1
chr10	1.34E+08	1.34E+08	-0.10983	0.219665	27976	237363	237364	2	11	0.047576	1	0.12116	1
chr12	1.29E+08	1.29E+08	0.260602	0.260602	47993	283386	283386	1	1	0.047589	1	0.079593	1
chr19	5892460	5892460	-0.26054	0.26054	88649	376332	376332	1	5	0.047621	1	0.079642	1
chr20	62552607	62552607	0.260521	0.260521	114118	403290	403290	1	1	0.047631	1	0.079657	1
chr8	1.05E+08	1.05E+08	0.260516	0.260516	177733	203022	203022	1	4	0.047633	1	0.079661	1
chr3	1.23E+08	1.23E+08	0.260508	0.260508	125467	84745	84745	1	1	0.047637	1	0.079667	1
chr17	185227	185263	0.109691	0.219382	74812	342484	342485	2	5	0.047664	1	0.121526	1
chr2	26702081	26702081	-0.26025	0.260255	97712	45522	45522	1	1	0.047769	1	0.079867	1
chr13	1.14E+08	1.14E+08	0.10939	0.21878	53645	295923	295924	2	3	0.047851	1	0.122306	1
chr17	6797466	6797708	-0.10937	0.218737	75898	345176	345177	2	8	0.047865	1	0.122364	1
chr16	89168508	89168599	-0.10934	0.218671	74371	341422	341423	2	11	0.047886	1	0.122451	1
chr14	98670848	98670848	0.260013	0.260013	58458	306192	306192	1	1	0.047896	1	0.080055	1
chr11	61735315	61735327	-0.10928	0.218552	33185	250119	250120	2	13	0.047924	1	0.12261	1
chr1	2.3E+08	2.3E+08	-0.25992	0.25992	17493	38692	38692	1	1	0.047944	1	0.080127	1
chr11	1.34E+08	1.34E+08	0.10921	0.21842	39309	264062	264063	2	8	0.047965	1	0.122783	1
chr4	57547347	57547525	0.109108	0.218216	133603	102103	102104	2	6	0.048029	1	0.123048	1
chr1	1.11E+08	1.11E+08	-0.10908	0.218166	10273	23155	23156	2	3	0.048047	1	0.123118	1
chr5	1.56E+08	1.56E+08	0.109078	0.218155	146729	129079	129080	2	4	0.04805	1	0.123132	1
chr5	1.35E+08	1.35E+08	0.259691	0.259691	144895	124911	124911	1	3	0.04806	1	0.080306	1
chr7	56019451	56019451	0.259673	0.259673	164560	175181	175181	1	11	0.048068	1	0.08032	1
chr2	1.75E+08	1.75E+08	-0.25962	0.259622	105872	61717	61717	1	24	0.048094	1	0.08036	1
chr17	13472852	13472852	-0.25953	0.259528	76664	347249	347249	1	1	0.048144	1	0.080436	1
chr21	33765622	33765737	0.10889	0.21778	114553	404252	404253	2	8	0.048163	1	0.123622	1
chr12	52545884	52545978	0.108885	0.21777	42785	271616	271617	2	5	0.048166	1	0.123636	1
chr10	50821597	50821652	0.108872	0.217743	22090	224515	224516	2	11	0.048174	1	0.12367	1
chr7	29519297	29519297	0.259414	0.259414	162788	171815	171815	1	7	0.048204	1	0.080527	1
chr7	1.5E+08	1.5E+08	-0.2594	0.259399	170217	187047	187047	1	2	0.048213	1	0.080541	1
chr8	1030319	1030521	-0.1088	0.217603	171851	191119	191120	2	2	0.048216	1	0.123855	1
chr6	1.7E+08	1.7E+08	-0.25935	0.259348	159857	164220	164220	1	5	0.048239	1	0.080581	1
chr17	62971706	62971708	0.1087	0.217399	81789	359484	359485	2	11	0.048274	1	0.124127	1
chr12	1.17E+08	1.17E+08	-0.2592	0.259195	46739	280412	280412	1	1	0.048316	1	0.080698	1
chr11	45822831	45822831	0.25916	0.25916	32049	247702	247702	1	1	0.048334	1	0.080725	1
chr12	1.05E+08	1.05E+08	-0.25914	0.259141	45616	277879	277879	1	1	0.048344	1	0.08074	1
chr2	2.41E+08	2.41E+08	-0.25912	0.259117	110068	70508	70508	1	3	0.048355	1	0.080758	1
chr5	1.41E+08	1.41E+08	-0.10855	0.217093	145588	126701	126702	2	13	0.04836	1	0.124519	1
chr12	1.11E+08	1.11E+08	-0.10853	0.217053	46235	279227	279228	2	5	0.048372	1	0.124572	1
chr8	1.43E+08	1.43E+08	0.108506	0.217013	179718	206580	206581	2	2	0.048384	1	0.124624	1
chr6	1.57E+08	1.57E+08	-0.1085	0.216999	158370	160956	160957	2	3	0.048388	1	0.124643	1
chr3	1E+08	1E+08	-0.1084	0.21681	124454	82780	82781	2	9	0.048438	1	0.124886	1
chr19	12707977	12707977	0.258923	0.258923	89706	379177	379177	1	4	0.048453	1	0.080907	1
chr16	88290370	88290370	-0.25891	0.258912	74004	340465	340465	1	2	0.048458	1	0.080916	1
chr17	7643808	7644073	0.108364	0.216728	76117	345980	345981	2	4	0.048461	1	0.124996	1
chr9	1.3E+08	1.3E+08	0.258853	0.258853	184026	214337	214337	1	1	0.048491	1	0.080964	1
chr21	46219184	46219184	0.258841	0.258841	115524	406433	406433	1	1	0.048497	1	0.080974	1
chr19	1442231	1442231	0.258805	0.258805	87346	373284	373284	1	1	0.048515	1	0.081001	1
chr11	971527	971902	0.108199	0.216399	28782	239908	239909	2	4	0.048553	1	0.125437	1
chr8	23310132	23310132	0.258695	0.258695	173736	194908	194908	1	1	0.048569	1	0.081087	1
chr4	1.88E+08	1.88E+08	-0.10815	0.216302	138454	111734	111735	2	9	0.048578	1	0.125566	1
chr6	10495364	10495435	0.108102	0.216204	149718	135433	135434	2	2	0.048608	1	0.125699	1
chr6	32769203	32769203	0.258542	0.258542	152179	146528	146528	1	1	0.048649	1	0.081209	1
chr7	17826367	17826367	-0.25851	0.258506	162010	169877	169877	1	1	0.04867	1	0.081239	1
chr6	8087916	8087916	0.258478	0.258478	149614	135190	135190	1	1	0.048686	1	0.081261	1
chr1	1.51E+08	1.51E+08	-0.10789	0.215788	11639	26301	26302	2	8	0.048727	1	0.126262	1

chr1	2.1E+08	2.1E+08	-0.25832	0.258322	15991	35592	35592	1	9	0.048766	1	0.081382	1
chr7	37484562	37484562	0.258239	0.258239	163346	172826	172826	1	1	0.048809	1	0.081446	1
chr1	19637256	19637256	-0.25822	0.258218	3788	8717	8717	1	1	0.048819	1	0.081463	1
chr13	88328471	88328813	0.107723	0.215446	51903	291901	291902	2	13	0.048819	1	0.126718	1
chr11	2323459	2323801	-0.10771	0.215412	29236	241400	241401	2	30	0.048829	1	0.126769	1
chr6	1.06E+08	1.06E+08	-0.25811	0.258112	155709	155777	155777	1	1	0.048874	1	0.081546	1
chr7	66785137	66785137	-0.25805	0.258052	165070	176258	176258	1	1	0.048906	1	0.081595	1
chr14	97493395	97493395	0.258033	0.258033	58418	306116	306116	1	1	0.048916	1	0.081611	1
chr17	45727224	45727226	-0.10753	0.215068	80173	355588	355589	2	11	0.048921	1	0.127236	1
chr6	29599319	29599331	-0.10753	0.215057	151373	139830	139831	2	33	0.048923	1	0.127247	1
chr17	72322143	72322154	0.107513	0.215026	82390	360631	360632	2	10	0.048933	1	0.127289	1
chr15	29442040	29442169	-0.10751	0.215018	60585	311123	311124	2	3	0.048934	1	0.127299	1
chr1	1.72E+08	1.72E+08	-0.10745	0.214908	13694	31065	31066	2	6	0.048962	1	0.127446	1
chr6	29524112	29524117	-0.10744	0.214889	151347	139657	139658	2	7	0.048966	1	0.127471	1
chr1	2.08E+08	2.08E+08	0.107393	0.214787	15863	35373	35374	2	6	0.048991	1	0.127607	1
chr9	1.16E+08	1.16E+08	0.257872	0.257872	183322	213262	213262	1	1	0.048998	1	0.081738	1
chr17	80394529	80394529	-0.25785	0.257853	84361	366124	366124	1	6	0.049009	1	0.081754	1
chr14	50698469	50698663	-0.10735	0.21469	55177	299651	299652	2	8	0.049016	1	0.127739	1
chr12	1.09E+08	1.09E+08	-0.25777	0.257773	45912	278504	278504	1	1	0.049051	1	0.081818	1
chr8	43136327	43136445	-0.10718	0.214356	175050	197624	197625	2	3	0.049104	1	0.12819	1
chr11	1859381	1859381	-0.25763	0.257629	29082	240784	240784	1	13	0.049124	1	0.08193	1
chr10	1.25E+08	1.25E+08	-0.10711	0.21423	26914	234688	234689	2	10	0.049136	1	0.128359	1
chr8	1.1E+08	1.1E+08	-0.10711	0.214224	177896	203300	203301	2	3	0.049137	1	0.128366	1
chr6	1.08E+08	1.08E+08	0.257591	0.257591	155883	156113	156113	1	1	0.049144	1	0.081959	1
chr20	44682963	44682963	0.257549	0.257549	112768	400086	400086	1	1	0.049165	1	0.081993	1
chr6	1.34E+08	1.34E+08	0.107001	0.214001	157040	158386	158387	2	43	0.049191	1	0.128667	1
chr11	63852858	63852887	-0.10696	0.213927	33483	250969	250970	2	5	0.049209	1	0.128765	1
chr1	94559714	94559714	-0.25731	0.257314	9642	21761	21761	1	1	0.049293	1	0.082182	1
chr6	95220699	95221182	0.106744	0.213487	155425	155084	155085	2	2	0.049317	1	0.129369	1
chr8	40618425	40618425	0.257255	0.257255	174780	197050	197050	1	1	0.049324	1	0.082228	1
chr10	1.34E+08	1.34E+08	0.106713	0.213426	27889	237112	237113	2	2	0.049332	1	0.129454	1
chr10	22609629	22609897	0.106683	0.213367	20516	221431	221432	2	12	0.049347	1	0.129535	1
chr20	23018108	23018108	-0.25717	0.257172	111576	396891	396891	1	2	0.049367	1	0.082292	1
chr17	77136834	77136834	-0.25716	0.257162	83368	363149	363149	1	1	0.049372	1	0.0823	1
chr4	1.07E+08	1.07E+08	-0.25715	0.257154	135284	105405	105405	1	20	0.049377	1	0.082308	1
chr10	1.3E+08	1.3E+08	0.10645	0.212901	27383	235645	235646	2	11	0.049459	1	0.130175	1
chr6	32729764	32729823	-0.10637	0.212743	152173	146498	146499	2	28	0.049498	1	0.13039	1
chr4	1.23E+08	1.23E+08	0.106325	0.21265	135887	106627	106628	2	12	0.04952	1	0.130522	1
chr1	1074333	1074333	0.256828	0.256828	186	524	524	1	2	0.049555	1	0.082574	1
chr19	44764205	44764380	0.106164	0.212329	92957	387648	387649	2	6	0.049597	1	0.130964	1
chr16	81518122	81518253	-0.10616	0.212317	72817	337888	337889	2	4	0.0496	1	0.130981	1
chr11	33097876	33097876	0.256677	0.256677	31395	246372	246372	1	1	0.049636	1	0.082699	1
chr19	13319417	13319523	0.106015	0.21203	89897	379740	379741	2	5	0.049667	1	0.131382	1
chr19	3059753	3059753	0.256603	0.256603	87908	374607	374607	1	1	0.049676	1	0.082759	1
chr19	56042539	56042539	-0.2566	0.256599	95120	393087	393087	1	4	0.049679	1	0.082764	1
chr8	1.45E+08	1.45E+08	0.256553	0.256553	180228	207951	207951	1	3	0.049703	1	0.0828	1
chr7	27183133	27183196	0.105932	0.211864	162555	171155	171156	2	51	0.049706	1	0.13162	1
chr17	1808573	1808671	-0.10589	0.211771	75204	343431	343432	2	2	0.049726	1	0.131748	1
chr8	1.44E+08	1.44E+08	0.105872	0.211744	179947	207159	207160	2	10	0.049731	1	0.131785	1
chr9	1.18E+08	1.18E+08	0.256403	0.256403	183420	213400	213400	1	1	0.049782	1	0.082919	1
chr8	1789267	1789288	0.105734	0.211469	172110	191748	191749	2	6	0.049794	1	0.132178	1
chr7	27196153	27196286	0.105457	0.210914	162560	171246	171247	2	25	0.049914	1	0.132947	1
chr7	45431839	45431891	0.10542	0.21084	163955	174089	174090	2	3	0.049931	1	0.133051	1
chr4	87281412	87281434	0.105419	0.210839	134650	104138	104139	2	5	0.049931	1	0.133053	1
chr2	1.69E+08	1.69E+08	0.256126	0.256126	105397	60787	60787	1	5	0.049932	1	0.083148	1
chr7	42896801	42896970	0.105401	0.210802	163678	173394	173395	2	3	0.049939	1	0.133108	1
chr1	6845440	6845440	0.2561	0.2561	1835	4943	4943	1	12	0.049948	1	0.08317	1
chr6	29715062	29715062	-0.25608	0.256078	151415	140043	140043	1	3	0.049959	1	0.083188	1
chr21	47547796	47547796	-0.25607	0.25607	115808	406970	406970	1	1	0.049963	1	0.083195	1
chr6	32526414	32526414	-0.25607	0.256068	152144	146414	146414	1	6	0.049964	1	0.083197	1
chr7	21209509	21209725	-0.10531	0.210617	162144	170183	170184	2	7	0.049977	1	0.133369	1
chr22	31090071	31090071	0.25602	0.25602	117132	410001	410001	1	7	0.049991	1	0.083239	1
