# Supplementary material for: Epigenetic signature of preterm birth in adult twins
Source: Clin Epigenetics. 2018 Jun 27;10:87. doi: 10.1186/s13148-018-0518-8 (PMC6020425; doi:10.1186/s13148-018-0518-8)
Supplement: Supplementary file 5 — Table S3. CpGs under top DMRs. (PDF 71 kb) [file 13148_2018_518_MOESM5_ESM.pdf]

IlmnID	Basepair	discoverE	Std. Error	t value	Pr(>|t|)	replicateE	Std. Error	t value	Pr(>|t|)	Infinium_D	Color_Cha	CHR	MAPINFO	Strand	UCSC_Ref	UCSC_Ref	UCSC_Ref	UCSC_CpG	Relation_t	HMM_Isla	UCSC_RefGene
cg2647263	1594282	-0.33343	0.225765	-1.47687	0.141834	-0.14991	0.149043	-1.00585	0.315188	I	Red	5	1594282	F	SDHAP3	NR_00326	Body	chr5:1594	Island	5:1647215	Body
cg2714907	1594330	-0.43031	0.267364	-1.60944	0.109651	-0.18679	0.196473	-0.95069	0.34242	II	NA	5	1594330	F	SDHAP3	NR_00326	Body	chr5:1594	Island	5:1647215	Body
cg0877859	1594579	-0.62904	0.366378	-1.71691	0.088088	-0.23104	0.273232	-0.84558	0.39837	I	Red	5	1594579	F	SDHAP3	NR_00326	Body	chr5:1594	Island	5:1647215	Body
cg2496096	1594678	-0.53081	0.305643	-1.73669	0.084522	-0.10056	0.193307	-0.5202	0.603258	I	Grn	5	1594678	R	SDHAP3	NR_00326	TSS200	chr5:1594	Island	5:1647215	TSS200
cg2193171	1594715	-0.52261	0.336045	-1.55518	0.122039	-0.1598	0.21755	-0.73455	0.463109	I	Grn	5	1594715	R	SDHAP3	NR_00326	TSS200	chr5:1594	Island	5:1647215	TSS200
cg0842242	1594733	-0.66446	0.359006	-1.85083	0.066187	-0.18348	0.24367	-0.75298	0.45197	I	Grn	5	1594733	R	SDHAP3	NR_00326	TSS200	chr5:1594	Island	5:1647215	TSS200
cg0672154	1594808	-0.30518	0.186915	-1.63271	0.104656	-0.11692	0.109253	-1.07014	0.285298	II	NA	5	1594808	F	SDHAP3	NR_00326	TSS200	chr5:1594	Island	5:1647215	TSS200
cg1759763	1594843	-0.17209	0.132942	-1.29447	0.19752	-0.12461	0.093231	-1.33652	0.182251	II	NA	5	1594843	R	SDHAP3	NR_00326	TSS200	chr5:1594	Island	5:1647215	TSS200
cg2737853	1594863	-0.35365	0.242185	-1.46024	0.146345	-0.15742	0.161516	-0.97464	0.330418	II	NA	5	1594863	R	SDHAP3	NR_00326	TSS1500	chr5:1594	Island	5:1647215	TSS1500
cg0852247	1.12E+08	1.899622	0.589013	3.225094	0.00155	0.891474	0.473601	1.882334	0.060625	II	NA	3	1.12E+08	R	TAGLN3;T	NM_0010	Body;Body	NA	NA	NA	Body
cg2433004	24384159	0.285554	0.128173	2.227871	0.027398	0.149445	0.116749	1.280047	0.201381	II	NA	22	24384159	R	GSTT1	NM_0008	1stExon	chr22:243	Island	22:227138	1stExon
cg1054625	24384294	0.533465	0.370071	1.441522	0.15155	0.171748	0.255939	0.671049	0.502635	II	NA	22	24384294	R	GSTT1	NM_0008	TSS200	chr22:243	Island	22:227138	TSS200
cg2006477	24384315	0.58718	0.369873	1.587517	0.11453	0.202575	0.238632	0.848898	0.396522	I	Grn	22	24384315	R	GSTT1	NM_0008	TSS200	chr22:243	Island	22:227138	TSS200
cg1525464	24384393	0.525791	0.352435	1.49188	0.137859	0.191186	0.220441	0.867288	0.386382	I	Red	22	24384393	R	GSTT1	NM_0008	TSS200	chr22:243	Island	22:227138	TSS200
cg2433683	24384397	0.70032	0.390915	1.791489	0.075258	0.16782	0.246537	0.68071	0.496508	I	Red	22	24384397	R	GSTT1	NM_0008	TSS200	chr22:243	Island	22:227138	TSS200
cg1147860	24384400	0.709295	0.392431	1.807438	0.072725	0.218571	0.263695	0.82888	0.40774	I	Red	22	24384400	R	GSTT1	NM_0008	TSS200	chr22:243	Island	22:227138	TSS200
cg1700506	24384525	0.527133	0.367062	1.436089	0.153087	0.097141	0.219996	0.44156	0.659082	II	NA	22	24384525	F	GSTT1	NM_0008	TSS1500	chr22:243	S_Shore	NA	TSS1500
cg0821917	24384573	0.358851	0.139353	2.575132	0.011	0.06112	0.116528	0.524508	0.600259	II	NA	22	24384573	F	GSTT1	NM_0008	TSS1500	chr22:243	S_Shore	NA	TSS1500
cg0733256	291687	-0.41991	0.365291	-1.14952	0.252197	0.320583	0.176956	1.811656	0.070901	II	NA	6	291687	R	DUSP22	NM_0201	TSS1500	chr6:2919	N_Shore	NA	TSS1500
cg2154881	291882	-0.54313	0.444371	-1.22225	0.223556	0.35634	0.223832	1.591996	0.112293	II	NA	6	291882	R	DUSP22	NM_0201	TSS1500	chr6:2919	N_Shore	6:236882-	TSS1500
cg0339551	291903	-0.50881	0.377647	-1.34732	0.179935	0.277213	0.189108	1.465901	0.143578	II	NA	6	291903	R	DUSP22	NM_0201	TSS200	chr6:2919	N_Shore	6:236882-	TSS200
cg1538312	291909	-0.49201	0.412558	-1.19259	0.234939	0.341501	0.218335	1.564116	0.118699	II	NA	6	291909	R	DUSP22	NM_0201	TSS200	chr6:2919	N_Shore	6:236882-	TSS200
cg0506404	292385	-0.38964	0.318784	-1.22228	0.223544	0.236183	0.135076	1.748515	0.081257	II	NA	6	292385	F	DUSP22;D	NM_0201	1stExon;5'	chr6:2919	Island	6:236882-	1stExon
cg1123542	292522	-0.4195	0.293981	-1.42697	0.155696	0.313438	0.17762	1.764659	0.078498	II	NA	6	292522	F	DUSP22;D	NM_0201	1stExon;5'	chr6:2919	Island	6:236882-	1stExon
cg0151688	292596	-0.45292	0.327284	-1.38388	0.168478	0.328999	0.16987	1.93677	0.053583	I	Grn	6	292596	R	DUSP22	NM_0201	Body	chr6:2919	Island	6:236882-	Body
cg2511042	41068646	-0.40412	0.3064	-1.31893	0.189228	0.310165	0.204664	1.515484	0.130558	I	Grn	6	41068646	F	NFYA;NFY	NM_0217	3'UTR;3'U	chr6:4106	Island	6:411764	3'UTR
cg0434645	41068666	-0.52114	0.417977	-1.24681	0.214437	0.515203	0.299078	1.722639	0.085842	I	Grn	6	41068666	R	NFYA;NFY	NM_0217	3'UTR;3'U	chr6:4106	Island	6:411764	3'UTR
cg0958015	41068724	-0.55953	0.404351	-1.38377	0.168513	0.370527	0.250274	1.480487	0.139648	I	Red	6	41068724	R	NFYA;NFY	NM_0217	3'UTR;3'U	chr6:4106	Island	6:411764	3'UTR
cg0667166	41068741	-0.2793	0.227081	-1.22998	0.220657	0.198854	0.09412	2.11277	0.035332	II	NA	6	41068741	R	NFYA;NFY	NM_0217	3'UTR;3'U	chr6:4106	Island	6:411764	3'UTR
cg0364428	41068752	-0.59618	0.439324	-1.35704	0.176835	0.515611	0.297692	1.732027	0.084155	I	Red	6	41068752	R	NFYA;NFY	NM_0217	3'UTR;3'U	chr6:4106	Island	6:411764	3'UTR
cg1583728	1.35E+08	0.143047	0.135907	1.052533	0.29427	0.222803	0.167274	1.331962	0.183744	II	NA	5	1.35E+08	R	NA	NA	NA	chr5:1354	Island	5:1354429	NA
cg0715850	1.35E+08	0.302207	0.192561	1.569413	0.118687	0.140799	0.27608	0.509992	0.61038	II	NA	5	1.35E+08	R	NA	NA	NA	chr5:1354	N_Shore	5:1354435	NA
cg0451520	1.35E+08	0.203481	0.143858	1.414458	0.159328	0.10104	0.198907	0.507979	0.61179	II	NA	5	1.35E+08	F	NA	NA	NA	chr5:1354	N_Shore	5:1354435	NA
cg1197888	1.35E+08	0.308678	0.270367	1.1417	0.255423	0.117061	0.196943	0.594389	0.552638	II	NA	5	1.35E+08	F	NA	NA	NA	chr5:1354	N_Shore	5:1354435	NA
cg1160815	1.35E+08	0.316596	0.235128	1.346482	0.180205	0.242801	0.304735	0.796761	0.426133	I	Red	5	1.35E+08	R	NA	NA	NA	chr5:1354	N_Shore	5:1354437	NA
cg0647888	1.35E+08	0.186434	0.141527	1.317307	0.189772	0.171139	0.194138	0.881529	0.37864	II	NA	5	1.35E+08	R	NA	NA	NA	chr5:1354	N_Shore	5:1354437	NA
cg0448192	1.35E+08	0.330455	0.239608	1.379151	0.169929	0.286619	0.314324	0.911859	0.362474	II	NA	5	1.35E+08	R	MIR886	NR_03058	Body	chr5:1354	Island	NA	Body
cg1867864	1.35E+08	0.292282	0.219264	1.333011	0.184577	0.226047	0.265009	0.85298	0.394257	II	NA	5	1.35E+08	R	MIR886	NR_03058	TSS200	chr5:1354	Island	5:1354441	TSS200
cg0653661	1.35E+08	0.391278	0.205851	1.900785	0.059274	0.242886	0.287535	0.844718	0.398849	I	Grn	5	1.35E+08	F	MIR886	NR_03058	TSS200	chr5:1354	Island	5:1354441	TSS200
cg2632863	1.35E+08	0.345429	0.208514	1.656625	0.099714	0.230579	0.293276	0.786221	0.432273	I	Red	5	1.35E+08	F	MIR886	NR_03058	TSS200	chr5:1354	Island	5:1354441	TSS200
cg2534068	1.35E+08	0.386137	0.214187	1.802804	0.073454	0.225608	0.295277	0.764055	0.445352	I	Red	5	1.35E+08	F	MIR886	NR_03058	TSS200	chr5:1354	Island	5:1354441	TSS200
cg2689694	1.35E+08	0.250214	0.140692	1.778446	0.077384	0.169547	0.197936	0.856574	0.39227	I	Grn	5	1.35E+08	F	MIR886	NR_03058	TSS200	chr5:1354	Island	5:1354441	TSS200
cg0012499	1.35E+08	0.408627	0.213293	1.915799	0.057319	0.207221	0.293072	0.707064	0.48	I	Grn	5	1.35E+08	F	MIR886	NR_03058	TSS200	chr5:1354	Island	5:1354441	TSS200
cg1661535	1.35E+08	0.237433	0.142986	1.660539	0.098923	0.216062	0.223634	0.966141	0.334645	II	NA	5	1.35E+08	F	MIR886	NR_03058	TSS1500	chr5:1354	S_Shore	NA	TSS1500
cg1879765	1.35E+08	0.258169	0.170773	1.511769	0.132725	0.183427	0.223519	0.820632	0.412418	II	NA	5	1.35E+08	F	MIR886	NR_03058	TSS1500	chr5:1354	S_Shore	NA	TSS1500
